# Exploration of the association between 91 inflammatory proteins and immune thrombocytopenia: a two-sample Mendelian randomization analysis

**DOI:** 10.1007/s12185-025-03987-1

**Published:** 2025-04-22

**Authors:** Aihua Qin, Dan Liu, Heshan Tang, Jinqi Li, Liling Qiu, Baohua Qian, Yan Zang

**Affiliations:** 1 Department of Transfusion Medicine, The First Affiliated Hospital of Naval Medical University, Yangpu District, 168 Changhai Road, Shanghai, People’s Republic of China; 2 Department of Emergency, Shanghai Tenth People’s Hospital, Tongji University School of Medicine, Shanghai, People’s Republic of China

**Keywords:** Immune thrombocytopenia, Inflammatory proteins, Mendelian randomization

## Abstract

**Objective:**

This study aimed to explore the association between 91 circulating inflammatory proteins and immune thrombocytopenia (ITP) using Mendelian randomization (MR).

**Methods:**

Data from genome-wide association studies (GWAS) on 91 inflammatory proteins were aggregated from the Olink Target platform, involving 14,824 participants. ITP data were sourced from the Integrative Epidemiology Unit OPEN GWAS project, which included 675 ITP patients and 488,749 controls. Mendelian randomization analysis was primarily conducted using inverse-variance weighting (IVW), supplemented by MR-Egger, weighted median, simple mode, and weighted mode. Pleiotropy and heterogeneity of the instrumental variables were assessed using the MR-Egger-intercept test and Cochran’s *Q* test, with results visualized through scatter plots, funnel plots, and leave-one-out plots.

**Results:**

The IVW method indicated an association between six specific circulating inflammatory proteins and ITP. Four proteins (CCL4, CXCL9, IL-12B, and SCF) were positively associated with ITP, while two proteins (IL-1α, TRANCE) showed a negative correlation.

**Conclusion:**

The findings suggest a potential link between circulating inflammatory proteins and ITP, providing insights for future therapeutic strategies and biomarker identification.

**Supplementary Information:**

The online version contains supplementary material available at 10.1007/s12185-025-03987-1.

## Introduction

Immune thrombocytopenia (ITP) is an autoimmune condition characterized by reduced platelet counts and an elevated bleeding risk [1, 2]. ITP can affect individuals of any age, with a particularly high prevalence among women of reproductive age and individuals over 60 years. Recent epidemiological data suggest that the annual incidence of ITP in adults ranges from 3.3 to 39 cases per 100,000, while in children, the incidence varies from 1.9 to 64 per 100,000 [3].

The pathogenesis of immune thrombocytopenia (ITP) is intricate and remains incompletely understood. It is increasingly recognized that various immune-related mechanisms contribute to its development, including humoral and cellular immunity, genetic predisposition, abnormal gene expression, and infections [4, 5]. Inflammatory cytokines are critical molecules that facilitate immune responses and significantly influence the pathogenesis of autoimmune disorders [6]. Cytokine secretion and regulation are crucial for maintaining immune system balance. However, the relationship between the circulating inflammatory proteins and ITP and its potential mechanisms have not been systematically studied.

Mendelian randomization (MR) is a method that utilizes genetic variants linked to particular exposures to establish causal relationships between risk factors and disease outcomes [7]. This approach, which is based on the random distribution of genetic variants during meiosis, helps reduce confounding factors and biases that may arise from environmental or behavioral influences [8]. It is instrumental in addressing the limitations of conventional observational studies, especially for rare diseases like ITP [9]. Using public GWAS database summary statistics, this study investigated the relationship between 91 kinds of circulating inflammatory proteins and ITP through MR analysis.

## Materials and methods

### Research design

The study adhered to the STROBE-MR guidelines for reporting Mendelian randomization studies [10]. MR is based on three essential assumptions: (1) the instrumental variables (IVs) must have a strong association with the exposure being examined, (2) the IVs should not be associated with any confounding variables that could influence the relationship between the genetic variants and the outcome of interest, and (3) the IVs should only affect the outcome through their association with the exposure, without involving any alternative pathways [8].

### Data source

The datasets for 91 circulating inflammatory proteins were collected from 11 cohorts, encompassing a total of 14,824 participants. The Olink Inflammation Panel was utilized to assess both whole-genome genetic data and plasma proteomic data. This included a genome-wide study of protein quantitative trait loci (pQTLs) for the 91 plasma proteins among the 14,824 participants, with plasma protein concentrations determined using the Olink Target-96 Inflammation Immune Analysis panel. The proteomic data for each cohort were generated at the Olink laboratory in Uppsala [11]. Comprehensive summary statistics for the protein GWAS are available for download at https://www.phpc.cam.ac.uk/ceu/proteins and on the EBI GWAS directory (GCST90274758–GCST90274848). Summary statistics for the GWAS on immune thrombocytopenia (ITP) were derived from the dataset released by the IEU OPEN GWAS project, which included genetic data from 675 ITP patients and 488,749 control individuals (Dataset: ebi-a-GCST90018865). There was no overlap in population selection between the exposure group and the outcome group, and all included populations were of European descent, fulfilling the requirement that both samples in Mendelian randomization (MR) be from the same genetic background. Supplementary Table 1 summarizes the GWAS details for the 91 circulating inflammatory proteins included in the study. Supplementary Table 2 provides information on all single-nucleotide polymorphisms (SNPs) used as instrumental variables (IVs). Our research methods and reporting adhere to the STROBE-MR guidelines, as detailed in Supplementary Table 3. All datasets utilized in this study were obtained from publicly available GWAS databases and did not require ethical approval.

### Instrumental variable selection

Series of quality assurance procedures were implemented to ensure the reliability of the conclusions drawn regarding the relationship between the 91 circulating inflammatory proteins and ITP risk. First, in accordance with the standard methodology employed in the majority of MR investigations that focus on the role of circulating inflammatory proteins [12, 13]. A significance threshold of *p* < 5 × 10^–6^ was set to identify an adequate number of instrumental variables. This decision was based on the relatively small number of identified loci for circulating inflammatory proteins [14]. Second, single-nucleotide polymorphisms (SNPs) that were associated with confounders or outcomes with an *R*^2^ > 0.001 were excluded to prevent linkage disequilibrium (LD) within 10,000 kb. Potential confounding factors associated with the selected SNPs were examined to ensure compliance with the MR exclusion assumptions, and SNPs whose corresponding phenotypes were related to the outcomes were removed. Third, for any palindromic SNPs identified, allele frequency data were used to confirm the corresponding alleles on the forward strand. Fourth, the strength of association (*F*) for all instrumental variables was calculated to avoid bias from weak instrumental variables (*F* < 10) affecting the results [15].

### Statistical analysis

**Fig. 1:**
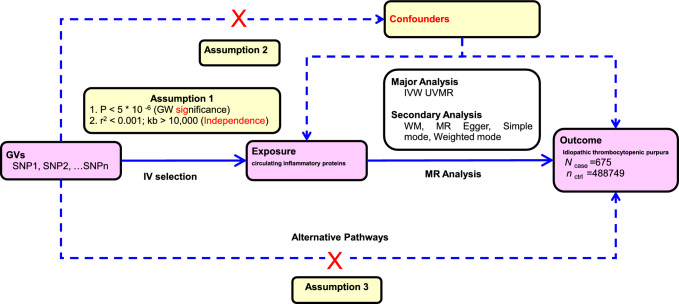
The flowchart of the study. The whole workflow of MR analysis. *GWAS* genome-wide association study; *SNP* single nucleotide polymorphism; *MR* Mendelian randomization

We employed the inverse-variance weighted (IVW) method as the gold standard for our analysis. In addition, to further validate the results, we used several other methods, including the weighted median (WM) method, the simple mode method, the weighted mode method, and MR‒Egger regression. The results were visually analyzed and presented to aid in interpretation [16]. To assess heterogeneity among individual genetic variants, we performed Cochran’s *Q* test using the mr_heterogeneity software package for SNPs that met the full hypothesis [17]. If the *p*-value of the Cochran’s *Q* test was less than 0.05, the results indicated significant heterogeneity. In such cases, the final MR results were derived from the random effects model using the IVW method as the gold standard. If there was no significant heterogeneity, the IVW method with the fixed effects model was considered the gold standard. In addition, we employed the Mendelian random pleiotropy test (Egger-intercept method) and the MR-PRESSO test to examine whether horizontal pleiotropy violated the Mendelian randomization assumptions. As part of the sensitivity analysis, we conducted a leave-one-out sensitivity test to check if any of the final SNPs were outliers. The stability of the results was further assessed by examining the symmetry of the funnel plot. Outliers were identified using the MR-PRESSO method, and their impact on the results was evaluated. We used the twoSample [18] MR package and MR-PRESSO [19] in R (version 4.1.2) for analysis. The overall workflow of our study is illustrated in Fig. 1.

## Result

**Fig. 2:**
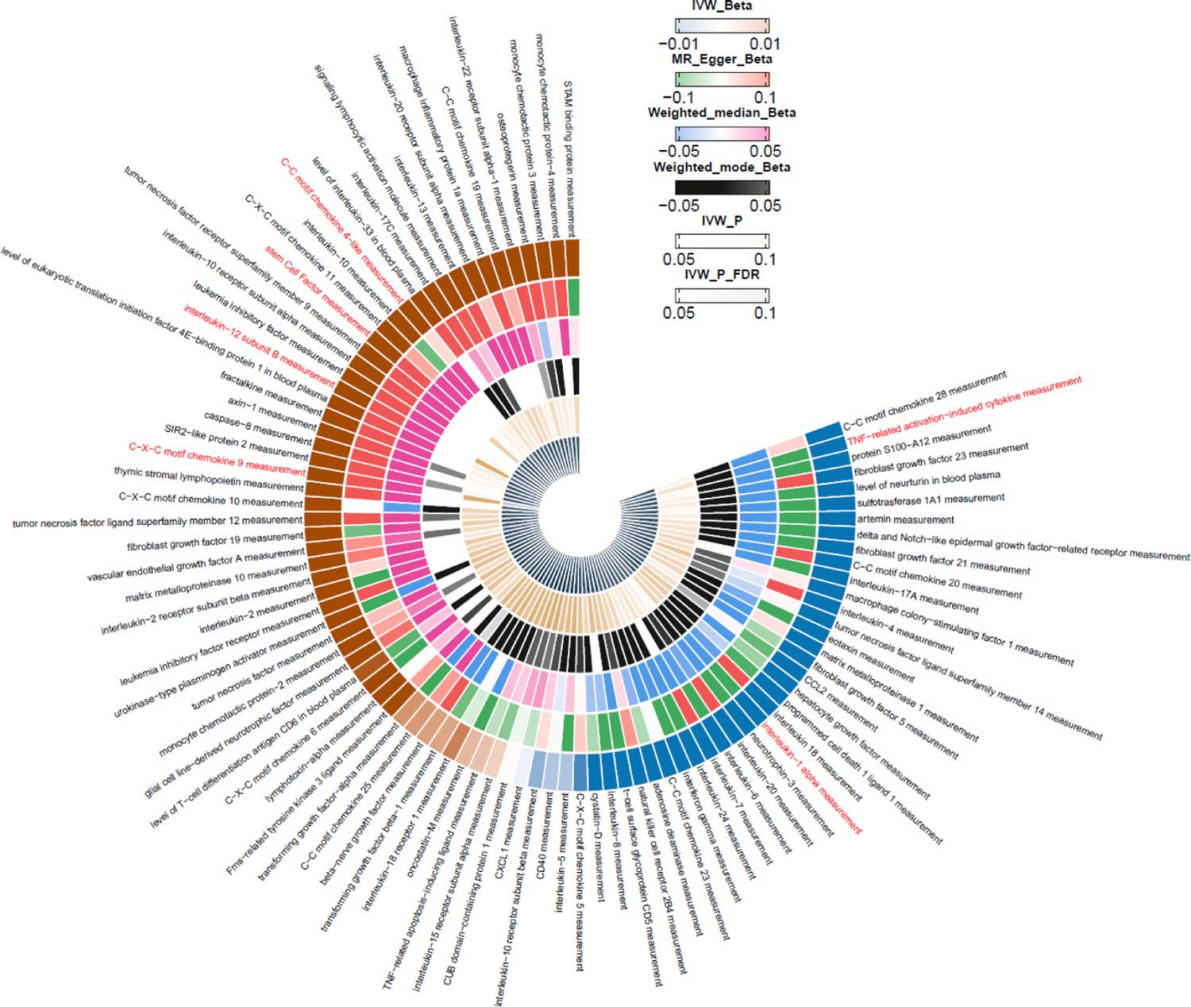
Circular heatmap of circulating inflammatory proteins and ITP association: significant proteins highlighted

The MR analysis identified six proteins that were significantly associated with ITP, including both risk factors and protective factors. A detailed list of the associations between the 91 circulating inflammatory proteins and ITP can be found in Supplementary Table 4. Among these 91 circulating inflammatory protein exposures, the IVW analytical method revealed significant associations for six proteins: C–C motif chemokine 4-like (CCL4), C–X–C motif chemokine 9 (CXCL9), interleukin-12 subunit B (IL-12B), stem cell factor (SCF), interleukin-1 alpha (IL-1α), and TNF-related activation-induced cytokine(TRANCE), as shown in Fig. 2. These results suggest a potential link to the onset of ITP.

### Selection of instrumental variables

Following an extensive quality control review, we identified 144 SNPs associated with six circulating inflammation-related proteins. Specifically, there were 19 SNPs linked to CCL4, 22 SNPs associated with CXCL9, 27 SNPs related to IL-12B, 14 SNPs associated with IL-1α, 33 SNPs linked to SCF, and 29 SNPs related to TRANCE. These SNPs were designated as IVs.

### Role of inflammation-related proteins in ITP

**Fig. 3:**
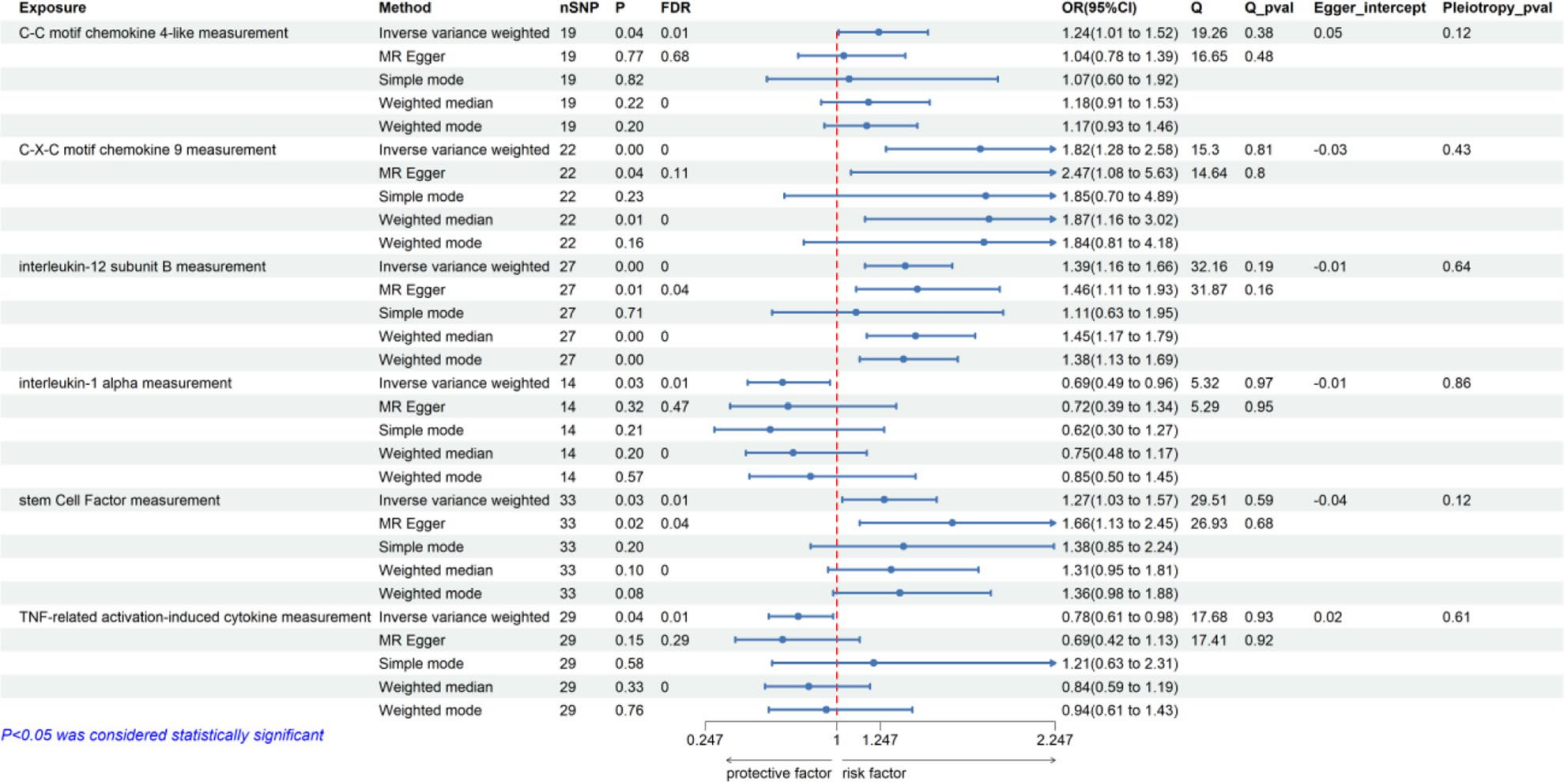
Relationship between circulating inflammatory proteins and ITP. *ITP* immune thrombocytopenia

Elevated genetically predicted levels of IL-1α were associated with a decreased risk of ITP, as shown in Fig. 3. Specifically, IL-1α levels (OR: 0.69; 95% confidence interval (CI): 0.49–0.96; *p* = 0.03; adjusted *p* = 0.01) demonstrated a suggestive inverse association with ITP. Similarly, TRANCE levels (OR: 0.78; 95% CI: 0.61–0.98; *p* = 0.04; adjusted *p* = 0.01) also indicated a suggestive inverse relationship with ITP, as depicted in Fig. 3. In contrast, higher levels of genetically predicted CCL4 (OR: 1.24; 95% CI: 1.01–1.52; *p* = 0.04; adjusted *p* = 0.01), CXCL9 (OR: 1.82; 95% CI: 1.28–2.58; *p* < 0.01; adjusted *p* < 0.01), IL-12B (OR: 1.39; 95% CI: 1.16–1.66; *p* < 0.01; adjusted *p* < 0.01), and SCF (OR: 1.27; 95% CI: 1.03–1.57; *p* = 0.03; adjusted *p* = 0.01) suggested increased associations with the risk of ITP.

### Sensitivity analyses

**Fig. 4:**
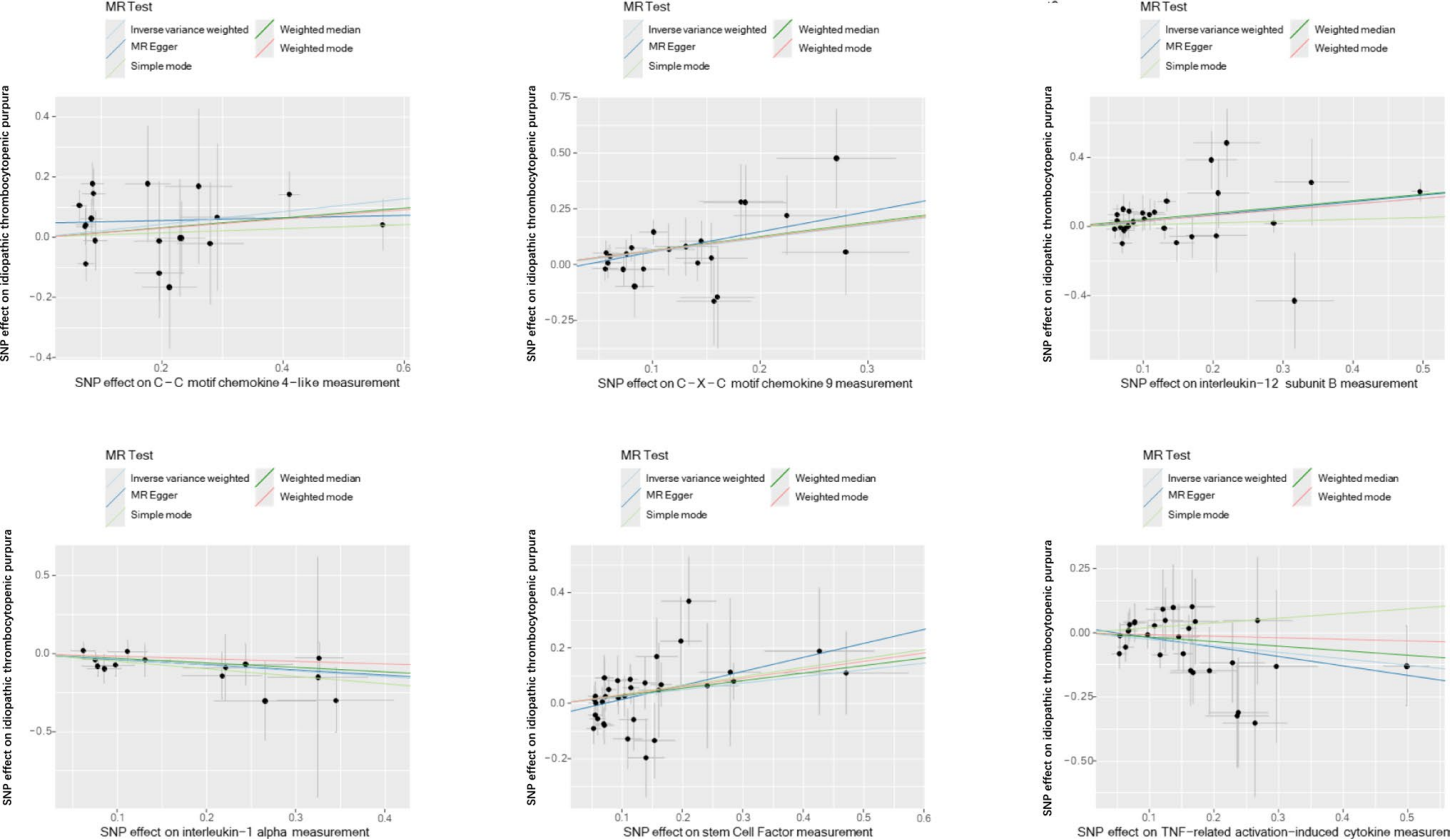
Scatter plots for the association between circulating inflammatory proteins and ITP. *ITP* immune thrombocytopenia

**Fig. 5:**
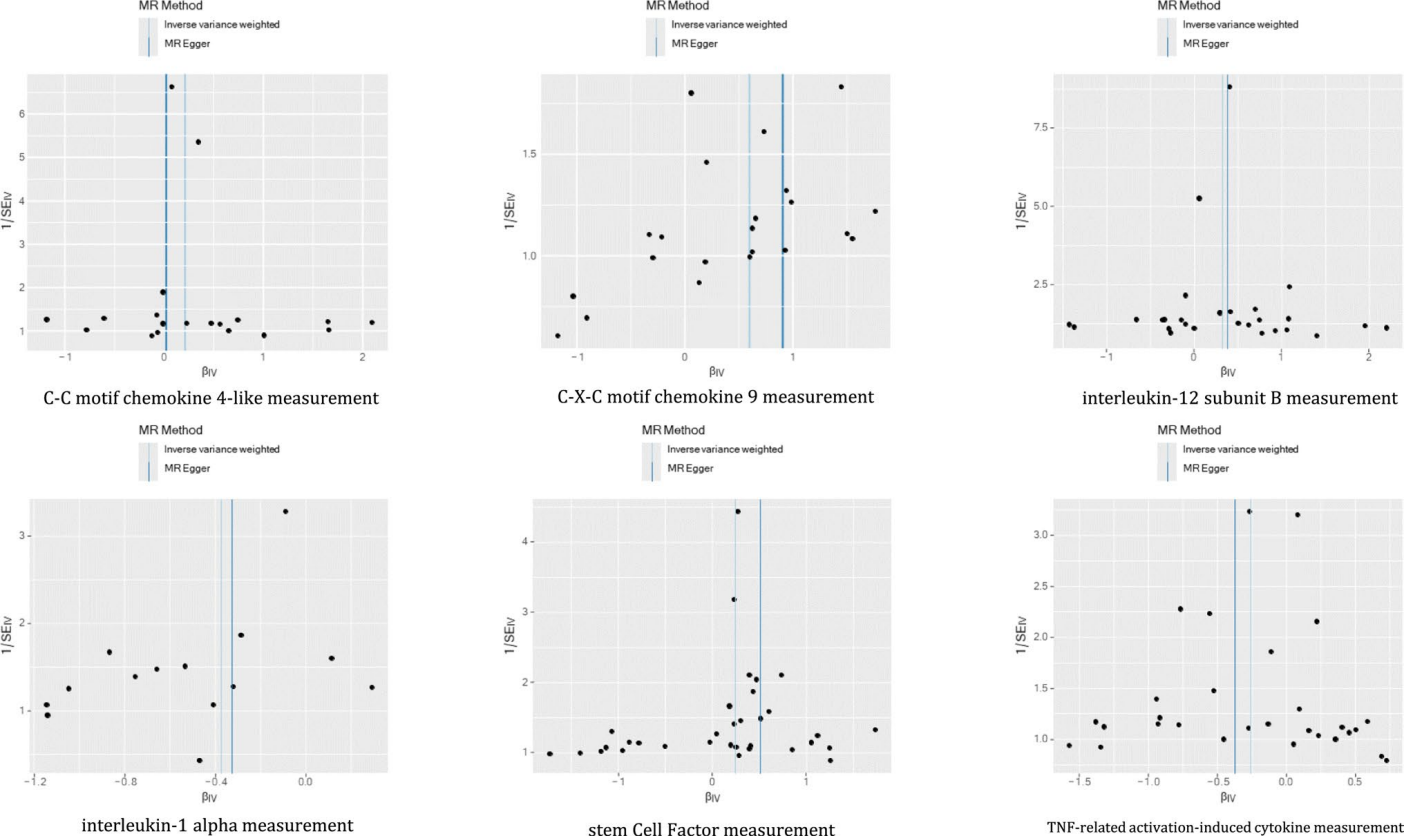
Funnel plots of circulating inflammatory proteins

**Fig. 6:**
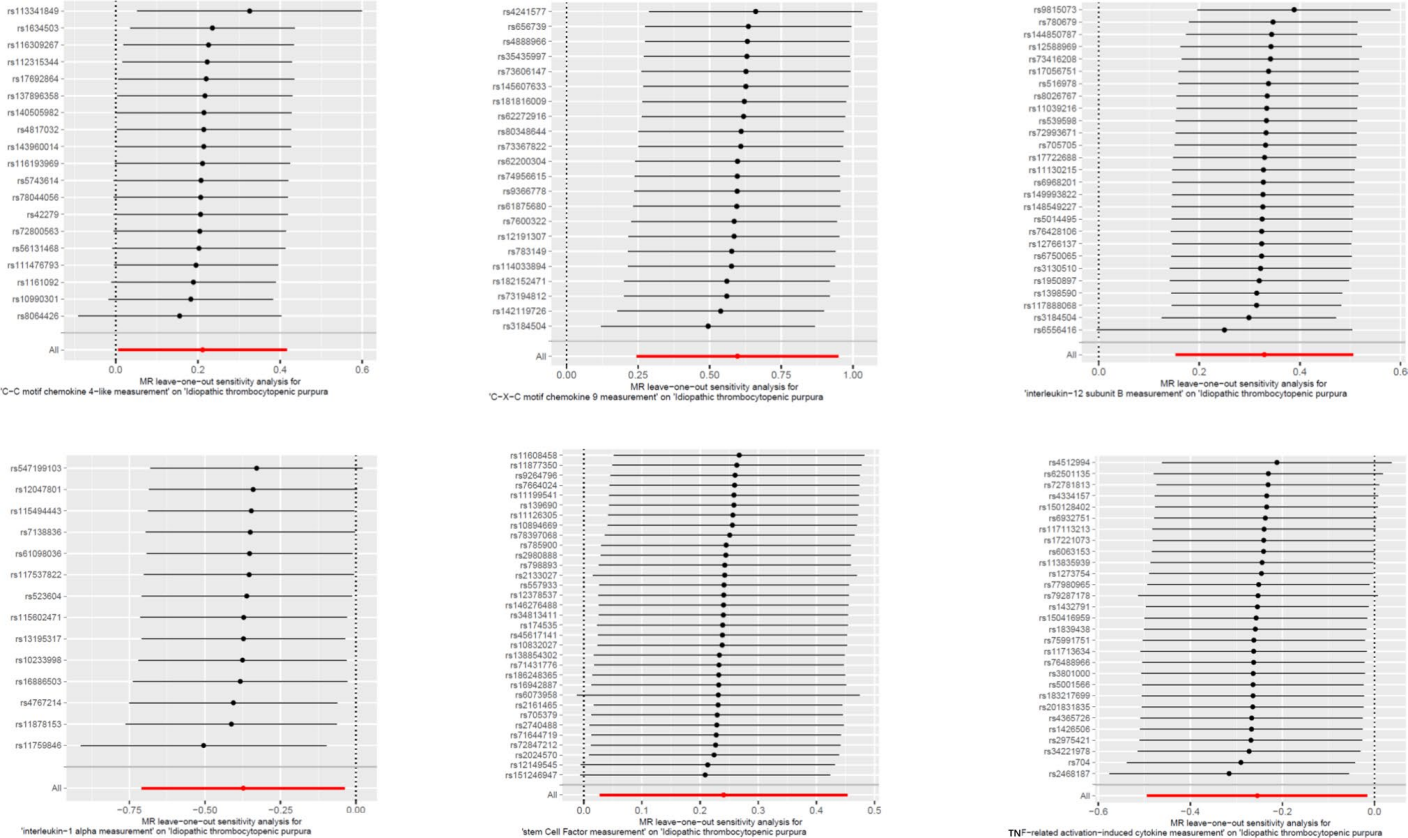
Leave-one-out plots for the association between circulating inflammatory proteins and ITP. *ITP* immune thrombocytopenia

The MR-Egger regression intercepts were not significantly different from zero, indicating the absence of horizontal pleiotropy (all intercept *p* > 0.05), as shown in Fig. 4. In addition, the MR-PRESSO test revealed no pleiotropic outliers among the SNPs (*p* > 0.05), further supporting the lack of pleiotropy. These findings were consistent with results from the weighted median approach. Scatter plots depicting the genetic associations between circulating inflammatory proteins and ITP are presented in Fig. 4. Cochran’s *Q* test showed no heterogeneity among the genetic instrumental variables for the measured levels (all *p* > 0.1). Furthermore, funnel plots demonstrated no significant asymmetry, suggesting minimal directional horizontal pleiotropy (Fig. 5). The robustness of these causal estimates was further confirmed through a leave-one-out analysis, which indicated that no single instrumental variable disproportionately affected the observed causal relationships, as illustrated in Fig. 6.

## Discussion

This study employed a two-sample approach to assess the relationships between specific circulating inflammation-related proteins and ITP. By utilizing summary statistics from GWAS meta-analyses for these proteins, in conjunction with ITP data from the IEU Open GWAS Project, our findings highlight the protective role of IL-1α and TRANCE against ITP. In addition, the study identifies elevated levels of CCL4, CXCL9, IL-12B, and SCF as potential risk factors for ITP.

IL-1α is a key cytokine that acts as an early signal, triggering and amplifying inflammatory responses [20]. It is primarily produced by activated macrophages, neutrophils, epithelial cells, and endothelial cells, and plays a role in various bodily responses, including hematopoietic, neurological, and endocrine functions, as well as in certain antitumor pathophysiological processes [21]. Moreover, it has metabolic, physiological, and hematopoietic activities, and plays a central role in the regulation of immune response. In ITP, disruptions in the signaling pathways involving IL-1α may contribute to its protective role in the disease. TRANCE, also known as receptor activator of nuclear factor-κB ligand (RANKL), is a member of the tumor necrosis factor superfamily, recognized for its significant role in bone development and various bone disorders, including osteoporosis, Paget’s disease, and metastatic bone cancer [22, 23]. TRANCE is also associated with promoting T cell development and growth, enhancing dendritic cell function, and inducing lymph node organogenesis [24]. Moreover, RANKL has been implicated in the modulation of immune responses, balancing inflammatory processes with immunosuppressive effects [25]. In autoimmune diseases, the role of RANKL becomes more intricate. While it can bolster the immune system’s defenses against pathogens, it may also contribute to autoimmunity under specific conditions, such as in Fas-deficient mice, where RANKL signaling is linked to rapidly progressing autoimmune symptoms [25]. This duality highlights the importance of the cellular source of RANKL and the context in which it operates, ultimately determining whether the outcome is immunostimulatory or immunosuppressive [26]. This complexity may account for the protective effect of TRANCE in ITP.

C–C motif chemokines are a subfamily of small, secreted proteins that interact with G protein-coupled chemokine receptors on cell surfaces, characterized by the presence of adjacent cysteine residues [27]. Their primary function is to regulate cell migration, particularly that of leukocytes, which is essential in both protective and harmful immune and inflammatory responses [28]. Elevated levels of CCL4 have been detected in patients with autoimmune diseases, suggesting its potential role in mediating immune responses linked to thrombocytopenia [29]. In addition, CCL4 can promote the recruitment of immune cells to inflamed sites, potentially exacerbating the pathophysiology of ITP [29].

The IL-12B gene encodes the IL-12B protein, an essential element of the immune response, located on human chromosome 5. It serves as a growth factor for activated T cells and natural killer (NK) cells, enhancing their activity and stimulating the production of interferon-gamma (IFN-γ). This gene encodes the shared subunit IL-12p40, which is part of the cytokines IL-12 and IL-23, playing a critical role in the pathogenesis of inflammatory bowel disease (IBD) by influencing the differentiation and activation of Th1 and Th17 cells [30]. In addition, IL-12B is linked to various pathogenic inflammatory responses, including silicosis, graft rejection, and asthma [31]. Dysregulation of this cytokine network may contribute to the autoimmune processes involved in ITP.

Monokine induced by interferon-gamma (MIG), commonly known as CXCL9, is classified within the CXC subfamily of chemokines. This chemokine is primarily produced in response to interferon-gamma (IFN-γ) and plays a significant role in recruiting immune cells, including cytotoxic T lymphocytes and natural killer cells, to sites of inflammation [32, 33]. Such recruitment may reactions intensify autoimmune, especially in the context of immune thrombocytopenia (ITP), where heightened levels of MIG have been observed, correlating with disease severity and reduced platelet counts.

SCF is an acidic glycoprotein secreted by stromal cells within the bone marrow microenvironment. It primarily influences early pluripotent stem cells and exhibits significant synergistic effects when combined with other cytokines. In immune thrombocytopenia (ITP), inadequate platelet production is a major contributor to thrombocytopenia. SCF plays a crucial role in promoting the proliferation and differentiation of hematopoietic stem cells and their progenitors, thereby enhancing megakaryocyte production, which is vital for platelet generation [34]. While SCF is mainly involved in hematopoietic regulation, it also exerts some influence on immune system functions. In the context of ITP, disruptions in the signaling pathways associated with SCF may further exacerbate the condition by impeding normal platelet formation.

To our knowledge, a Mendelian randomization study found a direct correlation between antibodies produced by *H. pylori* infection, specifically GroEL, and the incidence of ITP in a European population [35], suggesting a correlation between specific circulating proteins and ITP. Another Mendelian randomization study on the effect of gut flora on immune thrombocytopenia revealed a causal relationship between gut microbiota composition and ITP risk, highlighting three inflammatory cytokines as potential causal mediators of this relationship [36], which further supports the existence of a correlation between circulating proteins and ITP. These two studies suggest that using circulating inflammatory proteins as an entry point to explore the link between them and ITP could further expand the pathogenesis of ITP.

This study has several notable strengths. Primarily, it represents the inaugural application of Mendelian randomization (MR) analysis to investigate the associations between particular inflammation-related circulating proteins and immune thrombocytopenia (ITP). In addition, our research utilized a larger sample size and incorporated a greater number of genetic variants. Furthermore, the genetic variations associated with these proteins were derived from a recent meta-analysis of genome-wide association studies (GWAS), which bolsters the robustness of the instrumental variables employed in the MR analysis.

Our study acknowledges several limitations. First, the genome-wide association studies (GWAS) that provided summary statistics for this analysis were predominantly focused on individuals of European descent, which may limit the generalizability of our findings to other ethnic populations. Second, the relatively small number of cases included in our study raises the possibility of Type II errors; however, we meticulously selected robust instrumental variables (IVs) and conducted sensitivity analyses to mitigate these concerns, thereby reinforcing the reliability of our results. Lastly, the pathogenesis of immune thrombocytopenia (ITP) remains poorly understood, and there is a scarcity of research examining the relationship between the aforementioned proteins and ITP. Consequently, the precise pathophysiological mechanisms linking inflammatory proteins to ITP are not fully elucidated. Nonetheless, our innovative use of Mendelian randomization to investigate the association between relevant inflammatory proteins and ITP opens new avenues for further mechanistic studies by future researchers.

## Conclusion

This study confirms the potential relationship between circulating inflammatory proteins and immune thrombocytopenia (ITP), with four proteins (CCL4, CXCL9, IL-12B, and SCF) showing a positive association with ITP, while two proteins (IL-1α and TRANCE) exhibited a negative correlation. These findings not only provide important insights into the mechanisms of ITP but also broaden the idea for future therapeutic interventions and biomarker development.

## Supplementary Information

Below is the link to the electronic supplementary material.

Supplementary file1 (DOCX 344 KB)

## Data Availability

The authors are prepared to share that all data used are from publicly available datasets, with disease data sourced from https://gwas.mrcieu.ac.uk/datasets/ebi-a-GCST90018865/, and complete protein GWAS summary statistics can be found at https://www.phpc.cam.ac.uk/ceu/proteins and in the EBI GWAS catalog (GCST90274758–GCST90274848) for download. The above data have been included in the article/supplementary material, which can be found directly in the file “Supplementary material”.

## Abbreviations

ITP: Immune thrombocytopenia
IVW: Inverse-variance weighted
GWAS: Genome-Wide Association Studies
pQTLs: Protein quantitative trait loci
SNPs: Single-nucleotide polymorphisms
LD: Linkage disequilibrium
F: Strength of association
WM: Weighted median
MR-Egger: Mendelian randomization–Egger
MR-PRESSO: Mendelian randomization pleiotropy residual sum and outlier
OR: Odds ratio
CI: Confidence interval
MR: Mendelian randomization
CCL4: C–C motif chemokine ligand 4
CXCL9: C–X–C motif chemokine ligand 9
IL-1α: Interleukin-1 alpha
SCF: Stem cell factor
IL-12B: Interleukin-12 subunit beta
IFN: γ-Interferon gamma
NK: Natural killer
MIG: Monokine induced by gamma interferon
Th1: T helper cell type 1
Th17: T helper cell type 17

## References

[1] Lo E, Deane S. Diagnosis and classification of immune-mediated thrombocytopenia. Autoimmun Rev. 2014;13(4–5):577. 24444701 10.1016/j.autrev.2014.01.026

[2] Martinez-Carballeira D, Bernardo A, Caro A, et al. Pathophysiology, clinical manifestations and diagnosis of immune thrombocytopenia: contextualization from a historical perspective. Hematol Rep. 2024;16(2):204. 38651450 10.3390/hematolrep16020021 PMC11036214

[3] Terrell DR, Beebe LA, Vesely SK, et al. The incidence of immune thrombocytopenic purpura in children and adults: a critical review of published reports. Am J Hematol. 2010;85(3):174. 20131303 10.1002/ajh.21616

[4] Lin X, Xu A, Zhou L, et al. Imbalance of T lymphocyte subsets in adult immune thrombocytopenia. Int J Gen Med. 2021;14:937. 33776472 10.2147/IJGM.S298888 PMC7989055

[5] Fang J, Lin L, Lin D, et al. The imbalance between regulatory memory B cells reveals possible pathogenesis involvement in pediatric immune thrombocytopenia. Hematology. 2019;24(1):473. 31142214 10.1080/16078454.2019.1622292

[6] Kolls JK, Lindén A. Interleukin-17 family members and inflammation. Immunity. 2004;21(4):467. 15485625 10.1016/j.immuni.2004.08.018

[7] Davey Smith G, Hemani G. Mendelian randomization: genetic anchors for causal inference in epidemiological studies. Hum Mol Genet. 2014;23:R89. 25064373 10.1093/hmg/ddu328 PMC4170722

[8] Emdin CA, Khera AV, Kathiresan S. Mendelian Randomization. JAMA J Am Med Assoc. 2017;318(19):1925. 10.1001/jama.2017.17219 29164242

[9] Larsson SC, Butterworth AS, Burgess S. Mendelian randomization for cardiovascular diseases: principles and applications. Eur Heart J. 2023;44(47):4913. 37935836 10.1093/eurheartj/ehad736 PMC10719501

[10] Skrivankova VW, Richmond RC, Woolf BAR, et al. Strengthening the reporting of observational studies in epidemiology using Mendelian randomization: the STROBE-MR statement. JAMA J Am Med Assoc. 2021;326(16):1614. 10.1001/jama.2021.18236 34698778

[11] Zhao JH, Stacey D, Eriksson N, et al. Genetics of circulating inflammatory proteins identifies drivers of immune-mediated disease risk and therapeutic targets. Nat Immunol. 2023;24(9):1540. 37563310 10.1038/s41590-023-01588-w PMC10457199

[12] Li J, Niu QM, Wu AW, et al. Causal relationship between circulating immune cells and the risk of type 2 diabetes: a Mendelian randomization study. Front Endocrinol. 2023;14:1210415. 10.3389/fendo.2023.1210415 PMC10247959 37305035

[13] Wang CD, Zhu DD, Zhang DJ, et al. Causal role of immune cells in schizophrenia: Mendelian randomization (MR) study. BMC Psychiatry. 2023;23(1):590. 37582716 10.1186/s12888-023-05081-4 PMC10428653

[14] Cao J, Wang N, Luo Y, et al. A cause–effect relationship between Graves’ disease and the gut microbiome contributes to the thyroid-gut axis: a bidirectional two-sample Mendelian randomization study. Front Immunol. 2023;14:977587. 36865531 10.3389/fimmu.2023.977587 PMC9974146

[15] Dai JY, Chan KCG, Hsu L. Testing concordance of instrumental variable effects in generalized linear models with application to Mendelian randomization. Stat Med. 2014;33(23):3986. 24863158 10.1002/sim.6217 PMC4309290

[16] Manousaki D, Harroud A, Mitchell RE, et al. Vitamin D levels and risk of type 1 diabetes: a Mendelian randomization study. Plos Med. 2021;18(2): e1003536. 33630834 10.1371/journal.pmed.1003536 PMC7906317

[17] Hemani G, Tilling K, Smith GD. Orienting the causal relationship between imprecisely measured traits using GWAS summary data. PLoS Genet. 2017;13(11): e1007081. 29149188 10.1371/journal.pgen.1007081 PMC5711033

[18] Hemani G, Zhengn J, Elsworth B, et al. The MR-base platform supports systematic causal inference across the human phenome. Elife. 2018;7: e34408. 29846171 10.7554/eLife.34408 PMC5976434

[19] Verbanck M, Chen CY, Neale B, et al. Detection of widespread horizontal pleiotropy in causal relationships inferred from Mendelian randomization between complex traits and diseases. Nat Genet. 2018;50(5):693. 29686387 10.1038/s41588-018-0099-7 PMC6083837

[20] Chiu JW, Binte Hanafi Z, Chew LCY, et al. IL-1α processing, signaling and its role in cancer progression. Cells. 2021;10(1):92. 33430381 10.3390/cells10010092 PMC7827341

[21] Hickish T, Andre T, Wyrwicz L, et al. MABp1 as a novel antibody treatment for advanced colorectal cancer: a randomised, double-blind, placebo-controlled, phase 3 study. Lancet Oncol. 2017;18(2):192. 28094194 10.1016/S1470-2045(17)30006-2

[22] Puchner A, Simader E, Saferding V, et al. Bona fide dendritic cells are pivotal precursors for osteoclasts. Ann Rheum Dis. 2024;83(4):518. 38071515 10.1136/ard-2022-223817

[23] Banaganapalli B, Fallatah I, Alsubhi F, et al. Paget’s disease: a review of the epidemiology, etiology, genetics, and treatment. Front Genet. 2023;14:1131182. 37180975 10.3389/fgene.2023.1131182 PMC10169728

[24] Chang H, Marquez J, Chen BK, et al. Immune modulation with RANKL blockade through denosumab treatment in patients with cancer. Cancer Immunol Res. 2024;12(4):453. 38276989 10.1158/2326-6066.CIR-23-0184 PMC10993769

[25] Schifferli A, Cavalli F, Godeau B, et al. Understanding immune thrombocytopenia: looking out of the box. Front Med. 2021. 10.3389/fmed.2021.613192. 10.3389/fmed.2021.613192 PMC8266194 34249957

[26] Mussbacher M, Salzmann M, Brostjan C, et al. Cell type-specific roles of NF-κB linking inflammation and thrombosis. Front Immunol. 2019;10:85. 30778349 10.3389/fimmu.2019.00085 PMC6369217

[27] Hughes CE, Nibbs RJB. A guide to chemokines and their receptors. FEBS J. 2018;285(16):2944. 29637711 10.1111/febs.14466 PMC6120486

[28] Chang T-T, Chen J-W. Emerging role of chemokine CC motif ligand 4 related mechanisms in diabetes mellitus and cardiovascular disease: friends or foes? Cardiovasc Diabetol. 2016;15:117. 27553774 10.1186/s12933-016-0439-9 PMC4995753

[29] Yan M, Wang Z, Qiu Z, et al. Platelet signaling in immune landscape: comprehensive mechanism and clinical therapy. Biomarker Res. 2024;12(1):164. 10.1186/s40364-024-00700-y PMC11686937 39736771

[30] Cooper AM, Khader SA. IL-12p40: an inherently agonistic cytokine. Trends Immunol. 2007;28(1):33. 17126601 10.1016/j.it.2006.11.002

[31] Walter MJ, Kajiwara N, Karanja P, et al. Interleukin 12 p40 production by barrier epithelial cells during airway inflammation. J Exp Med. 2001;193(3):339. 11157054 10.1084/jem.193.3.339 PMC2195918

[32] TeruyaFeldstein J, Jaffe ES, Burd PR, et al. The role of mig, the monokine induced by interferon-gamma, and IP-10, the interferon-gamma-inducible protein-10, in tissue necrosis and vascular damage associated with Epstein-Barr virus-positive lymphoproliferative disease. Blood. 1997;90(10):4099. 9354680

[33] Zheng B, Keen KJ, Fritzler MJ, et al. Circulating cytokine levels in systemic sclerosis related interstitial lung disease and idiopathic pulmonary fibrosis. Sci Rep. 2023;13(1):6647. 37095095 10.1038/s41598-023-31232-4 PMC10125994

[34] Okumura N, Tsuji K, Ebihara Y, et al. Chemotactic and chemokinetic activities of stem cell factor on murine hematopoietic progenitor cells. Blood. 1996;87(10):4100. 8639767

[35] Chen Y, Mu Q, Ouyang G. Causal relationship between *Helicobacter pylori* antibodies and immune thrombocytopenia: a Mendelian randomization study. Mediterranean J Hematol Infect Dis. 2024;17(1): e2025003. 10.4084/MJHID.2025.003 PMC11740916 39830794

[36] Wang J-G, Dou H-H, Liang Q-Y. Impact of gut microbiota and inflammatory cytokines on immune thrombocytopenia. Eur J Haematol. 2025;114(1):120. 39380298 10.1111/ejh.14310

