# Supplementary material for: Exploration of the association between 91 inflammatory proteins and immune thrombocytopenia: a two-sample Mendelian randomization analysis
Source: Int J Hematol. 2025 Apr 22;122(3):372–80. doi: 10.1007/s12185-025-03987-1 (PMC12380979; doi:10.1007/s12185-025-03987-1)
Supplement: Supplementary file 1 — Supplementary file1 (DOCX 344 KB) [file 12185_2025_3987_MOESM1_ESM.docx]

[**Supplementary material**](https://www.dovepress.com/get_supplementary_file.php?f=456326.docx)

**Supplementary Table 1.** Circulating inflammatory protein number names

| ID | Reported trait | Symbol | Efo Traits | Discovery Sample Ancestry |
| --- | --- | --- | --- | --- |
| GCST90274758 | Eukaryotic translation initiation factor 4E-binding protein 1 levels | 4EBP1 | level of eukaryotic translation initiation factor 4E-binding protein 1 in blood plasma | 14736 European |
| GCST90274759 | Adenosine Deaminase levels | ADA | adenosine deaminase measurement | 14736 European |
| GCST90274760 | Artemin levels | ARTN | artemin measurement | 11778 European |
| GCST90274761 | Axin-1 levels | AXIN1 | axin-1 measurement | 11793 European |
| GCST90274762 | beta-nerve growth factor levels | Beta-NGF | beta-nerve growth factor measurement | 14743 European |
| GCST90274763 | Caspase 8 levels | CASP-8 | caspase-8 measurement | 14744 European |
| GCST90274764 | Eotaxin levels | CCL11 | eotaxin measurement | 14734 European |
| GCST90274765 | C-C motif chemokine 19 levels | CCL19 | C-C motif chemokine 19 measurement | 14736 European |
| GCST90274766 | C-C motif chemokine 20 levels | CCL20 | C-C motif chemokine 20 measurement | 14736 European |
| GCST90274767 | C-C motif chemokine 23 levels | CCL23 | C-C motif chemokine 23 measurement | 14736 European |
| GCST90274768 | C-C motif chemokine 25 levels | CCL25 | C-C motif chemokine 25 measurement | 14736 European |
| GCST90274769 | C-C motif chemokine 28 levels | CCL28 | C-C motif chemokine 28 measurement | 14734 European |
| GCST90274770 | C-C motif chemokine 4 levels | CCL4 | C-C motif chemokine 4-like measurement | 14744 European |
| GCST90274771 | Natural killer cell receptor 2B4 levels | CD244 | natural killer cell receptor 2B4 measurement | 14735 European |
| GCST90274772 | CD40L receptor levels | CD40 | CD40 measurement | 14736 European |
| GCST90274773 | T-cell surface glycoprotein CD5 levels | CD5 | t-cell surface glycoprotein CD5 measurement | 14735 European |
| GCST90274774 | T-cell surface glycoprotein CD6 isoform levels | CD6 | level of T-cell differentiation antigen CD6 in blood plasma | 14735 European |
| GCST90274775 | CUB domain-containing protein 1 levels | CDCP1 | CUB domain-containing protein 1 measurement | 14734 European |
| GCST90274776 | Macrophage colony-stimulating factor 1 levels | CSF-1 | macrophage colony-stimulating factor 1 measurement | 14734 European |
| GCST90274777 | Cystatin D levels | CST5 | cystatin-D measurement | 14736 European |
| GCST90274778 | Fractalkine levels | CX3CL1 | fractalkine measurement | 14743 European |
| GCST90274779 | C-X-C motif chemokine 1 levels | CXCL1 | CXCL1 measurement | 14736 European |
| GCST90274780 | C-X-C motif chemokine 10 levels | CXCL10 | C-X-C motif chemokine 10 measurement | 14744 European |
| GCST90274781 | C-X-C motif chemokine 11 levels | CXCL11 | C-X-C motif chemokine 11 measurement | 14736 European |
| GCST90274782 | C-X-C motif chemokine 5 levels | CXCL5 | C-X-C motif chemokine 5 measurement | 14736 European |
| GCST90274783 | C-X-C motif chemokine 6 levels | CXCL6 | C-X-C motif chemokine 6 measurement | 14744 European |
| GCST90274784 | C-X-C motif chemokine 9 levels | CXCL9 | C-X-C motif chemokine 9 measurement | 14735 European |
| GCST90274785 | Delta and Notch-like epidermal growth factor-related receptor levels | DNER | delta and Notch-like epidermal growth factor-related receptor measurement | 14735 European |
| GCST90274786 | Protein S100-A12 levels | EN-RAGE | protein S100-A12 measurement | 14743 European |
| GCST90274787 | Fibroblast growth factor 19 levels | FGF-19 | fibroblast growth factor 19 measurement | 14744 European |
| GCST90274788 | Fibroblast growth factor 21 levels | FGF-21 | fibroblast growth factor 21 measurement | 14743 European |
| GCST90274789 | Fibroblast growth factor 23 levels | FGF-23 | fibroblast growth factor 23 measurement | 14735 European |
| GCST90274790 | Fibroblast growth factor 5 levels | FGF-5 | fibroblast growth factor 5 measurement | 11789 European |
| GCST90274791 | Fms-related tyrosine kinase 3 ligand levels | FIt3L | obsolete_Fms-related tyrosine kinase 3 ligand measurement | 14734 European |
| GCST90274792 | Glial cell line-derived neurotrophic factor levels | hGDNF | glial cell line-derived neurotrophic factor measurement | 14736 European |
| GCST90274793 | Hepatocyte growth factor levels | HGF | hepatocyte growth factor measurement | 14734 European |
| GCST90274794 | Interferon gamma levels | IFN-gamma | interferon gamma measurement | 11793 European |
| GCST90274795 | Interleukin-10 levels | IL-10 | interleukin-10 measurement | 14744 European |
| GCST90274796 | Interleukin-10 receptor subunit alpha levels | IL-10RA | interleukin-10 receptor subunit alpha measurement | 11793 European |
| GCST90274797 | Interleukin-10 receptor subunit beta levels | IL-10RB | interleukin-10 receptor subunit beta measurement | 14734 European |
| GCST90274798 | Interleukin-12 subunit beta levels | IL-12B | obsolete_interleukin-12 subunit B measurement | 14735 European |
| GCST90274799 | Interleukin-13 levels | IL-13 | interleukin-13 measurement | 11792 European |
| GCST90274800 | Interleukin-15 receptor subunit alpha levels | IL-15RA | interleukin-15 receptor subunit alpha measurement | 11792 European |
| GCST90274801 | Interleukin-17A levels | IL-17A | interleukin-17A measurement | 11784 European |
| GCST90274802 | Interleukin-17C levels | IL-17C | interleukin-17C measurement | 11793 European |
| GCST90274803 | Interleukin-18 levels | IL-18 | interleukin 18 measurement | 14744 European |
| GCST90274804 | interleukin-18 receptor 1 levels | IL-18R1 | interleukin-18 receptor 1 measurement | 14743 European |
| GCST90274805 | Interleukin-1-alpha levels | IL-1 alpha | obsolete_interleukin-1 alpha measurement | 11788 European |
| GCST90274806 | Interleukin-2 levels | IL-2 | interleukin-2 measurement | 11789 European |
| GCST90274807 | Interleukin-20 levels | IL-20 | interleukin-20 measurement | 11784 European |
| GCST90274808 | Interleukin-20 receptor subunit alpha levels | IL-20RA | interleukin-20 receptor subunit alpha measurement | 11792 European |
| GCST90274809 | Interleukin-22 receptor subunit alpha-1 levels | IL-22RA1 | interleukin-22 receptor subunit alpha-1 measurement | 11793 European |
| GCST90274810 | Interleukin-24 levels | IL-24 | interleukin-24 measurement | 11785 European |
| GCST90274811 | Interleukin-2 receptor subunit beta levels | IL-2RB | interleukin-2 receptor subunit beta measurement | 11792 European |
| GCST90274812 | Interleukin-33 levels | IL-33 | level of interleukin-33 in blood plasma | 11793 European |
| GCST90274813 | Interleukin-4 levels | IL-4 | interleukin-4 measurement | 11793 European |
| GCST90274814 | Interleukin-5 levels | IL-5 | interleukin-5 measurement | 11792 European |
| GCST90274815 | Interleukin-6 levels | IL-6 | interleukin-6 measurement | 14743 European |
| GCST90274816 | Interleukin-7 levels | IL-7 | interleukin-7 measurement | 14736 European |
| GCST90274817 | Interleukin-8 levels | IL-8 | interleukin-8 measurement | 14744 European |
| GCST90274818 | Latency-associated peptide transforming growth factor beta 1 levels | LAP TGF-beta-1 | transforming growth factor beta-1 measurement | 14736 European |
| GCST90274819 | Leukemia inhibitory factor levels | LIF | leukemia inhibitory factor measurement | 11793 European |
| GCST90274820 | Leukemia inhibitory factor receptor levels | LIF-R | leukemia inhibitory factor receptor measurement | 11784 European |
| GCST90274821 | Monocyte chemoattractant protein-1 levels | CCL8 | CCL2 measurement | 14733 European |
| GCST90274822 | Monocyte chemoattractant protein 2 levels | CCL2 | monocyte chemotactic protein-2 measurement | 14736 European |
| GCST90274823 | Monocyte chemoattractant protein-3 levels | CCL7 | monocyte chemotactic protein 3 measurement | 11783 European |
| GCST90274824 | Monocyte chemoattractant protein-4 levels | CCL13 | monocyte chemotactic protein-4 measurement | 14736 European |
| GCST90274825 | Macrophage inflammatory protein 1a levels | MIP-1 alpha | macrophage inflammatory protein 1a measurement | 14743 European |
| GCST90274826 | Matrix metalloproteinase-1 levels | MMP-1 | matrix metalloproteinase 1 measurement | 14744 European |
| GCST90274827 | Matrix metalloproteinase-10 levels | MMP-10 | matrix metalloproteinase 10 measurement | 14744 European |
| GCST90274828 | Neurturin levels | NRTN | level of neurturin in blood plasma | 11791 European |
| GCST90274829 | Neurotrophin-3 levels | NT-3 | neurotrophin-3 measurement | 14744 European |
| GCST90274830 | Osteoprotegerin levels | OPG | osteoprotegerin measurement | 14733 European |
| GCST90274831 | Oncostatin-M levels | OSM | oncostatin-M measurement | 14736 European |
| GCST90274832 | Programmed cell death 1 ligand 1 levels | PD-L1 | programmed cell death 1 ligand 1 measurement | 14736 European |
| GCST90274833 | Stem cell factor levels | SCF | stem Cell Factor measurement | 14736 European |
| GCST90274834 | SIR2-like protein 2 levels | SIRT2 | SIR2-like protein 2 measurement | 14736 European |
| GCST90274835 | Signaling lymphocytic activation molecule levels | SLAMF1 | signaling lymphocytic activation molecule measurement | 14734 European |
| GCST90274836 | Sulfotransferase 1A1 levels | ST1A1 | sulfotrasferase 1A1 measurement | 11793 European |
| GCST90274837 | STAM binding protein levels | STAMPB | STAM binding protein measurement | 14736 European |
| GCST90274838 | Transforming growth factor-alpha levels | TGF-alpha | transforming growth factor-alpha measurement | 14733 European |
| GCST90274839 | Tumor necrosis factor levels | TWEAK | tumor necrosis factor measurement | 11785 European |
| GCST90274840 | TNF-beta levels | TNFB | lymphotoxin-alpha measurement | 11792 European |
| GCST90274841 | Tumor necrosis factor receptor superfamily member 9 levels | TNFRSF9 | tumor necrosis factor receptor superfamily member 9 measurement | 11784 European |
| GCST90274842 | Tumor necrosis factor ligand superfamily member 14 levels | TNFSF14 | tumor necrosis factor ligand superfamily member 14 measurement | 11793 European |
| GCST90274843 | TNF-related apoptosis-inducing ligand levels | TRAIL | TNF-related apoptosis-inducing ligand measurement | 14735 European |
| GCST90274844 | TNF-related activation-induced cytokine levels | TRANCE | TNF-related activation-induced cytokine measurement | 14736 European |
| GCST90274845 | Thymic stromal lymphopoietin levels | TSLP | thymic stromal lymphopoietin measurement | 11793 European |
| GCST90274846 | Tumor necrosis factor ligand superfamily member 12 levels | TNF | tumor necrosis factor ligand superfamily member 12 measurement | 14736 European |
| GCST90274847 | Urokinase-type plasminogen activator levels | uPA | urokinase-type plasminogen activator measurement | 14734 European |
| GCST90274848 | Vascular endothelial growth factor A levels | VEGF_A | vascular endothelial growth factor A measurement | 14744 European |

**Supplementary Table 2.** All SNPs information used as IVs.

| Symbol | chr | pos | effect_allele | other_allele | beta | se | eaf | pval | SNP | samplesize |
| --- | --- | --- | --- | --- | --- | --- | --- | --- | --- | --- |
| 4EBP1 | 11 | 68921211 | T | C | -0.0593 | 0.0124 | 0.3383 | 1.73E-06 | rs10792025 | 14727 |
| 4EBP1 | 1 | 2.41E+08 | A | G | -0.0884 | 0.0175 | 0.8334 | 4.39E-07 | rs10926313 | 14288 |
| 4EBP1 | 20 | 44566861 | C | G | 0.2235 | 0.0486 | 0.9827 | 4.25E-06 | rs137887001 | 14288 |
| 4EBP1 | 12 | 23923166 | T | C | -0.2226 | 0.0474 | 0.0227 | 2.65E-06 | rs181095999 | 14736 |
| 4EBP1 | 5 | 1.77E+08 | T | C | 0.0926 | 0.0152 | 0.2679 | 1.11E-09 | rs2731672 | 11783 |
| 4EBP1 | 17 | 15470414 | T | C | -0.0995 | 0.0214 | 0.8975 | 3.33E-06 | rs2856226 | 12404 |
| 4EBP1 | 1 | 80975190 | T | G | 0.0588 | 0.0123 | 0.6475 | 1.75E-06 | rs356283 | 14730 |
| 4EBP1 | 4 | 1.87E+08 | A | G | -0.1305 | 0.0116 | 0.5102 | 2.32E-29 | rs3733402 | 14736 |
| 4EBP1 | 3 | 1.86E+08 | T | C | 0.0594 | 0.0119 | 0.5711 | 5.99E-07 | rs5030072 | 14716 |
| 4EBP1 | 7 | 17594314 | T | C | 0.0754 | 0.0142 | 0.2938 | 1.10E-07 | rs6963617 | 14288 |
| 4EBP1 | 2 | 38198906 | T | G | 0.2607 | 0.0521 | 0.015 | 5.62E-07 | rs76686678 | 14730 |
| ADA | 4 | 5801734 | T | C | -0.0925 | 0.0199 | 0.905 | 3.35E-06 | rs10024178 | 14716 |
| ADA | 2 | 1.45E+08 | T | C | 0.2044 | 0.044 | 0.0243 | 3.39E-06 | rs111478357 | 13801 |
| ADA | 20 | 43175453 | C | G | -0.8597 | 0.025 | 0.9474 | 1.00E-200 | rs111555146 | 14733 |
| ADA | 1 | 57353848 | A | G | 0.139 | 0.0293 | 0.0462 | 2.10E-06 | rs115657851 | 14715 |
| ADA | 18 | 917376 | A | T | 0.3494 | 0.0754 | 0.0125 | 3.59E-06 | rs117645472 | 11095 |
| ADA | 20 | 43300667 | A | G | 0.12 | 0.0147 | 0.32 | 3.26E-16 | rs12479560 | 12404 |
| ADA | 7 | 22807634 | A | G | 0.0598 | 0.0122 | 0.656 | 9.50E-07 | rs1608554 | 14733 |
| ADA | 4 | 75665562 | A | T | 0.2754 | 0.0584 | 0.9857 | 2.41E-06 | rs189104742 | 14729 |
| ADA | 7 | 80678923 | T | C | 0.0562 | 0.0122 | 0.5989 | 4.09E-06 | rs2527856 | 14288 |
| ADA | 12 | 65118323 | C | G | -0.0551 | 0.0119 | 0.5267 | 3.65E-06 | rs2620728 | 14288 |
| ADA | 10 | 8502517 | A | T | 0.5548 | 0.1204 | 0.0104 | 4.07E-06 | rs4749761 | 6862 |
| ADA | 1 | 2.05E+08 | T | C | 0.1673 | 0.0358 | 0.0316 | 2.97E-06 | rs541944924 | 14080 |
| ADA | 4 | 1.25E+08 | T | C | 0.1281 | 0.0258 | 0.9456 | 6.87E-07 | rs62319741 | 14733 |
| ADA | 22 | 37921425 | A | G | -0.0599 | 0.0131 | 0.2529 | 4.82E-06 | rs7292320 | 14729 |
| ADA | 1 | 1.51E+08 | A | G | 0.3552 | 0.077 | 0.9905 | 3.97E-06 | rs75492374 | 11139 |
| ADA | 20 | 43223930 | T | C | 0.9687 | 0.0448 | 0.0189 | 1.10E-103 | rs80212763 | 14249 |
| ARTN | 12 | 81724190 | T | C | 0.1703 | 0.0362 | 0.0344 | 2.55E-06 | rs118115625 | 11778 |
| ARTN | 9 | 92208227 | A | G | -0.0674 | 0.0136 | 0.5323 | 7.20E-07 | rs1329733 | 11330 |
| ARTN | 1 | 921716 | A | C | -0.0664 | 0.0144 | 0.5539 | 4.01E-06 | rs13303278 | 10265 |
| ARTN | 18 | 54688939 | T | C | -0.07 | 0.0153 | 0.7417 | 4.76E-06 | rs1370215 | 11330 |
| ARTN | 4 | 4424044 | A | G | -0.0811 | 0.0176 | 0.84 | 4.07E-06 | rs138311590 | 11758 |
| ARTN | 9 | 1.4E+08 | T | C | 0.2119 | 0.0435 | 0.038 | 1.11E-06 | rs142585779 | 10266 |
| ARTN | 7 | 1.07E+08 | A | G | 0.311 | 0.0639 | 0.0191 | 1.13E-06 | rs142970662 | 9834 |
| ARTN | 12 | 1.3E+08 | A | G | 0.2112 | 0.0451 | 0.9758 | 2.83E-06 | rs148226005 | 11765 |
| ARTN | 5 | 1.03E+08 | A | G | -0.0873 | 0.0189 | 0.8494 | 3.86E-06 | rs17340161 | 11330 |
| ARTN | 1 | 17628383 | T | G | 0.0632 | 0.0137 | 0.6627 | 3.97E-06 | rs3003450 | 11762 |
| ARTN | 8 | 3686552 | A | T | -0.079 | 0.0162 | 0.6759 | 1.08E-06 | rs4993886 | 8371 |
| ARTN | 8 | 19493177 | A | G | -0.2047 | 0.044 | 0.0245 | 3.28E-06 | rs55885546 | 11778 |
| ARTN | 11 | 1.13E+08 | T | G | -0.2687 | 0.0588 | 0.028 | 4.88E-06 | rs569463048 | 7943 |
| ARTN | 3 | 1.12E+08 | A | G | 0.0833 | 0.0166 | 0.1972 | 5.22E-07 | rs62277657 | 11777 |
| ARTN | 19 | 55422034 | C | G | -0.0881 | 0.0176 | 0.8122 | 5.57E-07 | rs6509909 | 11330 |
| ARTN | 1 | 2.25E+08 | A | C | -0.3226 | 0.0682 | 0.9882 | 2.24E-06 | rs76672219 | 11291 |
| ARTN | 11 | 1.23E+08 | C | G | 0.0679 | 0.0141 | 0.318 | 1.47E-06 | rs77502336 | 11775 |
| ARTN | 6 | 1.43E+08 | T | C | 0.0853 | 0.0181 | 0.1545 | 2.44E-06 | rs9496445 | 11768 |
| ARTN | 4 | 1.57E+08 | C | G | 0.0692 | 0.014 | 0.626 | 7.70E-07 | rs9684768 | 11330 |
| ARTN | 22 | 25646493 | T | C | 0.0765 | 0.0167 | 0.7151 | 4.63E-06 | rs9941952 | 11774 |
| AXIN1 | 5 | 78787853 | A | G | 0.1447 | 0.0305 | 0.9489 | 2.09E-06 | rs115455827 | 11792 |
| AXIN1 | 6 | 1.51E+08 | A | C | 0.2444 | 0.0522 | 0.0197 | 2.84E-06 | rs117097664 | 11345 |
| AXIN1 | 18 | 50513818 | A | G | -0.212 | 0.042 | 0.0295 | 4.47E-07 | rs148317708 | 10919 |
| AXIN1 | 8 | 1.02E+08 | A | G | -0.1229 | 0.0242 | 0.9127 | 3.80E-07 | rs2470622 | 11345 |
| AXIN1 | 7 | 77960243 | T | C | -0.067 | 0.0133 | 0.5969 | 4.71E-07 | rs2691530 | 11793 |
| AXIN1 | 3 | 1.28E+08 | A | G | 0.0626 | 0.0134 | 0.4935 | 2.99E-06 | rs2712427 | 11345 |
| AXIN1 | 18 | 77253338 | T | C | 0.0692 | 0.0148 | 0.6047 | 2.93E-06 | rs428324 | 10281 |
| AXIN1 | 15 | 81221771 | T | C | 0.1554 | 0.0329 | 0.9514 | 2.32E-06 | rs7171268 | 11345 |
| Beta-NGF | 19 | 43780396 | T | C | -0.271 | 0.0565 | 0.014 | 1.61E-06 | rs117164089 | 13421 |
| Beta-NGF | 5 | 1.33E+08 | T | G | 0.1213 | 0.0247 | 0.9435 | 9.06E-07 | rs12188775 | 14737 |
| Beta-NGF | 10 | 89839430 | A | G | -0.075 | 0.0148 | 0.1928 | 4.03E-07 | rs12259196 | 13244 |
| Beta-NGF | 7 | 30944899 | A | G | 0.1389 | 0.0276 | 0.0802 | 4.84E-07 | rs12533747 | 14295 |
| Beta-NGF | 7 | 1.51E+08 | C | G | 0.1867 | 0.0408 | 0.966 | 4.74E-06 | rs12539643 | 11344 |
| Beta-NGF | 22 | 49775973 | C | G | -0.2164 | 0.0467 | 0.0256 | 3.59E-06 | rs13058191 | 14295 |
| Beta-NGF | 16 | 3327943 | A | G | 0.2773 | 0.0602 | 0.0196 | 4.10E-06 | rs140072192 | 11925 |
| Beta-NGF | 21 | 43942626 | T | C | 0.2541 | 0.0556 | 0.0235 | 4.87E-06 | rs148834134 | 13421 |
| Beta-NGF | 7 | 1.3E+08 | A | C | -0.1617 | 0.0352 | 0.9727 | 4.35E-06 | rs17647697 | 14741 |
| Beta-NGF | 4 | 1.67E+08 | A | G | 0.2208 | 0.0462 | 0.0229 | 1.76E-06 | rs17689534 | 13421 |
| Beta-NGF | 14 | 83608081 | A | T | -0.2055 | 0.0432 | 0.9685 | 1.97E-06 | rs182658308 | 10894 |
| Beta-NGF | 8 | 1.23E+08 | A | G | 0.0626 | 0.0126 | 0.719 | 6.76E-07 | rs1878359 | 14740 |
| Beta-NGF | 9 | 90362040 | T | C | -0.0775 | 0.0118 | 0.4965 | 5.11E-11 | rs3128517 | 14295 |
| Beta-NGF | 19 | 16434937 | T | C | 0.0607 | 0.0124 | 0.3088 | 9.82E-07 | rs34559191 | 13675 |
| Beta-NGF | 6 | 32192617 | T | C | 0.1203 | 0.0262 | 0.9514 | 4.40E-06 | rs397081 | 14743 |
| Beta-NGF | 4 | 1.64E+08 | T | C | -0.1187 | 0.0258 | 0.9491 | 4.21E-06 | rs4017 | 14742 |
| Beta-NGF | 14 | 69285337 | C | G | -0.055 | 0.0117 | 0.5283 | 2.59E-06 | rs4899264 | 14295 |
| Beta-NGF | 18 | 55441626 | T | C | -0.1249 | 0.0259 | 0.1005 | 1.42E-06 | rs62092633 | 11344 |
| Beta-NGF | 8 | 13332042 | T | C | 0.0578 | 0.0117 | 0.6636 | 7.81E-07 | rs62494116 | 14729 |
| Beta-NGF | 1 | 1.16E+08 | A | C | -0.0797 | 0.0122 | 0.3498 | 6.46E-11 | rs6328 | 13224 |
| Beta-NGF | 4 | 96824143 | A | G | 0.0584 | 0.0126 | 0.3152 | 3.57E-06 | rs72673086 | 12858 |
| Beta-NGF | 6 | 838092 | T | C | 0.0853 | 0.0185 | 0.8904 | 4.01E-06 | rs77493302 | 14743 |
| Beta-NGF | 6 | 1.53E+08 | T | G | 0.0629 | 0.0137 | 0.2135 | 4.41E-06 | rs9479501 | 14729 |
| CASP-8 | 12 | 62645486 | C | G | -0.0858 | 0.0165 | 0.1545 | 1.99E-07 | rs1032417 | 14296 |
| CASP-8 | 6 | 1.58E+08 | A | G | 0.0681 | 0.0145 | 0.6981 | 2.65E-06 | rs1034059 | 14296 |
| CASP-8 | 8 | 5557775 | C | G | 0.1095 | 0.0238 | 0.9304 | 4.21E-06 | rs111764025 | 14731 |
| CASP-8 | 2 | 2.31E+08 | A | T | -0.0806 | 0.0162 | 0.1582 | 6.51E-07 | rs11695193 | 14743 |
| CASP-8 | 3 | 1.52E+08 | T | C | 0.0781 | 0.0168 | 0.856 | 3.34E-06 | rs11709039 | 14743 |
| CASP-8 | 1 | 1.57E+08 | A | C | -0.0812 | 0.0167 | 0.8466 | 1.16E-06 | rs12073499 | 14295 |
| CASP-8 | 1 | 1.57E+08 | A | C | -0.0812 | 0.0167 | 0.8466 | 1.16E-06 | rs12073499 | 14295 |
| CASP-8 | 20 | 44566861 | C | G | 0.2318 | 0.0486 | 0.9827 | 1.85E-06 | rs137887001 | 14296 |
| CASP-8 | 11 | 63817395 | C | G | -0.3615 | 0.0776 | 0.0099 | 3.19E-06 | rs140844269 | 12330 |
| CASP-8 | 5 | 1.8E+08 | A | G | 0.3474 | 0.0683 | 0.0145 | 3.65E-07 | rs148608719 | 12745 |
| CASP-8 | 1 | 2338015 | T | C | -0.2016 | 0.0422 | 0.9714 | 1.78E-06 | rs34154371 | 13232 |
| CASP-8 | 2 | 2.02E+08 | C | G | -0.1987 | 0.0173 | 0.8696 | 1.56E-30 | rs56328050 | 14743 |
| CASP-8 | 10 | 84027526 | T | C | 0.2044 | 0.0412 | 0.978 | 7.01E-07 | rs73306603 | 14741 |
| CASP-8 | 9 | 22742536 | A | G | -0.2001 | 0.0428 | 0.9804 | 2.94E-06 | rs78449258 | 14744 |
| CCL11 | 16 | 24006879 | T | C | 0.2107 | 0.0451 | 0.9774 | 2.99E-06 | rs114744647 | 14730 |
| CCL11 | 1 | 1.59E+08 | A | G | 0.0749 | 0.0114 | 0.5547 | 5.03E-11 | rs12075 | 14731 |
| CCL11 | 20 | 49033630 | T | G | 0.2826 | 0.0606 | 0.0125 | 3.11E-06 | rs139476527 | 12933 |
| CCL11 | 4 | 1.05E+08 | A | C | -0.3374 | 0.0714 | 0.0128 | 2.30E-06 | rs139597204 | 12303 |
| CCL11 | 11 | 24773107 | A | G | -0.0609 | 0.0131 | 0.6155 | 3.34E-06 | rs1564995 | 14286 |
| CCL11 | 19 | 37955248 | C | G | 0.0817 | 0.016 | 0.8362 | 3.29E-07 | rs2112920 | 14286 |
| CCL11 | 3 | 46258902 | T | C | 0.0785 | 0.0123 | 0.3561 | 1.75E-10 | rs2201150 | 14286 |
| CCL11 | 3 | 42906116 | T | C | -0.3316 | 0.0225 | 0.9289 | 3.69E-49 | rs2228467 | 14733 |
| CCL11 | 11 | 95274487 | T | G | 0.1017 | 0.0208 | 0.9023 | 1.01E-06 | rs34536806 | 14286 |
| CCL11 | 12 | 1.06E+08 | A | G | -0.1138 | 0.0244 | 0.9282 | 3.10E-06 | rs35543857 | 14286 |
| CCL11 | 17 | 12254722 | A | G | 0.1063 | 0.0226 | 0.8771 | 2.56E-06 | rs554698477 | 10894 |
| CCL11 | 18 | 1508223 | C | G | 0.0555 | 0.0121 | 0.5296 | 4.50E-06 | rs55843637 | 14286 |
| CCL11 | 5 | 1.18E+08 | A | C | -0.0656 | 0.0137 | 0.2333 | 1.68E-06 | rs6894241 | 14715 |
| CCL11 | 22 | 45003109 | A | G | -0.0861 | 0.0187 | 0.1199 | 4.14E-06 | rs73171545 | 14286 |
| CCL11 | 8 | 11700967 | A | T | -0.0913 | 0.0189 | 0.1104 | 1.36E-06 | rs73209015 | 14728 |
| CCL11 | 7 | 75495667 | A | G | 0.2002 | 0.0205 | 0.09 | 1.58E-22 | rs757973 | 14713 |
| CCL11 | 10 | 1.28E+08 | T | G | 0.1929 | 0.0422 | 0.9754 | 4.85E-06 | rs79432525 | 14247 |
| CCL11 | 3 | 43179320 | T | C | 0.1502 | 0.0303 | 0.0433 | 7.16E-07 | rs79684468 | 14286 |
| CCL11 | 17 | 32619052 | T | C | -0.1215 | 0.0156 | 0.167 | 6.78E-15 | rs79722574 | 14720 |
| CCL11 | 16 | 80051931 | T | C | -0.0818 | 0.0133 | 0.7358 | 7.73E-10 | rs9940390 | 14286 |
| CCL11 | 21 | 39593226 | A | G | -0.1678 | 0.0345 | 0.0333 | 1.15E-06 | rs9976282 | 14722 |
| CCL19 | 7 | 3911280 | A | G | 0.0729 | 0.0154 | 0.8219 | 2.20E-06 | rs10242459 | 14729 |
| CCL19 | 2 | 66640363 | C | G | 0.1856 | 0.0384 | 0.9689 | 1.34E-06 | rs10496135 | 14288 |
| CCL19 | 3 | 58516854 | C | G | 0.4485 | 0.0922 | 0.992 | 1.15E-06 | rs113539352 | 11439 |
| CCL19 | 9 | 34710084 | A | C | -0.1464 | 0.017 | 0.8639 | 7.19E-18 | rs11574915 | 14733 |
| CCL19 | 2 | 2.05E+08 | C | G | -0.0803 | 0.0123 | 0.4754 | 6.65E-11 | rs13010492 | 13422 |
| CCL19 | 14 | 76855291 | A | G | -0.2939 | 0.0616 | 0.0119 | 1.83E-06 | rs138108512 | 13422 |
| CCL19 | 6 | 1.25E+08 | C | G | 0.0757 | 0.0157 | 0.1687 | 1.42E-06 | rs1415763 | 14731 |
| CCL19 | 17 | 14918425 | T | C | -0.2707 | 0.0574 | 0.0151 | 2.40E-06 | rs147708384 | 13801 |
| CCL19 | 9 | 35612978 | A | G | 0.1238 | 0.0265 | 0.0534 | 2.99E-06 | rs34791102 | 14735 |
| CCL19 | 5 | 1.5E+08 | A | C | 0.0551 | 0.012 | 0.4736 | 4.40E-06 | rs3792790 | 14288 |
| CCL19 | 4 | 1.45E+08 | T | C | 0.0596 | 0.0122 | 0.3904 | 1.03E-06 | rs4554017 | 14288 |
| CCL19 | 12 | 1.12E+08 | A | T | -0.0801 | 0.0132 | 0.5095 | 1.29E-09 | rs4766578 | 11785 |
| CCL19 | 13 | 1.14E+08 | A | G | -0.0671 | 0.0137 | 0.2653 | 9.69E-07 | rs4907572 | 14722 |
| CCL19 | 3 | 1.32E+08 | T | G | 0.2346 | 0.0185 | 0.8822 | 7.53E-37 | rs62292952 | 14728 |
| CCL19 | 12 | 19813981 | A | T | -0.0697 | 0.0135 | 0.2452 | 2.43E-07 | rs6486995 | 14735 |
| CCL19 | 5 | 1.74E+08 | T | C | 0.0863 | 0.0169 | 0.8559 | 3.28E-07 | rs6870560 | 14732 |
| CCL19 | 8 | 61418130 | T | C | -0.3645 | 0.0756 | 0.989 | 1.43E-06 | rs73257451 | 11337 |
| CCL19 | 16 | 57300587 | A | T | 0.1349 | 0.0294 | 0.9557 | 4.47E-06 | rs74764632 | 14734 |
| CCL19 | 2 | 7425475 | C | G | 0.0615 | 0.013 | 0.6941 | 2.24E-06 | rs7595241 | 14288 |
| CCL19 | 4 | 1.24E+08 | T | C | -0.2096 | 0.045 | 0.0195 | 3.20E-06 | rs79086127 | 14247 |
| CCL19 | 6 | 32444093 | C | G | -0.4884 | 0.0277 | 0.9365 | 1.41E-69 | rs9469127 | 14735 |
| CCL20 | 2 | 2.29E+08 | T | C | -0.0996 | 0.0128 | 0.326 | 7.18E-15 | rs10207134 | 14287 |
| CCL20 | 11 | 42142787 | T | C | 0.1191 | 0.026 | 0.9413 | 4.63E-06 | rs10837780 | 14288 |
| CCL20 | 1 | 2.39E+08 | T | C | -0.0953 | 0.0208 | 0.0967 | 4.61E-06 | rs111674022 | 14288 |
| CCL20 | 6 | 1448190 | T | C | 0.0637 | 0.0135 | 0.3674 | 2.38E-06 | rs11242693 | 12404 |
| CCL20 | 8 | 22584718 | T | C | 0.0573 | 0.012 | 0.5396 | 1.80E-06 | rs1129474 | 14288 |
| CCL20 | 1 | 32260562 | T | C | 0.2585 | 0.0548 | 0.9859 | 2.39E-06 | rs11582772 | 14243 |
| CCL20 | 17 | 67136325 | C | G | -0.185 | 0.0359 | 0.9688 | 2.56E-07 | rs117753190 | 14243 |
| CCL20 | 17 | 72761195 | C | G | -0.2256 | 0.0429 | 0.9694 | 1.45E-07 | rs117900576 | 13224 |
| CCL20 | 21 | 44005002 | A | G | -0.1315 | 0.0276 | 0.9476 | 1.89E-06 | rs13049624 | 14288 |
| CCL20 | 5 | 1.33E+08 | T | C | 0.1091 | 0.0239 | 0.9296 | 5.00E-06 | rs13185268 | 14724 |
| CCL20 | 14 | 94625543 | T | C | -0.1512 | 0.0328 | 0.0376 | 4.03E-06 | rs151092565 | 14728 |
| CCL20 | 14 | 1.07E+08 | A | T | -0.1602 | 0.0344 | 0.0632 | 3.21E-06 | rs2003431 | 9786 |
| CCL20 | 9 | 1.16E+08 | T | C | 0.0636 | 0.0127 | 0.3159 | 5.50E-07 | rs2796032 | 14718 |
| CCL20 | 21 | 32961256 | T | C | 0.0804 | 0.0166 | 0.1452 | 1.28E-06 | rs35833676 | 14732 |
| CCL20 | 10 | 1.28E+08 | A | G | 0.1939 | 0.0414 | 0.9774 | 2.82E-06 | rs41302923 | 14726 |
| CCL20 | 12 | 1.04E+08 | A | G | 0.0635 | 0.0128 | 0.6933 | 7.02E-07 | rs4981022 | 14721 |
| CCL20 | 22 | 49877754 | T | G | -0.1184 | 0.0257 | 0.9406 | 4.09E-06 | rs5769672 | 14732 |
| CCL20 | 18 | 56487606 | T | C | -0.0736 | 0.0158 | 0.1755 | 3.19E-06 | rs6420556 | 14288 |
| CCL20 | 16 | 63795822 | A | T | 0.0616 | 0.0134 | 0.3426 | 4.29E-06 | rs6498926 | 12844 |
| CCL20 | 12 | 19657540 | A | G | 0.2057 | 0.0422 | 0.9739 | 1.09E-06 | rs73072893 | 13230 |
| CCL20 | 6 | 40998167 | T | C | 0.1459 | 0.0185 | 0.883 | 3.11E-15 | rs742493 | 14735 |
| CCL20 | 6 | 32635998 | T | G | -0.076 | 0.0148 | 0.215 | 2.82E-07 | rs9274623 | 14288 |
| CCL23 | 2 | 2.42E+08 | T | C | 0.0983 | 0.0215 | 0.9113 | 4.83E-06 | rs1001495 | 13650 |
| CCL23 | 10 | 1.33E+08 | A | G | 0.0971 | 0.0186 | 0.1154 | 1.79E-07 | rs11017721 | 14288 |
| CCL23 | 11 | 1989274 | A | G | -0.0692 | 0.014 | 0.7293 | 7.70E-07 | rs112492 | 13224 |
| CCL23 | 22 | 51076281 | T | C | -0.1397 | 0.0285 | 0.0559 | 9.50E-07 | rs113959305 | 13224 |
| CCL23 | 3 | 14445911 | A | G | -0.21 | 0.0426 | 0.0278 | 8.24E-07 | rs116683909 | 14724 |
| CCL23 | 17 | 34195180 | A | C | -0.3246 | 0.0413 | 0.9561 | 3.85E-15 | rs117639761 | 14736 |
| CCL23 | 6 | 86144743 | C | G | 0.1164 | 0.0237 | 0.9291 | 9.04E-07 | rs12204524 | 14727 |
| CCL23 | 17 | 21386459 | A | G | -0.0643 | 0.0137 | 0.7287 | 2.69E-06 | rs12949647 | 14288 |
| CCL23 | 3 | 6127208 | T | C | 0.2157 | 0.0449 | 0.0255 | 1.56E-06 | rs143910182 | 14730 |
| CCL23 | 1 | 2.03E+08 | A | G | -0.3061 | 0.0626 | 0.0116 | 1.01E-06 | rs146074782 | 12935 |
| CCL23 | 17 | 34445397 | T | C | -0.1285 | 0.025 | 0.0956 | 2.75E-07 | rs16971993 | 14288 |
| CCL23 | 11 | 84372512 | A | T | 0.0927 | 0.0202 | 0.0922 | 4.45E-06 | rs17735284 | 14725 |
| CCL23 | 10 | 18295482 | A | G | 0.0574 | 0.0125 | 0.3093 | 4.39E-06 | rs1926742 | 14725 |
| CCL23 | 4 | 77373079 | T | C | 0.0574 | 0.0114 | 0.4869 | 4.78E-07 | rs2870238 | 14736 |
| CCL23 | 3 | 46462144 | T | C | 0.0585 | 0.0127 | 0.6736 | 4.10E-06 | rs4317138 | 14288 |
| CCL23 | 19 | 45416741 | T | C | 0.0669 | 0.0138 | 0.2405 | 1.25E-06 | rs438811 | 14720 |
| CCL23 | 7 | 3238978 | T | C | -0.0548 | 0.0118 | 0.5907 | 3.42E-06 | rs4719850 | 14726 |
| CCL23 | 17 | 34326215 | A | C | -0.5147 | 0.0158 | 0.1497 | 1.00E-200 | rs712048 | 14723 |
| CCL23 | 17 | 34140999 | T | G | -0.2256 | 0.0291 | 0.0643 | 9.00E-15 | rs7225405 | 11538 |
| CCL23 | 7 | 17545964 | A | G | -0.1497 | 0.0319 | 0.0393 | 2.70E-06 | rs73071153 | 14728 |
| CCL23 | 12 | 57405049 | A | G | 0.0948 | 0.0207 | 0.0846 | 4.66E-06 | rs73114426 | 14731 |
| CCL23 | 1 | 1.62E+08 | C | G | 0.0603 | 0.0132 | 0.608 | 4.92E-06 | rs74127049 | 13422 |
| CCL23 | 3 | 40054358 | A | G | 0.1273 | 0.0278 | 0.048 | 4.67E-06 | rs74491711 | 14735 |
| CCL23 | 11 | 1.15E+08 | C | G | 0.224 | 0.0416 | 0.0223 | 7.26E-08 | rs77083410 | 14734 |
| CCL23 | 10 | 34433796 | A | G | -0.2064 | 0.0437 | 0.9788 | 2.32E-06 | rs77354000 | 14735 |
| CCL23 | 17 | 34816052 | T | C | -0.2538 | 0.0465 | 0.0298 | 4.81E-08 | rs77527696 | 13829 |
| CCL25 | 19 | 8041383 | A | G | 0.1261 | 0.0178 | 0.1309 | 1.40E-12 | rs10401186 | 14288 |
| CCL25 | 19 | 8238293 | T | G | -0.0733 | 0.012 | 0.4919 | 1.01E-09 | rs10426282 | 14288 |
| CCL25 | 11 | 64434151 | T | C | -0.0865 | 0.0181 | 0.8838 | 1.76E-06 | rs10792444 | 14732 |
| CCL25 | 11 | 24600146 | A | G | 0.0546 | 0.0117 | 0.5707 | 3.06E-06 | rs11028036 | 14728 |
| CCL25 | 9 | 86838024 | A | T | -0.2681 | 0.0579 | 0.9693 | 3.65E-06 | rs11140450 | 10797 |
| CCL25 | 8 | 10694001 | T | C | 0.1437 | 0.0297 | 0.0428 | 1.31E-06 | rs111437511 | 14733 |
| CCL25 | 1 | 23748852 | C | G | 0.0566 | 0.0119 | 0.4781 | 1.97E-06 | rs11589432 | 14287 |
| CCL25 | 9 | 1.25E+08 | T | C | 0.3717 | 0.0777 | 0.9916 | 1.72E-06 | rs116534774 | 11139 |
| CCL25 | 8 | 42100809 | A | G | 0.0769 | 0.0142 | 0.7856 | 6.11E-08 | rs12114935 | 14732 |
| CCL25 | 18 | 2511738 | T | C | -0.0673 | 0.0134 | 0.2475 | 5.10E-07 | rs12606983 | 14715 |
| CCL25 | 1 | 1.61E+08 | A | C | -0.1934 | 0.0406 | 0.0225 | 1.90E-06 | rs143132666 | 14734 |
| CCL25 | 15 | 56638156 | A | G | 0.2127 | 0.0449 | 0.0215 | 2.17E-06 | rs147851492 | 14727 |
| CCL25 | 2 | 1.31E+08 | T | C | -0.2782 | 0.0609 | 0.0129 | 4.92E-06 | rs149395110 | 12880 |
| CCL25 | 8 | 23781453 | T | C | -0.0546 | 0.0116 | 0.5447 | 2.52E-06 | rs1705699 | 14714 |
| CCL25 | 19 | 49206145 | C | G | -0.3279 | 0.0114 | 0.4445 | 6.20E-182 | rs516316 | 14734 |
| CCL25 | 13 | 22284926 | T | C | -0.0624 | 0.0122 | 0.3844 | 3.14E-07 | rs521795 | 14288 |
| CCL25 | 19 | 8120498 | A | G | -0.7591 | 0.0145 | 0.8223 | 1.00E-200 | rs62124688 | 14288 |
| CCL25 | 19 | 8187968 | A | T | -0.1775 | 0.0156 | 0.1669 | 5.37E-30 | rs62126084 | 14730 |
| CCL25 | 9 | 1.36E+08 | T | C | -0.2482 | 0.0166 | 0.1875 | 1.52E-50 | rs635634 | 11785 |
| CCL25 | 11 | 75419643 | A | G | 0.0548 | 0.0119 | 0.3757 | 4.12E-06 | rs636479 | 14731 |
| CCL25 | 12 | 81177765 | A | G | -0.0637 | 0.0133 | 0.6502 | 1.67E-06 | rs7137942 | 12404 |
| CCL25 | 12 | 578100 | A | G | -0.1159 | 0.0116 | 0.4365 | 1.66E-23 | rs7296588 | 14725 |
| CCL25 | 3 | 1.44E+08 | A | G | 0.0941 | 0.0198 | 0.103 | 2.01E-06 | rs7611785 | 14736 |
| CCL25 | 7 | 1.55E+08 | T | C | -0.0755 | 0.0161 | 0.1668 | 2.74E-06 | rs7806274 | 13667 |
| CCL25 | 10 | 29446793 | A | G | 0.2192 | 0.0478 | 0.9825 | 4.52E-06 | rs79990760 | 14725 |
| CCL25 | 6 | 67085077 | A | G | -0.0777 | 0.0163 | 0.846 | 1.87E-06 | rs851850 | 14288 |
| CCL28 | 17 | 7459299 | T | G | -0.0855 | 0.017 | 0.7824 | 4.92E-07 | rs11078696 | 10894 |
| CCL28 | 2 | 2.07E+08 | T | C | -0.1056 | 0.0198 | 0.0895 | 9.64E-08 | rs112641086 | 14731 |
| CCL28 | 5 | 84924989 | A | C | 0.2298 | 0.0488 | 0.9815 | 2.49E-06 | rs116782490 | 12633 |
| CCL28 | 22 | 38406782 | A | C | 0.0684 | 0.0121 | 0.3659 | 1.58E-08 | rs139902 | 14285 |
| CCL28 | 7 | 1.16E+08 | T | C | 0.1812 | 0.0392 | 0.0244 | 3.79E-06 | rs143400203 | 14722 |
| CCL28 | 1 | 64836646 | T | C | -0.1263 | 0.0264 | 0.9486 | 1.72E-06 | rs1514964 | 14734 |
| CCL28 | 10 | 90264140 | T | C | -0.058 | 0.012 | 0.6211 | 1.34E-06 | rs2477954 | 14286 |
| CCL28 | 5 | 1.32E+08 | A | G | 0.0597 | 0.0128 | 0.5481 | 3.10E-06 | rs2631360 | 11780 |
| CCL28 | 4 | 1.03E+08 | C | G | -0.0597 | 0.0121 | 0.6079 | 8.06E-07 | rs2866626 | 14285 |
| CCL28 | 1 | 2.07E+08 | C | G | 0.0638 | 0.0111 | 0.5279 | 9.04E-09 | rs291084 | 14726 |
| CCL28 | 11 | 25452442 | A | G | -0.0959 | 0.0208 | 0.8571 | 4.02E-06 | rs371663005 | 10894 |
| CCL28 | 5 | 43405459 | T | C | 0.0702 | 0.0122 | 0.4126 | 8.71E-09 | rs4866742 | 12829 |
| CCL28 | 12 | 55558442 | A | G | 0.3427 | 0.0672 | 0.0122 | 3.40E-07 | rs546951873 | 11273 |
| CCL28 | 18 | 1508223 | C | G | 0.0549 | 0.0119 | 0.5287 | 3.96E-06 | rs55843637 | 14286 |
| CCL28 | 12 | 54692061 | A | G | -0.0688 | 0.0118 | 0.4015 | 5.53E-09 | rs6580980 | 14285 |
| CCL28 | 1 | 1.55E+08 | T | C | 0.0534 | 0.0116 | 0.5411 | 4.16E-06 | rs6671688 | 14286 |
| CCL28 | 18 | 74179338 | C | G | 0.2181 | 0.0466 | 0.9802 | 2.87E-06 | rs71363004 | 11982 |
| CCL28 | 11 | 1.14E+08 | A | G | -0.1935 | 0.0355 | 0.0347 | 5.02E-08 | rs73000929 | 14734 |
| CCL28 | 2 | 1.12E+08 | A | G | 0.2696 | 0.0542 | 0.9859 | 6.55E-07 | rs76291845 | 14244 |
| CCL28 | 4 | 1.57E+08 | T | C | 0.0531 | 0.0116 | 0.6323 | 4.70E-06 | rs7661151 | 14730 |
| CCL28 | 9 | 90419900 | A | C | -0.2853 | 0.0513 | 0.0176 | 2.68E-08 | rs76669782 | 12933 |
| CCL28 | 13 | 1.07E+08 | T | G | 0.129 | 0.0282 | 0.9561 | 4.77E-06 | rs77569365 | 14734 |
| CCL28 | 10 | 1.05E+08 | T | C | -0.1844 | 0.0395 | 0.0271 | 3.04E-06 | rs78552613 | 14734 |
| CCL28 | 5 | 1.03E+08 | A | G | -0.1684 | 0.0323 | 0.0376 | 1.85E-07 | rs78843608 | 14725 |
| CCL4 | 9 | 1.06E+08 | A | T | 0.085 | 0.0186 | 0.8839 | 4.88E-06 | rs10990301 | 14296 |
| CCL4 | 4 | 53951496 | A | G | 0.0869 | 0.019 | 0.1344 | 4.79E-06 | rs111476793 | 12800 |
| CCL4 | 5 | 33775793 | T | G | 0.2123 | 0.0455 | 0.0284 | 3.07E-06 | rs112315344 | 14255 |
| CCL4 | 3 | 46384204 | A | G | 0.5651 | 0.0184 | 0.1268 | 1.00E-200 | rs113341849 | 14296 |
| CCL4 | 12 | 67722012 | A | G | -0.0635 | 0.0125 | 0.6742 | 3.77E-07 | rs1161092 | 14296 |
| CCL4 | 2 | 2.37E+08 | A | T | 0.2915 | 0.0617 | 0.0134 | 2.31E-06 | rs116193969 | 14296 |
| CCL4 | 3 | 756117 | C | G | 0.1956 | 0.0405 | 0.0299 | 1.37E-06 | rs116309267 | 14723 |
| CCL4 | 19 | 18917908 | T | C | 0.2797 | 0.0551 | 0.0186 | 3.85E-07 | rs137896358 | 12745 |
| CCL4 | 3 | 46459749 | T | C | -0.2294 | 0.0415 | 0.0248 | 3.24E-08 | rs140505982 | 13865 |
| CCL4 | 12 | 52285090 | A | G | 0.1953 | 0.0416 | 0.0228 | 2.67E-06 | rs143960014 | 14257 |
| CCL4 | 17 | 34421139 | A | G | -0.0745 | 0.0138 | 0.2416 | 6.72E-08 | rs1634503 | 14295 |
| CCL4 | 17 | 34945214 | T | C | 0.2321 | 0.0283 | 0.9406 | 2.38E-16 | rs17692864 | 13662 |
| CCL4 | 5 | 11317698 | T | C | 0.0743 | 0.0162 | 0.1598 | 4.51E-06 | rs42279 | 14738 |
| CCL4 | 21 | 26966560 | A | T | -0.0899 | 0.0196 | 0.8283 | 4.50E-06 | rs4817032 | 10894 |
| CCL4 | 10 | 1.33E+08 | A | G | 0.0833 | 0.0178 | 0.1323 | 2.87E-06 | rs56131468 | 14296 |
| CCL4 | 4 | 38798935 | T | C | 0.0728 | 0.0143 | 0.2565 | 3.56E-07 | rs5743614 | 12859 |
| CCL4 | 16 | 79338459 | T | G | -0.1765 | 0.0374 | 0.9693 | 2.37E-06 | rs72800563 | 14741 |
| CCL4 | 4 | 1.57E+08 | A | G | -0.2607 | 0.0552 | 0.0138 | 2.33E-06 | rs78044056 | 13809 |
| CCL4 | 17 | 34819750 | A | G | -0.4104 | 0.0173 | 0.1459 | 2.11E-124 | rs8064426 | 14295 |
| CD244 | 1 | 25261696 | A | G | -0.0559 | 0.0122 | 0.4429 | 4.61E-06 | rs10903115 | 14286 |
| CD244 | 9 | 92856642 | T | C | -0.3705 | 0.0763 | 0.9873 | 1.20E-06 | rs10992054 | 12304 |
| CD244 | 11 | 94575608 | A | G | 0.0768 | 0.0161 | 0.8167 | 1.84E-06 | rs11020961 | 14287 |
| CD244 | 11 | 1.26E+08 | A | G | -0.0702 | 0.0147 | 0.1876 | 1.79E-06 | rs11220506 | 14729 |
| CD244 | 1 | 1.61E+08 | A | G | -0.0624 | 0.0118 | 0.4675 | 1.24E-07 | rs11265488 | 14287 |
| CD244 | 1 | 1.61E+08 | A | G | 0.2122 | 0.0119 | 0.4066 | 3.99E-71 | rs11265493 | 14287 |
| CD244 | 4 | 1.75E+08 | A | G | -0.1804 | 0.0337 | 0.0393 | 8.65E-08 | rs114758826 | 14728 |
| CD244 | 22 | 17214005 | A | G | 0.1618 | 0.0348 | 0.9666 | 3.33E-06 | rs139924614 | 14725 |
| CD244 | 2 | 79554556 | T | C | -0.0589 | 0.0123 | 0.4388 | 1.68E-06 | rs1434219 | 14287 |
| CD244 | 1 | 88677329 | A | G | 0.2053 | 0.0411 | 0.0249 | 5.88E-07 | rs147033439 | 14731 |
| CD244 | 16 | 48902293 | A | C | -0.2146 | 0.047 | 0.0182 | 4.97E-06 | rs149458216 | 14725 |
| CD244 | 4 | 27611759 | C | G | -0.0637 | 0.0139 | 0.2366 | 4.59E-06 | rs1574847 | 14287 |
| CD244 | 8 | 3921805 | A | G | 0.0627 | 0.0134 | 0.6347 | 2.88E-06 | rs2554697 | 11782 |
| CD244 | 12 | 1.12E+08 | T | C | 0.116 | 0.0128 | 0.4764 | 1.27E-19 | rs3184504 | 11784 |
| CD244 | 8 | 1.07E+08 | T | G | 0.063 | 0.0138 | 0.248 | 4.99E-06 | rs34826779 | 14287 |
| CD244 | 1 | 44253015 | T | C | 0.0744 | 0.0119 | 0.4677 | 4.05E-10 | rs3828139 | 14286 |
| CD244 | 2 | 62495209 | A | G | 0.0661 | 0.0117 | 0.4329 | 1.61E-08 | rs4672486 | 14720 |
| CD244 | 2 | 1.12E+08 | T | C | 0.0704 | 0.0126 | 0.6994 | 2.31E-08 | rs56272634 | 14729 |
| CD244 | 6 | 614064 | A | G | -0.1074 | 0.0215 | 0.0794 | 5.87E-07 | rs6928352 | 14731 |
| CD244 | 17 | 7088423 | T | C | -0.0776 | 0.0125 | 0.6405 | 5.37E-10 | rs72837690 | 14287 |
| CD244 | 5 | 96078542 | T | C | 0.2288 | 0.0497 | 0.0184 | 4.15E-06 | rs78142752 | 14734 |
| CD40 | 20 | 44464356 | T | C | 0.3553 | 0.0437 | 0.0208 | 4.28E-16 | rs117191103 | 14736 |
| CD40 | 22 | 45002809 | A | G | 0.1085 | 0.0205 | 0.8849 | 1.21E-07 | rs12152184 | 12402 |
| CD40 | 4 | 7304426 | T | G | 0.3143 | 0.0687 | 0.9875 | 4.76E-06 | rs12500563 | 10795 |
| CD40 | 21 | 18103398 | T | G | 0.1632 | 0.0351 | 0.9648 | 3.33E-06 | rs138739860 | 11979 |
| CD40 | 2 | 1.23E+08 | A | G | 0.109 | 0.0216 | 0.914 | 4.50E-07 | rs138897138 | 14288 |
| CD40 | 3 | 3773438 | A | G | 0.0789 | 0.017 | 0.1353 | 3.46E-06 | rs3967170 | 14721 |
| CD40 | 16 | 84304113 | A | G | -0.0599 | 0.0123 | 0.6433 | 1.12E-06 | rs404842 | 14287 |
| CD40 | 1 | 2.11E+08 | C | G | 0.1978 | 0.0413 | 0.0203 | 1.67E-06 | rs4278412 | 14725 |
| CD40 | 2 | 1.61E+08 | T | C | 0.0541 | 0.0118 | 0.5003 | 4.55E-06 | rs4664334 | 14288 |
| CD40 | 6 | 1.42E+08 | T | C | -0.3275 | 0.0705 | 0.9887 | 3.39E-06 | rs551896428 | 12778 |
| CD40 | 20 | 44735263 | T | G | -0.4505 | 0.0126 | 0.256 | 1.00E-200 | rs6032663 | 14727 |
| CD40 | 19 | 57861085 | T | C | 0.0686 | 0.0116 | 0.3648 | 3.34E-09 | rs7250371 | 14722 |
| CD40 | 20 | 44606446 | A | T | -0.1788 | 0.0268 | 0.9406 | 2.53E-11 | rs73128569 | 14288 |
| CD40 | 9 | 29426599 | C | G | 0.1458 | 0.0316 | 0.9638 | 3.95E-06 | rs75626299 | 14728 |
| CD40 | 13 | 45595067 | A | C | 0.064 | 0.012 | 0.4882 | 9.64E-08 | rs9526031 | 14288 |
| CD40 | 4 | 55046724 | A | G | -0.0582 | 0.0125 | 0.4883 | 3.22E-06 | rs9993874 | 12403 |
| CD5 | 14 | 93329692 | T | C | -0.0588 | 0.0128 | 0.3206 | 4.35E-06 | rs11622090 | 13236 |
| CD5 | 10 | 6251691 | A | G | -0.1261 | 0.0256 | 0.0751 | 8.40E-07 | rs117276991 | 14287 |
| CD5 | 13 | 39003323 | A | G | 0.2374 | 0.0476 | 0.9826 | 6.12E-07 | rs117516652 | 14248 |
| CD5 | 10 | 71211945 | A | G | -0.0629 | 0.0132 | 0.7492 | 1.89E-06 | rs12416520 | 14709 |
| CD5 | 12 | 31688535 | A | G | 0.0569 | 0.0119 | 0.4361 | 1.74E-06 | rs1259355 | 14287 |
| CD5 | 2 | 2.05E+08 | C | G | -0.0589 | 0.0122 | 0.4754 | 1.38E-06 | rs13010492 | 13421 |
| CD5 | 4 | 77211819 | A | C | -0.0803 | 0.0151 | 0.2321 | 1.05E-07 | rs138861398 | 11967 |
| CD5 | 7 | 97499410 | A | C | -0.1134 | 0.0245 | 0.9301 | 3.68E-06 | rs17345759 | 14287 |
| CD5 | 9 | 27496604 | T | C | 0.1638 | 0.0334 | 0.9589 | 9.38E-07 | rs17769062 | 14287 |
| CD5 | 9 | 1.06E+08 | A | T | -0.4169 | 0.0806 | 0.9916 | 2.31E-07 | rs192582121 | 11438 |
| CD5 | 7 | 50716017 | T | C | 0.0623 | 0.0124 | 0.6389 | 5.06E-07 | rs2237450 | 14287 |
| CD5 | 6 | 32600375 | T | C | 0.0973 | 0.0165 | 0.1469 | 3.70E-09 | rs3104373 | 14735 |
| CD5 | 12 | 1.12E+08 | T | C | 0.1427 | 0.0128 | 0.4764 | 7.29E-29 | rs3184504 | 11784 |
| CD5 | 5 | 1.77E+08 | A | G | -0.0683 | 0.0131 | 0.7003 | 1.85E-07 | rs4073745 | 14287 |
| CD5 | 19 | 48833608 | A | G | 0.1471 | 0.0313 | 0.9627 | 2.61E-06 | rs4893 | 14735 |
| CD5 | 11 | 60793651 | C | G | 0.1083 | 0.012 | 0.6328 | 1.80E-19 | rs4939490 | 14735 |
| CD5 | 3 | 47239457 | A | G | 0.1833 | 0.0391 | 0.9694 | 2.76E-06 | rs563284470 | 11720 |
| CD5 | 10 | 1.14E+08 | A | T | -0.1681 | 0.0352 | 0.0327 | 1.79E-06 | rs60562286 | 14726 |
| CD5 | 1 | 1.8E+08 | T | C | 0.1841 | 0.0313 | 0.0399 | 4.06E-09 | rs61747728 | 14287 |
| CD5 | 1 | 1103150 | A | G | 0.0695 | 0.0147 | 0.2776 | 2.27E-06 | rs61768480 | 13670 |
| CD5 | 11 | 60922561 | C | G | 0.1193 | 0.0126 | 0.6395 | 2.85E-21 | rs674379 | 12835 |
| CD5 | 18 | 45545720 | A | G | -0.1199 | 0.0187 | 0.8853 | 1.44E-10 | rs7242462 | 14287 |
| CD5 | 6 | 4714797 | T | C | -0.1014 | 0.0217 | 0.0833 | 2.97E-06 | rs72821405 | 14727 |
| CD6 | 11 | 60109169 | A | G | 0.0613 | 0.0132 | 0.261 | 3.42E-06 | rs11230282 | 14734 |
| CD6 | 1 | 1.94E+08 | A | G | -0.1855 | 0.0392 | 0.0301 | 2.22E-06 | rs12074680 | 12404 |
| CD6 | 1 | 4327524 | T | C | 0.1766 | 0.0329 | 0.0397 | 7.97E-08 | rs143374617 | 14733 |
| CD6 | 11 | 60886913 | T | C | 0.0848 | 0.0155 | 0.1739 | 4.48E-08 | rs2241002 | 14732 |
| CD6 | 2 | 1.73E+08 | T | C | -0.2416 | 0.0517 | 0.017 | 2.97E-06 | rs35086654 | 13800 |
| CD6 | 22 | 37638221 | C | G | 0.1752 | 0.0376 | 0.9711 | 3.17E-06 | rs35634144 | 14237 |
| CD6 | 11 | 1.34E+08 | T | C | 0.2909 | 0.0584 | 0.0166 | 6.32E-07 | rs534268736 | 11760 |
| CD6 | 11 | 60731771 | A | G | 0.3 | 0.0115 | 0.5559 | 5.13E-150 | rs550421 | 14726 |
| CD6 | 12 | 1.12E+08 | A | G | 0.116 | 0.0134 | 0.4779 | 4.86E-18 | rs597808 | 11335 |
| CD6 | 9 | 33117954 | T | C | 0.0955 | 0.0193 | 0.1058 | 7.49E-07 | rs60988380 | 14735 |
| CD6 | 11 | 60819594 | A | G | -0.2482 | 0.0303 | 0.0439 | 2.58E-16 | rs7119502 | 14726 |
| CD6 | 2 | 2.28E+08 | A | T | -0.1796 | 0.0376 | 0.97 | 1.78E-06 | rs72969761 | 14710 |
| CD6 | 11 | 60770601 | T | C | 0.4025 | 0.0118 | 0.3408 | 1.00E-200 | rs7396422 | 14729 |
| CD6 | 4 | 83099601 | A | T | 0.0688 | 0.0129 | 0.6149 | 9.64E-08 | rs74886615 | 12404 |
| CD6 | 16 | 86486037 | A | G | -0.1079 | 0.0236 | 0.0691 | 4.83E-06 | rs8051133 | 14729 |
| CD6 | 1 | 95171460 | A | G | -0.0721 | 0.0154 | 0.8286 | 2.84E-06 | rs841332 | 14727 |
| CD6 | 7 | 1.54E+08 | A | C | 0.0646 | 0.0134 | 0.2505 | 1.43E-06 | rs9691968 | 14735 |
| CDCP1 | 5 | 2103391 | C | G | -0.0781 | 0.0156 | 0.8283 | 5.55E-07 | rs10044847 | 14719 |
| CDCP1 | 9 | 21413803 | C | G | -0.0616 | 0.0114 | 0.5862 | 6.53E-08 | rs10964983 | 14724 |
| CDCP1 | 4 | 53951496 | A | G | 0.0895 | 0.0182 | 0.1339 | 8.76E-07 | rs111476793 | 12790 |
| CDCP1 | 6 | 1.62E+08 | A | T | 0.0957 | 0.0197 | 0.0969 | 1.19E-06 | rs118116993 | 14286 |
| CDCP1 | 11 | 1.26E+08 | A | G | 0.1221 | 0.0122 | 0.3187 | 1.40E-23 | rs12290068 | 14726 |
| CDCP1 | 16 | 79183747 | A | T | -0.0658 | 0.0134 | 0.2312 | 9.09E-07 | rs12928188 | 14715 |
| CDCP1 | 20 | 18761853 | A | G | 0.4557 | 0.0968 | 0.0265 | 2.51E-06 | rs146583535 | 8372 |
| CDCP1 | 13 | 84915900 | A | G | 0.0609 | 0.0128 | 0.7179 | 1.96E-06 | rs17078000 | 14286 |
| CDCP1 | 22 | 31852311 | A | G | -0.1938 | 0.0409 | 0.9581 | 2.15E-06 | rs191929998 | 9844 |
| CDCP1 | 3 | 45187785 | C | G | 0.303 | 0.0146 | 0.8212 | 1.14E-95 | rs2276862 | 14730 |
| CDCP1 | 14 | 59010649 | T | C | -0.0541 | 0.0118 | 0.6294 | 4.55E-06 | rs28535751 | 14733 |
| CDCP1 | 1 | 1.55E+08 | C | G | -0.0566 | 0.012 | 0.4028 | 2.40E-06 | rs2990246 | 14286 |
| CDCP1 | 3 | 43423959 | T | C | 0.0757 | 0.0143 | 0.2607 | 1.20E-07 | rs34027772 | 12403 |
| CDCP1 | 3 | 58453757 | T | C | -0.148 | 0.0312 | 0.0367 | 2.10E-06 | rs35478654 | 14725 |
| CDCP1 | 19 | 11270867 | T | C | -0.0619 | 0.0125 | 0.7187 | 7.35E-07 | rs4804148 | 14734 |
| CDCP1 | 16 | 46462357 | C | G | 0.3879 | 0.084 | 0.9894 | 3.88E-06 | rs544425471 | 8827 |
| CDCP1 | 7 | 89774204 | A | G | -0.2998 | 0.0599 | 0.0121 | 5.59E-07 | rs573808300 | 11718 |
| CDCP1 | 2 | 1.48E+08 | A | G | -0.1093 | 0.0232 | 0.0652 | 2.46E-06 | rs59872649 | 14734 |
| CDCP1 | 1 | 1.02E+08 | T | C | -0.2176 | 0.0471 | 0.9829 | 3.84E-06 | rs61782461 | 14286 |
| CDCP1 | 5 | 72411335 | A | G | -0.0738 | 0.0159 | 0.8367 | 3.46E-06 | rs6452820 | 14286 |
| CDCP1 | 11 | 1.26E+08 | A | G | 0.0816 | 0.0116 | 0.4097 | 2.00E-12 | rs672806 | 14286 |
| CDCP1 | 8 | 61937553 | T | C | -0.0579 | 0.0119 | 0.6455 | 1.14E-06 | rs6991332 | 14286 |
| CDCP1 | 17 | 38720826 | T | C | -0.1323 | 0.0277 | 0.0513 | 1.79E-06 | rs74811341 | 14734 |
| CDCP1 | 18 | 19991319 | A | C | 0.0737 | 0.0144 | 0.7965 | 3.09E-07 | rs7506429 | 14286 |
| CDCP1 | 3 | 45130725 | T | C | -0.1738 | 0.0151 | 0.1811 | 1.18E-30 | rs7618923 | 14286 |
| CDCP1 | 6 | 32602396 | T | C | -0.0933 | 0.0118 | 0.6505 | 2.64E-15 | rs9272226 | 14733 |
| CSF-1 | 5 | 78775099 | C | G | 0.0583 | 0.0122 | 0.3043 | 1.76E-06 | rs10042665 | 14727 |
| CSF-1 | 2 | 1.4E+08 | A | G | -0.2277 | 0.0496 | 0.9815 | 4.42E-06 | rs115370595 | 13799 |
| CSF-1 | 2 | 27764122 | T | C | 0.0827 | 0.0173 | 0.1595 | 1.75E-06 | rs116443177 | 12403 |
| CSF-1 | 2 | 8775320 | T | C | 0.0944 | 0.0196 | 0.1197 | 1.46E-06 | rs11685487 | 14286 |
| CSF-1 | 19 | 58875573 | C | G | -0.1989 | 0.0435 | 0.0348 | 4.82E-06 | rs117591817 | 14286 |
| CSF-1 | 1 | 1.8E+08 | A | G | 0.1439 | 0.031 | 0.044 | 3.45E-06 | rs142421172 | 12840 |
| CSF-1 | 12 | 58388232 | C | G | -0.0601 | 0.0124 | 0.6484 | 1.25E-06 | rs1605333 | 13237 |
| CSF-1 | 1 | 1.11E+08 | T | C | 0.1684 | 0.0118 | 0.4821 | 3.31E-46 | rs17610659 | 14286 |
| CSF-1 | 14 | 58461604 | T | C | -0.1225 | 0.0265 | 0.9466 | 3.79E-06 | rs17734417 | 14723 |
| CSF-1 | 6 | 30075103 | T | G | -0.075 | 0.0152 | 0.8323 | 8.05E-07 | rs2523992 | 14734 |
| CSF-1 | 3 | 47239457 | A | G | 0.2087 | 0.0385 | 0.9696 | 5.93E-08 | rs563284470 | 11720 |
| CSF-1 | 18 | 45546185 | A | G | -0.0872 | 0.0185 | 0.8823 | 2.43E-06 | rs7227917 | 14286 |
| CSF-1 | 16 | 60437963 | T | C | -0.199 | 0.0414 | 0.0231 | 1.53E-06 | rs72783071 | 14247 |
| CSF-1 | 3 | 1.71E+08 | T | G | -0.0914 | 0.02 | 0.8992 | 4.88E-06 | rs74849049 | 14286 |
| CSF-1 | 1 | 1.1E+08 | A | C | 0.0775 | 0.0165 | 0.2211 | 2.64E-06 | rs7538029 | 11335 |
| CSF-1 | 14 | 78693608 | A | G | 0.1151 | 0.0239 | 0.936 | 1.47E-06 | rs75869070 | 14715 |
| CSF-1 | 3 | 1.15E+08 | A | G | 0.0778 | 0.0168 | 0.853 | 3.64E-06 | rs76421506 | 14286 |
| CSF-1 | 4 | 13713646 | C | G | -0.1189 | 0.026 | 0.0697 | 4.81E-06 | rs78652121 | 12838 |
| CST5 | 12 | 11058117 | T | C | 0.1674 | 0.013 | 0.7421 | 6.07E-38 | rs11054069 | 14724 |
| CST5 | 11 | 34860199 | T | C | 0.0702 | 0.0118 | 0.3671 | 2.70E-09 | rs12294339 | 14726 |
| CST5 | 20 | 23231946 | T | C | -0.0591 | 0.0118 | 0.5968 | 5.49E-07 | rs12625710 | 14733 |
| CST5 | 4 | 23899510 | A | G | 0.0545 | 0.0117 | 0.4095 | 3.19E-06 | rs13127297 | 14730 |
| CST5 | 2 | 62640887 | T | G | 0.0701 | 0.0141 | 0.7748 | 6.64E-07 | rs13397003 | 14732 |
| CST5 | 5 | 96079646 | T | C | 0.3373 | 0.0676 | 0.0111 | 6.05E-07 | rs139918398 | 13457 |
| CST5 | 11 | 21963583 | A | G | 0.3276 | 0.0691 | 0.0089 | 2.13E-06 | rs140505821 | 13381 |
| CST5 | 12 | 31381343 | T | C | 0.1016 | 0.0213 | 0.0812 | 1.84E-06 | rs141192864 | 14728 |
| CST5 | 5 | 59193431 | A | C | 0.0548 | 0.0119 | 0.4071 | 4.12E-06 | rs150547 | 14288 |
| CST5 | 1 | 1.02E+08 | A | G | -0.1244 | 0.0264 | 0.9481 | 2.45E-06 | rs1536565 | 14735 |
| CST5 | 8 | 11713852 | A | G | -0.0558 | 0.0121 | 0.6302 | 4.00E-06 | rs17154027 | 14288 |
| CST5 | 6 | 7051402 | A | G | 0.0885 | 0.0191 | 0.8624 | 3.60E-06 | rs3889401 | 11759 |
| CST5 | 8 | 56143053 | T | C | 0.057 | 0.0122 | 0.6482 | 2.98E-06 | rs4507745 | 13866 |
| CST5 | 18 | 24286681 | T | C | 0.0568 | 0.0119 | 0.6369 | 1.81E-06 | rs470841 | 14736 |
| CST5 | 11 | 34495358 | A | C | -0.094 | 0.0172 | 0.1273 | 4.63E-08 | rs4756151 | 14736 |
| CST5 | 12 | 1.17E+08 | A | G | -0.0574 | 0.0117 | 0.4935 | 9.30E-07 | rs4766797 | 14288 |
| CST5 | 19 | 49206145 | C | G | 0.0845 | 0.0115 | 0.4441 | 2.01E-13 | rs516316 | 14734 |
| CST5 | 20 | 23847165 | T | G | 0.6649 | 0.0137 | 0.19 | 1.00E-200 | rs6049191 | 14733 |
| CST5 | 18 | 3479423 | T | C | -0.0654 | 0.0134 | 0.3077 | 1.06E-06 | rs60586299 | 14288 |
| CST5 | 20 | 24298968 | A | G | 0.2365 | 0.0474 | 0.9818 | 6.06E-07 | rs6083489 | 14288 |
| CST5 | 6 | 1.47E+08 | A | G | 0.1194 | 0.0234 | 0.0762 | 3.35E-07 | rs62436191 | 14288 |
| CST5 | 15 | 63639644 | T | G | 0.079 | 0.0115 | 0.4609 | 6.44E-12 | rs67020211 | 14734 |
| CST5 | 20 | 51363052 | A | C | 0.1098 | 0.0237 | 0.0675 | 3.61E-06 | rs697699 | 14288 |
| CST5 | 21 | 18049996 | T | C | -0.0572 | 0.0119 | 0.6243 | 1.53E-06 | rs7275493 | 14727 |
| CST5 | 2 | 2.34E+08 | A | G | 0.0607 | 0.0126 | 0.2813 | 1.45E-06 | rs72980022 | 14721 |
| CST5 | 22 | 37630079 | A | G | -0.2516 | 0.0532 | 0.983 | 2.25E-06 | rs77121303 | 13383 |
| CST5 | 5 | 1.09E+08 | T | C | 0.073 | 0.0156 | 0.8152 | 2.88E-06 | rs79359257 | 14288 |
| CST5 | 1 | 2.02E+08 | A | G | 0.0579 | 0.0114 | 0.547 | 3.80E-07 | rs930735 | 14734 |
| CST5 | 5 | 1.35E+08 | T | C | 0.059 | 0.0118 | 0.3955 | 5.73E-07 | rs9327707 | 14710 |
| CST5 | 6 | 1.68E+08 | A | G | 0.0537 | 0.0115 | 0.4261 | 3.02E-06 | rs9455923 | 14734 |
| CST5 | 9 | 1.09E+08 | T | C | 0.0558 | 0.0119 | 0.5837 | 2.74E-06 | rs962720 | 14287 |
| CX3CL1 | 12 | 79905558 | A | C | 0.0792 | 0.0158 | 0.1687 | 5.37E-07 | rs10778675 | 14295 |
| CX3CL1 | 2 | 44117788 | C | G | -0.1191 | 0.0248 | 0.9355 | 1.57E-06 | rs111472651 | 14728 |
| CX3CL1 | 1 | 6532317 | A | G | -0.1881 | 0.0405 | 0.9747 | 3.41E-06 | rs114359340 | 13651 |
| CX3CL1 | 2 | 1.14E+08 | A | G | -0.0675 | 0.0126 | 0.5568 | 8.45E-08 | rs11682107 | 12412 |
| CX3CL1 | 15 | 85657494 | A | G | -0.1996 | 0.0432 | 0.0224 | 3.83E-06 | rs117698194 | 13859 |
| CX3CL1 | 4 | 1.87E+08 | A | G | -0.0582 | 0.0114 | 0.4914 | 3.30E-07 | rs12331618 | 14729 |
| CX3CL1 | 5 | 1.2E+08 | T | C | -0.0903 | 0.0193 | 0.89 | 2.89E-06 | rs13167439 | 14295 |
| CX3CL1 | 3 | 1260482 | C | G | -0.0569 | 0.012 | 0.3525 | 2.12E-06 | rs1976172 | 14732 |
| CX3CL1 | 6 | 31351764 | T | C | -0.0589 | 0.0117 | 0.6177 | 4.80E-07 | rs2442752 | 14743 |
| CX3CL1 | 5 | 1.77E+08 | T | G | -0.0681 | 0.0128 | 0.274 | 1.04E-07 | rs2731674 | 14740 |
| CX3CL1 | 7 | 30524015 | A | T | 0.0597 | 0.012 | 0.4243 | 6.52E-07 | rs2736723 | 14295 |
| CX3CL1 | 3 | 70136592 | T | G | 0.0633 | 0.0122 | 0.3327 | 2.12E-07 | rs35026817 | 14740 |
| CX3CL1 | 3 | 39307162 | A | G | 0.0941 | 0.0154 | 0.1671 | 9.94E-10 | rs3732378 | 14740 |
| CX3CL1 | 14 | 85801666 | T | G | -0.2257 | 0.0491 | 0.979 | 4.29E-06 | rs530057572 | 12127 |
| CX3CL1 | 16 | 57508595 | T | C | 0.1129 | 0.0244 | 0.9372 | 3.71E-06 | rs55896747 | 14734 |
| CX3CL1 | 8 | 26741279 | A | G | 0.1968 | 0.0424 | 0.9744 | 3.46E-06 | rs56397903 | 14743 |
| CX3CL1 | 9 | 88885541 | A | C | -0.2404 | 0.0512 | 0.981 | 2.66E-06 | rs571946532 | 11768 |
| CX3CL1 | 9 | 1.36E+08 | T | C | -0.1145 | 0.0166 | 0.1884 | 5.29E-12 | rs635634 | 11792 |
| CX3CL1 | 16 | 57412802 | C | G | 0.2041 | 0.0118 | 0.424 | 4.99E-67 | rs671623 | 14295 |
| CX3CL1 | 12 | 41381284 | A | C | 0.0563 | 0.012 | 0.6601 | 2.71E-06 | rs712137 | 14737 |
| CX3CL1 | 20 | 52569781 | C | G | 0.2442 | 0.0529 | 0.9812 | 3.91E-06 | rs73137770 | 13808 |
| CX3CL1 | 6 | 32424882 | T | C | 0.0811 | 0.012 | 0.3484 | 1.40E-11 | rs7763262 | 14743 |
| CX3CL1 | 1 | 1.72E+08 | A | G | -0.1436 | 0.0304 | 0.0402 | 2.32E-06 | rs77915710 | 14742 |
| CX3CL1 | 16 | 57167230 | A | G | -0.1511 | 0.027 | 0.9463 | 2.19E-08 | rs8052631 | 14295 |
| CX3CL1 | 4 | 1.8E+08 | A | G | 0.1479 | 0.0301 | 0.9343 | 8.94E-07 | rs980364 | 9902 |
| CXCL1 | 11 | 20486401 | A | G | 0.123 | 0.0267 | 0.067 | 4.09E-06 | rs10734292 | 11776 |
| CXCL1 | 19 | 14808453 | A | G | 0.0583 | 0.0127 | 0.3161 | 4.42E-06 | rs11880837 | 14288 |
| CXCL1 | 10 | 65297720 | A | C | 0.0648 | 0.0126 | 0.321 | 2.71E-07 | rs12770839 | 14724 |
| CXCL1 | 4 | 74738469 | T | C | -0.3134 | 0.0134 | 0.7638 | 5.65E-121 | rs1366946 | 14730 |
| CXCL1 | 4 | 59689961 | A | G | -0.0538 | 0.0117 | 0.5637 | 4.26E-06 | rs2122155 | 14717 |
| CXCL1 | 15 | 81240669 | A | G | -0.1443 | 0.0312 | 0.0405 | 3.75E-06 | rs2280441 | 14735 |
| CXCL1 | 21 | 39102421 | C | G | -0.0564 | 0.012 | 0.3876 | 2.60E-06 | rs2835916 | 14735 |
| CXCL1 | 21 | 39102421 | C | G | -0.0564 | 0.012 | 0.3876 | 2.60E-06 | rs2835916 | 14735 |
| CXCL1 | 13 | 37854253 | T | C | -0.365 | 0.0779 | 0.0146 | 2.79E-06 | rs4474574 | 9555 |
| CXCL1 | 4 | 84005961 | T | C | -0.186 | 0.0407 | 0.9588 | 4.88E-06 | rs62311721 | 14288 |
| CXCL1 | 9 | 4396102 | C | G | 0.0822 | 0.0179 | 0.1691 | 4.39E-06 | rs67797811 | 14288 |
| CXCL1 | 6 | 54641812 | A | G | -0.1047 | 0.0206 | 0.8675 | 3.72E-07 | rs77914035 | 10893 |
| CXCL1 | 1 | 1.59E+08 | T | C | 0.0661 | 0.0119 | 0.3809 | 2.78E-08 | rs863004 | 14722 |
| CXCL10 | 9 | 84196490 | T | C | -0.0548 | 0.0119 | 0.4567 | 4.12E-06 | rs10481754 | 14296 |
| CXCL10 | 15 | 80065337 | A | G | -0.0546 | 0.0118 | 0.5648 | 3.71E-06 | rs1077965 | 14731 |
| CXCL10 | 1 | 44566391 | A | G | -0.1609 | 0.0336 | 0.9671 | 1.68E-06 | rs112202639 | 14744 |
| CXCL10 | 4 | 76910673 | T | C | -0.5186 | 0.042 | 0.9732 | 5.02E-35 | rs115140093 | 14744 |
| CXCL10 | 7 | 3425301 | T | C | -0.1765 | 0.0375 | 0.0284 | 2.52E-06 | rs12155428 | 14733 |
| CXCL10 | 6 | 88557407 | A | G | -0.0582 | 0.0121 | 0.6254 | 1.51E-06 | rs12174864 | 14295 |
| CXCL10 | 2 | 14400474 | T | C | 0.0536 | 0.0115 | 0.4864 | 3.15E-06 | rs12476448 | 14737 |
| CXCL10 | 3 | 54104178 | T | G | -0.0879 | 0.0182 | 0.1209 | 1.37E-06 | rs12629593 | 14744 |
| CXCL10 | 4 | 77175245 | T | C | 0.0977 | 0.0136 | 0.246 | 6.78E-13 | rs12646113 | 14743 |
| CXCL10 | 9 | 4150061 | C | G | -0.2858 | 0.062 | 0.012 | 4.03E-06 | rs143796249 | 14296 |
| CXCL10 | 22 | 50774821 | T | C | -0.1841 | 0.0376 | 0.9688 | 9.77E-07 | rs150519056 | 13661 |
| CXCL10 | 2 | 3891773 | A | G | 0.0592 | 0.0127 | 0.7042 | 3.14E-06 | rs1563171 | 14740 |
| CXCL10 | 8 | 62003730 | A | G | -0.0789 | 0.0164 | 0.2555 | 1.50E-06 | rs2349689 | 14296 |
| CXCL10 | 6 | 31377978 | T | C | 0.1095 | 0.0196 | 0.1048 | 2.31E-08 | rs2523495 | 14743 |
| CXCL10 | 12 | 1.12E+08 | T | C | 0.1138 | 0.0131 | 0.4756 | 3.72E-18 | rs3184504 | 11793 |
| CXCL10 | 10 | 1845059 | T | C | -0.0567 | 0.0119 | 0.5587 | 1.89E-06 | rs4367871 | 14296 |
| CXCL10 | 19 | 11270867 | T | C | -0.0629 | 0.013 | 0.7227 | 1.31E-06 | rs4804148 | 14744 |
| CXCL10 | 11 | 73731422 | A | G | 0.1175 | 0.025 | 0.929 | 2.60E-06 | rs55732300 | 14730 |
| CXCL10 | 6 | 32516855 | T | C | -0.2991 | 0.0606 | 0.9813 | 7.99E-07 | rs71534595 | 11719 |
| CXCL10 | 4 | 76808216 | T | C | -0.6096 | 0.0619 | 0.9892 | 6.98E-23 | rs72651343 | 14257 |
| CXCL10 | 4 | 1.13E+08 | A | G | 0.1089 | 0.0209 | 0.0979 | 1.88E-07 | rs72682331 | 14731 |
| CXCL10 | 12 | 68597086 | A | G | -0.06 | 0.013 | 0.7036 | 3.92E-06 | rs741344 | 14296 |
| CXCL10 | 19 | 10427721 | A | T | -0.1358 | 0.0294 | 0.0495 | 3.86E-06 | rs74956615 | 13671 |
| CXCL11 | 10 | 64948684 | T | C | -0.0815 | 0.0127 | 0.6875 | 1.39E-10 | rs10733789 | 14287 |
| CXCL11 | 11 | 83554112 | A | G | 0.0609 | 0.0121 | 0.6028 | 4.83E-07 | rs10898158 | 14288 |
| CXCL11 | 7 | 1.58E+08 | A | C | -0.1445 | 0.0314 | 0.951 | 4.19E-06 | rs111787182 | 13224 |
| CXCL11 | 11 | 22351553 | A | G | -0.1162 | 0.025 | 0.9044 | 3.35E-06 | rs111848253 | 11538 |
| CXCL11 | 4 | 1.34E+08 | T | C | -0.0976 | 0.0209 | 0.0892 | 3.01E-06 | rs112884138 | 14728 |
| CXCL11 | 4 | 77374393 | T | C | -0.1975 | 0.0427 | 0.0216 | 3.74E-06 | rs116470923 | 14736 |
| CXCL11 | 14 | 50775476 | A | G | 0.0571 | 0.012 | 0.4282 | 1.95E-06 | rs12433317 | 14288 |
| CXCL11 | 20 | 17839484 | A | G | -0.3323 | 0.0684 | 0.0117 | 1.18E-06 | rs191536201 | 11194 |
| CXCL11 | 20 | 4795382 | A | G | -0.0599 | 0.0131 | 0.6525 | 4.82E-06 | rs2245786 | 14721 |
| CXCL11 | 6 | 32011678 | A | G | -0.2236 | 0.047 | 0.9724 | 1.96E-06 | rs2894233 | 11917 |
| CXCL11 | 12 | 1.12E+08 | T | C | 0.1236 | 0.0132 | 0.4764 | 7.70E-21 | rs3184504 | 11785 |
| CXCL11 | 10 | 17590681 | T | C | 0.0901 | 0.019 | 0.1188 | 2.12E-06 | rs4748380 | 14732 |
| CXCL11 | 4 | 77130802 | A | G | 0.1821 | 0.0378 | 0.0278 | 1.45E-06 | rs59148364 | 14721 |
| CXCL11 | 1 | 26395741 | A | G | -0.0777 | 0.0165 | 0.1703 | 2.49E-06 | rs60141220 | 13221 |
| CXCL11 | 3 | 1.88E+08 | C | G | 0.2674 | 0.0518 | 0.9763 | 2.44E-07 | rs62291421 | 12305 |
| CXCL11 | 4 | 76916146 | A | G | -0.1724 | 0.0118 | 0.6041 | 2.42E-48 | rs6827617 | 14718 |
| CXCL11 | 8 | 96176796 | T | C | 0.1175 | 0.025 | 0.9321 | 2.60E-06 | rs7009937 | 14288 |
| CXCL11 | 17 | 34335694 | T | C | -0.0882 | 0.0182 | 0.1205 | 1.26E-06 | rs7222922 | 14726 |
| CXCL11 | 5 | 18009856 | A | G | 0.1535 | 0.0326 | 0.9608 | 2.49E-06 | rs72747175 | 14730 |
| CXCL11 | 5 | 18009856 | A | G | 0.1535 | 0.0326 | 0.9608 | 2.49E-06 | rs72747175 | 14730 |
| CXCL11 | 6 | 89498971 | A | G | -0.0973 | 0.0185 | 0.1651 | 1.44E-07 | rs72925566 | 11337 |
| CXCL11 | 10 | 1.04E+08 | A | C | -0.0646 | 0.0124 | 0.3172 | 1.89E-07 | rs7475335 | 14735 |
| CXCL11 | 7 | 1.02E+08 | T | C | 0.5352 | 0.0705 | 0.0084 | 3.16E-14 | rs75612655 | 12935 |
| CXCL11 | 6 | 1.54E+08 | T | C | -0.0773 | 0.0164 | 0.165 | 2.44E-06 | rs7750544 | 13644 |
| CXCL11 | 8 | 70982905 | T | C | 0.1082 | 0.0214 | 0.9152 | 4.28E-07 | rs78632565 | 14722 |
| CXCL5 | 19 | 14764810 | A | G | -0.0601 | 0.0127 | 0.2925 | 2.22E-06 | rs10403201 | 14730 |
| CXCL5 | 9 | 1.01E+08 | A | C | -0.0619 | 0.0121 | 0.6443 | 3.13E-07 | rs10760022 | 14734 |
| CXCL5 | 9 | 1.37E+08 | A | C | -0.1132 | 0.0156 | 0.3353 | 3.97E-13 | rs10821552 | 9841 |
| CXCL5 | 4 | 6891435 | A | G | 0.0769 | 0.0153 | 0.1845 | 5.00E-07 | rs11734099 | 14731 |
| CXCL5 | 13 | 53109423 | A | G | -0.1474 | 0.0322 | 0.9544 | 4.70E-06 | rs140828666 | 12401 |
| CXCL5 | 3 | 1.84E+08 | A | G | 0.1844 | 0.0377 | 0.0265 | 1.00E-06 | rs146553494 | 14733 |
| CXCL5 | 8 | 1.45E+08 | T | C | 0.0675 | 0.0147 | 0.2492 | 4.39E-06 | rs2004100 | 13224 |
| CXCL5 | 6 | 76351999 | C | G | 0.0631 | 0.013 | 0.2784 | 1.21E-06 | rs2647400 | 14734 |
| CXCL5 | 4 | 74863997 | T | C | 0.5063 | 0.0181 | 0.1127 | 3.52E-172 | rs425535 | 14731 |
| CXCL5 | 1 | 2.11E+08 | T | C | 0.2082 | 0.042 | 0.0209 | 7.15E-07 | rs4279906 | 14717 |
| CXCL5 | 16 | 88582345 | A | G | -0.19 | 0.0385 | 0.0309 | 8.01E-07 | rs59312158 | 13672 |
| CXCL5 | 8 | 1.07E+08 | A | T | -0.1058 | 0.0135 | 0.7312 | 4.61E-15 | rs6993770 | 14288 |
| CXCL5 | 10 | 65077994 | C | G | -0.1734 | 0.0117 | 0.5834 | 1.08E-49 | rs7090111 | 14735 |
| CXCL5 | 3 | 56874033 | A | G | -0.0768 | 0.0137 | 0.7593 | 2.07E-08 | rs7636889 | 14733 |
| CXCL5 | 6 | 54641812 | A | G | -0.1174 | 0.0206 | 0.8675 | 1.20E-08 | rs77914035 | 10893 |
| CXCL5 | 4 | 74880852 | T | G | -0.1721 | 0.0276 | 0.0605 | 4.50E-10 | rs79649467 | 14288 |
| CXCL6 | 16 | 2222286 | T | C | 0.1285 | 0.0272 | 0.0649 | 2.31E-06 | rs11547311 | 13232 |
| CXCL6 | 21 | 39099887 | C | G | 0.1897 | 0.0415 | 0.0242 | 4.85E-06 | rs117109857 | 14743 |
| CXCL6 | 1 | 1.59E+08 | A | G | 0.0816 | 0.0115 | 0.5554 | 1.29E-12 | rs12075 | 14741 |
| CXCL6 | 4 | 74689945 | A | T | -0.3628 | 0.0117 | 0.5391 | 1.00E-200 | rs12503139 | 14296 |
| CXCL6 | 8 | 93838957 | A | G | -0.3831 | 0.0799 | 0.0074 | 1.63E-06 | rs182381042 | 12935 |
| CXCL6 | 1 | 2.22E+08 | T | C | -0.1603 | 0.0351 | 0.039 | 4.95E-06 | rs184103539 | 12841 |
| CXCL6 | 4 | 74290159 | A | G | 0.4281 | 0.0872 | 0.0091 | 9.14E-07 | rs186000650 | 10429 |
| CXCL6 | 4 | 74763057 | T | C | -0.1069 | 0.0135 | 0.7516 | 2.40E-15 | rs2886969 | 14743 |
| CXCL6 | 12 | 1.12E+08 | T | C | 0.0679 | 0.0131 | 0.4766 | 2.18E-07 | rs3184504 | 11793 |
| CXCL6 | 14 | 89402542 | T | G | 0.0605 | 0.0131 | 0.715 | 3.87E-06 | rs34080302 | 14296 |
| CXCL6 | 4 | 75172482 | T | C | 0.2109 | 0.0427 | 0.0308 | 7.85E-07 | rs35096759 | 14296 |
| CXCL6 | 9 | 86683575 | T | G | 0.0625 | 0.0134 | 0.5188 | 3.10E-06 | rs4877818 | 11538 |
| CXCL6 | 1 | 2.11E+08 | T | G | -0.215 | 0.0457 | 0.9774 | 2.54E-06 | rs4951518 | 11323 |
| CXCL6 | 10 | 35485924 | A | T | 0.3432 | 0.0692 | 0.9894 | 7.07E-07 | rs574432935 | 12778 |
| CXCL6 | 4 | 74602553 | T | C | -0.2245 | 0.0459 | 0.0228 | 1.00E-06 | rs79640485 | 14744 |
| CXCL6 | 6 | 1.54E+08 | T | C | -0.0919 | 0.0193 | 0.1328 | 1.92E-06 | rs9371716 | 12412 |
| CXCL9 | 2 | 86977227 | T | G | -0.2245 | 0.0488 | 0.981 | 4.22E-06 | rs114033894 | 14287 |
| CXCL9 | 6 | 1.61E+08 | A | G | -0.1452 | 0.0197 | 0.098 | 1.70E-13 | rs12191307 | 14287 |
| CXCL9 | 7 | 48859798 | T | C | 0.2705 | 0.0551 | 0.9851 | 9.14E-07 | rs142119726 | 13800 |
| CXCL9 | 5 | 19287867 | T | C | 0.0728 | 0.0154 | 0.1625 | 2.28E-06 | rs145607633 | 14287 |
| CXCL9 | 6 | 31423412 | A | G | -0.1603 | 0.0342 | 0.0378 | 2.77E-06 | rs181816009 | 11985 |
| CXCL9 | 16 | 1803589 | T | C | 0.1861 | 0.0385 | 0.028 | 1.34E-06 | rs182152471 | 13667 |
| CXCL9 | 12 | 1.12E+08 | T | C | 0.1008 | 0.013 | 0.4744 | 8.92E-15 | rs3184504 | 11784 |
| CXCL9 | 2 | 2.43E+08 | A | G | 0.0915 | 0.02 | 0.1125 | 4.76E-06 | rs35435997 | 12791 |
| CXCL9 | 4 | 76930776 | A | C | -0.1419 | 0.0128 | 0.2629 | 1.47E-28 | rs4241577 | 14724 |
| CXCL9 | 16 | 79431853 | C | G | -0.157 | 0.0343 | 0.9683 | 4.71E-06 | rs4888966 | 14287 |
| CXCL9 | 10 | 1.08E+08 | A | G | -0.0758 | 0.0158 | 0.1611 | 1.61E-06 | rs61875680 | 14287 |
| CXCL9 | 20 | 20119931 | A | C | -0.115 | 0.0244 | 0.9336 | 2.44E-06 | rs62200304 | 14287 |
| CXCL9 | 3 | 1.52E+08 | A | G | -0.0834 | 0.0176 | 0.1182 | 2.15E-06 | rs62272916 | 14710 |
| CXCL9 | 7 | 29049949 | A | G | 0.0562 | 0.0119 | 0.6566 | 2.33E-06 | rs656739 | 14735 |
| CXCL9 | 14 | 74739091 | A | T | -0.0574 | 0.0115 | 0.5236 | 6.00E-07 | rs7149776 | 14286 |
| CXCL9 | 21 | 18734554 | A | G | 0.1821 | 0.0391 | 0.9758 | 3.20E-06 | rs73194812 | 14730 |
| CXCL9 | 21 | 45024252 | T | C | -0.0588 | 0.0126 | 0.5804 | 3.06E-06 | rs73367822 | 11760 |
| CXCL9 | 20 | 2538467 | A | C | 0.2792 | 0.059 | 0.0133 | 2.22E-06 | rs73606147 | 13379 |
| CXCL9 | 19 | 10427721 | A | T | -0.1309 | 0.0286 | 0.049 | 4.72E-06 | rs74956615 | 13662 |
| CXCL9 | 2 | 2.05E+08 | T | C | 0.057 | 0.0118 | 0.5214 | 1.36E-06 | rs7600322 | 13849 |
| CXCL9 | 6 | 1.61E+08 | A | C | -0.0805 | 0.0144 | 0.1958 | 2.27E-08 | rs783149 | 14287 |
| CXCL9 | 4 | 1.61E+08 | T | C | 0.1546 | 0.0332 | 0.0336 | 3.21E-06 | rs80348644 | 14734 |
| CXCL9 | 6 | 31269173 | A | G | -0.0605 | 0.0115 | 0.4075 | 1.43E-07 | rs9366778 | 14735 |
| DNER | 2 | 41864408 | T | C | -0.0591 | 0.0125 | 0.4361 | 2.27E-06 | rs10191831 | 12403 |
| DNER | 11 | 1.2E+08 | C | G | 0.0808 | 0.0175 | 0.1236 | 3.89E-06 | rs10892466 | 14732 |
| DNER | 18 | 3722038 | A | G | -0.0664 | 0.0142 | 0.2533 | 2.92E-06 | rs1133851 | 14286 |
| DNER | 12 | 31458615 | T | C | -0.2481 | 0.0537 | 0.0138 | 3.84E-06 | rs116179073 | 13800 |
| DNER | 7 | 1.52E+08 | C | G | -0.1281 | 0.0274 | 0.942 | 2.94E-06 | rs117343254 | 14287 |
| DNER | 4 | 1.03E+08 | T | C | 0.1066 | 0.0233 | 0.0697 | 4.76E-06 | rs13107325 | 14732 |
| DNER | 14 | 69282887 | T | C | 0.0528 | 0.0112 | 0.4849 | 2.43E-06 | rs194746 | 14723 |
| DNER | 2 | 2.3E+08 | A | G | 0.1016 | 0.0117 | 0.5359 | 3.83E-18 | rs207671 | 14287 |
| DNER | 21 | 42619544 | A | C | 0.0576 | 0.0118 | 0.3578 | 1.05E-06 | rs2837988 | 14733 |
| DNER | 17 | 47247257 | T | C | 0.0918 | 0.0184 | 0.1074 | 6.07E-07 | rs28689968 | 14733 |
| DNER | 2 | 2.3E+08 | T | G | 0.132 | 0.0132 | 0.7027 | 1.52E-23 | rs35032874 | 14287 |
| DNER | 2 | 2.31E+08 | A | T | 0.213 | 0.0123 | 0.6507 | 3.50E-67 | rs62193248 | 14287 |
| DNER | 21 | 42536246 | C | G | 0.0757 | 0.0129 | 0.289 | 4.41E-09 | rs6517655 | 14287 |
| DNER | 14 | 95752666 | T | C | 0.0879 | 0.0186 | 0.1139 | 2.29E-06 | rs75685422 | 14287 |
| DNER | 3 | 1.4E+08 | T | C | -0.0652 | 0.0142 | 0.7033 | 4.40E-06 | rs9289590 | 12404 |
| DNER | 21 | 28101979 | T | G | 0.0718 | 0.0152 | 0.1718 | 2.32E-06 | rs9967959 | 14731 |
| EN-RAGE | 12 | 96571503 | T | C | 0.1884 | 0.0407 | 0.0246 | 3.67E-06 | rs112956934 | 14737 |
| EN-RAGE | 14 | 59990514 | T | C | -0.1396 | 0.0293 | 0.9492 | 1.89E-06 | rs112988802 | 12851 |
| EN-RAGE | 2 | 1.25E+08 | T | C | -0.0622 | 0.0133 | 0.7266 | 2.92E-06 | rs13384799 | 14295 |
| EN-RAGE | 19 | 4247567 | C | G | 0.0865 | 0.0183 | 0.191 | 2.28E-06 | rs138439507 | 11537 |
| EN-RAGE | 16 | 4602209 | A | G | 0.1806 | 0.0345 | 0.0379 | 1.65E-07 | rs147302029 | 13649 |
| EN-RAGE | 3 | 1.75E+08 | A | C | 0.194 | 0.0423 | 0.0217 | 4.51E-06 | rs150015576 | 14740 |
| EN-RAGE | 22 | 18032226 | A | G | 0.0617 | 0.0125 | 0.6296 | 7.97E-07 | rs174344 | 14295 |
| EN-RAGE | 7 | 75133433 | A | C | 0.3059 | 0.0665 | 0.983 | 4.22E-06 | rs191808657 | 12412 |
| EN-RAGE | 4 | 12920594 | T | C | -0.0604 | 0.0119 | 0.613 | 3.86E-07 | rs2056031 | 14739 |
| EN-RAGE | 20 | 43028037 | A | G | -0.0583 | 0.0123 | 0.3511 | 2.14E-06 | rs2425640 | 14741 |
| EN-RAGE | 1 | 1.53E+08 | A | G | -0.1726 | 0.0137 | 0.2623 | 2.15E-36 | rs3014874 | 14294 |
| EN-RAGE | 8 | 1.24E+08 | A | G | 0.0555 | 0.0118 | 0.5633 | 2.56E-06 | rs3802266 | 14740 |
| EN-RAGE | 22 | 17406250 | T | C | 0.2064 | 0.0425 | 0.0239 | 1.19E-06 | rs55783131 | 14728 |
| EN-RAGE | 3 | 1.53E+08 | T | C | -0.0936 | 0.0203 | 0.0955 | 4.01E-06 | rs56268787 | 14737 |
| EN-RAGE | 1 | 2.45E+08 | C | G | -0.1188 | 0.0258 | 0.0622 | 4.13E-06 | rs59538998 | 14295 |
| EN-RAGE | 1 | 1.53E+08 | T | G | 0.1501 | 0.0194 | 0.1069 | 1.02E-14 | rs61803119 | 14295 |
| EN-RAGE | 11 | 31879143 | A | G | -0.2529 | 0.0534 | 0.0156 | 2.18E-06 | rs78800546 | 14295 |
| FGF-19 | 12 | 91080475 | A | G | -0.0767 | 0.0166 | 0.1923 | 3.83E-06 | rs10777251 | 12411 |
| FGF-19 | 3 | 1.32E+08 | A | G | -0.0927 | 0.0199 | 0.1012 | 3.19E-06 | rs112126444 | 14735 |
| FGF-19 | 15 | 59188192 | T | C | 0.2583 | 0.0564 | 0.0178 | 4.65E-06 | rs117398067 | 14744 |
| FGF-19 | 6 | 1.69E+08 | T | C | -0.136 | 0.0297 | 0.0418 | 4.67E-06 | rs12110436 | 14743 |
| FGF-19 | 1 | 85295202 | A | G | -0.1614 | 0.0352 | 0.9664 | 4.53E-06 | rs12407505 | 14735 |
| FGF-19 | 5 | 54847098 | T | C | -0.1014 | 0.0221 | 0.9175 | 4.47E-06 | rs12520287 | 14296 |
| FGF-19 | 4 | 39457617 | A | G | 0.0837 | 0.0124 | 0.3415 | 1.48E-11 | rs13103023 | 14296 |
| FGF-19 | 12 | 76644195 | A | G | -0.2295 | 0.0492 | 0.0198 | 3.09E-06 | rs142067135 | 13809 |
| FGF-19 | 7 | 17445553 | T | C | 0.1992 | 0.0421 | 0.9777 | 2.23E-06 | rs145274424 | 14735 |
| FGF-19 | 11 | 1.14E+08 | T | C | 0.0621 | 0.0132 | 0.6316 | 2.54E-06 | rs1671812 | 12412 |
| FGF-19 | 13 | 85357827 | T | C | 0.2161 | 0.0471 | 0.0209 | 4.47E-06 | rs17326790 | 14741 |
| FGF-19 | 11 | 5610048 | T | C | 0.2325 | 0.0488 | 0.9812 | 1.89E-06 | rs191522330 | 14738 |
| FGF-19 | 19 | 5851801 | A | G | 0.0674 | 0.0141 | 0.2338 | 1.75E-06 | rs2306969 | 14739 |
| FGF-19 | 7 | 1.02E+08 | T | C | -0.0623 | 0.0135 | 0.2503 | 3.93E-06 | rs2906655 | 14743 |
| FGF-19 | 9 | 84435749 | A | G | 0.2151 | 0.0456 | 0.0203 | 2.39E-06 | rs35836706 | 14734 |
| FGF-19 | 19 | 49206172 | T | C | -0.1661 | 0.0118 | 0.445 | 5.31E-45 | rs516246 | 14744 |
| FGF-19 | 3 | 74674996 | A | G | 0.2371 | 0.0482 | 0.9755 | 8.69E-07 | rs574352299 | 10894 |
| FGF-19 | 8 | 59382715 | A | G | -0.1055 | 0.0123 | 0.3458 | 9.72E-18 | rs7005978 | 14744 |
| FGF-19 | 12 | 1.08E+08 | T | C | 0.1024 | 0.0222 | 0.9158 | 3.98E-06 | rs76708593 | 14296 |
| FGF-19 | 9 | 18148302 | A | G | 0.073 | 0.0156 | 0.1762 | 2.88E-06 | rs79110990 | 14295 |
| FGF-19 | 1 | 1E+08 | A | G | -0.0614 | 0.0128 | 0.3002 | 1.61E-06 | rs834971 | 14728 |
| FGF-21 | 1 | 2.17E+08 | T | G | -0.1557 | 0.0318 | 0.0383 | 9.77E-07 | rs10495032 | 14720 |
| FGF-21 | 11 | 76480255 | T | C | -0.0689 | 0.0145 | 0.2168 | 2.02E-06 | rs12290350 | 14295 |
| FGF-21 | 2 | 27730940 | T | C | 0.1323 | 0.0119 | 0.3977 | 1.03E-28 | rs1260326 | 14730 |
| FGF-21 | 7 | 73030175 | A | G | -0.1609 | 0.0176 | 0.1297 | 6.13E-20 | rs13229619 | 14743 |
| FGF-21 | 21 | 33860969 | T | C | -0.2352 | 0.0455 | 0.0232 | 2.35E-07 | rs142603673 | 14252 |
| FGF-21 | 1 | 2.04E+08 | A | G | -0.1747 | 0.036 | 0.037 | 1.22E-06 | rs145127946 | 14295 |
| FGF-21 | 10 | 1.28E+08 | A | G | -0.3801 | 0.0749 | 0.0104 | 3.88E-07 | rs188758663 | 11668 |
| FGF-21 | 12 | 47198899 | A | C | -0.0812 | 0.0147 | 0.795 | 3.32E-08 | rs2429473 | 14295 |
| FGF-21 | 5 | 55882435 | T | C | -0.063 | 0.0136 | 0.2698 | 3.62E-06 | rs4700382 | 14295 |
| FGF-21 | 5 | 84356011 | T | C | -0.0881 | 0.019 | 0.8922 | 3.54E-06 | rs60277384 | 14742 |
| FGF-21 | 8 | 9173209 | A | G | -0.0605 | 0.012 | 0.4812 | 4.61E-07 | rs7012637 | 14295 |
| FGF-21 | 18 | 72826607 | A | G | -0.2215 | 0.0444 | 0.031 | 6.08E-07 | rs72965996 | 13808 |
| FGF-21 | 3 | 11689679 | T | C | -0.0568 | 0.012 | 0.4644 | 2.21E-06 | rs7610704 | 14295 |
| FGF-21 | 19 | 49260677 | A | C | 0.1627 | 0.013 | 0.501 | 6.15E-36 | rs838131 | 14294 |
| FGF-23 | 16 | 83137317 | T | C | -0.056 | 0.0119 | 0.4938 | 2.53E-06 | rs11150539 | 14287 |
| FGF-23 | 11 | 1.07E+08 | A | G | 0.2362 | 0.0507 | 0.0187 | 3.18E-06 | rs118177380 | 14287 |
| FGF-23 | 4 | 1.24E+08 | A | T | -0.1845 | 0.0389 | 0.9748 | 2.11E-06 | rs11930290 | 12848 |
| FGF-23 | 6 | 81343345 | A | G | -0.0768 | 0.0166 | 0.8634 | 3.72E-06 | rs12195292 | 14710 |
| FGF-23 | 6 | 31175118 | A | C | -0.2224 | 0.0479 | 0.0191 | 3.43E-06 | rs181409232 | 12846 |
| FGF-23 | 5 | 1.77E+08 | A | G | -0.0653 | 0.0135 | 0.2766 | 1.32E-06 | rs33921462 | 14287 |
| FGF-23 | 5 | 1.47E+08 | A | G | 0.1704 | 0.0348 | 0.0295 | 9.75E-07 | rs34551523 | 14730 |
| FGF-23 | 22 | 22238107 | T | C | 0.0803 | 0.0166 | 0.1851 | 1.32E-06 | rs35590112 | 12386 |
| FGF-23 | 7 | 12540548 | C | G | 0.1059 | 0.0222 | 0.1002 | 1.84E-06 | rs370203222 | 11759 |
| FGF-23 | 2 | 1.9E+08 | C | G | 0.1163 | 0.0131 | 0.2897 | 6.82E-19 | rs3811621 | 14286 |
| FGF-23 | 20 | 52731402 | A | T | 0.0907 | 0.0134 | 0.706 | 1.30E-11 | rs6127099 | 14287 |
| FGF-23 | 1 | 1.47E+08 | T | C | 0.0708 | 0.0136 | 0.7502 | 1.93E-07 | rs671205 | 14287 |
| FGF-23 | 2 | 1.22E+08 | T | G | 0.0941 | 0.0185 | 0.8733 | 3.65E-07 | rs6753743 | 14287 |
| FGF-23 | 14 | 22607677 | A | G | -0.1085 | 0.0235 | 0.8871 | 3.89E-06 | rs75338333 | 10893 |
| FGF-23 | 4 | 40142535 | T | C | 0.0685 | 0.0144 | 0.2406 | 1.97E-06 | rs794007 | 12844 |
| FGF-23 | 12 | 4479549 | A | G | -0.0959 | 0.0178 | 0.1141 | 7.14E-08 | rs7955866 | 14719 |
| FGF-5 | 4 | 81169912 | T | G | 0.5176 | 0.0126 | 0.3175 | 1.00E-200 | rs11099098 | 11789 |
| FGF-5 | 1 | 2.16E+08 | T | C | -0.0599 | 0.0127 | 0.6038 | 2.40E-06 | rs11120686 | 11786 |
| FGF-5 | 12 | 48680454 | A | G | 0.509 | 0.1062 | 0.006 | 1.64E-06 | rs112790107 | 9845 |
| FGF-5 | 2 | 45095097 | A | G | -0.0586 | 0.0128 | 0.364 | 4.69E-06 | rs11674185 | 11787 |
| FGF-5 | 9 | 7476426 | T | C | 0.2266 | 0.0453 | 0.0223 | 5.67E-07 | rs117253850 | 11781 |
| FGF-5 | 9 | 1.33E+08 | T | C | 0.1509 | 0.0322 | 0.0458 | 2.78E-06 | rs12005489 | 10725 |
| FGF-5 | 9 | 1354486 | A | G | -0.0909 | 0.0192 | 0.8798 | 2.20E-06 | rs1412253 | 11778 |
| FGF-5 | 2 | 1.45E+08 | T | C | 0.2883 | 0.0608 | 0.0166 | 2.12E-06 | rs148332905 | 11341 |
| FGF-5 | 6 | 89057163 | A | G | -0.1705 | 0.0361 | 0.036 | 2.32E-06 | rs148797749 | 11785 |
| FGF-5 | 8 | 1.06E+08 | A | G | 0.2554 | 0.0552 | 0.9848 | 3.71E-06 | rs150524893 | 11298 |
| FGF-5 | 4 | 81152728 | C | G | 0.1719 | 0.0187 | 0.8489 | 3.84E-20 | rs17004843 | 11341 |
| FGF-5 | 2 | 2.3E+08 | T | C | -0.3042 | 0.0659 | 0.0146 | 3.91E-06 | rs189846846 | 9980 |
| FGF-5 | 18 | 14708913 | A | G | -0.1842 | 0.0395 | 0.0464 | 3.11E-06 | rs192746810 | 11341 |
| FGF-5 | 6 | 39710570 | T | C | 0.1185 | 0.0249 | 0.0828 | 1.95E-06 | rs305897 | 11341 |
| FGF-5 | 2 | 2.42E+08 | A | G | -0.0988 | 0.0213 | 0.1585 | 3.51E-06 | rs369482528 | 8813 |
| FGF-5 | 4 | 81192363 | A | G | 0.1629 | 0.0283 | 0.9341 | 8.60E-09 | rs3796605 | 11782 |
| FGF-5 | 4 | 70058335 | A | C | -0.0728 | 0.015 | 0.2193 | 1.21E-06 | rs4326078 | 11789 |
| FGF-5 | 1 | 1.1E+08 | T | C | -0.0729 | 0.0155 | 0.2041 | 2.56E-06 | rs4970834 | 11786 |
| FGF-5 | 16 | 62005709 | A | G | -0.1197 | 0.0249 | 0.0816 | 1.53E-06 | rs56391958 | 11341 |
| FGF-5 | 4 | 81817429 | A | T | 0.1406 | 0.0289 | 0.0656 | 1.14E-06 | rs62302222 | 11341 |
| FGF-5 | 11 | 75754620 | T | G | 0.0619 | 0.0133 | 0.4196 | 3.25E-06 | rs624605 | 11341 |
| FGF-5 | 13 | 62078600 | T | C | -0.0826 | 0.0165 | 0.1699 | 5.56E-07 | rs66613644 | 11787 |
| FGF-5 | 8 | 1.43E+08 | A | T | 0.1673 | 0.0356 | 0.9648 | 2.61E-06 | rs77210320 | 11786 |
| FGF-5 | 1 | 1.02E+08 | A | G | 0.1023 | 0.0224 | 0.0841 | 4.95E-06 | rs9433760 | 11788 |
| FIt3L | 18 | 70532840 | T | G | 0.0556 | 0.0121 | 0.408 | 4.33E-06 | rs10164255 | 14729 |
| FIt3L | 11 | 1.08E+08 | A | G | -0.0772 | 0.0117 | 0.4171 | 4.16E-11 | rs11212636 | 14723 |
| FIt3L | 9 | 94348687 | T | C | -0.0851 | 0.0186 | 0.1096 | 4.76E-06 | rs12338549 | 14719 |
| FIt3L | 4 | 57902945 | A | T | 0.0539 | 0.0117 | 0.4587 | 4.09E-06 | rs1277312 | 14719 |
| FIt3L | 6 | 91011673 | T | C | 0.0676 | 0.0128 | 0.3278 | 1.28E-07 | rs1321859 | 14286 |
| FIt3L | 5 | 6289394 | T | C | 0.0603 | 0.0127 | 0.3741 | 2.05E-06 | rs1379304 | 14286 |
| FIt3L | 11 | 62294309 | T | C | -0.1519 | 0.0331 | 0.0364 | 4.45E-06 | rs141117375 | 14286 |
| FIt3L | 20 | 4261712 | T | C | -0.4307 | 0.0883 | 0.0075 | 1.07E-06 | rs142870956 | 11952 |
| FIt3L | 4 | 1.06E+08 | A | T | 0.2206 | 0.0324 | 0.9647 | 9.85E-12 | rs144317085 | 14722 |
| FIt3L | 19 | 49979398 | A | G | 0.1179 | 0.022 | 0.0907 | 8.36E-08 | rs17272847 | 13222 |
| FIt3L | 10 | 1.01E+08 | T | C | 0.1483 | 0.0306 | 0.9513 | 1.26E-06 | rs185979634 | 12847 |
| FIt3L | 2 | 65602149 | T | C | -0.0746 | 0.0115 | 0.4771 | 8.76E-11 | rs1866051 | 14732 |
| FIt3L | 2 | 8442736 | C | G | 0.0554 | 0.0119 | 0.382 | 3.23E-06 | rs186975 | 14703 |
| FIt3L | 9 | 243950 | T | C | 0.1812 | 0.0386 | 0.026 | 2.68E-06 | rs192857133 | 14727 |
| FIt3L | 4 | 55408999 | T | C | 0.0833 | 0.016 | 0.8361 | 1.93E-07 | rs218265 | 14731 |
| FIt3L | 7 | 3914756 | A | G | -0.0749 | 0.0147 | 0.1924 | 3.48E-07 | rs28533776 | 14731 |
| FIt3L | 1 | 1.6E+08 | A | G | 0.0725 | 0.0129 | 0.4738 | 1.91E-08 | rs55633025 | 12404 |
| FIt3L | 9 | 10640144 | A | G | -0.1429 | 0.0299 | 0.9571 | 1.76E-06 | rs57191857 | 14734 |
| FIt3L | 4 | 20519864 | A | G | -0.0904 | 0.0192 | 0.8501 | 2.50E-06 | rs575588389 | 12129 |
| FIt3L | 3 | 1.71E+08 | A | G | -0.1569 | 0.0338 | 0.9624 | 3.45E-06 | rs61792394 | 14286 |
| FIt3L | 6 | 77907355 | T | C | -0.0559 | 0.0117 | 0.4699 | 1.77E-06 | rs6453962 | 14732 |
| FIt3L | 14 | 51156734 | A | G | 0.1302 | 0.0266 | 0.9447 | 9.84E-07 | rs72681653 | 14734 |
| FIt3L | 11 | 1.14E+08 | A | T | 0.0588 | 0.0125 | 0.3111 | 2.55E-06 | rs73000965 | 14731 |
| FIt3L | 10 | 1.05E+08 | T | C | 0.3529 | 0.076 | 0.9914 | 3.43E-06 | rs74722523 | 11437 |
| FIt3L | 3 | 1.28E+08 | T | G | -0.1061 | 0.0119 | 0.6093 | 4.84E-19 | rs7624160 | 14719 |
| FIt3L | 3 | 1.41E+08 | A | G | 0.0611 | 0.0129 | 0.5605 | 2.18E-06 | rs7625643 | 12404 |
| FIt3L | 13 | 28604007 | T | C | -1.1019 | 0.0524 | 0.9832 | 3.59E-98 | rs76428106 | 13799 |
| FIt3L | 5 | 1282319 | A | C | -0.1191 | 0.0128 | 0.3326 | 1.34E-20 | rs7726159 | 14733 |
| FIt3L | 20 | 30439298 | T | G | 0.0884 | 0.016 | 0.1818 | 3.29E-08 | rs78429945 | 14286 |
| FIt3L | 6 | 41985436 | T | C | 0.0643 | 0.0136 | 0.2577 | 2.27E-06 | rs9381118 | 14285 |
| FIt3L | 13 | 28455266 | A | T | 0.0821 | 0.0128 | 0.3602 | 1.42E-10 | rs9554186 | 14286 |
| hGDNF | 6 | 1.35E+08 | T | C | 0.1531 | 0.0333 | 0.0371 | 4.27E-06 | rs112594435 | 14721 |
| hGDNF | 5 | 37868088 | A | G | -0.3888 | 0.0198 | 0.0906 | 7.56E-86 | rs11740708 | 14710 |
| hGDNF | 12 | 98629454 | A | G | 0.2584 | 0.0539 | 0.0148 | 1.63E-06 | rs138691533 | 14731 |
| hGDNF | 2 | 2.09E+08 | A | T | 0.3366 | 0.0697 | 0.9898 | 1.37E-06 | rs144181640 | 13988 |
| hGDNF | 1 | 2E+08 | A | T | 0.1128 | 0.0219 | 0.8739 | 2.60E-07 | rs199503547 | 10894 |
| hGDNF | 9 | 5230371 | C | G | 0.0606 | 0.0129 | 0.3 | 2.63E-06 | rs2093446 | 14288 |
| hGDNF | 5 | 37918839 | A | T | -0.1683 | 0.0179 | 0.1296 | 5.34E-21 | rs2194229 | 14288 |
| hGDNF | 5 | 38094871 | A | T | 0.0829 | 0.0124 | 0.352 | 2.30E-11 | rs2453338 | 14734 |
| hGDNF | 17 | 1490782 | A | G | -0.0718 | 0.0154 | 0.7373 | 3.13E-06 | rs35740453 | 13224 |
| hGDNF | 8 | 1.08E+08 | T | G | -0.2941 | 0.0616 | 0.0124 | 1.80E-06 | rs540456481 | 10894 |
| hGDNF | 19 | 14805280 | T | C | 0.0816 | 0.014 | 0.3119 | 5.59E-09 | rs55850217 | 11755 |
| hGDNF | 13 | 28147359 | A | G | 0.0915 | 0.0196 | 0.903 | 3.04E-06 | rs57104839 | 14736 |
| hGDNF | 3 | 36072052 | T | C | -0.0981 | 0.0196 | 0.0997 | 5.58E-07 | rs62243355 | 14731 |
| hGDNF | 10 | 87694408 | A | C | -0.1194 | 0.0208 | 0.0859 | 9.45E-09 | rs77671113 | 14725 |
| hGDNF | 13 | 67293674 | T | C | 0.1918 | 0.042 | 0.0254 | 4.96E-06 | rs79953230 | 14719 |
| hGDNF | 17 | 14221290 | T | G | 0.2096 | 0.0453 | 0.0231 | 3.71E-06 | rs9303102 | 12935 |
| HGF | 8 | 72362254 | A | G | 0.3666 | 0.0747 | 0.014 | 9.22E-07 | rs113903858 | 11137 |
| HGF | 8 | 72362254 | A | G | 0.3666 | 0.0747 | 0.014 | 9.22E-07 | rs113903858 | 11137 |
| HGF | 6 | 622484 | A | G | 0.4297 | 0.0934 | 0.0078 | 4.21E-06 | rs115009171 | 10300 |
| HGF | 15 | 1.02E+08 | A | T | -0.0638 | 0.012 | 0.6021 | 1.06E-07 | rs11631440 | 14709 |
| HGF | 1 | 83864840 | T | C | -0.405 | 0.0832 | 0.9907 | 1.13E-06 | rs138848271 | 10793 |
| HGF | 18 | 76506666 | A | T | 0.1566 | 0.0327 | 0.9642 | 1.68E-06 | rs150498795 | 14732 |
| HGF | 17 | 45959996 | T | C | -0.1954 | 0.039 | 0.9572 | 5.44E-07 | rs1986719 | 14718 |
| HGF | 20 | 6103587 | T | C | -0.0698 | 0.0151 | 0.7983 | 3.79E-06 | rs2281532 | 14286 |
| HGF | 3 | 1.28E+08 | A | G | 0.2047 | 0.0391 | 0.0276 | 1.65E-07 | rs2737756 | 14718 |
| HGF | 1 | 38949303 | T | C | 0.0992 | 0.0217 | 0.8959 | 4.84E-06 | rs34219417 | 14286 |
| HGF | 17 | 7014969 | T | C | -0.0992 | 0.0217 | 0.0942 | 4.84E-06 | rs4290523 | 12846 |
| HGF | 9 | 1.08E+08 | T | C | 0.0769 | 0.0164 | 0.7969 | 2.75E-06 | rs5007364 | 11760 |
| HGF | 14 | 65654346 | T | C | 0.1572 | 0.0311 | 0.9611 | 4.31E-07 | rs57193877 | 14725 |
| HGF | 7 | 81359051 | T | C | -0.1943 | 0.0242 | 0.0608 | 9.83E-16 | rs5745687 | 14733 |
| HGF | 7 | 81358266 | C | G | -0.1545 | 0.0332 | 0.964 | 3.26E-06 | rs5745692 | 14716 |
| HGF | 4 | 3452345 | A | G | 0.1219 | 0.0131 | 0.328 | 1.34E-20 | rs59950280 | 13222 |
| HGF | 2 | 1.09E+08 | A | G | 0.0564 | 0.0114 | 0.4434 | 7.52E-07 | rs6709159 | 14734 |
| HGF | 10 | 30042437 | T | C | -0.2429 | 0.0489 | 0.9809 | 6.79E-07 | rs74130123 | 14734 |
| HGF | 3 | 1.72E+08 | A | G | -0.0856 | 0.0179 | 0.1397 | 1.73E-06 | rs7620568 | 12846 |
| HGF | 19 | 58888317 | A | G | 0.0592 | 0.0127 | 0.5179 | 3.14E-06 | rs8106345 | 12404 |
| HGF | 19 | 42769693 | A | G | -0.1182 | 0.0257 | 0.9405 | 4.24E-06 | rs851612 | 14286 |
| IFN-gamma | 11 | 1.11E+08 | T | C | 0.4666 | 0.0897 | 0.0122 | 1.97E-07 | rs140562194 | 7396 |
| IFN-gamma | 13 | 90334795 | C | G | 0.1935 | 0.0384 | 0.9683 | 4.68E-07 | rs146527076 | 11781 |
| IFN-gamma | 20 | 46408844 | T | C | 0.0671 | 0.0137 | 0.5585 | 9.69E-07 | rs4810686 | 11344 |
| IFN-gamma | 16 | 33979312 | T | G | 0.2684 | 0.0573 | 0.0324 | 2.81E-06 | rs531747046 | 6257 |
| IFN-gamma | 10 | 1.31E+08 | A | G | 0.136 | 0.0283 | 0.9427 | 1.54E-06 | rs61863416 | 11774 |
| IFN-gamma | 21 | 37335521 | A | G | 0.2184 | 0.0403 | 0.0412 | 5.98E-08 | rs62230781 | 11345 |
| IFN-gamma | 3 | 2921515 | A | C | 0.0648 | 0.014 | 0.4253 | 3.68E-06 | rs6442763 | 11345 |
| IFN-gamma | 2 | 19234534 | T | G | 0.1461 | 0.0307 | 0.9482 | 1.95E-06 | rs67101138 | 11793 |
| IFN-gamma | 17 | 26694861 | A | G | -0.0635 | 0.0127 | 0.4705 | 5.73E-07 | rs704 | 11793 |
| IFN-gamma | 13 | 27473585 | T | C | 0.3485 | 0.0708 | 0.9882 | 8.55E-07 | rs74903999 | 10558 |
| IL-10 | 5 | 19244332 | T | C | -0.0753 | 0.0149 | 0.8011 | 4.33E-07 | rs10067443 | 14296 |
| IL-10 | 1 | 2.07E+08 | A | G | -0.0996 | 0.0151 | 0.2042 | 4.22E-11 | rs12123181 | 14296 |
| IL-10 | 2 | 1.26E+08 | T | G | 0.1937 | 0.0418 | 0.0455 | 3.59E-06 | rs138203589 | 14730 |
| IL-10 | 7 | 590757 | C | G | 0.1112 | 0.0239 | 0.1005 | 3.28E-06 | rs148332061 | 13232 |
| IL-10 | 6 | 1.23E+08 | A | G | 0.3666 | 0.0761 | 0.9898 | 1.45E-06 | rs150408090 | 12313 |
| IL-10 | 6 | 18825879 | T | C | 0.2951 | 0.0626 | 0.014 | 2.43E-06 | rs17654493 | 12313 |
| IL-10 | 2 | 2.02E+08 | T | C | -0.2351 | 0.0486 | 0.0174 | 1.32E-06 | rs188535859 | 14741 |
| IL-10 | 11 | 1.18E+08 | A | G | -0.1597 | 0.0154 | 0.823 | 3.39E-25 | rs3135932 | 14744 |
| IL-10 | 2 | 1.74E+08 | A | G | 0.0771 | 0.0162 | 0.8403 | 1.94E-06 | rs3769274 | 14742 |
| IL-10 | 6 | 7880027 | T | C | 0.0555 | 0.0119 | 0.6126 | 3.10E-06 | rs385697 | 14727 |
| IL-10 | 6 | 1.69E+08 | A | G | -0.0574 | 0.0125 | 0.6162 | 4.39E-06 | rs389239 | 14296 |
| IL-10 | 3 | 8954959 | A | G | -0.1293 | 0.0218 | 0.9146 | 3.01E-09 | rs398518 | 14296 |
| IL-10 | 1 | 87832695 | A | G | 0.0565 | 0.0121 | 0.3683 | 3.02E-06 | rs4655953 | 14722 |
| IL-10 | 2 | 87296860 | A | C | 0.1647 | 0.0338 | 0.0539 | 1.10E-06 | rs549056348 | 11768 |
| IL-10 | 8 | 61861039 | T | C | 0.0681 | 0.0143 | 0.2791 | 1.91E-06 | rs66624860 | 12799 |
| IL-10 | 6 | 32453163 | T | C | -0.1528 | 0.0196 | 0.8893 | 6.39E-15 | rs67037615 | 14296 |
| IL-10 | 4 | 1.85E+08 | A | G | 0.0981 | 0.0211 | 0.0999 | 3.33E-06 | rs72701782 | 14296 |
| IL-10 | 9 | 18625666 | A | T | -0.2845 | 0.0618 | 0.0122 | 4.15E-06 | rs79438518 | 13809 |
| IL-10 | 6 | 31325886 | C | G | -0.065 | 0.0129 | 0.6959 | 4.69E-07 | rs9266257 | 14728 |
| IL-10RA | 8 | 9104264 | T | G | 0.064 | 0.0135 | 0.4945 | 2.13E-06 | rs10091841 | 11345 |
| IL-10RA | 15 | 93461371 | C | G | 0.0623 | 0.0131 | 0.4267 | 1.98E-06 | rs1406714 | 11783 |
| IL-10RA | 16 | 5247086 | T | C | -0.3868 | 0.0841 | 0.0107 | 4.24E-06 | rs143313574 | 9956 |
| IL-10RA | 14 | 55005535 | A | G | 0.1676 | 0.0343 | 0.0481 | 1.03E-06 | rs17832137 | 10289 |
| IL-10RA | 6 | 78703747 | A | C | 0.4057 | 0.0865 | 0.9896 | 2.73E-06 | rs180893431 | 9340 |
| IL-10RA | 5 | 1.54E+08 | T | G | 0.0623 | 0.0131 | 0.5891 | 1.98E-06 | rs286605 | 11793 |
| IL-10RA | 11 | 1.18E+08 | A | G | -0.0844 | 0.0168 | 0.8171 | 5.07E-07 | rs3135932 | 11793 |
| IL-10RA | 3 | 1.59E+08 | T | G | 0.082 | 0.0179 | 0.1526 | 4.63E-06 | rs41272625 | 11784 |
| IL-10RA | 1 | 56566352 | A | G | -0.1247 | 0.0252 | 0.0839 | 7.48E-07 | rs6670799 | 11345 |
| IL-10RA | 14 | 66407688 | A | G | 0.3915 | 0.0823 | 0.9853 | 1.97E-06 | rs74719858 | 10858 |
| IL-10RA | 10 | 18335103 | T | C | 0.2347 | 0.0511 | 0.9822 | 4.37E-06 | rs80137559 | 11791 |
| IL-10RB | 7 | 25740142 | A | G | -0.0843 | 0.0181 | 0.1348 | 3.20E-06 | rs111850948 | 14286 |
| IL-10RB | 1 | 2.28E+08 | T | C | 0.1926 | 0.0411 | 0.026 | 2.78E-06 | rs112798338 | 14286 |
| IL-10RB | 1 | 2.13E+08 | T | C | 0.1449 | 0.0311 | 0.0363 | 3.17E-06 | rs114288486 | 14730 |
| IL-10RB | 3 | 4275803 | T | C | -0.0548 | 0.0119 | 0.5866 | 4.12E-06 | rs1153572 | 14286 |
| IL-10RB | 2 | 1.77E+08 | T | C | 0.1988 | 0.0402 | 0.0212 | 7.60E-07 | rs116315732 | 14734 |
| IL-10RB | 19 | 49553051 | C | G | -0.4295 | 0.0907 | 0.9904 | 2.19E-06 | rs117883662 | 8938 |
| IL-10RB | 1 | 1.8E+08 | A | G | 0.208 | 0.031 | 0.0438 | 1.95E-11 | rs142421172 | 12840 |
| IL-10RB | 20 | 39437913 | A | G | 0.2066 | 0.0444 | 0.0323 | 3.27E-06 | rs149156511 | 14286 |
| IL-10RB | 12 | 58388232 | C | G | -0.0601 | 0.0124 | 0.6484 | 1.25E-06 | rs1605333 | 13237 |
| IL-10RB | 10 | 71477178 | A | G | -0.2327 | 0.0505 | 0.9827 | 4.07E-06 | rs192325200 | 13867 |
| IL-10RB | 21 | 35160180 | T | C | -0.0815 | 0.014 | 0.2114 | 5.83E-09 | rs2834261 | 14720 |
| IL-10RB | 6 | 41174158 | A | G | 0.0532 | 0.0113 | 0.5488 | 2.50E-06 | rs34361138 | 14732 |
| IL-10RB | 16 | 13209083 | T | C | 0.1783 | 0.0348 | 0.9674 | 3.00E-07 | rs55780441 | 14734 |
| IL-10RB | 6 | 55775469 | T | G | 0.0652 | 0.0142 | 0.3356 | 4.40E-06 | rs566450266 | 11759 |
| IL-10RB | 10 | 1.04E+08 | T | C | 0.0743 | 0.0161 | 0.8362 | 3.93E-06 | rs6584485 | 14286 |
| IL-10RB | 2 | 43150924 | A | G | 0.0637 | 0.0135 | 0.2684 | 2.38E-06 | rs7574167 | 12832 |
| IL-10RB | 9 | 1.36E+08 | T | C | 0.0861 | 0.0169 | 0.1723 | 3.49E-07 | rs7855466 | 11783 |
| IL-10RB | 21 | 34660980 | A | G | -0.3997 | 0.0116 | 0.4732 | 1.00E-200 | rs8178528 | 14286 |
| IL-10RB | 4 | 1.2E+08 | A | G | -0.0618 | 0.0134 | 0.2721 | 3.99E-06 | rs9685374 | 14286 |
| IL-12B | 11 | 47406592 | T | C | 0.0588 | 0.0116 | 0.5207 | 4.00E-07 | rs11039216 | 14730 |
| IL-12B | 3 | 5026008 | A | G | -0.1009 | 0.0151 | 0.8154 | 2.36E-11 | rs11130215 | 14732 |
| IL-12B | 8 | 4597842 | T | C | 0.219 | 0.0472 | 0.9787 | 3.49E-06 | rs117888068 | 11783 |
| IL-12B | 14 | 1.03E+08 | C | G | -0.1297 | 0.0127 | 0.6875 | 1.74E-24 | rs12588969 | 14287 |
| IL-12B | 10 | 94525783 | C | G | 0.0709 | 0.0149 | 0.8109 | 1.95E-06 | rs12766137 | 14726 |
| IL-12B | 12 | 17314071 | A | G | -0.197 | 0.0363 | 0.0298 | 5.73E-08 | rs1398590 | 14726 |
| IL-12B | 5 | 1.59E+08 | T | G | 0.3161 | 0.056 | 0.9869 | 1.66E-08 | rs144850787 | 14287 |
| IL-12B | 3 | 1.71E+08 | T | C | 0.1088 | 0.0228 | 0.0889 | 1.82E-06 | rs148549227 | 12403 |
| IL-12B | 2 | 1.12E+08 | T | C | 0.0984 | 0.0194 | 0.1662 | 3.93E-07 | rs149993822 | 14287 |
| IL-12B | 5 | 1.59E+08 | A | G | 0.1694 | 0.0325 | 0.9592 | 1.87E-07 | rs17056751 | 14735 |
| IL-12B | 5 | 1.59E+08 | T | C | -0.0857 | 0.0131 | 0.7246 | 6.07E-11 | rs17722688 | 14729 |
| IL-12B | 14 | 68760141 | T | C | 0.0798 | 0.0128 | 0.7151 | 4.54E-10 | rs1950897 | 14735 |
| IL-12B | 6 | 31154493 | A | G | 0.1155 | 0.014 | 0.2275 | 1.58E-16 | rs3130510 | 14735 |
| IL-12B | 12 | 1.12E+08 | T | C | 0.1332 | 0.0131 | 0.4766 | 2.76E-24 | rs3184504 | 11785 |
| IL-12B | 4 | 1.91E+08 | T | C | 0.207 | 0.0442 | 0.0363 | 2.82E-06 | rs5014495 | 10208 |
| IL-12B | 12 | 1.21E+08 | C | G | 0.0721 | 0.0122 | 0.3473 | 3.42E-09 | rs516978 | 14725 |
| IL-12B | 11 | 96026074 | T | C | -0.0672 | 0.0129 | 0.2822 | 1.90E-07 | rs539598 | 14731 |
| IL-12B | 5 | 1.59E+08 | A | C | -0.4957 | 0.0122 | 0.3044 | 1.00E-200 | rs6556416 | 14287 |
| IL-12B | 2 | 64889908 | T | C | 0.0628 | 0.0134 | 0.7333 | 2.78E-06 | rs6750065 | 14286 |
| IL-12B | 7 | 6408796 | T | G | -0.0622 | 0.0119 | 0.5425 | 1.72E-07 | rs6968201 | 14287 |
| IL-12B | 12 | 56435504 | C | G | 0.0785 | 0.0133 | 0.323 | 3.59E-09 | rs705705 | 12841 |
| IL-12B | 11 | 1.16E+08 | A | G | -0.204 | 0.0435 | 0.0216 | 2.74E-06 | rs72993671 | 14246 |
| IL-12B | 22 | 37881211 | A | G | -0.1477 | 0.0246 | 0.0654 | 1.92E-09 | rs73416208 | 14729 |
| IL-12B | 13 | 28604007 | T | C | -0.3406 | 0.0535 | 0.9832 | 1.94E-10 | rs76428106 | 13800 |
| IL-12B | 10 | 73114509 | T | C | -0.0695 | 0.0139 | 0.2389 | 5.73E-07 | rs780679 | 14729 |
| IL-12B | 15 | 80260554 | A | G | -0.0743 | 0.0132 | 0.2595 | 1.81E-08 | rs8026767 | 14734 |
| IL-12B | 3 | 1.88E+08 | A | C | 0.2861 | 0.013 | 0.3539 | 2.43E-107 | rs9815073 | 14287 |
| IL-13 | 3 | 1.43E+08 | A | G | 0.064 | 0.0132 | 0.3961 | 1.24E-06 | rs10446322 | 11777 |
| IL-13 | 11 | 1.23E+08 | C | G | -0.0702 | 0.015 | 0.6744 | 2.87E-06 | rs10893042 | 11344 |
| IL-13 | 1 | 1.63E+08 | A | G | 0.0692 | 0.0136 | 0.3447 | 3.61E-07 | rs10917637 | 11772 |
| IL-13 | 10 | 3623961 | A | G | -0.1845 | 0.0397 | 0.0751 | 3.36E-06 | rs11251952 | 11344 |
| IL-13 | 10 | 53942324 | T | G | 0.0734 | 0.0155 | 0.3902 | 2.19E-06 | rs12146346 | 8388 |
| IL-13 | 4 | 40938582 | A | G | 0.0739 | 0.0132 | 0.4958 | 2.16E-08 | rs13119159 | 11344 |
| IL-13 | 13 | 41810092 | T | C | 0.2093 | 0.0446 | 0.9682 | 2.69E-06 | rs141726773 | 11344 |
| IL-13 | 7 | 61999529 | A | G | 0.1832 | 0.039 | 0.9229 | 2.63E-06 | rs149979178 | 6901 |
| IL-13 | 5 | 9974643 | C | G | -0.2968 | 0.0628 | 0.9858 | 2.29E-06 | rs183684201 | 11782 |
| IL-13 | 15 | 57830694 | T | C | -0.0934 | 0.0183 | 0.8535 | 3.33E-07 | rs55924857 | 11792 |
| IL-13 | 16 | 79977921 | A | G | 0.2122 | 0.0439 | 0.0274 | 1.34E-06 | rs59235092 | 11784 |
| IL-13 | 7 | 1.54E+08 | A | G | 0.169 | 0.0357 | 0.0397 | 2.20E-06 | rs76219628 | 11344 |
| IL-13 | 3 | 1.24E+08 | A | G | -0.0656 | 0.0141 | 0.3094 | 3.28E-06 | rs9881117 | 11791 |
| IL-15RA | 15 | 94729209 | A | T | -0.0849 | 0.017 | 0.4367 | 5.91E-07 | rs117046642 | 7943 |
| IL-15RA | 8 | 27696981 | A | C | -0.3132 | 0.0684 | 0.0119 | 4.67E-06 | rs117428573 | 10857 |
| IL-15RA | 21 | 15303777 | A | G | 0.3794 | 0.0799 | 0.0158 | 2.05E-06 | rs117484888 | 7423 |
| IL-15RA | 10 | 5815194 | A | T | -0.0867 | 0.0174 | 0.8028 | 6.27E-07 | rs12570881 | 11344 |
| IL-15RA | 11 | 37594094 | A | G | -0.2278 | 0.0492 | 0.9801 | 3.66E-06 | rs145793996 | 11779 |
| IL-15RA | 20 | 2629394 | A | T | 0.1159 | 0.0241 | 0.8743 | 1.52E-06 | rs192538756 | 9461 |
| IL-15RA | 10 | 6002368 | T | G | 0.3692 | 0.0127 | 0.5146 | 8.39E-186 | rs2228059 | 11344 |
| IL-15RA | 21 | 39896592 | A | G | 0.0796 | 0.0169 | 0.8384 | 2.48E-06 | rs2836464 | 11791 |
| IL-15RA | 18 | 12175379 | T | C | 0.14 | 0.0294 | 0.0658 | 1.92E-06 | rs4459616 | 9899 |
| IL-15RA | 4 | 20169693 | A | T | 0.0919 | 0.0193 | 0.8082 | 1.92E-06 | rs56240706 | 8861 |
| IL-15RA | 4 | 1.41E+08 | C | G | 0.0778 | 0.0162 | 0.8203 | 1.57E-06 | rs6845172 | 11772 |
| IL-15RA | 12 | 1.12E+08 | T | C | -0.0748 | 0.0133 | 0.5243 | 1.87E-08 | rs7137828 | 11344 |
| IL-15RA | 10 | 6009462 | T | G | -0.1967 | 0.0135 | 0.3975 | 4.34E-48 | rs7898286 | 11344 |
| IL-15RA | 6 | 32560501 | A | G | 0.0749 | 0.0153 | 0.7077 | 9.81E-07 | rs9270551 | 11344 |
| IL-17A | 3 | 63484616 | T | C | 0.1688 | 0.0349 | 0.0418 | 1.32E-06 | rs115193412 | 11778 |
| IL-17A | 16 | 8011846 | T | C | -0.0726 | 0.0153 | 0.7391 | 2.08E-06 | rs12599620 | 11336 |
| IL-17A | 8 | 70106392 | C | G | -0.0893 | 0.017 | 0.1776 | 1.50E-07 | rs1462017 | 11774 |
| IL-17A | 3 | 1.9E+08 | A | G | -0.0585 | 0.0128 | 0.5609 | 4.87E-06 | rs17448036 | 11783 |
| IL-17A | 17 | 2272057 | T | C | 0.2309 | 0.0486 | 0.0251 | 2.02E-06 | rs2429911 | 11294 |
| IL-17A | 7 | 6380998 | A | G | -0.068 | 0.0148 | 0.2897 | 4.34E-06 | rs28852438 | 11336 |
| IL-17A | 1 | 96622380 | A | C | -0.0802 | 0.0164 | 0.7781 | 1.01E-06 | rs33998516 | 11336 |
| IL-17A | 10 | 67955765 | T | G | 0.2446 | 0.0517 | 0.9819 | 2.23E-06 | rs34557923 | 10913 |
| IL-17A | 6 | 32628224 | A | T | 0.0923 | 0.0189 | 0.221 | 1.04E-06 | rs41270871 | 8742 |
| IL-17A | 9 | 91976386 | A | G | 0.0908 | 0.0192 | 0.1411 | 2.25E-06 | rs4877075 | 11336 |
| IL-17A | 2 | 13468626 | T | C | -0.2432 | 0.0525 | 0.021 | 3.61E-06 | rs75788494 | 10574 |
| IL-17A | 9 | 27215497 | T | C | -0.1279 | 0.0272 | 0.9323 | 2.57E-06 | rs77529534 | 11775 |
| IL-17A | 12 | 1.18E+08 | T | G | 0.1696 | 0.0371 | 0.9648 | 4.84E-06 | rs77595989 | 11767 |
| IL-17A | 9 | 31560030 | C | G | -0.1467 | 0.0295 | 0.0555 | 6.60E-07 | rs79982350 | 11336 |
| IL-17C | 4 | 24758319 | A | G | -0.0649 | 0.0141 | 0.5847 | 4.17E-06 | rs1072074 | 11785 |
| IL-17C | 13 | 44396409 | A | G | -0.0816 | 0.0171 | 0.8025 | 1.82E-06 | rs10870773 | 11345 |
| IL-17C | 1 | 1.77E+08 | A | T | -0.3259 | 0.0548 | 0.9804 | 2.73E-09 | rs114882743 | 11345 |
| IL-17C | 4 | 1.52E+08 | T | C | -0.1219 | 0.0262 | 0.9068 | 3.28E-06 | rs11931996 | 11345 |
| IL-17C | 1 | 2.43E+08 | T | G | 0.1508 | 0.0327 | 0.0512 | 4.00E-06 | rs12092228 | 9892 |
| IL-17C | 16 | 76626873 | C | G | -0.09 | 0.0195 | 0.1382 | 3.92E-06 | rs12445031 | 11339 |
| IL-17C | 13 | 96638651 | T | C | -0.0857 | 0.018 | 0.1597 | 1.93E-06 | rs12863903 | 11345 |
| IL-17C | 14 | 1.02E+08 | T | C | 0.1091 | 0.0236 | 0.0906 | 3.78E-06 | rs12895022 | 11774 |
| IL-17C | 2 | 2.4E+08 | A | C | -0.2749 | 0.0524 | 0.9809 | 1.55E-07 | rs149756404 | 11302 |
| IL-17C | 16 | 88684495 | T | G | -0.105 | 0.0136 | 0.3783 | 1.16E-14 | rs17700884 | 11775 |
| IL-17C | 7 | 21240100 | T | C | 0.0633 | 0.0132 | 0.3948 | 1.62E-06 | rs1920106 | 11792 |
| IL-17C | 6 | 66547346 | C | G | 0.0624 | 0.0135 | 0.6437 | 3.80E-06 | rs2814092 | 11792 |
| IL-17C | 16 | 8393906 | C | G | 0.1077 | 0.0227 | 0.1032 | 2.09E-06 | rs39779 | 11345 |
| IL-17C | 5 | 19253845 | A | G | -0.0793 | 0.0167 | 0.6301 | 2.05E-06 | rs4593271 | 7943 |
| IL-17C | 12 | 52750252 | A | G | 0.1926 | 0.0401 | 0.0286 | 1.56E-06 | rs554604740 | 11149 |
| IL-17C | 5 | 1.07E+08 | A | G | -0.0882 | 0.0177 | 0.8432 | 6.26E-07 | rs59356989 | 11790 |
| IL-17C | 5 | 1.75E+08 | T | C | -0.1673 | 0.0366 | 0.0423 | 4.85E-06 | rs72815428 | 11775 |
| IL-17C | 5 | 1.74E+08 | T | C | -0.0951 | 0.0201 | 0.1233 | 2.23E-06 | rs75246422 | 11793 |
| IL-17C | 2 | 1.37E+08 | T | G | -0.2174 | 0.0469 | 0.9707 | 3.56E-06 | rs77654242 | 10294 |
| IL-17C | 14 | 92763907 | C | G | -0.1162 | 0.0253 | 0.0991 | 4.37E-06 | rs8015296 | 9461 |
| IL-18 | 9 | 20069041 | A | C | -0.1954 | 0.0425 | 0.0236 | 4.27E-06 | rs112338272 | 14721 |
| IL-18 | 16 | 84730435 | C | G | -0.278 | 0.0581 | 0.9792 | 1.71E-06 | rs113552426 | 11249 |
| IL-18 | 11 | 268430 | C | G | -0.0629 | 0.0137 | 0.7543 | 4.41E-06 | rs11605127 | 14730 |
| IL-18 | 6 | 1.63E+08 | A | T | 0.0676 | 0.0138 | 0.2767 | 9.65E-07 | rs12201187 | 13245 |
| IL-18 | 4 | 1.22E+08 | A | G | 0.072 | 0.0146 | 0.8077 | 8.16E-07 | rs12648080 | 14743 |
| IL-18 | 8 | 1.45E+08 | C | G | -0.0935 | 0.0157 | 0.8179 | 2.59E-09 | rs12681624 | 13657 |
| IL-18 | 2 | 1.27E+08 | T | C | -0.0702 | 0.0153 | 0.7336 | 4.47E-06 | rs12711729 | 11345 |
| IL-18 | 3 | 1.15E+08 | A | T | -0.3049 | 0.0604 | 0.989 | 4.46E-07 | rs139760701 | 13809 |
| IL-18 | 20 | 51289816 | A | T | -0.0567 | 0.0124 | 0.371 | 4.82E-06 | rs1403742 | 14296 |
| IL-18 | 16 | 54161464 | A | C | 0.2532 | 0.0525 | 0.9857 | 1.42E-06 | rs148504635 | 14737 |
| IL-18 | 5 | 68682536 | A | C | -0.16 | 0.0267 | 0.9351 | 2.07E-09 | rs17229943 | 14744 |
| IL-18 | 19 | 5344989 | T | C | 0.0581 | 0.0126 | 0.3565 | 4.01E-06 | rs2108621 | 14296 |
| IL-18 | 8 | 1.26E+08 | T | C | -0.0759 | 0.0156 | 0.3451 | 1.14E-06 | rs2954016 | 13421 |
| IL-18 | 11 | 1.12E+08 | T | C | 0.1374 | 0.0291 | 0.0465 | 2.34E-06 | rs35495214 | 14735 |
| IL-18 | 2 | 32489851 | T | C | -0.1857 | 0.0123 | 0.3734 | 1.68E-51 | rs385076 | 14296 |
| IL-18 | 6 | 38300402 | A | G | 0.2157 | 0.0461 | 0.0204 | 2.88E-06 | rs4140444 | 14741 |
| IL-18 | 5 | 70375337 | T | G | 0.1154 | 0.0252 | 0.8692 | 4.66E-06 | rs567093451 | 10894 |
| IL-18 | 7 | 1.56E+08 | T | C | -0.163 | 0.0301 | 0.0477 | 6.12E-08 | rs56836641 | 14743 |
| IL-18 | 11 | 1.12E+08 | A | C | 0.2039 | 0.0135 | 0.7618 | 1.53E-51 | rs5744249 | 14742 |
| IL-18 | 11 | 86686181 | C | G | -0.1248 | 0.0259 | 0.0665 | 1.45E-06 | rs57954386 | 12201 |
| IL-18 | 11 | 1.05E+08 | T | C | 0.1154 | 0.0225 | 0.9237 | 2.91E-07 | rs60235102 | 14722 |
| IL-18 | 7 | 11104167 | T | G | -0.082 | 0.0165 | 0.8341 | 6.71E-07 | rs67424072 | 14296 |
| IL-18 | 18 | 28763724 | A | G | -0.2314 | 0.0472 | 0.9814 | 9.46E-07 | rs77994606 | 14743 |
| IL-18R1 | 2 | 1.03E+08 | T | C | -0.3681 | 0.0115 | 0.3754 | 1.00E-200 | rs10192157 | 14737 |
| IL-18R1 | 2 | 1.03E+08 | T | C | 0.1489 | 0.0115 | 0.5422 | 2.42E-38 | rs1115282 | 14743 |
| IL-18R1 | 2 | 1.02E+08 | T | C | 0.184 | 0.0305 | 0.0508 | 1.61E-09 | rs114429030 | 14295 |
| IL-18R1 | 22 | 34904594 | A | G | 0.2387 | 0.0512 | 0.9848 | 3.13E-06 | rs116935210 | 14733 |
| IL-18R1 | 7 | 29690677 | A | G | -0.3006 | 0.0649 | 0.0131 | 3.63E-06 | rs117145942 | 13164 |
| IL-18R1 | 10 | 61379709 | A | G | -0.1505 | 0.0313 | 0.0389 | 1.52E-06 | rs117195704 | 14730 |
| IL-18R1 | 16 | 87926882 | A | C | -0.1483 | 0.0317 | 0.0536 | 2.89E-06 | rs117599131 | 13231 |
| IL-18R1 | 14 | 20930038 | A | C | 0.0657 | 0.0142 | 0.7591 | 3.71E-06 | rs1760941 | 14295 |
| IL-18R1 | 14 | 94844947 | T | C | 0.2643 | 0.0445 | 0.0204 | 2.86E-09 | rs28929474 | 14743 |
| IL-18R1 | 20 | 51193977 | A | G | 0.073 | 0.0149 | 0.8157 | 9.62E-07 | rs34174304 | 14725 |
| IL-18R1 | 3 | 58414784 | A | G | -0.1646 | 0.0342 | 0.0317 | 1.49E-06 | rs35021151 | 14735 |
| IL-18R1 | 8 | 28819417 | T | G | 0.1906 | 0.0387 | 0.9625 | 8.43E-07 | rs536613714 | 12193 |
| IL-18R1 | 3 | 79589418 | A | G | -0.2015 | 0.042 | 0.9745 | 1.61E-06 | rs62257659 | 14727 |
| IL-18R1 | 7 | 1.56E+08 | T | C | -0.1045 | 0.022 | 0.0762 | 2.03E-06 | rs73176827 | 14733 |
| IL-18R1 | 2 | 1.03E+08 | T | G | 0.1911 | 0.0143 | 0.5584 | 9.86E-41 | rs74180212 | 10893 |
| IL-18R1 | 1 | 15404794 | A | G | 0.0921 | 0.0201 | 0.1213 | 4.60E-06 | rs7512350 | 11771 |
| IL-18R1 | 3 | 10580281 | A | C | 0.0935 | 0.02 | 0.8764 | 2.94E-06 | rs7642750 | 11788 |
| IL-18R1 | 6 | 801754 | A | C | 0.1039 | 0.0222 | 0.0787 | 2.87E-06 | rs78035061 | 14295 |
| IL-18R1 | 3 | 1.47E+08 | A | G | -0.0956 | 0.0196 | 0.8928 | 1.07E-06 | rs78199195 | 14295 |
| IL-18R1 | 17 | 64305051 | A | G | -0.3382 | 0.0378 | 0.9715 | 3.65E-19 | rs78357146 | 14743 |
| IL-18R1 | 22 | 19662319 | T | C | -0.2754 | 0.056 | 0.9865 | 8.75E-07 | rs79097546 | 14256 |
| IL-18R1 | 7 | 47517191 | A | G | 0.1408 | 0.0299 | 0.0468 | 2.49E-06 | rs80317525 | 14730 |
| IL-1 alpha | 7 | 89060931 | T | G | 0.131 | 0.0286 | 0.0604 | 4.64E-06 | rs10233998 | 11785 |
| IL-1 alpha | 1 | 1.17E+08 | A | G | 0.2658 | 0.0565 | 0.0186 | 2.55E-06 | rs115494443 | 10853 |
| IL-1 alpha | 2 | 1.71E+08 | T | C | 0.2215 | 0.0469 | 0.0311 | 2.33E-06 | rs115602471 | 11340 |
| IL-1 alpha | 17 | 32073614 | T | C | 0.2176 | 0.0439 | 0.9755 | 7.17E-07 | rs117537822 | 11788 |
| IL-1 alpha | 6 | 32586222 | A | G | -0.3263 | 0.0266 | 0.0799 | 1.36E-34 | rs11759846 | 11788 |
| IL-1 alpha | 18 | 308555 | A | G | 0.1116 | 0.0221 | 0.9015 | 4.42E-07 | rs11878153 | 11787 |
| IL-1 alpha | 1 | 1.05E+08 | T | C | 0.0783 | 0.0171 | 0.174 | 4.67E-06 | rs12047801 | 11785 |
| IL-1 alpha | 6 | 32656094 | T | C | -0.3251 | 0.0554 | 0.0358 | 4.40E-09 | rs13195317 | 11301 |
| IL-1 alpha | 8 | 89744017 | A | T | 0.2438 | 0.0524 | 0.9824 | 3.28E-06 | rs16886503 | 11786 |
| IL-1 alpha | 12 | 1.15E+08 | T | C | 0.062 | 0.013 | 0.5326 | 1.85E-06 | rs4767214 | 11772 |
| IL-1 alpha | 11 | 1.19E+08 | A | G | 0.0754 | 0.0142 | 0.5264 | 1.10E-07 | rs523604 | 10290 |
| IL-1 alpha | 6 | 32606006 | A | T | -0.3452 | 0.0647 | 0.0283 | 9.53E-08 | rs547199103 | 7943 |
| IL-1 alpha | 17 | 53509585 | A | G | -0.0856 | 0.0187 | 0.8232 | 4.70E-06 | rs61098036 | 9891 |
| IL-1 alpha | 12 | 1.04E+08 | A | T | -0.0982 | 0.0207 | 0.1141 | 2.10E-06 | rs7138836 | 11775 |
| IL-2 | 10 | 67202938 | T | C | -0.1222 | 0.0259 | 0.9188 | 2.38E-06 | rs10996419 | 11784 |
| IL-2 | 3 | 12396234 | T | C | 0.2095 | 0.0419 | 0.0287 | 5.73E-07 | rs142641125 | 11789 |
| IL-2 | 14 | 55563894 | C | G | -0.2699 | 0.0576 | 0.9727 | 2.79E-06 | rs144129701 | 11341 |
| IL-2 | 19 | 55502846 | C | G | -0.0849 | 0.017 | 0.2053 | 5.91E-07 | rs1671226 | 11341 |
| IL-2 | 9 | 88775115 | A | C | -0.1468 | 0.0309 | 0.9396 | 2.03E-06 | rs201229721 | 9257 |
| IL-2 | 17 | 103299 | A | C | -0.0723 | 0.0149 | 0.6969 | 1.22E-06 | rs28515912 | 10723 |
| IL-2 | 16 | 77431739 | T | C | -0.0728 | 0.0153 | 0.7384 | 1.95E-06 | rs36006983 | 11341 |
| IL-2 | 5 | 35188802 | T | C | -0.1216 | 0.026 | 0.9334 | 2.91E-06 | rs4703508 | 11789 |
| IL-2 | 4 | 1.38E+08 | T | G | -0.0646 | 0.0139 | 0.6861 | 3.36E-06 | rs6535212 | 11784 |
| IL-2 | 10 | 2293803 | T | G | -0.1999 | 0.0431 | 0.0244 | 3.52E-06 | rs74852566 | 11789 |
| IL-2 | 3 | 1.37E+08 | T | C | 0.1737 | 0.038 | 0.039 | 4.85E-06 | rs75739108 | 11341 |
| IL-2 | 3 | 1.26E+08 | A | T | 0.0854 | 0.0174 | 0.8293 | 9.20E-07 | rs7638939 | 11769 |
| IL-2 | 13 | 85868678 | A | G | 0.1922 | 0.0414 | 0.0318 | 3.44E-06 | rs77832761 | 11785 |
| IL-2 | 3 | 64865615 | T | C | -0.1048 | 0.0217 | 0.8961 | 1.37E-06 | rs77853517 | 11787 |
| IL-2 | 2 | 1.93E+08 | A | G | 0.2016 | 0.0424 | 0.9733 | 1.99E-06 | rs79419497 | 11789 |
| IL-2 | 4 | 34713434 | T | G | 0.1176 | 0.0255 | 0.9036 | 3.99E-06 | rs79925007 | 11341 |
| IL-20 | 2 | 52215130 | A | G | -0.2134 | 0.046 | 0.9756 | 3.50E-06 | rs114209791 | 11784 |
| IL-20 | 1 | 41433965 | T | C | -0.2372 | 0.0487 | 0.0189 | 1.11E-06 | rs114363239 | 11782 |
| IL-20 | 16 | 80689313 | T | G | -0.1546 | 0.0316 | 0.9553 | 9.96E-07 | rs117977288 | 11784 |
| IL-20 | 4 | 1.33E+08 | A | G | 0.0635 | 0.0137 | 0.3202 | 3.57E-06 | rs12498781 | 11763 |
| IL-20 | 8 | 68839107 | A | G | -0.3596 | 0.0778 | 0.9909 | 3.80E-06 | rs138771212 | 11336 |
| IL-20 | 7 | 17753237 | T | C | 0.0719 | 0.0156 | 0.5043 | 4.05E-06 | rs143181662 | 8587 |
| IL-20 | 2 | 1.42E+08 | T | C | -0.2551 | 0.0515 | 0.0228 | 7.29E-07 | rs146918864 | 11438 |
| IL-20 | 11 | 1.01E+08 | T | C | -0.222 | 0.0427 | 0.0255 | 2.00E-07 | rs188205021 | 11774 |
| IL-20 | 7 | 76239511 | A | G | -0.3598 | 0.071 | 0.0177 | 4.03E-07 | rs200680034 | 8243 |
| IL-20 | 15 | 23769949 | T | C | -0.1753 | 0.0372 | 0.965 | 2.45E-06 | rs28542561 | 11774 |
| IL-20 | 1 | 9746065 | T | G | -0.1722 | 0.0376 | 0.0444 | 4.65E-06 | rs72633862 | 11774 |
| IL-20 | 14 | 21169862 | T | C | -0.1232 | 0.0267 | 0.9142 | 3.95E-06 | rs72659995 | 11336 |
| IL-20RA | 8 | 1.4E+08 | A | G | 0.1913 | 0.0407 | 0.972 | 2.60E-06 | rs113153423 | 11792 |
| IL-20RA | 7 | 12560554 | A | T | 0.3176 | 0.0676 | 0.9871 | 2.62E-06 | rs113563938 | 11344 |
| IL-20RA | 8 | 35154239 | A | G | 0.1171 | 0.0198 | 0.1374 | 3.34E-09 | rs11987533 | 11344 |
| IL-20RA | 8 | 79840734 | T | G | -0.0761 | 0.0166 | 0.7494 | 4.55E-06 | rs12056343 | 9894 |
| IL-20RA | 1 | 1.68E+08 | C | G | 0.119 | 0.0256 | 0.1039 | 3.34E-06 | rs12138611 | 11344 |
| IL-20RA | 5 | 46131995 | T | C | -0.4166 | 0.091 | 0.0114 | 4.69E-06 | rs143067549 | 7315 |
| IL-20RA | 17 | 59389022 | A | G | 0.2357 | 0.0511 | 0.0208 | 3.98E-06 | rs149111746 | 11783 |
| IL-20RA | 1 | 1.53E+08 | A | C | -0.0817 | 0.0155 | 0.4221 | 1.36E-07 | rs540645152 | 8814 |
| IL-20RA | 3 | 1.13E+08 | A | G | 0.3626 | 0.075 | 0.0114 | 1.33E-06 | rs544388667 | 9261 |
| IL-20RA | 5 | 1.55E+08 | A | G | 0.1741 | 0.038 | 0.0321 | 4.61E-06 | rs59446445 | 11790 |
| IL-20RA | 6 | 1.58E+08 | A | G | -0.0664 | 0.0144 | 0.704 | 4.01E-06 | rs6920753 | 11762 |
| IL-20RA | 16 | 14288564 | T | C | 0.0897 | 0.0195 | 0.8313 | 4.22E-06 | rs72785422 | 11343 |
| IL-20RA1 | 21 | 42295154 | A | G | 0.3467 | 0.0752 | 0.0109 | 4.02E-06 | rs117263973 | 10858 |
| IL-20RA1 | 10 | 44669337 | A | G | 0.2203 | 0.047 | 0.9713 | 2.77E-06 | rs12240324 | 11793 |
| IL-20RA1 | 8 | 87485765 | T | C | 0.2714 | 0.0584 | 0.0167 | 3.36E-06 | rs13257574 | 11306 |
| IL-20RA1 | 4 | 26143082 | A | C | 0.2679 | 0.0561 | 0.0253 | 1.79E-06 | rs1473089 | 10916 |
| IL-20RA1 | 14 | 62394907 | T | C | -0.0751 | 0.0162 | 0.7694 | 3.56E-06 | rs17256492 | 11345 |
| IL-20RA1 | 9 | 1.18E+08 | T | C | 0.27 | 0.0582 | 0.0163 | 3.50E-06 | rs3181361 | 11345 |
| IL-20RA1 | 20 | 50001752 | T | C | 0.0632 | 0.0134 | 0.5504 | 2.40E-06 | rs4811167 | 11793 |
| IL-20RA1 | 8 | 81502336 | A | G | 0.2815 | 0.0571 | 0.0165 | 8.23E-07 | rs56282088 | 10919 |
| IL-20RA1 | 14 | 94197473 | A | G | -0.0674 | 0.0138 | 0.6092 | 1.04E-06 | rs7158900 | 11344 |
| IL-20RA1 | 4 | 1.57E+08 | A | G | 0.1722 | 0.037 | 0.0376 | 3.25E-06 | rs75590335 | 11345 |
| IL-20RA1 | 2 | 85320044 | T | C | -0.1271 | 0.0273 | 0.0671 | 3.23E-06 | rs78594373 | 11767 |
| IL-20RA1 | 7 | 81154741 | A | T | -0.2102 | 0.0412 | 0.9695 | 3.36E-07 | rs79229049 | 11781 |
| IL-24 | 12 | 1.13E+08 | A | G | 0.0655 | 0.0137 | 0.4172 | 1.74E-06 | rs11066309 | 11336 |
| IL-24 | 10 | 6122343 | A | G | 0.3005 | 0.0641 | 0.0145 | 2.76E-06 | rs11256610 | 10850 |
| IL-24 | 1 | 18931322 | T | C | 0.1729 | 0.0371 | 0.0521 | 3.16E-06 | rs116790447 | 9453 |
| IL-24 | 14 | 97741799 | A | G | -0.3 | 0.0657 | 0.9862 | 4.97E-06 | rs117613902 | 10850 |
| IL-24 | 21 | 26067535 | A | G | 0.2969 | 0.0644 | 0.0144 | 4.02E-06 | rs143986178 | 10850 |
| IL-24 | 7 | 9959098 | A | T | 0.3063 | 0.0647 | 0.014 | 2.20E-06 | rs145295031 | 11287 |
| IL-24 | 7 | 1.56E+08 | A | G | 0.0657 | 0.0136 | 0.4443 | 1.36E-06 | rs167018 | 11336 |
| IL-24 | 1 | 1.53E+08 | A | T | 0.0762 | 0.0162 | 0.4768 | 2.55E-06 | rs199770642 | 7943 |
| IL-24 | 5 | 1.49E+08 | A | G | -0.1318 | 0.028 | 0.0716 | 2.51E-06 | rs2341294 | 9900 |
| IL-24 | 1 | 2.37E+08 | T | C | 0.0815 | 0.0172 | 0.4915 | 2.15E-06 | rs36183478 | 8587 |
| IL-24 | 16 | 49575164 | A | G | -0.0886 | 0.0191 | 0.1398 | 3.51E-06 | rs60306270 | 11781 |
| IL-24 | 22 | 48661109 | A | G | 0.111 | 0.0207 | 0.1327 | 8.22E-08 | rs73169406 | 11337 |
| IL-24 | 14 | 86024172 | A | T | 0.0892 | 0.0195 | 0.2392 | 4.78E-06 | rs7401175 | 7943 |
| IL-2RB | 2 | 1.71E+08 | A | G | 0.232 | 0.0502 | 0.9786 | 3.81E-06 | rs115890206 | 10403 |
| IL-2RB | 4 | 1.37E+08 | A | G | -0.2101 | 0.0441 | 0.025 | 1.90E-06 | rs116007066 | 10295 |
| IL-2RB | 15 | 84665328 | T | C | -0.2102 | 0.0443 | 0.9766 | 2.09E-06 | rs11630708 | 11788 |
| IL-2RB | 17 | 4507431 | T | C | 0.1213 | 0.0261 | 0.0695 | 3.36E-06 | rs117538197 | 11774 |
| IL-2RB | 2 | 31896839 | T | C | 0.2769 | 0.0557 | 0.9745 | 6.65E-07 | rs13001788 | 8243 |
| IL-2RB | 11 | 40470032 | T | C | -0.2471 | 0.0535 | 0.9783 | 3.86E-06 | rs141460846 | 11304 |
| IL-2RB | 7 | 98121843 | C | G | 0.0704 | 0.0144 | 0.346 | 1.01E-06 | rs1472270 | 11344 |
| IL-2RB | 4 | 1.08E+08 | T | C | 0.0631 | 0.0129 | 0.5622 | 1.00E-06 | rs17549820 | 11791 |
| IL-2RB | 12 | 1.12E+08 | T | G | -0.1133 | 0.0239 | 0.0879 | 2.13E-06 | rs2157876 | 11343 |
| IL-2RB | 11 | 36198233 | T | C | -0.0721 | 0.0156 | 0.2619 | 3.80E-06 | rs34246740 | 11344 |
| IL-2RB | 6 | 1.31E+08 | A | C | -0.157 | 0.0325 | 0.9549 | 1.36E-06 | rs9321271 | 11779 |
| IL-2RB | 16 | 80775714 | A | G | -0.0606 | 0.0128 | 0.4465 | 2.20E-06 | rs9921181 | 11786 |
| IL-33 | 21 | 40484010 | T | C | -0.0732 | 0.0158 | 0.2201 | 3.61E-06 | rs1013434 | 11783 |
| IL-33 | 19 | 14299739 | A | C | -0.1184 | 0.0233 | 0.1177 | 3.74E-07 | rs10415966 | 10281 |
| IL-33 | 18 | 73359115 | A | G | 0.3432 | 0.0748 | 0.0112 | 4.47E-06 | rs112495884 | 10858 |
| IL-33 | 16 | 79491576 | A | C | 0.0936 | 0.0203 | 0.8755 | 4.01E-06 | rs13336496 | 11780 |
| IL-33 | 7 | 24423445 | C | G | 0.1833 | 0.04 | 0.0307 | 4.59E-06 | rs17149396 | 11789 |
| IL-33 | 4 | 1.89E+08 | T | G | 0.1741 | 0.0364 | 0.047 | 1.73E-06 | rs35370544 | 8385 |
| IL-33 | 9 | 21663051 | C | G | 0.1308 | 0.0277 | 0.9172 | 2.34E-06 | rs62556318 | 11345 |
| IL-33 | 8 | 87995921 | A | G | -0.0752 | 0.0162 | 0.2321 | 3.45E-06 | rs66755903 | 11345 |
| IL-33 | 6 | 38719850 | C | G | 0.0691 | 0.0148 | 0.6997 | 3.03E-06 | rs7742625 | 11345 |
| IL-33 | 6 | 1.57E+08 | T | C | -0.1166 | 0.0255 | 0.1064 | 4.82E-06 | rs77792454 | 8817 |
| IL-33 | 12 | 96439814 | A | G | 0.1259 | 0.0273 | 0.9273 | 3.99E-06 | rs78455659 | 11345 |
| IL-33 | 15 | 66970271 | T | C | -0.0681 | 0.0135 | 0.5496 | 4.55E-07 | rs8042883 | 11345 |
| IL-4 | 10 | 34284406 | A | G | 0.2805 | 0.0592 | 0.9846 | 2.16E-06 | rs116963481 | 11793 |
| IL-4 | 8 | 22649912 | A | G | 0.0678 | 0.0136 | 0.5982 | 6.19E-07 | rs11989681 | 11345 |
| IL-4 | 4 | 1.86E+08 | C | G | -0.1181 | 0.025 | 0.9065 | 2.31E-06 | rs12641440 | 11345 |
| IL-4 | 15 | 79066932 | A | G | 0.0716 | 0.0152 | 0.4842 | 2.47E-06 | rs12900168 | 10471 |
| IL-4 | 4 | 40156881 | A | G | -0.0773 | 0.0159 | 0.2374 | 1.16E-06 | rs13130198 | 11345 |
| IL-4 | 2 | 1.08E+08 | A | G | 0.0701 | 0.0153 | 0.7233 | 4.61E-06 | rs13418255 | 11345 |
| IL-4 | 9 | 1.17E+08 | T | G | -0.1338 | 0.0278 | 0.0752 | 1.49E-06 | rs1490743 | 10281 |
| IL-4 | 1 | 2.43E+08 | T | C | -0.077 | 0.0167 | 0.2195 | 4.01E-06 | rs2502347 | 11345 |
| IL-4 | 8 | 1.19E+08 | T | G | -0.1391 | 0.0299 | 0.9451 | 3.28E-06 | rs4876407 | 11788 |
| IL-4 | 10 | 6800809 | T | C | -0.2354 | 0.045 | 0.0253 | 1.68E-07 | rs75610895 | 11787 |
| IL-4 | 6 | 32556783 | T | C | 0.1212 | 0.0255 | 0.1501 | 2.00E-06 | rs9270229 | 6252 |
| IL-5 | 9 | 1.32E+08 | A | G | 0.0777 | 0.0167 | 0.2002 | 3.28E-06 | rs10988407 | 11791 |
| IL-5 | 1 | 37473314 | T | C | 0.1661 | 0.0361 | 0.0436 | 4.20E-06 | rs114829303 | 11344 |
| IL-5 | 15 | 70546132 | T | C | 0.0838 | 0.0173 | 0.8068 | 1.27E-06 | rs12592185 | 11344 |
| IL-5 | 22 | 48143913 | T | C | -0.0686 | 0.0141 | 0.3408 | 1.14E-06 | rs132210 | 11344 |
| IL-5 | 6 | 1.24E+08 | A | G | 0.3833 | 0.0825 | 0.9859 | 3.38E-06 | rs141941146 | 10280 |
| IL-5 | 1 | 2.08E+08 | A | G | -0.1169 | 0.0236 | 0.0881 | 7.29E-07 | rs17009223 | 11789 |
| IL-5 | 5 | 95530542 | A | G | 0.0765 | 0.0147 | 0.7289 | 1.95E-07 | rs34639000 | 11773 |
| IL-5 | 11 | 10380223 | A | G | -0.2747 | 0.0592 | 0.9858 | 3.48E-06 | rs4277106 | 11790 |
| IL-5 | 17 | 59402478 | A | G | 0.2206 | 0.0482 | 0.0267 | 4.72E-06 | rs62069649 | 10431 |
| IL-5 | 14 | 93316119 | A | G | -0.0924 | 0.0188 | 0.1442 | 8.88E-07 | rs7142197 | 11773 |
| IL-5 | 16 | 86933894 | C | G | 0.2407 | 0.0521 | 0.0351 | 3.84E-06 | rs7193141 | 9377 |
| IL-6 | 7 | 28486167 | T | C | 0.0582 | 0.0127 | 0.6371 | 4.59E-06 | rs10234370 | 14295 |
| IL-6 | 9 | 954741 | A | C | -0.3418 | 0.0701 | 0.9865 | 1.08E-06 | rs10977774 | 12312 |
| IL-6 | 8 | 21260780 | A | G | -0.0659 | 0.0135 | 0.7459 | 1.05E-06 | rs1346147 | 14295 |
| IL-6 | 2 | 1.14E+08 | T | G | 0.0975 | 0.0211 | 0.9152 | 3.82E-06 | rs17486819 | 14739 |
| IL-6 | 13 | 68942611 | T | G | -0.3427 | 0.0682 | 0.0105 | 5.04E-07 | rs185969953 | 12934 |
| IL-6 | 7 | 76792340 | T | C | -0.2244 | 0.0469 | 0.0182 | 1.71E-06 | rs187416244 | 14726 |
| IL-6 | 1 | 1.54E+08 | A | C | -0.1684 | 0.0119 | 0.6128 | 1.83E-45 | rs2228145 | 14742 |
| IL-6 | 5 | 1.41E+08 | T | G | -0.0671 | 0.0143 | 0.2276 | 2.70E-06 | rs248536 | 14295 |
| IL-7 | 19 | 14764810 | A | G | -0.0584 | 0.0127 | 0.2923 | 4.26E-06 | rs10403201 | 14730 |
| IL-7 | 5 | 1.32E+08 | T | C | -0.0651 | 0.0133 | 0.4122 | 9.84E-07 | rs1050152 | 11785 |
| IL-7 | 8 | 1.07E+08 | T | C | 0.0745 | 0.014 | 0.3642 | 1.03E-07 | rs111066336 | 11538 |
| IL-7 | 8 | 79713766 | A | G | 0.1691 | 0.0225 | 0.9 | 5.67E-14 | rs112359206 | 10894 |
| IL-7 | 5 | 35881376 | T | G | -0.0647 | 0.0131 | 0.2796 | 7.86E-07 | rs11742240 | 14736 |
| IL-7 | 12 | 97807415 | A | G | 0.0698 | 0.0139 | 0.237 | 5.12E-07 | rs12321566 | 14287 |
| IL-7 | 7 | 2401569 | A | G | 0.068 | 0.0138 | 0.2517 | 8.33E-07 | rs12699881 | 14288 |
| IL-7 | 3 | 35502404 | T | C | 0.1495 | 0.0316 | 0.0436 | 2.23E-06 | rs144728120 | 14717 |
| IL-7 | 5 | 1.34E+08 | A | G | 0.0729 | 0.0159 | 0.7913 | 4.54E-06 | rs55849454 | 12202 |
| IL-7 | 1 | 1.74E+08 | A | G | 0.097 | 0.021 | 0.8744 | 3.86E-06 | rs56889120 | 11340 |
| IL-7 | 15 | 73960703 | T | C | -0.2336 | 0.0505 | 0.0215 | 3.73E-06 | rs570880779 | 13605 |
| IL-7 | 4 | 1.48E+08 | A | T | 0.1597 | 0.0345 | 0.9615 | 3.67E-06 | rs576004227 | 12045 |
| IL-7 | 9 | 1.34E+08 | A | G | 0.0691 | 0.0148 | 0.5682 | 3.03E-06 | rs7026771 | 12403 |
| IL-7 | 1 | 1.94E+08 | A | G | 0.1372 | 0.0274 | 0.9451 | 5.52E-07 | rs74131483 | 13240 |
| IL-7 | 1 | 97287518 | A | C | 0.2512 | 0.0531 | 0.9836 | 2.24E-06 | rs77336315 | 13801 |
| IL-7 | 15 | 81321256 | T | C | -0.136 | 0.028 | 0.0497 | 1.19E-06 | rs79072837 | 14715 |
| IL-8 | 1 | 1.59E+08 | A | G | 0.0667 | 0.0116 | 0.5552 | 8.92E-09 | rs12075 | 14741 |
| IL-8 | 5 | 1.21E+08 | A | T | -0.0755 | 0.0147 | 0.2006 | 2.81E-07 | rs12188297 | 14740 |
| IL-8 | 3 | 16705058 | T | C | 0.056 | 0.012 | 0.4104 | 3.06E-06 | rs13064825 | 14296 |
| IL-8 | 3 | 1.73E+08 | T | C | 0.2829 | 0.0608 | 0.0146 | 3.27E-06 | rs141979722 | 11669 |
| IL-8 | 1 | 2.34E+08 | T | C | 0.3186 | 0.0622 | 0.9879 | 3.02E-07 | rs144815207 | 13394 |
| IL-8 | 7 | 17688229 | A | G | 0.2687 | 0.0555 | 0.9866 | 1.29E-06 | rs145125644 | 14733 |
| IL-8 | 3 | 76218188 | T | C | 0.1479 | 0.0322 | 0.0386 | 4.37E-06 | rs1516478 | 14741 |
| IL-8 | 18 | 12258051 | T | C | 0.0555 | 0.012 | 0.5073 | 3.75E-06 | rs34519991 | 14296 |
| IL-8 | 4 | 74574265 | A | G | -0.1894 | 0.0241 | 0.0665 | 3.87E-15 | rs6446951 | 14729 |
| IL-8 | 10 | 1.19E+08 | T | C | 0.1545 | 0.0325 | 0.9598 | 2.00E-06 | rs6585420 | 14744 |
| IL-8 | 2 | 1.09E+08 | A | G | 0.0562 | 0.0117 | 0.4424 | 1.56E-06 | rs6709159 | 14744 |
| IL-8 | 17 | 73572798 | A | G | -0.1324 | 0.0279 | 0.0541 | 2.08E-06 | rs7216538 | 13672 |
| IL-8 | 15 | 1.02E+08 | A | C | 0.0574 | 0.0124 | 0.4025 | 3.67E-06 | rs72633250 | 14296 |
| IL-8 | 18 | 36692976 | T | G | -0.3895 | 0.076 | 0.0095 | 2.98E-07 | rs72886502 | 12291 |
| IL-8 | 17 | 54450134 | A | G | 0.0668 | 0.0131 | 0.7222 | 3.41E-07 | rs8069322 | 14726 |
| IL-8 | 18 | 73700336 | T | C | 0.0941 | 0.02 | 0.9034 | 2.54E-06 | rs906401 | 14729 |
| IL-8 | 5 | 37903927 | A | C | -0.0936 | 0.0184 | 0.1404 | 3.64E-07 | rs972492 | 12412 |
| LAP TGF-beta-1 | 19 | 14764810 | A | G | -0.0597 | 0.0125 | 0.292 | 1.79E-06 | rs10403201 | 14730 |
| LAP TGF-beta-1 | 10 | 18285342 | T | C | 0.0523 | 0.0114 | 0.4627 | 4.48E-06 | rs10508556 | 14735 |
| LAP TGF-beta-1 | 4 | 1.76E+08 | T | C | -0.299 | 0.0613 | 0.0112 | 1.07E-06 | rs114152855 | 14249 |
| LAP TGF-beta-1 | 8 | 51696312 | T | C | 0.1447 | 0.0303 | 0.0388 | 1.79E-06 | rs143420877 | 14729 |
| LAP TGF-beta-1 | 12 | 24809427 | T | C | -0.2662 | 0.0526 | 0.0227 | 4.17E-07 | rs144834546 | 12935 |
| LAP TGF-beta-1 | 16 | 48902293 | A | C | -0.2301 | 0.0472 | 0.0182 | 1.09E-06 | rs149458216 | 14726 |
| LAP TGF-beta-1 | 19 | 41847860 | A | G | -0.4623 | 0.0335 | 0.0345 | 2.55E-43 | rs1800472 | 14736 |
| LAP TGF-beta-1 | 9 | 87111083 | T | G | 0.2185 | 0.0461 | 0.0182 | 2.14E-06 | rs183319799 | 14733 |
| LAP TGF-beta-1 | 4 | 84952443 | A | C | 0.1265 | 0.0263 | 0.9391 | 1.51E-06 | rs1921885 | 14288 |
| LAP TGF-beta-1 | 22 | 45002724 | T | C | -0.0873 | 0.019 | 0.1204 | 4.33E-06 | rs2281119 | 14284 |
| LAP TGF-beta-1 | 5 | 1.32E+08 | C | G | -0.0605 | 0.013 | 0.4529 | 3.26E-06 | rs2631367 | 11785 |
| LAP TGF-beta-1 | 11 | 95274487 | T | G | 0.0989 | 0.021 | 0.9023 | 2.48E-06 | rs34536806 | 14288 |
| LAP TGF-beta-1 | 7 | 16406570 | T | C | -0.0931 | 0.0199 | 0.1103 | 2.89E-06 | rs34992551 | 14734 |
| LAP TGF-beta-1 | 12 | 95312882 | A | C | -0.1083 | 0.0232 | 0.0779 | 3.04E-06 | rs60821320 | 12852 |
| LAP TGF-beta-1 | 8 | 1.07E+08 | A | G | -0.0608 | 0.0121 | 0.4469 | 5.04E-07 | rs7013321 | 14288 |
| LAP TGF-beta-1 | 19 | 41811072 | A | G | -0.1921 | 0.0276 | 0.9378 | 3.40E-12 | rs73045256 | 14288 |
| LAP TGF-beta-1 | 4 | 26142254 | C | G | 0.2053 | 0.0433 | 0.9689 | 2.12E-06 | rs73112514 | 12393 |
| LAP TGF-beta-1 | 2 | 1.07E+08 | A | G | -0.0849 | 0.0183 | 0.8857 | 3.50E-06 | rs7570818 | 14729 |
| LAP TGF-beta-1 | 4 | 5793052 | A | T | 0.0626 | 0.0121 | 0.5957 | 2.30E-07 | rs7656674 | 14288 |
| LAP TGF-beta-1 | 8 | 68719454 | T | C | -0.0747 | 0.0155 | 0.8321 | 1.44E-06 | rs7820135 | 14730 |
| LIF | 2 | 3091471 | T | C | 0.0707 | 0.0153 | 0.3559 | 3.82E-06 | rs11127419 | 9252 |
| LIF | 12 | 68026044 | A | T | -0.3084 | 0.0661 | 0.0168 | 3.08E-06 | rs11608349 | 9362 |
| LIF | 2 | 2.24E+08 | C | G | -0.3015 | 0.0631 | 0.9851 | 1.77E-06 | rs116794364 | 10858 |
| LIF | 19 | 54851842 | A | G | -0.1011 | 0.0197 | 0.1385 | 2.87E-07 | rs11883375 | 11776 |
| LIF | 8 | 1.09E+08 | A | T | 0.0757 | 0.0158 | 0.6575 | 1.66E-06 | rs13264315 | 9460 |
| LIF | 3 | 58954742 | C | G | 0.2548 | 0.0556 | 0.021 | 4.59E-06 | rs145344087 | 10281 |
| LIF | 14 | 97087097 | A | G | -0.0635 | 0.0136 | 0.5456 | 3.02E-06 | rs234592 | 11345 |
| LIF | 19 | 6801966 | A | G | 0.112 | 0.0238 | 0.0899 | 2.53E-06 | rs332426 | 11789 |
| LIF | 17 | 28341679 | T | G | 0.0671 | 0.0143 | 0.486 | 2.70E-06 | rs7225483 | 9849 |
| LIF | 10 | 31490202 | A | G | 0.1725 | 0.0367 | 0.0351 | 2.60E-06 | rs74992559 | 11784 |
| LIF | 2 | 46125903 | A | T | 0.0993 | 0.0213 | 0.8758 | 3.13E-06 | rs80092539 | 11345 |
| LIF | 2 | 2.23E+08 | A | G | -0.2119 | 0.0453 | 0.0321 | 2.90E-06 | rs825264 | 10291 |
| LIF-R | 5 | 1.11E+08 | T | C | -0.2181 | 0.0467 | 0.0215 | 3.01E-06 | rs115938660 | 11770 |
| LIF-R | 8 | 1.2E+08 | A | G | 0.1586 | 0.0333 | 0.961 | 1.91E-06 | rs117117000 | 11784 |
| LIF-R | 1 | 2.39E+08 | A | G | 0.0563 | 0.0122 | 0.4817 | 3.94E-06 | rs12754806 | 11768 |
| LIF-R | 17 | 36964094 | A | G | -0.2724 | 0.0546 | 0.0169 | 6.07E-07 | rs141933232 | 11724 |
| LIF-R | 21 | 43780807 | A | G | -0.2602 | 0.0566 | 0.0179 | 4.28E-06 | rs147006060 | 11775 |
| LIF-R | 10 | 27930818 | A | C | 0.0904 | 0.0192 | 0.1109 | 2.50E-06 | rs1690678 | 11784 |
| LIF-R | 5 | 38527228 | T | G | 0.0788 | 0.0134 | 0.5419 | 4.09E-09 | rs2289779 | 11336 |
| LIF-R | 3 | 12385357 | A | C | -0.0951 | 0.0203 | 0.1784 | 2.80E-06 | rs3963364 | 9453 |
| LIF-R | 9 | 1.36E+08 | T | C | 0.2027 | 0.0299 | 0.067 | 1.21E-11 | rs41307428 | 11774 |
| LIF-R | 11 | 1.26E+08 | T | G | 0.1262 | 0.0248 | 0.9129 | 3.61E-07 | rs4937122 | 11336 |
| LIF-R | 22 | 41054967 | A | G | 0.6489 | 0.1417 | 0.0043 | 4.66E-06 | rs6002001 | 6861 |
| LIF-R | 3 | 58363371 | T | C | -0.063 | 0.013 | 0.6603 | 1.26E-06 | rs62258077 | 11762 |
| LIF-R | 9 | 1.36E+08 | T | C | -0.2885 | 0.0153 | 0.1918 | 2.61E-79 | rs635634 | 11784 |
| LIF-R | 5 | 36424241 | A | T | 0.1414 | 0.0302 | 0.9403 | 2.84E-06 | rs72730728 | 11336 |
| LIF-R | 17 | 7063650 | T | C | -0.08 | 0.0172 | 0.7807 | 3.30E-06 | rs72837682 | 11336 |
| LIF-R | 7 | 79349730 | T | C | -0.1306 | 0.0285 | 0.9229 | 4.60E-06 | rs73157388 | 11336 |
| LIF-R | 6 | 68918580 | A | G | -0.0681 | 0.0145 | 0.2875 | 2.65E-06 | rs778016 | 9883 |
| LIF-R | 5 | 6378192 | C | G | -0.0824 | 0.0173 | 0.1414 | 1.91E-06 | rs9313148 | 11777 |
| CCL8 | 16 | 4938452 | C | G | 0.1734 | 0.0377 | 0.0256 | 4.24E-06 | rs117265389 | 14733 |
| CCL8 | 22 | 21921920 | T | G | -0.3227 | 0.0704 | 0.0125 | 4.57E-06 | rs117317477 | 11725 |
| CCL8 | 11 | 87277670 | T | C | -0.2587 | 0.0562 | 0.9806 | 4.16E-06 | rs117510656 | 14285 |
| CCL8 | 17 | 32567679 | T | C | 0.0751 | 0.013 | 0.2772 | 7.61E-09 | rs11870164 | 14733 |
| CCL8 | 1 | 1.59E+08 | A | G | 0.1694 | 0.0113 | 0.5552 | 8.39E-51 | rs12075 | 14730 |
| CCL8 | 1 | 14260220 | T | G | 0.0728 | 0.0157 | 0.8345 | 3.54E-06 | rs12133241 | 14713 |
| CCL8 | 12 | 30998369 | A | G | 0.0739 | 0.0159 | 0.1756 | 3.35E-06 | rs12367122 | 14284 |
| CCL8 | 6 | 20408729 | T | G | -0.2293 | 0.0497 | 0.9789 | 3.96E-06 | rs146186562 | 14285 |
| CCL8 | 5 | 2885014 | A | G | 0.2553 | 0.0538 | 0.0243 | 2.08E-06 | rs148307902 | 13798 |
| CCL8 | 3 | 1.85E+08 | C | G | -0.2813 | 0.0556 | 0.9851 | 4.21E-07 | rs149604038 | 13798 |
| CCL8 | 3 | 46519788 | T | C | 0.3723 | 0.0695 | 0.9872 | 8.47E-08 | rs181998213 | 14285 |
| CCL8 | 8 | 1.15E+08 | A | T | -0.4028 | 0.0864 | 0.991 | 3.13E-06 | rs182599222 | 11334 |
| CCL8 | 3 | 46032441 | A | T | 0.0856 | 0.0119 | 0.362 | 6.33E-13 | rs1846616 | 14719 |
| CCL8 | 18 | 75133468 | A | G | -0.2084 | 0.0455 | 0.0286 | 4.64E-06 | rs190108694 | 11325 |
| CCL8 | 4 | 19075878 | T | C | 0.0579 | 0.0126 | 0.6697 | 4.32E-06 | rs207311 | 14285 |
| CCL8 | 13 | 43553825 | T | C | -0.0684 | 0.0134 | 0.2742 | 3.32E-07 | rs34292320 | 14285 |
| CCL8 | 3 | 46390228 | A | G | 0.3004 | 0.0198 | 0.0959 | 5.44E-52 | rs35728689 | 14732 |
| CCL8 | 3 | 42877413 | A | G | 0.0576 | 0.0122 | 0.6154 | 2.34E-06 | rs4682862 | 14285 |
| CCL8 | 3 | 1.32E+08 | A | G | 0.0986 | 0.0201 | 0.118 | 9.32E-07 | rs7619350 | 11764 |
| CCL8 | 20 | 15213518 | T | C | -0.1063 | 0.0218 | 0.0875 | 1.08E-06 | rs77128226 | 14285 |
| CCL8 | 9 | 78838039 | T | C | -0.1381 | 0.0293 | 0.0491 | 2.44E-06 | rs77344300 | 14733 |
| CCL8 | 20 | 9086080 | T | C | -0.348 | 0.0653 | 0.0136 | 9.86E-08 | rs79697698 | 13752 |
| CCL8 | 7 | 17977054 | T | C | 0.0618 | 0.0117 | 0.4922 | 1.28E-07 | rs9942602 | 14728 |
| CCL2 | 16 | 4704769 | T | C | -0.1553 | 0.0316 | 0.9555 | 8.90E-07 | rs112168535 | 13670 |
| CCL2 | 17 | 32632378 | A | G | -0.9051 | 0.0354 | 0.0323 | 3.48E-144 | rs11652256 | 14736 |
| CCL2 | 1 | 1.59E+08 | A | G | -0.0973 | 0.0116 | 0.5553 | 4.95E-17 | rs12075 | 14733 |
| CCL2 | 5 | 1.66E+08 | A | T | 0.1121 | 0.0232 | 0.0746 | 1.35E-06 | rs12523126 | 14288 |
| CCL2 | 3 | 47071457 | T | C | 0.2198 | 0.0473 | 0.0188 | 3.37E-06 | rs138884871 | 13672 |
| CCL2 | 17 | 32774385 | T | C | -0.3694 | 0.0547 | 0.014 | 1.45E-11 | rs144101664 | 14288 |
| CCL2 | 17 | 32652307 | T | C | 0.5171 | 0.0538 | 0.0169 | 7.15E-22 | rs144618566 | 13801 |
| CCL2 | 5 | 1.47E+08 | A | G | 0.2225 | 0.048 | 0.979 | 3.56E-06 | rs145516279 | 14288 |
| CCL2 | 17 | 32633263 | T | C | -0.4966 | 0.0757 | 0.9763 | 5.38E-11 | rs16969443 | 10826 |
| CCL2 | 9 | 95657986 | A | G | -0.2479 | 0.0536 | 0.9838 | 3.75E-06 | rs181726805 | 14736 |
| CCL2 | 17 | 32649988 | T | C | 0.4013 | 0.0478 | 0.0184 | 4.64E-17 | rs1821142 | 13422 |
| CCL2 | 17 | 32625248 | C | G | -0.3591 | 0.0509 | 0.981 | 1.73E-12 | rs188234686 | 12847 |
| CCL2 | 17 | 32647357 | A | T | 0.6652 | 0.0371 | 0.9715 | 6.89E-72 | rs34202026 | 14719 |
| CCL2 | 17 | 76944702 | T | C | 0.0761 | 0.0165 | 0.2662 | 3.99E-06 | rs35904471 | 11337 |
| CCL2 | 5 | 1.25E+08 | A | C | 0.0697 | 0.0147 | 0.2073 | 2.12E-06 | rs407595 | 14287 |
| CCL2 | 7 | 30472091 | T | C | -0.0626 | 0.0133 | 0.709 | 2.52E-06 | rs4722985 | 14288 |
| CCL2 | 1 | 2.25E+08 | A | G | 0.1462 | 0.0289 | 0.0505 | 4.22E-07 | rs6685547 | 13239 |
| CCL2 | 21 | 36741897 | A | G | 0.2798 | 0.0608 | 0.012 | 4.19E-06 | rs74350159 | 13801 |
| CCL2 | 1 | 2.39E+08 | C | G | 0.0566 | 0.0122 | 0.6032 | 3.50E-06 | rs7538845 | 14288 |
| CCL2 | 11 | 1.01E+08 | C | G | 0.1906 | 0.0408 | 0.976 | 2.99E-06 | rs75573205 | 14725 |
| CCL2 | 19 | 2151761 | T | C | -0.0904 | 0.0188 | 0.8703 | 1.52E-06 | rs75580960 | 13224 |
| CCL2 | 17 | 32645129 | A | G | 0.296 | 0.0455 | 0.0187 | 7.74E-11 | rs76263310 | 14727 |
| CCL2 | 1 | 3301962 | A | C | -0.2226 | 0.048 | 0.019 | 3.53E-06 | rs78743764 | 13656 |
| CCL2 | 6 | 1.67E+08 | A | C | -0.3284 | 0.0669 | 0.9877 | 9.16E-07 | rs79567569 | 13386 |
| CCL2 | 2 | 43748079 | A | G | 0.1092 | 0.0233 | 0.0823 | 2.78E-06 | rs79965992 | 12851 |
| CCL2 | 18 | 31677370 | A | G | 0.1962 | 0.0399 | 0.9686 | 8.77E-07 | rs80131163 | 14730 |
| CCL2 | 17 | 32610534 | T | G | -0.904 | 0.0322 | 0.0365 | 2.00E-173 | rs80219280 | 14736 |
| CCL2 | 17 | 32602915 | T | C | 0.8196 | 0.0267 | 0.9492 | 1.00E-200 | rs8081047 | 14730 |
| CCL2 | 11 | 76919478 | A | C | 0.0544 | 0.0119 | 0.4764 | 4.84E-06 | rs948962 | 14736 |
| CCL2 | 17 | 4704175 | A | G | 0.0609 | 0.0121 | 0.4783 | 4.83E-07 | rs9889517 | 14288 |
| CCL7 | 3 | 1.77E+08 | T | C | -0.0849 | 0.0167 | 0.5118 | 3.70E-07 | rs11276535 | 7943 |
| CCL7 | 1 | 1.59E+08 | A | G | -0.0899 | 0.013 | 0.5624 | 4.67E-12 | rs12075 | 11780 |
| CCL7 | 1 | 1.93E+08 | T | C | 0.0778 | 0.0166 | 0.6951 | 2.78E-06 | rs12142504 | 9448 |
| CCL7 | 9 | 1.11E+08 | A | G | 0.2334 | 0.0492 | 0.0249 | 2.10E-06 | rs139796674 | 11335 |
| CCL7 | 2 | 68614232 | A | G | 0.2663 | 0.0547 | 0.0247 | 1.13E-06 | rs146174820 | 10469 |
| CCL7 | 1 | 2.02E+08 | A | G | 0.1941 | 0.0413 | 0.0345 | 2.60E-06 | rs147793446 | 11770 |
| CCL7 | 11 | 42245173 | A | G | 0.1507 | 0.0328 | 0.05 | 4.34E-06 | rs148859911 | 11771 |
| CCL7 | 5 | 1.51E+08 | C | G | -0.0638 | 0.0134 | 0.4563 | 1.92E-06 | rs153473 | 11335 |
| CCL7 | 15 | 93092575 | A | G | 0.2264 | 0.0487 | 0.9779 | 3.34E-06 | rs16947053 | 11783 |
| CCL7 | 4 | 1.45E+08 | T | G | -0.1716 | 0.0359 | 0.9096 | 1.75E-06 | rs182239847 | 9453 |
| CCL7 | 3 | 42906116 | T | C | -0.3169 | 0.0252 | 0.9268 | 2.88E-36 | rs2228467 | 11782 |
| CCL7 | 4 | 47682997 | T | G | -0.0613 | 0.0133 | 0.4117 | 4.05E-06 | rs28667091 | 11782 |
| CCL7 | 3 | 1.51E+08 | T | C | -0.0933 | 0.0165 | 0.7978 | 1.56E-08 | rs4146770 | 11760 |
| CCL7 | 1 | 1.09E+08 | A | G | -0.0754 | 0.015 | 0.3263 | 4.99E-07 | rs481267 | 9889 |
| CCL7 | 11 | 68891695 | T | C | -0.0998 | 0.0179 | 0.1822 | 2.47E-08 | rs7116054 | 11335 |
| CCL7 | 17 | 32522613 | A | G | 0.0942 | 0.0132 | 0.4161 | 9.58E-13 | rs7213460 | 11780 |
| CCL7 | 13 | 58931726 | C | G | -0.0969 | 0.0194 | 0.1497 | 5.89E-07 | rs7327202 | 10903 |
| CCL7 | 10 | 1.21E+08 | T | C | 0.1574 | 0.0343 | 0.0404 | 4.46E-06 | rs74157668 | 11781 |
| CCL7 | 20 | 41126657 | T | C | 0.2441 | 0.0514 | 0.0198 | 2.04E-06 | rs78848134 | 11296 |
| CCL7 | 6 | 1E+08 | T | G | -0.1046 | 0.0221 | 0.8991 | 2.21E-06 | rs9968876 | 11774 |
| CCL13 | 14 | 70451473 | A | G | 0.2704 | 0.0553 | 0.0209 | 1.01E-06 | rs10140592 | 13753 |
| CCL13 | 4 | 1.16E+08 | A | G | 0.1956 | 0.042 | 0.9694 | 3.21E-06 | rs115511138 | 13944 |
| CCL13 | 1 | 1.59E+08 | A | G | 0.189 | 0.0114 | 0.5553 | 9.89E-62 | rs12075 | 14733 |
| CCL13 | 6 | 31214807 | A | G | 0.1749 | 0.0368 | 0.963 | 2.01E-06 | rs144118870 | 14735 |
| CCL13 | 17 | 56210459 | T | C | -0.1032 | 0.0208 | 0.8805 | 6.99E-07 | rs181250 | 11538 |
| CCL13 | 10 | 94520346 | T | C | 0.0668 | 0.0145 | 0.2018 | 4.09E-06 | rs2497351 | 14736 |
| CCL13 | 5 | 1.32E+08 | A | G | 0.0609 | 0.0131 | 0.5448 | 3.34E-06 | rs2631360 | 11782 |
| CCL13 | 8 | 1.17E+08 | T | G | -0.1201 | 0.0133 | 0.7293 | 1.72E-19 | rs2721961 | 14288 |
| CCL13 | 17 | 32683289 | A | G | 0.3903 | 0.0251 | 0.9384 | 1.60E-54 | rs3136676 | 14728 |
| CCL13 | 17 | 32587314 | A | G | 0.1557 | 0.012 | 0.6401 | 1.70E-38 | rs3917900 | 14730 |
| CCL13 | 3 | 46289113 | A | G | -0.0638 | 0.0131 | 0.3598 | 1.11E-06 | rs55842014 | 12404 |
| CCL13 | 20 | 12689462 | A | C | -0.2136 | 0.0451 | 0.9807 | 2.18E-06 | rs62201455 | 14722 |
| CCL13 | 4 | 1.76E+08 | T | C | 0.1569 | 0.0332 | 0.0417 | 2.29E-06 | rs62336911 | 14288 |
| CCL13 | 17 | 32529710 | T | G | -0.1997 | 0.0251 | 0.0594 | 1.77E-15 | rs72825017 | 14730 |
| CCL13 | 5 | 22143588 | T | C | 0.0674 | 0.0139 | 0.4123 | 1.24E-06 | rs74794857 | 11760 |
| CCL13 | 3 | 42910621 | T | C | 0.1281 | 0.012 | 0.6276 | 1.33E-26 | rs7612912 | 14714 |
| CCL13 | 19 | 14773077 | A | G | -0.0578 | 0.0126 | 0.2923 | 4.49E-06 | rs8112014 | 14724 |
| CCL13 | 6 | 40051588 | A | G | 0.077 | 0.0168 | 0.8533 | 4.58E-06 | rs847767 | 14733 |
| CCL13 | 20 | 20023326 | T | C | 0.1432 | 0.0304 | 0.9545 | 2.47E-06 | rs926646 | 14732 |
| MIP-1 alpha | 17 | 34042025 | A | G | -0.3088 | 0.0623 | 0.978 | 7.17E-07 | rs111734780 | 12412 |
| MIP-1 alpha | 12 | 67790676 | T | C | -0.0644 | 0.0124 | 0.632 | 2.06E-07 | rs1125560 | 13863 |
| MIP-1 alpha | 7 | 1108954 | A | G | 0.0818 | 0.0179 | 0.1528 | 4.88E-06 | rs113448118 | 11793 |
| MIP-1 alpha | 16 | 81922813 | T | C | -0.1229 | 0.024 | 0.0627 | 3.04E-07 | rs1143687 | 14743 |
| MIP-1 alpha | 17 | 34433029 | A | G | -0.409 | 0.0135 | 0.7667 | 1.00E-200 | rs1634518 | 14295 |
| MIP-1 alpha | 18 | 12747235 | T | C | -0.0621 | 0.0136 | 0.2605 | 4.97E-06 | rs2847249 | 14294 |
| MIP-1 alpha | 6 | 31263051 | A | G | -0.0608 | 0.0133 | 0.7392 | 4.84E-06 | rs2853926 | 14743 |
| MIP-1 alpha | 17 | 34358060 | A | C | -0.1806 | 0.0325 | 0.0383 | 2.75E-08 | rs56675286 | 14743 |
| MIP-1 alpha | 4 | 38801285 | A | G | -0.0736 | 0.0135 | 0.7503 | 4.98E-08 | rs5743604 | 14737 |
| MIP-1 alpha | 17 | 15741465 | T | C | -0.0668 | 0.0143 | 0.7284 | 2.99E-06 | rs677925 | 12411 |
| MIP-1 alpha | 12 | 1.12E+08 | T | C | -0.0837 | 0.0133 | 0.5247 | 3.11E-10 | rs7137828 | 11344 |
| MIP-1 alpha | 16 | 4774017 | T | C | 0.1812 | 0.0371 | 0.0304 | 1.04E-06 | rs78871521 | 13246 |
| MIP-1 alpha | 13 | 25675278 | A | G | -0.0626 | 0.0133 | 0.4935 | 2.52E-06 | rs9507462 | 11767 |
| MIP-1 alpha | 13 | 66085374 | A | G | 0.0926 | 0.0181 | 0.8776 | 3.12E-07 | rs9540456 | 14295 |
| MIP-1 alpha | 17 | 34820996 | A | G | 0.176 | 0.0162 | 0.8208 | 1.71E-27 | rs9911791 | 14295 |
| MMP-1 | 9 | 1.1E+08 | A | T | 0.0649 | 0.014 | 0.7462 | 3.56E-06 | rs10978641 | 14296 |
| MMP-1 | 9 | 99091009 | T | C | 0.0703 | 0.0135 | 0.253 | 1.91E-07 | rs10990535 | 14734 |
| MMP-1 | 19 | 14763748 | T | C | -0.0585 | 0.0127 | 0.296 | 4.10E-06 | rs11673125 | 14741 |
| MMP-1 | 11 | 1.03E+08 | A | G | -0.176 | 0.03 | 0.0455 | 4.45E-09 | rs116889819 | 14744 |
| MMP-1 | 1 | 1.56E+08 | A | G | 0.0859 | 0.013 | 0.6981 | 3.90E-11 | rs12141791 | 14296 |
| MMP-1 | 12 | 83594043 | T | G | 0.0861 | 0.0186 | 0.1146 | 3.67E-06 | rs12367368 | 14744 |
| MMP-1 | 11 | 1.02E+08 | A | G | 0.3767 | 0.0445 | 0.9777 | 2.56E-17 | rs141782294 | 14731 |
| MMP-1 | 7 | 1.21E+08 | T | C | -0.2509 | 0.053 | 0.0177 | 2.20E-06 | rs188626385 | 12935 |
| MMP-1 | 11 | 1.03E+08 | T | C | -0.3212 | 0.0264 | 0.9461 | 4.68E-34 | rs2155053 | 14741 |
| MMP-1 | 2 | 75116527 | A | G | -0.3153 | 0.0673 | 0.0135 | 2.80E-06 | rs2229629 | 10887 |
| MMP-1 | 12 | 57098597 | T | C | -0.0704 | 0.0126 | 0.3436 | 2.31E-08 | rs2926741 | 14296 |
| MMP-1 | 8 | 1.07E+08 | A | G | 0.1136 | 0.0137 | 0.7422 | 1.11E-16 | rs4734879 | 14296 |
| MMP-1 | 11 | 1.03E+08 | A | T | -0.3517 | 0.0119 | 0.5613 | 5.73E-192 | rs484915 | 14295 |
| MMP-1 | 2 | 63657053 | A | T | 0.1548 | 0.0338 | 0.966 | 4.65E-06 | rs6717478 | 14742 |
| MMP-1 | 17 | 4351312 | A | G | 0.0569 | 0.0121 | 0.5023 | 2.57E-06 | rs7219205 | 14296 |
| MMP-1 | 11 | 1.03E+08 | T | G | -0.1416 | 0.0238 | 0.0705 | 2.69E-09 | rs77095316 | 14744 |
| MMP-1 | 3 | 64991445 | T | C | 0.1234 | 0.0234 | 0.0684 | 1.34E-07 | rs873675 | 14742 |
| MMP-10 | 6 | 1.34E+08 | T | G | 0.1203 | 0.0249 | 0.0601 | 1.36E-06 | rs112062276 | 14733 |
| MMP-10 | 11 | 1.03E+08 | T | C | 0.2957 | 0.0181 | 0.879 | 5.38E-60 | rs11225354 | 14296 |
| MMP-10 | 8 | 77953661 | C | G | -0.2862 | 0.0608 | 0.0153 | 2.51E-06 | rs112327130 | 12456 |
| MMP-10 | 20 | 20711695 | A | G | -0.2315 | 0.0503 | 0.0188 | 4.18E-06 | rs113958571 | 14296 |
| MMP-10 | 1 | 1.6E+08 | A | C | 0.07 | 0.0153 | 0.822 | 4.76E-06 | rs12567824 | 14296 |
| MMP-10 | 2 | 1.41E+08 | T | G | -0.069 | 0.015 | 0.7974 | 4.22E-06 | rs1356799 | 13869 |
| MMP-10 | 11 | 1.03E+08 | A | G | -0.3256 | 0.0494 | 0.9794 | 4.37E-11 | rs142915220 | 13809 |
| MMP-10 | 11 | 1.03E+08 | A | G | -0.2064 | 0.0233 | 0.9296 | 8.12E-19 | rs17099622 | 14296 |
| MMP-10 | 11 | 1.03E+08 | T | C | 1.1371 | 0.047 | 0.983 | 2.60E-129 | rs17860955 | 14256 |
| MMP-10 | 6 | 31423412 | A | G | -0.1769 | 0.0358 | 0.0368 | 7.76E-07 | rs181816009 | 11985 |
| MMP-10 | 17 | 47470487 | A | C | -0.0597 | 0.0122 | 0.585 | 9.91E-07 | rs2584662 | 14296 |
| MMP-10 | 14 | 33014869 | T | C | -0.0912 | 0.0196 | 0.0978 | 3.27E-06 | rs3742926 | 14744 |
| MMP-10 | 19 | 49206145 | C | G | 0.1003 | 0.0117 | 0.4452 | 1.01E-17 | rs516316 | 14742 |
| MMP-10 | 10 | 13585421 | A | C | -0.2059 | 0.0444 | 0.9719 | 3.53E-06 | rs539147801 | 11329 |
| MMP-10 | 10 | 1063892 | A | G | -0.2185 | 0.0469 | 0.0185 | 3.18E-06 | rs55804377 | 14741 |
| MMP-10 | 9 | 6176871 | C | G | -0.0931 | 0.0166 | 0.8507 | 2.04E-08 | rs72699188 | 14744 |
| MMP-10 | 12 | 1.11E+08 | C | G | -0.0819 | 0.0178 | 0.1335 | 4.20E-06 | rs7966257 | 14295 |
| NRTN | 19 | 14691684 | A | C | -0.1731 | 0.0366 | 0.0363 | 2.25E-06 | rs10412462 | 11790 |
| NRTN | 20 | 55469957 | A | G | -0.2288 | 0.0495 | 0.0208 | 3.80E-06 | rs113580784 | 11768 |
| NRTN | 4 | 43313220 | T | C | -0.4361 | 0.0867 | 0.0118 | 4.91E-07 | rs143131035 | 7585 |
| NRTN | 11 | 10358788 | A | G | 0.1565 | 0.031 | 0.0646 | 4.46E-07 | rs182274799 | 9461 |
| NRTN | 8 | 43185781 | T | G | 0.3743 | 0.0786 | 0.0252 | 1.92E-06 | rs192427410 | 8783 |
| NRTN | 5 | 1.71E+08 | T | C | -0.074 | 0.0159 | 0.2493 | 3.25E-06 | rs29630 | 11343 |
| NRTN | 1 | 2.15E+08 | A | G | -0.0652 | 0.0141 | 0.3445 | 3.76E-06 | rs4360502 | 11342 |
| NRTN | 2 | 15734798 | A | G | 0.0786 | 0.0172 | 0.8077 | 4.88E-06 | rs62120726 | 11343 |
| NRTN | 16 | 26080883 | A | T | 0.0746 | 0.0157 | 0.6725 | 2.02E-06 | rs7202385 | 9460 |
| NRTN | 3 | 73067086 | T | C | 0.3387 | 0.0738 | 0.0176 | 4.44E-06 | rs76777800 | 6941 |
| NRTN | 5 | 21508959 | C | G | -0.0879 | 0.0176 | 0.6738 | 5.90E-07 | rs7712604 | 8587 |
| NRTN | 11 | 4475910 | T | C | 0.1667 | 0.034 | 0.958 | 9.44E-07 | rs77216559 | 11791 |
| NRTN | 15 | 24046105 | T | C | 0.1332 | 0.0286 | 0.9133 | 3.20E-06 | rs8033310 | 11343 |
| NRTN | 2 | 1.72E+08 | C | G | -0.0622 | 0.0136 | 0.3324 | 4.80E-06 | rs908372 | 11773 |
| NT-3 | 9 | 87478172 | T | G | 0.0557 | 0.012 | 0.5265 | 3.46E-06 | rs10780690 | 14296 |
| NT-3 | 18 | 72123860 | C | G | 0.0734 | 0.0124 | 0.5258 | 3.23E-09 | rs11151953 | 14296 |
| NT-3 | 11 | 1.02E+08 | A | C | -0.0973 | 0.0203 | 0.8737 | 1.64E-06 | rs11225042 | 12412 |
| NT-3 | 6 | 1.51E+08 | T | C | 0.1366 | 0.0263 | 0.0567 | 2.06E-07 | rs112418830 | 14296 |
| NT-3 | 5 | 1731054 | A | G | -0.0555 | 0.0121 | 0.3609 | 4.50E-06 | rs12517801 | 14726 |
| NT-3 | 2 | 46306174 | C | G | -0.2464 | 0.0539 | 0.0161 | 4.84E-06 | rs145686993 | 14257 |
| NT-3 | 10 | 1.16E+08 | T | C | -0.1859 | 0.0386 | 0.0249 | 1.46E-06 | rs146532158 | 14741 |
| NT-3 | 7 | 40026172 | A | C | -0.2571 | 0.0553 | 0.9866 | 3.33E-06 | rs17537796 | 14254 |
| NT-3 | 5 | 1.14E+08 | A | C | 0.0582 | 0.012 | 0.5677 | 1.23E-06 | rs2034246 | 14296 |
| NT-3 | 7 | 8762956 | A | G | -0.2393 | 0.0507 | 0.0175 | 2.36E-06 | rs2107473 | 13394 |
| NT-3 | 11 | 1.23E+08 | A | G | 0.0582 | 0.0125 | 0.3022 | 3.22E-06 | rs2846299 | 14721 |
| NT-3 | 15 | 88514855 | C | G | -0.1168 | 0.0164 | 0.1435 | 1.06E-12 | rs28735437 | 14737 |
| NT-3 | 6 | 1.12E+08 | A | G | 0.1364 | 0.0294 | 0.0429 | 3.49E-06 | rs3218587 | 14744 |
| NT-3 | 18 | 55310060 | C | G | -0.0819 | 0.0172 | 0.7735 | 1.92E-06 | rs376150779 | 10894 |
| NT-3 | 22 | 43649715 | T | C | 0.1823 | 0.0398 | 0.0287 | 4.64E-06 | rs5751454 | 13680 |
| NT-3 | 12 | 5680104 | A | T | 0.1106 | 0.022 | 0.0799 | 4.98E-07 | rs58399805 | 14737 |
| NT-3 | 8 | 72031901 | C | G | 0.058 | 0.0125 | 0.3811 | 3.48E-06 | rs72652073 | 14296 |
| NT-3 | 7 | 1.52E+08 | T | C | -0.092 | 0.02 | 0.1114 | 4.22E-06 | rs73166403 | 14296 |
| NT-3 | 4 | 7533099 | A | G | -0.1163 | 0.0251 | 0.0638 | 3.60E-06 | rs79759094 | 14728 |
| OPG | 14 | 69271784 | A | G | 0.057 | 0.0109 | 0.49 | 1.70E-07 | rs10142466 | 14721 |
| OPG | 14 | 94838142 | T | G | 0.2618 | 0.0433 | 0.0205 | 1.48E-09 | rs112635299 | 14733 |
| OPG | 1 | 27021913 | C | G | -0.1948 | 0.034 | 0.0337 | 1.01E-08 | rs114165349 | 14727 |
| OPG | 4 | 99986023 | C | G | -0.0671 | 0.0129 | 0.7288 | 1.98E-07 | rs1230164 | 14285 |
| OPG | 15 | 84250648 | A | G | -0.2007 | 0.0423 | 0.0206 | 2.09E-06 | rs138408096 | 14721 |
| OPG | 12 | 1.2E+08 | T | C | -0.2253 | 0.0473 | 0.0196 | 1.91E-06 | rs138557324 | 14285 |
| OPG | 11 | 16272199 | A | C | -0.079 | 0.0147 | 0.2072 | 7.69E-08 | rs1437633 | 12851 |
| OPG | 6 | 1.35E+08 | T | C | 0.3067 | 0.0628 | 0.0154 | 1.04E-06 | rs144471361 | 12145 |
| OPG | 3 | 1.72E+08 | T | C | -0.1569 | 0.0299 | 0.9631 | 1.54E-07 | rs17600346 | 14723 |
| OPG | 6 | 1.31E+08 | A | G | -0.1555 | 0.0334 | 0.962 | 3.23E-06 | rs192337992 | 11978 |
| OPG | 8 | 1.2E+08 | T | C | -0.1705 | 0.0114 | 0.5577 | 1.42E-50 | rs2247769 | 14285 |
| OPG | 17 | 47938521 | T | C | -0.0672 | 0.0129 | 0.748 | 1.90E-07 | rs271665 | 14710 |
| OPG | 4 | 32870458 | C | G | -0.2242 | 0.0486 | 0.9758 | 3.97E-06 | rs28454196 | 14285 |
| OPG | 12 | 71789031 | A | G | -0.2692 | 0.0568 | 0.0133 | 2.14E-06 | rs376613127 | 13154 |
| OPG | 1 | 40045085 | T | G | -0.0637 | 0.0139 | 0.3753 | 4.59E-06 | rs56009454 | 10894 |
| OPG | 13 | 1.1E+08 | A | G | -0.2671 | 0.0567 | 0.9843 | 2.47E-06 | rs61968587 | 13798 |
| OPG | 8 | 8604423 | A | C | 0.0522 | 0.0114 | 0.5556 | 4.67E-06 | rs6985207 | 14285 |
| OPG | 17 | 26694861 | A | G | -0.1404 | 0.0111 | 0.4726 | 1.14E-36 | rs704 | 14733 |
| OPG | 12 | 85607051 | T | C | -0.1471 | 0.0298 | 0.9603 | 7.96E-07 | rs74111611 | 14732 |
| OPG | 10 | 55029035 | A | G | 0.1965 | 0.0429 | 0.9791 | 4.64E-06 | rs77507094 | 14726 |
| OPG | 8 | 1.19E+08 | C | G | 0.0837 | 0.0146 | 0.4689 | 9.87E-09 | rs9643126 | 10893 |
| OPG | 18 | 76523646 | T | C | 0.172 | 0.0376 | 0.9707 | 4.77E-06 | rs9946923 | 13230 |
| OSM | 14 | 88422929 | T | C | -0.1077 | 0.0178 | 0.2079 | 1.44E-09 | rs111865607 | 10894 |
| OSM | 17 | 75178814 | A | G | -0.2566 | 0.0536 | 0.019 | 1.69E-06 | rs116928468 | 11770 |
| OSM | 9 | 1.38E+08 | C | G | 0.2926 | 0.0621 | 0.0172 | 2.46E-06 | rs117867445 | 10471 |
| OSM | 22 | 38211954 | A | G | -0.0663 | 0.0122 | 0.5586 | 5.50E-08 | rs2285179 | 14288 |
| OSM | 11 | 72945341 | T | C | 0.177 | 0.0236 | 0.9163 | 6.38E-14 | rs2511241 | 13668 |
| OSM | 22 | 49231540 | A | G | 0.0636 | 0.0136 | 0.7378 | 2.92E-06 | rs28444486 | 14735 |
| OSM | 17 | 38137033 | A | G | 0.1101 | 0.0117 | 0.5367 | 4.95E-21 | rs3859189 | 14729 |
| OSM | 22 | 30600105 | C | G | -0.159 | 0.0346 | 0.0363 | 4.32E-06 | rs4823082 | 14724 |
| OSM | 8 | 41555366 | T | C | -0.0594 | 0.0125 | 0.6496 | 2.01E-06 | rs559651 | 14715 |
| OSM | 11 | 47424864 | A | G | -0.0847 | 0.017 | 0.139 | 6.28E-07 | rs61897429 | 14735 |
| OSM | 12 | 1.12E+08 | T | C | -0.0728 | 0.0132 | 0.5224 | 3.48E-08 | rs653178 | 11785 |
| OSM | 8 | 2621859 | A | T | 0.1155 | 0.0243 | 0.9154 | 2.00E-06 | rs7007281 | 11775 |
| OSM | 11 | 1.02E+08 | T | C | -0.0803 | 0.0173 | 0.8631 | 3.46E-06 | rs7102957 | 14288 |
| OSM | 3 | 1.56E+08 | A | G | 0.0882 | 0.0167 | 0.5176 | 1.28E-07 | rs7432752 | 10894 |
| OSM | 10 | 30032133 | A | G | 0.2665 | 0.0525 | 0.0176 | 3.85E-07 | rs76275710 | 14735 |
| OSM | 21 | 42594678 | A | G | -0.152 | 0.0327 | 0.0371 | 3.35E-06 | rs80303768 | 14722 |
| OSM | 6 | 31274196 | T | C | -0.1048 | 0.0191 | 0.8812 | 4.09E-08 | rs9264931 | 14736 |
| PD-L1 | 5 | 1.6E+08 | T | C | -0.1179 | 0.0248 | 0.0613 | 1.99E-06 | rs10043030 | 14091 |
| PD-L1 | 8 | 68297799 | T | G | -0.1142 | 0.025 | 0.074 | 4.92E-06 | rs113674769 | 12404 |
| PD-L1 | 7 | 1.51E+08 | C | G | 0.1913 | 0.0407 | 0.966 | 2.60E-06 | rs12539643 | 11337 |
| PD-L1 | 1 | 27646512 | A | G | -0.4926 | 0.0993 | 0.0079 | 7.02E-07 | rs146328951 | 9354 |
| PD-L1 | 1 | 2.39E+08 | A | G | 0.0961 | 0.0192 | 0.868 | 5.58E-07 | rs184265227 | 14288 |
| PD-L1 | 15 | 89721461 | T | C | -0.0577 | 0.012 | 0.4351 | 1.52E-06 | rs2283438 | 14288 |
| PD-L1 | 5 | 18901704 | C | G | -0.1008 | 0.0219 | 0.0929 | 4.17E-06 | rs35024691 | 12825 |
| PD-L1 | 5 | 18901704 | C | G | -0.1008 | 0.0219 | 0.0929 | 4.17E-06 | rs35024691 | 12825 |
| PD-L1 | 3 | 1.27E+08 | A | G | -0.0816 | 0.0176 | 0.8717 | 3.55E-06 | rs4974444 | 14288 |
| PD-L1 | 9 | 5485387 | A | T | 0.1138 | 0.0213 | 0.916 | 9.16E-08 | rs60830384 | 14718 |
| PD-L1 | 5 | 90058982 | T | G | 0.1407 | 0.0273 | 0.9234 | 2.55E-07 | rs62373966 | 11777 |
| PD-L1 | 6 | 19842497 | T | C | 0.0554 | 0.0116 | 0.4457 | 1.79E-06 | rs6917605 | 14733 |
| PD-L1 | 9 | 34007646 | A | T | 0.0832 | 0.016 | 0.1779 | 1.99E-07 | rs7026097 | 12834 |
| PD-L1 | 11 | 1948186 | T | C | -0.0609 | 0.0128 | 0.6543 | 1.96E-06 | rs7125631 | 14288 |
| PD-L1 | 12 | 1.12E+08 | T | C | -0.0622 | 0.0133 | 0.5235 | 2.92E-06 | rs7137828 | 11337 |
| PD-L1 | 12 | 48670152 | T | C | 0.0537 | 0.0117 | 0.5862 | 4.44E-06 | rs7304709 | 14727 |
| PD-L1 | 6 | 1.35E+08 | A | G | 0.1933 | 0.0385 | 0.0292 | 5.15E-07 | rs75510105 | 14727 |
| PD-L1 | 19 | 5799008 | A | G | 0.1619 | 0.0324 | 0.0521 | 5.83E-07 | rs79045618 | 10714 |
| PD-L1 | 11 | 1.28E+08 | T | G | 0.061 | 0.0114 | 0.5018 | 8.75E-08 | rs7945680 | 14728 |
| PD-L1 | 9 | 5448218 | T | C | -0.1295 | 0.012 | 0.3471 | 3.77E-27 | rs822335 | 14736 |
| SCF | 11 | 13357183 | A | G | -0.0568 | 0.012 | 0.6474 | 2.21E-06 | rs10832027 | 14734 |
| SCF | 11 | 1.33E+08 | A | C | -0.0606 | 0.0129 | 0.3129 | 2.63E-06 | rs10894669 | 14735 |
| SCF | 2 | 71106068 | A | G | -0.0567 | 0.0116 | 0.4624 | 1.02E-06 | rs11126305 | 14718 |
| SCF | 10 | 1.23E+08 | T | C | 0.1548 | 0.0339 | 0.0334 | 4.96E-06 | rs11199541 | 14736 |
| SCF | 12 | 89369806 | T | C | -0.0707 | 0.0122 | 0.6366 | 6.83E-09 | rs11608458 | 14288 |
| SCF | 18 | 43867311 | A | G | 0.0538 | 0.0117 | 0.4265 | 4.26E-06 | rs11877350 | 14728 |
| SCF | 16 | 56993161 | A | G | 0.1146 | 0.0123 | 0.3094 | 1.20E-20 | rs12149545 | 14736 |
| SCF | 9 | 1.36E+08 | T | C | 0.105 | 0.0219 | 0.1215 | 1.63E-06 | rs12378537 | 11337 |
| SCF | 9 | 1.29E+08 | T | C | -0.1391 | 0.0205 | 0.0915 | 1.16E-11 | rs138854302 | 14736 |
| SCF | 22 | 25253027 | A | G | 0.1104 | 0.024 | 0.9366 | 4.22E-06 | rs139690 | 14734 |
| SCF | 20 | 44109198 | A | G | 0.2422 | 0.0516 | 0.0144 | 2.68E-06 | rs146276488 | 14728 |
| SCF | 17 | 16356498 | A | G | 0.2116 | 0.0453 | 0.0274 | 3.00E-06 | rs151246947 | 13224 |
| SCF | 16 | 67928042 | A | G | 0.1658 | 0.0171 | 0.1303 | 3.14E-22 | rs16942887 | 14736 |
| SCF | 11 | 61551356 | T | C | -0.0735 | 0.0121 | 0.6459 | 1.24E-09 | rs174535 | 14736 |
| SCF | 4 | 60174833 | A | G | -0.428 | 0.0907 | 0.9928 | 2.37E-06 | rs186248365 | 11538 |
| SCF | 20 | 44776543 | T | C | -0.1985 | 0.0341 | 0.0394 | 5.85E-09 | rs2024570 | 14288 |
| SCF | 8 | 47325114 | A | G | 0.4722 | 0.1032 | 0.007 | 4.75E-06 | rs2133027 | 8290 |
| SCF | 16 | 25205611 | A | T | -0.0715 | 0.0152 | 0.2479 | 2.55E-06 | rs2161465 | 11966 |
| SCF | 9 | 1.08E+08 | A | C | 0.1156 | 0.0133 | 0.7485 | 3.57E-18 | rs2740488 | 14732 |
| SCF | 8 | 1.27E+08 | T | C | -0.0683 | 0.0126 | 0.2937 | 5.94E-08 | rs2980888 | 14735 |
| SCF | 4 | 1.84E+08 | T | C | -0.1619 | 0.0339 | 0.0373 | 1.79E-06 | rs34813411 | 14717 |
| SCF | 15 | 43623397 | T | G | 0.2803 | 0.0512 | 0.9812 | 4.38E-08 | rs45617141 | 12935 |
| SCF | 1 | 2.35E+08 | A | C | -0.0553 | 0.0118 | 0.4647 | 2.78E-06 | rs557933 | 14287 |
| SCF | 20 | 44551855 | T | C | -0.2858 | 0.0144 | 0.8039 | 1.16E-87 | rs6073958 | 14730 |
| SCF | 7 | 94953895 | A | G | 0.0789 | 0.012 | 0.4598 | 4.87E-11 | rs705379 | 14288 |
| SCF | 13 | 26799167 | A | C | -0.0938 | 0.0192 | 0.1062 | 1.03E-06 | rs71431776 | 14714 |
| SCF | 1 | 2.23E+08 | A | G | -0.1584 | 0.0345 | 0.0354 | 4.40E-06 | rs71644719 | 14727 |
| SCF | 11 | 834389 | T | C | -0.0721 | 0.015 | 0.7926 | 1.53E-06 | rs72847212 | 13659 |
| SCF | 4 | 18944656 | T | C | -0.0718 | 0.0153 | 0.185 | 2.69E-06 | rs7664024 | 14288 |
| SCF | 1 | 88911632 | T | C | 0.1202 | 0.0233 | 0.9155 | 2.49E-07 | rs78397068 | 14288 |
| SCF | 9 | 79147122 | T | C | 0.0575 | 0.0118 | 0.4446 | 1.10E-06 | rs785900 | 14288 |
| SCF | 19 | 54793830 | C | G | 0.095 | 0.0141 | 0.2138 | 1.61E-11 | rs798893 | 14725 |
| SCF | 6 | 31270311 | A | G | 0.1407 | 0.0303 | 0.0716 | 3.42E-06 | rs9264796 | 12404 |
| SIRT2 | 6 | 1.58E+08 | A | G | 0.076 | 0.0145 | 0.6981 | 1.59E-07 | rs1034059 | 14288 |
| SIRT2 | 12 | 83600525 | A | C | -0.0841 | 0.0184 | 0.8864 | 4.86E-06 | rs11115639 | 14730 |
| SIRT2 | 5 | 78787853 | A | G | 0.1277 | 0.0277 | 0.9506 | 4.02E-06 | rs115455827 | 14735 |
| SIRT2 | 3 | 14613224 | T | C | -0.1521 | 0.0329 | 0.9617 | 3.78E-06 | rs116198125 | 14727 |
| SIRT2 | 22 | 45002809 | A | G | 0.0965 | 0.0208 | 0.885 | 3.49E-06 | rs12152184 | 12402 |
| SIRT2 | 7 | 14143486 | C | G | -0.1286 | 0.0243 | 0.9177 | 1.21E-07 | rs12531843 | 12404 |
| SIRT2 | 2 | 1.23E+08 | A | G | 0.104 | 0.0218 | 0.9138 | 1.84E-06 | rs138897138 | 14288 |
| SIRT2 | 19 | 39379770 | T | C | -0.5739 | 0.0515 | 0.0161 | 7.69E-29 | rs144373891 | 14736 |
| SIRT2 | 8 | 89590527 | T | C | 0.2573 | 0.0551 | 0.9858 | 3.02E-06 | rs147383134 | 13801 |
| SIRT2 | 4 | 1.61E+08 | A | G | -0.1063 | 0.0229 | 0.0886 | 3.45E-06 | rs60459634 | 14288 |
| SIRT2 | 6 | 63413043 | A | G | -0.3428 | 0.0737 | 0.989 | 3.30E-06 | rs72883370 | 12737 |
| SIRT2 | 5 | 1.78E+08 | A | T | 0.0639 | 0.0138 | 0.7496 | 3.65E-06 | rs7718478 | 14288 |
| SIRT2 | 13 | 37413465 | T | C | 0.0862 | 0.0184 | 0.8546 | 2.80E-06 | rs77381020 | 14288 |
| SLAMF1 | 3 | 1.87E+08 | A | T | 0.0727 | 0.0121 | 0.6151 | 1.88E-09 | rs10212190 | 14285 |
| SLAMF1 | 7 | 38626562 | T | C | 0.058 | 0.0125 | 0.3266 | 3.48E-06 | rs10499604 | 14730 |
| SLAMF1 | 12 | 1.21E+08 | T | G | 0.0569 | 0.0123 | 0.5251 | 3.73E-06 | rs11065264 | 12848 |
| SLAMF1 | 13 | 26736047 | T | C | 0.2712 | 0.0569 | 0.9863 | 1.88E-06 | rs111865527 | 14244 |
| SLAMF1 | 13 | 1.08E+08 | T | C | 0.2421 | 0.0506 | 0.9791 | 1.71E-06 | rs112742549 | 13499 |
| SLAMF1 | 1 | 1.61E+08 | A | C | -0.1256 | 0.0159 | 0.1616 | 2.80E-15 | rs12058421 | 14731 |
| SLAMF1 | 2 | 62575443 | A | G | -0.0612 | 0.0123 | 0.3695 | 6.50E-07 | rs13014154 | 14285 |
| SLAMF1 | 4 | 1.06E+08 | A | G | -0.0609 | 0.0127 | 0.5964 | 1.62E-06 | rs13147502 | 12843 |
| SLAMF1 | 10 | 66091335 | A | G | -0.2863 | 0.0577 | 0.0181 | 6.98E-07 | rs138657007 | 12790 |
| SLAMF1 | 14 | 87822926 | T | C | -0.0644 | 0.0128 | 0.3774 | 4.87E-07 | rs147616029 | 12850 |
| SLAMF1 | 18 | 59233474 | A | G | -0.3592 | 0.073 | 0.9888 | 8.63E-07 | rs149092077 | 11137 |
| SLAMF1 | 15 | 55294543 | T | G | -0.0557 | 0.012 | 0.5136 | 3.46E-06 | rs1609310 | 14286 |
| SLAMF1 | 9 | 78767497 | C | G | -0.1982 | 0.043 | 0.0213 | 4.04E-06 | rs17720330 | 14727 |
| SLAMF1 | 2 | 2.17E+08 | A | G | 0.3328 | 0.0655 | 0.989 | 3.76E-07 | rs192245879 | 13799 |
| SLAMF1 | 3 | 98605646 | A | C | 0.066 | 0.0132 | 0.4501 | 5.73E-07 | rs1972433 | 11333 |
| SLAMF1 | 6 | 1.53E+08 | T | C | 0.0688 | 0.0143 | 0.2204 | 1.50E-06 | rs2623960 | 14286 |
| SLAMF1 | 17 | 79220224 | C | G | 0.0894 | 0.0141 | 0.4462 | 2.29E-10 | rs2725405 | 10271 |
| SLAMF1 | 10 | 28873202 | T | C | -0.0747 | 0.0126 | 0.2977 | 3.06E-09 | rs2790462 | 14733 |
| SLAMF1 | 12 | 1.12E+08 | T | C | 0.097 | 0.013 | 0.4758 | 8.55E-14 | rs3184504 | 11783 |
| SLAMF1 | 18 | 72388527 | A | G | -0.1486 | 0.0324 | 0.0449 | 4.51E-06 | rs35271532 | 14286 |
| SLAMF1 | 19 | 17337555 | A | C | 0.0674 | 0.0145 | 0.2299 | 3.35E-06 | rs3745163 | 13669 |
| SLAMF1 | 4 | 6642331 | A | G | 0.0707 | 0.0153 | 0.1909 | 3.82E-06 | rs3806818 | 13853 |
| SLAMF1 | 17 | 7063899 | A | C | 0.1013 | 0.0156 | 0.2121 | 8.38E-11 | rs56244095 | 14286 |
| SLAMF1 | 5 | 95263427 | A | G | 0.1321 | 0.0164 | 0.2395 | 7.96E-16 | rs570025519 | 10894 |
| SLAMF1 | 1 | 1.61E+08 | T | C | 0.2209 | 0.0162 | 0.8506 | 2.45E-42 | rs60094514 | 14732 |
| SLAMF1 | 22 | 22451222 | T | C | 0.1063 | 0.0229 | 0.0805 | 3.45E-06 | rs73151926 | 14286 |
| SLAMF1 | 13 | 78282092 | C | G | -0.2791 | 0.0598 | 0.0148 | 3.05E-06 | rs73231041 | 12735 |
| SLAMF1 | 6 | 42462406 | T | C | -0.0589 | 0.0119 | 0.4969 | 7.44E-07 | rs7745783 | 14286 |
| SLAMF1 | 7 | 1.35E+08 | A | G | -0.1585 | 0.0295 | 0.0447 | 7.75E-08 | rs79348253 | 14719 |
| ST1A1 | 12 | 23759406 | T | C | -0.08 | 0.0173 | 0.8156 | 3.76E-06 | rs10842189 | 11784 |
| ST1A1 | 16 | 28285775 | A | G | 0.0695 | 0.0139 | 0.561 | 5.73E-07 | rs11074902 | 11345 |
| ST1A1 | 17 | 54851764 | A | G | 0.271 | 0.0581 | 0.9826 | 3.10E-06 | rs117222566 | 10403 |
| ST1A1 | 16 | 28561581 | T | C | -0.1882 | 0.0135 | 0.5314 | 3.58E-44 | rs149278 | 11344 |
| ST1A1 | 4 | 1.28E+08 | T | C | -0.3184 | 0.0684 | 0.9871 | 3.24E-06 | rs149869672 | 10858 |
| ST1A1 | 1 | 86468724 | T | C | -0.0984 | 0.0206 | 0.8775 | 1.78E-06 | rs1698742 | 11790 |
| ST1A1 | 8 | 17377551 | A | G | -0.1504 | 0.0318 | 0.95 | 2.25E-06 | rs2213907 | 11790 |
| ST1A1 | 12 | 1.13E+08 | T | C | 0.0622 | 0.0136 | 0.4128 | 4.80E-06 | rs232928 | 11345 |
| ST1A1 | 7 | 77960330 | T | C | -0.0645 | 0.0135 | 0.5986 | 1.77E-06 | rs2691531 | 11793 |
| ST1A1 | 1 | 1.19E+08 | A | G | 0.0702 | 0.0147 | 0.466 | 1.79E-06 | rs2712255 | 9849 |
| ST1A1 | 5 | 1.77E+08 | T | G | -0.083 | 0.0152 | 0.2653 | 4.75E-08 | rs2731674 | 11790 |
| ST1A1 | 4 | 1.42E+08 | A | C | 0.1048 | 0.0214 | 0.8462 | 9.72E-07 | rs371387526 | 8817 |
| ST1A1 | 6 | 52231674 | A | G | 0.0924 | 0.0199 | 0.8711 | 3.43E-06 | rs55793562 | 11781 |
| ST1A1 | 8 | 1.26E+08 | A | G | 0.0768 | 0.0166 | 0.4768 | 3.72E-06 | rs55939507 | 9460 |
| ST1A1 | 4 | 1.87E+08 | T | C | -0.1678 | 0.0136 | 0.4926 | 5.64E-35 | rs66530140 | 10913 |
| ST1A1 | 12 | 1.03E+08 | T | C | -0.0715 | 0.0147 | 0.2856 | 1.15E-06 | rs703595 | 11789 |
| ST1A1 | 3 | 1.86E+08 | T | C | -0.0712 | 0.0135 | 0.599 | 1.33E-07 | rs710446 | 11793 |
| ST1A1 | 21 | 28777897 | A | C | -0.18 | 0.0376 | 0.9587 | 1.69E-06 | rs72643498 | 11788 |
| ST1A1 | 12 | 12719921 | C | G | 0.1077 | 0.0224 | 0.8871 | 1.52E-06 | rs72656644 | 11345 |
| ST1A1 | 11 | 1.29E+08 | A | T | 0.134 | 0.0285 | 0.0644 | 2.58E-06 | rs76833948 | 11345 |
| ST1A1 | 11 | 19560616 | T | C | -0.1433 | 0.0287 | 0.9287 | 5.94E-07 | rs77919742 | 11345 |
| ST1A1 | 8 | 11410797 | T | C | -0.3483 | 0.0756 | 0.9898 | 4.08E-06 | rs7812322 | 10858 |
| ST1A1 | 12 | 74902158 | A | C | -0.1927 | 0.0361 | 0.0662 | 9.40E-08 | rs9804965 | 9265 |
| STAMPB | 6 | 1.58E+08 | A | G | 0.0695 | 0.0145 | 0.698 | 1.64E-06 | rs1034059 | 14288 |
| STAMPB | 19 | 56709656 | A | G | 0.2031 | 0.0437 | 0.0246 | 3.36E-06 | rs10426867 | 12785 |
| STAMPB | 14 | 1.04E+08 | A | G | -0.1722 | 0.0352 | 0.0323 | 9.98E-07 | rs111581172 | 14736 |
| STAMPB | 2 | 74061232 | A | G | 0.2195 | 0.0425 | 0.0229 | 2.41E-07 | rs111872246 | 14248 |
| STAMPB | 3 | 14613224 | T | C | -0.1484 | 0.0325 | 0.9619 | 4.97E-06 | rs116198125 | 14727 |
| STAMPB | 22 | 45002809 | A | G | 0.0961 | 0.0208 | 0.8851 | 3.83E-06 | rs12152184 | 12402 |
| STAMPB | 7 | 14143486 | C | G | -0.1251 | 0.0243 | 0.9177 | 2.63E-07 | rs12531843 | 12404 |
| STAMPB | 2 | 1.23E+08 | A | G | 0.1085 | 0.0218 | 0.9138 | 6.46E-07 | rs138897138 | 14288 |
| STAMPB | 19 | 6195636 | A | G | 0.1288 | 0.0272 | 0.0724 | 2.19E-06 | rs140656901 | 13224 |
| STAMPB | 15 | 81240669 | A | G | -0.1571 | 0.0309 | 0.0404 | 3.69E-07 | rs2280441 | 14735 |
| STAMPB | 17 | 60294757 | A | G | 0.235 | 0.0512 | 0.0269 | 4.44E-06 | rs73327656 | 13801 |
| STAMPB | 10 | 6800809 | T | C | -0.1908 | 0.0394 | 0.0247 | 1.28E-06 | rs75610895 | 14730 |
| TGF-alpha | 5 | 1.32E+08 | A | C | -0.0761 | 0.0166 | 0.7246 | 4.55E-06 | rs11242125 | 9453 |
| TGF-alpha | 11 | 1.2E+08 | T | C | 0.0959 | 0.019 | 0.1107 | 4.48E-07 | rs12270510 | 14731 |
| TGF-alpha | 19 | 58857883 | A | G | 0.2856 | 0.0569 | 0.9866 | 5.19E-07 | rs149146289 | 14285 |
| TGF-alpha | 8 | 2654153 | C | G | 0.1081 | 0.023 | 0.905 | 2.60E-06 | rs1963491 | 11334 |
| TGF-alpha | 4 | 16165036 | A | G | -0.2684 | 0.05 | 0.0153 | 7.96E-08 | rs34007466 | 14733 |
| TGF-alpha | 8 | 13724808 | C | G | 0.1678 | 0.0361 | 0.9681 | 3.35E-06 | rs34630685 | 14285 |
| TGF-alpha | 17 | 38137033 | A | G | 0.0655 | 0.0116 | 0.5369 | 1.64E-08 | rs3859189 | 14726 |
| TGF-alpha | 10 | 90836825 | A | G | -0.095 | 0.0198 | 0.1322 | 1.60E-06 | rs4934441 | 11780 |
| TGF-alpha | 12 | 1.12E+08 | T | C | -0.0708 | 0.0131 | 0.5228 | 6.50E-08 | rs653178 | 11782 |
| TGF-alpha | 2 | 70774295 | A | T | 0.137 | 0.0184 | 0.114 | 9.65E-14 | rs72912115 | 14728 |
| TGF-alpha | 10 | 30042437 | T | C | -0.2601 | 0.0508 | 0.9817 | 3.05E-07 | rs74130123 | 14733 |
| TGF-alpha | 19 | 15885492 | A | T | 0.143 | 0.0307 | 0.9552 | 3.19E-06 | rs78464136 | 14285 |
| TGF-alpha | 1 | 1.66E+08 | C | G | -0.2692 | 0.056 | 0.9835 | 1.53E-06 | rs78912149 | 13798 |
| TGF-alpha | 6 | 31034990 | T | C | 0.0717 | 0.0146 | 0.2343 | 9.06E-07 | rs9262670 | 12842 |
| TWEAK | 7 | 80365289 | A | G | -0.3062 | 0.0629 | 0.0173 | 1.13E-06 | rs117427219 | 10850 |
| TWEAK | 2 | 86307128 | A | G | -0.0649 | 0.013 | 0.478 | 5.97E-07 | rs12328100 | 11784 |
| TWEAK | 10 | 11469516 | A | G | -0.1207 | 0.0247 | 0.0885 | 1.03E-06 | rs12354434 | 11337 |
| TWEAK | 17 | 10503956 | A | T | 0.1372 | 0.0288 | 0.0615 | 1.90E-06 | rs1266163 | 11337 |
| TWEAK | 19 | 33635097 | C | G | -0.1127 | 0.0226 | 0.8939 | 6.14E-07 | rs12975019 | 10278 |
| TWEAK | 10 | 1.07E+08 | A | G | -0.0653 | 0.0142 | 0.2861 | 4.25E-06 | rs1377015 | 11782 |
| TWEAK | 6 | 1.27E+08 | A | G | 0.2512 | 0.0533 | 0.9796 | 2.44E-06 | rs138684734 | 10850 |
| TWEAK | 12 | 50824101 | T | C | 0.3405 | 0.066 | 0.0132 | 2.48E-07 | rs141295666 | 11337 |
| TWEAK | 8 | 1.37E+08 | A | G | -0.298 | 0.0646 | 0.9824 | 3.97E-06 | rs148560093 | 9984 |
| TWEAK | 2 | 1.18E+08 | A | T | 0.1289 | 0.0254 | 0.9163 | 3.88E-07 | rs333835 | 11337 |
| TWEAK | 4 | 1.47E+08 | T | C | -0.1517 | 0.031 | 0.9506 | 9.90E-07 | rs4835254 | 11770 |
| TWEAK | 4 | 1.27E+08 | T | C | 0.246 | 0.0472 | 0.0296 | 1.87E-07 | rs537908127 | 9243 |
| TWEAK | 20 | 2931738 | C | G | 0.51 | 0.1077 | 0.0088 | 2.19E-06 | rs6138961 | 7308 |
| TWEAK | 3 | 1.08E+08 | T | C | 0.0674 | 0.0135 | 0.4514 | 5.96E-07 | rs7621032 | 11337 |
| TWEAK | 7 | 1.5E+08 | T | C | -0.0606 | 0.0131 | 0.5366 | 3.73E-06 | rs7806921 | 11778 |
| TWEAK | 6 | 44325149 | T | C | 0.1175 | 0.0255 | 0.078 | 4.07E-06 | rs867063 | 11337 |
| TWEAK | 22 | 45932279 | T | C | 0.0826 | 0.0177 | 0.1679 | 3.06E-06 | rs9614693 | 11763 |
| TWEAK | 17 | 15740146 | T | C | -0.0836 | 0.0183 | 0.344 | 4.92E-06 | rs9913313 | 7957 |
| TNFB | 2 | 1.2E+08 | T | C | -0.1441 | 0.0309 | 0.0534 | 3.11E-06 | rs112317147 | 11344 |
| TNFB | 4 | 35497008 | T | C | 0.2324 | 0.0499 | 0.0237 | 3.20E-06 | rs113244922 | 10470 |
| TNFB | 5 | 21093728 | T | C | 0.2469 | 0.049 | 0.9743 | 4.69E-07 | rs114250919 | 11305 |
| TNFB | 1 | 1.1E+08 | C | G | -0.1918 | 0.0403 | 0.029 | 1.94E-06 | rs12743015 | 11784 |
| TNFB | 14 | 34880659 | A | G | -0.1252 | 0.0259 | 0.0934 | 1.34E-06 | rs12883833 | 9259 |
| TNFB | 11 | 72666063 | T | C | -0.3418 | 0.0735 | 0.0137 | 3.31E-06 | rs138471526 | 9983 |
| TNFB | 12 | 6429443 | A | G | -0.2485 | 0.0521 | 0.0294 | 1.85E-06 | rs139511528 | 10280 |
| TNFB | 13 | 47656376 | T | C | 0.2122 | 0.0451 | 0.9751 | 2.54E-06 | rs143019966 | 10913 |
| TNFB | 12 | 52132524 | A | G | 0.1732 | 0.035 | 0.0415 | 7.48E-07 | rs147423408 | 11344 |
| TNFB | 2 | 2.16E+08 | T | C | 0.1514 | 0.0317 | 0.0511 | 1.79E-06 | rs16852556 | 11344 |
| TNFB | 3 | 1.42E+08 | T | C | -0.3872 | 0.0842 | 0.0118 | 4.25E-06 | rs192303297 | 7843 |
| TNFB | 6 | 30137890 | T | C | 0.2308 | 0.0421 | 0.9618 | 4.20E-08 | rs2074475 | 11792 |
| TNFB | 12 | 6514963 | A | C | 0.1759 | 0.0189 | 0.1645 | 1.32E-20 | rs2364485 | 11344 |
| TNFB | 6 | 31042217 | T | C | -0.1991 | 0.0144 | 0.2883 | 1.77E-43 | rs2523882 | 11792 |
| TNFB | 6 | 31474685 | T | C | -0.6283 | 0.0196 | 0.116 | 1.00E-200 | rs2844500 | 11791 |
| TNFB | 15 | 1E+08 | T | C | -0.0626 | 0.0136 | 0.5824 | 4.17E-06 | rs28700215 | 11344 |
| TNFB | 13 | 23714434 | C | G | -0.229 | 0.0491 | 0.9731 | 3.10E-06 | rs537860389 | 9240 |
| TNFB | 6 | 31821910 | T | C | -0.3554 | 0.0467 | 0.0294 | 2.74E-14 | rs542608643 | 8387 |
| TNFB | 17 | 65264208 | A | G | 0.0748 | 0.0159 | 0.7583 | 2.55E-06 | rs55914821 | 11344 |
| TNFB | 6 | 29152190 | T | C | 0.2813 | 0.056 | 0.9837 | 5.08E-07 | rs72841573 | 11792 |
| TNFB | 12 | 1.12E+08 | C | G | 0.1356 | 0.0134 | 0.4758 | 4.53E-24 | rs7310615 | 11344 |
| TNFB | 7 | 46566236 | T | C | 0.2496 | 0.0532 | 0.0218 | 2.71E-06 | rs74363807 | 11344 |
| TNFB | 2 | 53931190 | A | G | 0.0748 | 0.0161 | 0.2098 | 3.38E-06 | rs7583622 | 11769 |
| TNFB | 5 | 1.68E+08 | A | G | -0.1741 | 0.0379 | 0.967 | 4.36E-06 | rs76913603 | 11782 |
| TNFB | 1 | 63459761 | T | C | -0.1917 | 0.041 | 0.9711 | 2.93E-06 | rs77897196 | 11792 |
| TNFRSF9 | 8 | 99918668 | C | G | -0.4015 | 0.0847 | 0.9895 | 2.13E-06 | rs10101219 | 9540 |
| TNFRSF9 | 2 | 8443803 | A | G | -0.0682 | 0.0144 | 0.3043 | 2.18E-06 | rs10178845 | 11765 |
| TNFRSF9 | 12 | 1.3E+08 | A | G | 0.064 | 0.0131 | 0.4447 | 1.03E-06 | rs10744409 | 11764 |
| TNFRSF9 | 10 | 90880926 | T | C | -0.0995 | 0.0212 | 0.1219 | 2.69E-06 | rs11202989 | 11336 |
| TNFRSF9 | 17 | 16522922 | A | G | 0.3284 | 0.0668 | 0.019 | 8.83E-07 | rs118083884 | 9785 |
| TNFRSF9 | 12 | 67215987 | A | G | -0.0669 | 0.014 | 0.3517 | 1.77E-06 | rs12227836 | 11336 |
| TNFRSF9 | 13 | 1.09E+08 | A | G | -0.1402 | 0.0296 | 0.9406 | 2.17E-06 | rs12874404 | 11336 |
| TNFRSF9 | 4 | 28155085 | T | G | 0.2317 | 0.0504 | 0.9796 | 4.28E-06 | rs140214797 | 11769 |
| TNFRSF9 | 13 | 58754621 | A | T | -0.3325 | 0.0719 | 0.9767 | 3.76E-06 | rs143274881 | 9353 |
| TNFRSF9 | 8 | 50188685 | A | G | 0.3742 | 0.0814 | 0.0105 | 4.29E-06 | rs146380867 | 10849 |
| TNFRSF9 | 3 | 1.78E+08 | A | G | 0.375 | 0.0809 | 0.0104 | 3.56E-06 | rs150949259 | 8674 |
| TNFRSF9 | 19 | 49126012 | C | G | 0.097 | 0.0206 | 0.8419 | 2.49E-06 | rs1645331 | 9033 |
| TNFRSF9 | 1 | 7972201 | A | G | -0.1301 | 0.013 | 0.5203 | 1.41E-23 | rs1776354 | 11783 |
| TNFRSF9 | 8 | 1.18E+08 | A | G | -0.1626 | 0.0313 | 0.9533 | 2.05E-07 | rs1846520 | 11782 |
| TNFRSF9 | 20 | 2629394 | A | T | 0.1189 | 0.0242 | 0.8742 | 8.96E-07 | rs192538756 | 9453 |
| TNFRSF9 | 2 | 1.2E+08 | A | C | -0.0968 | 0.0199 | 0.1322 | 1.15E-06 | rs2579649 | 11336 |
| TNFRSF9 | 6 | 31312538 | A | G | 0.0926 | 0.0149 | 0.2629 | 5.14E-10 | rs2854008 | 11784 |
| TNFRSF9 | 12 | 1.12E+08 | T | C | 0.0681 | 0.013 | 0.476 | 1.62E-07 | rs3184504 | 11784 |
| TNFRSF9 | 5 | 1.77E+08 | A | G | -0.0693 | 0.0139 | 0.6695 | 6.18E-07 | rs4976685 | 11763 |
| TNFRSF9 | 14 | 63713358 | C | G | -0.1155 | 0.0228 | 0.901 | 4.07E-07 | rs57700441 | 11773 |
| TNFRSF9 | 11 | 2632174 | A | T | -0.1438 | 0.0281 | 0.0736 | 3.10E-07 | rs72850206 | 11336 |
| TNFRSF9 | 4 | 1.54E+08 | A | C | 0.3111 | 0.065 | 0.9869 | 1.70E-06 | rs72964492 | 9983 |
| TNFRSF9 | 5 | 1.74E+08 | T | C | -0.0982 | 0.0203 | 0.1231 | 1.32E-06 | rs75246422 | 11784 |
| TNFRSF9 | 13 | 28604007 | T | C | -0.2714 | 0.0593 | 0.9828 | 4.72E-06 | rs76428106 | 10849 |
| TNFRSF9 | 5 | 1.24E+08 | A | G | 0.0924 | 0.0201 | 0.1947 | 4.29E-06 | rs7737635 | 9453 |
| TNFRSF9 | 9 | 87717237 | A | G | 0.0707 | 0.0152 | 0.7165 | 3.30E-06 | rs7858310 | 11336 |
| TNFRSF9 | 14 | 20709993 | C | G | 0.0889 | 0.0182 | 0.2828 | 1.04E-06 | rs8005108 | 9453 |
| TNFRSF9 | 12 | 6503786 | T | C | 0.0703 | 0.015 | 0.2853 | 2.78E-06 | rs9669611 | 11783 |
| TNFSF14 | 2 | 1.37E+08 | T | C | -0.0628 | 0.0137 | 0.6354 | 4.56E-06 | rs10211598 | 11770 |
| TNFSF14 | 19 | 5968688 | A | G | -0.0811 | 0.0176 | 0.1772 | 4.07E-06 | rs11085155 | 11774 |
| TNFSF14 | 12 | 91045724 | T | C | -0.0683 | 0.0147 | 0.3338 | 3.38E-06 | rs11105725 | 11345 |
| TNFSF14 | 19 | 6682873 | A | G | 0.2584 | 0.0327 | 0.0429 | 2.74E-15 | rs11569551 | 11793 |
| TNFSF14 | 6 | 56788897 | A | G | -0.2574 | 0.0506 | 0.0213 | 3.64E-07 | rs115950744 | 11302 |
| TNFSF14 | 7 | 28896826 | A | T | 0.1692 | 0.0364 | 0.9583 | 3.35E-06 | rs117442125 | 11345 |
| TNFSF14 | 8 | 36844946 | T | G | -0.1456 | 0.0303 | 0.0551 | 1.55E-06 | rs117720118 | 11773 |
| TNFSF14 | 5 | 1.32E+08 | A | C | 0.0785 | 0.0132 | 0.5494 | 2.73E-09 | rs11950562 | 11793 |
| TNFSF14 | 12 | 74925803 | T | C | -0.1519 | 0.0316 | 0.0635 | 1.53E-06 | rs1461044 | 11792 |
| TNFSF14 | 4 | 1.26E+08 | A | C | -0.2421 | 0.0498 | 0.0299 | 1.17E-06 | rs147119146 | 11788 |
| TNFSF14 | 15 | 23989218 | T | C | -0.6334 | 0.1109 | 0.0083 | 1.12E-08 | rs184708984 | 6942 |
| TNFSF14 | 16 | 78681855 | T | C | -0.063 | 0.0133 | 0.5474 | 2.17E-06 | rs2738591 | 11789 |
| TNFSF14 | 19 | 6665020 | T | C | -0.4894 | 0.0273 | 0.0625 | 7.29E-72 | rs344560 | 11793 |
| TNFSF14 | 18 | 14002436 | C | G | -0.0698 | 0.0152 | 0.2595 | 4.39E-06 | rs34925583 | 11345 |
| TNFSF14 | 13 | 1.01E+08 | T | C | 0.0619 | 0.0135 | 0.5736 | 4.54E-06 | rs4772260 | 11790 |
| TNFSF14 | 9 | 1.32E+08 | A | G | 0.229 | 0.0497 | 0.024 | 4.07E-06 | rs59801342 | 10721 |
| TNFSF14 | 15 | 79463978 | T | C | -0.2255 | 0.0472 | 0.0287 | 1.77E-06 | rs74600570 | 11778 |
| TNFSF14 | 19 | 29938464 | T | C | 0.1032 | 0.0215 | 0.8112 | 1.59E-06 | rs76688046 | 7942 |
| TNFSF14 | 5 | 39861942 | C | G | -0.2117 | 0.043 | 0.0273 | 8.51E-07 | rs76778362 | 11791 |
| TNFSF14 | 4 | 94769686 | A | G | -0.0818 | 0.0166 | 0.2019 | 8.32E-07 | rs7688836 | 11791 |
| TNFSF14 | 8 | 27416526 | A | C | 0.2392 | 0.0522 | 0.0209 | 4.60E-06 | rs78336300 | 10917 |
| TNFSF14 | 8 | 99636603 | A | G | -0.2358 | 0.0506 | 0.023 | 3.16E-06 | rs79233212 | 11345 |
| TNFSF14 | 10 | 1.11E+08 | T | C | -0.2336 | 0.0511 | 0.9787 | 4.84E-06 | rs79791117 | 10903 |
| TNFSF14 | 6 | 22713651 | T | C | -0.0651 | 0.0141 | 0.3144 | 3.89E-06 | rs9295562 | 11771 |
| TNFSF14 | 18 | 11626563 | A | G | 0.1048 | 0.0208 | 0.1883 | 4.69E-07 | rs9961731 | 9461 |
| TNFSF14 | 18 | 70105531 | T | C | -0.0905 | 0.0186 | 0.1483 | 1.14E-06 | rs9962377 | 11793 |
| TRAIL | 6 | 90047013 | A | G | 0.0655 | 0.0118 | 0.4536 | 2.84E-08 | rs1065657 | 14287 |
| TRAIL | 12 | 1.04E+08 | A | T | -0.0881 | 0.0185 | 0.8739 | 1.92E-06 | rs10745981 | 12850 |
| TRAIL | 5 | 1.63E+08 | A | G | 0.189 | 0.037 | 0.9721 | 3.25E-07 | rs111257277 | 14729 |
| TRAIL | 5 | 75017740 | T | C | -0.26 | 0.0564 | 0.0161 | 4.03E-06 | rs114597610 | 14735 |
| TRAIL | 6 | 33692744 | A | C | 0.1342 | 0.0265 | 0.063 | 4.10E-07 | rs140862652 | 14287 |
| TRAIL | 6 | 33692744 | A | C | 0.1342 | 0.0265 | 0.063 | 4.10E-07 | rs140862652 | 14287 |
| TRAIL | 7 | 1.36E+08 | A | G | -0.5024 | 0.1076 | 0.0051 | 3.02E-06 | rs141011299 | 9812 |
| TRAIL | 4 | 67749461 | C | G | -0.2835 | 0.0606 | 0.0106 | 2.89E-06 | rs142889372 | 14238 |
| TRAIL | 4 | 69327121 | T | G | 0.2641 | 0.0535 | 0.019 | 7.96E-07 | rs145386370 | 13386 |
| TRAIL | 4 | 57703725 | T | C | 0.1622 | 0.0347 | 0.0376 | 2.95E-06 | rs146922303 | 14727 |
| TRAIL | 1 | 1.97E+08 | T | C | -0.1191 | 0.0147 | 0.8073 | 5.40E-16 | rs16840522 | 14725 |
| TRAIL | 11 | 61549025 | A | G | 0.0787 | 0.0121 | 0.3509 | 7.81E-11 | rs174533 | 14732 |
| TRAIL | 4 | 1.81E+08 | T | G | 0.0674 | 0.0138 | 0.7733 | 1.04E-06 | rs17779404 | 14723 |
| TRAIL | 11 | 1.03E+08 | A | G | -0.244 | 0.0491 | 0.0227 | 6.71E-07 | rs180941492 | 13800 |
| TRAIL | 6 | 25887657 | A | G | -0.0756 | 0.0158 | 0.1589 | 1.71E-06 | rs1892247 | 14719 |
| TRAIL | 16 | 57345733 | T | C | 0.0691 | 0.0145 | 0.2543 | 1.88E-06 | rs2113173 | 14287 |
| TRAIL | 10 | 1.08E+08 | T | G | -0.1452 | 0.0316 | 0.0401 | 4.33E-06 | rs2418781 | 14287 |
| TRAIL | 3 | 1.14E+08 | A | T | -0.1311 | 0.0279 | 0.9501 | 2.62E-06 | rs28424593 | 14722 |
| TRAIL | 14 | 94844947 | T | C | 0.6692 | 0.044 | 0.0205 | 3.08E-52 | rs28929474 | 14735 |
| TRAIL | 19 | 44153100 | A | G | 0.3554 | 0.0163 | 0.8425 | 2.14E-105 | rs4760 | 14287 |
| TRAIL | 3 | 1.72E+08 | T | G | 0.1252 | 0.02 | 0.1051 | 3.85E-10 | rs490342 | 13228 |
| TRAIL | 3 | 1.86E+08 | A | G | 0.285 | 0.0187 | 0.886 | 1.90E-52 | rs5030044 | 14287 |
| TRAIL | 3 | 1.72E+08 | A | C | 0.5818 | 0.0399 | 0.9715 | 3.69E-48 | rs574044675 | 13173 |
| TRAIL | 18 | 29804863 | A | T | -0.0894 | 0.0115 | 0.4593 | 7.61E-15 | rs654488 | 14735 |
| TRAIL | 7 | 52792820 | T | C | 0.1823 | 0.038 | 0.9624 | 1.61E-06 | rs6969337 | 11336 |
| TRAIL | 16 | 72079657 | T | C | -0.0724 | 0.0146 | 0.1946 | 7.09E-07 | rs77303550 | 14728 |
| TRAIL | 17 | 64224775 | T | C | 0.2575 | 0.037 | 0.029 | 3.42E-12 | rs8178824 | 14735 |
| TRANCE | 2 | 30655453 | A | C | -0.1939 | 0.0416 | 0.976 | 3.15E-06 | rs113835939 | 14732 |
| TRANCE | 20 | 39165692 | A | G | -0.1646 | 0.03 | 0.0498 | 4.10E-08 | rs117113213 | 14723 |
| TRANCE | 3 | 1.94E+08 | A | G | -0.0978 | 0.015 | 0.2943 | 7.03E-11 | rs11713634 | 11336 |
| TRANCE | 6 | 1.21E+08 | T | C | -0.2296 | 0.0503 | 0.9805 | 5.00E-06 | rs1273754 | 10850 |
| TRANCE | 9 | 93148579 | T | C | 0.07 | 0.0148 | 0.8063 | 2.25E-06 | rs1426506 | 14729 |
| TRANCE | 5 | 1.6E+08 | T | C | -0.0549 | 0.0118 | 0.4357 | 3.28E-06 | rs1432791 | 14288 |
| TRANCE | 5 | 1.16E+08 | A | G | 0.2386 | 0.0459 | 0.9763 | 2.01E-07 | rs150128402 | 14736 |
| TRANCE | 19 | 35131252 | C | G | 0.1462 | 0.0315 | 0.0491 | 3.46E-06 | rs150416959 | 13224 |
| TRANCE | 6 | 33048371 | C | G | 0.2646 | 0.0498 | 0.018 | 1.08E-07 | rs17221073 | 13801 |
| TRANCE | 8 | 83420230 | C | G | -0.1254 | 0.0272 | 0.9313 | 4.02E-06 | rs183217699 | 12852 |
| TRANCE | 8 | 1.07E+08 | T | G | -0.0678 | 0.0143 | 0.6393 | 2.12E-06 | rs1839438 | 10894 |
| TRANCE | 2 | 1.36E+08 | A | C | 0.1377 | 0.0285 | 0.9227 | 1.35E-06 | rs201831835 | 10894 |
| TRANCE | 8 | 1.2E+08 | T | C | -0.1618 | 0.0118 | 0.4162 | 8.62E-43 | rs2468187 | 14735 |
| TRANCE | 8 | 19490180 | T | G | -0.078 | 0.017 | 0.8529 | 4.47E-06 | rs2975421 | 14288 |
| TRANCE | 1 | 22143382 | A | G | 0.167 | 0.0344 | 0.0349 | 1.21E-06 | rs34221978 | 14734 |
| TRANCE | 7 | 50811081 | A | G | 0.0686 | 0.013 | 0.2872 | 1.31E-07 | rs3801000 | 14288 |
| TRANCE | 13 | 68202750 | T | C | -0.1684 | 0.0356 | 0.0319 | 2.24E-06 | rs4334157 | 14736 |
| TRANCE | 4 | 1.06E+08 | A | G | -0.0777 | 0.0166 | 0.1504 | 2.86E-06 | rs4365726 | 14287 |
| TRANCE | 13 | 43039673 | A | C | 0.1172 | 0.0116 | 0.4634 | 5.33E-24 | rs4512994 | 14736 |
| TRANCE | 9 | 16905616 | A | T | 0.1215 | 0.0265 | 0.0705 | 4.54E-06 | rs5001566 | 11982 |
| TRANCE | 20 | 46454945 | T | C | 0.0636 | 0.0126 | 0.3364 | 4.47E-07 | rs6063153 | 14287 |
| TRANCE | 8 | 23089208 | A | G | -0.1532 | 0.0148 | 0.2051 | 4.13E-25 | rs62501135 | 14288 |
| TRANCE | 6 | 90249507 | A | G | 0.054 | 0.0116 | 0.5245 | 3.24E-06 | rs6932751 | 14730 |
| TRANCE | 17 | 26694861 | A | G | -0.1085 | 0.0116 | 0.4755 | 8.48E-21 | rs704 | 14736 |
| TRANCE | 2 | 17337409 | T | G | 0.2368 | 0.0486 | 0.9817 | 1.10E-06 | rs72781813 | 14735 |
| TRANCE | 12 | 1.29E+08 | T | G | -0.2685 | 0.0539 | 0.9818 | 6.31E-07 | rs75991751 | 13422 |
| TRANCE | 2 | 1.04E+08 | T | G | -0.1719 | 0.0366 | 0.9693 | 2.64E-06 | rs76488966 | 14720 |
| TRANCE | 3 | 83133339 | T | C | -0.2976 | 0.0641 | 0.9853 | 3.44E-06 | rs77980965 | 11337 |
| TRANCE | 3 | 1.72E+08 | A | G | -0.4987 | 0.0343 | 0.0334 | 6.82E-48 | rs79287178 | 14719 |
| TSLP | 9 | 23277990 | A | T | -0.0934 | 0.0201 | 0.1258 | 3.37E-06 | rs112206094 | 11792 |
| TSLP | 10 | 34395970 | A | G | -0.1569 | 0.0338 | 0.0499 | 3.45E-06 | rs117095006 | 11345 |
| TSLP | 17 | 27504563 | A | G | -0.4423 | 0.0943 | 0.0096 | 2.73E-06 | rs118119134 | 8576 |
| TSLP | 16 | 8805947 | T | G | 0.1784 | 0.0367 | 0.0353 | 1.17E-06 | rs12448125 | 11792 |
| TSLP | 2 | 2626966 | C | G | -0.074 | 0.0153 | 0.758 | 1.32E-06 | rs12472785 | 11792 |
| TSLP | 15 | 79915029 | A | G | 0.1619 | 0.0351 | 0.0381 | 3.98E-06 | rs140886020 | 11786 |
| TSLP | 11 | 1.22E+08 | A | G | 0.2674 | 0.0575 | 0.9812 | 3.31E-06 | rs145111430 | 11345 |
| TSLP | 20 | 37959633 | A | G | 0.3618 | 0.0747 | 0.9877 | 1.28E-06 | rs185922111 | 10858 |
| TSLP | 15 | 60209215 | A | C | 0.3219 | 0.066 | 0.0132 | 1.08E-06 | rs190906347 | 10858 |
| TSLP | 7 | 42730058 | T | G | 0.0846 | 0.0152 | 0.2624 | 2.61E-08 | rs4724126 | 11344 |
| TSLP | 8 | 69813968 | A | G | 0.1629 | 0.0345 | 0.9535 | 2.34E-06 | rs72652144 | 11782 |
| TSLP | 10 | 1.09E+08 | T | C | -0.2956 | 0.063 | 0.0141 | 2.70E-06 | rs72811179 | 11345 |
| TSLP | 17 | 80919387 | A | G | -0.0643 | 0.0139 | 0.4553 | 3.73E-06 | rs7406537 | 11149 |
| TSLP | 7 | 1.49E+08 | A | T | 0.0743 | 0.0158 | 0.632 | 2.57E-06 | rs75129390 | 8817 |
| TSLP | 1 | 2.41E+08 | C | G | -0.0928 | 0.0187 | 0.8143 | 6.96E-07 | rs7550374 | 11345 |
| TNF | 11 | 11644391 | A | G | 0.0692 | 0.0125 | 0.3558 | 3.09E-08 | rs10831650 | 14288 |
| TNF | 8 | 1.45E+08 | T | C | 0.0613 | 0.0122 | 0.388 | 5.05E-07 | rs11136339 | 13664 |
| TNF | 10 | 51775971 | C | G | -0.401 | 0.0871 | 0.9859 | 4.15E-06 | rs111641307 | 10446 |
| TNF | 5 | 1.67E+08 | T | C | 0.0577 | 0.0119 | 0.4339 | 1.24E-06 | rs11738159 | 14288 |
| TNF | 12 | 82487578 | C | G | -0.1855 | 0.0377 | 0.9706 | 8.64E-07 | rs117550445 | 14721 |
| TNF | 1 | 1.63E+08 | A | T | -0.0727 | 0.0141 | 0.2484 | 2.52E-07 | rs12145644 | 14287 |
| TNF | 1 | 2.34E+08 | A | G | 0.109 | 0.0228 | 0.0734 | 1.75E-06 | rs12726054 | 14721 |
| TNF | 4 | 1.03E+08 | T | C | 0.2027 | 0.0236 | 0.0696 | 8.77E-18 | rs13107325 | 14733 |
| TNF | 6 | 88516736 | A | G | -0.2027 | 0.0438 | 0.9577 | 3.69E-06 | rs16879825 | 11784 |
| TNF | 3 | 1.43E+08 | A | G | -0.1312 | 0.028 | 0.9544 | 2.79E-06 | rs1965399 | 14736 |
| TNF | 2 | 99371601 | A | G | -0.0849 | 0.0168 | 0.8184 | 4.34E-07 | rs201620545 | 11327 |
| TNF | 2 | 53077754 | A | G | 0.1404 | 0.0273 | 0.9522 | 2.71E-07 | rs2163333 | 14719 |
| TNF | 1 | 2.21E+08 | A | G | -0.0591 | 0.0129 | 0.6653 | 4.62E-06 | rs2738752 | 12848 |
| TNF | 17 | 7451110 | T | C | 0.211 | 0.0129 | 0.2563 | 3.90E-60 | rs34790908 | 14732 |
| TNF | 3 | 59625877 | A | G | -0.0622 | 0.0136 | 0.2327 | 4.80E-06 | rs3821473 | 14736 |
| TNF | 12 | 1.29E+08 | T | C | 0.0666 | 0.0131 | 0.3457 | 3.70E-07 | rs4882710 | 14288 |
| TNF | 14 | 55351508 | A | C | 0.1015 | 0.0218 | 0.1166 | 3.22E-06 | rs552941568 | 10894 |
| TNF | 9 | 5636355 | T | C | -0.0856 | 0.0186 | 0.8764 | 4.18E-06 | rs56023600 | 14287 |
| TNF | 6 | 70682566 | T | C | -0.2262 | 0.0471 | 0.025 | 1.57E-06 | rs575752535 | 10894 |
| TNF | 9 | 1.36E+08 | T | C | 0.1098 | 0.0157 | 0.7888 | 2.68E-12 | rs579459 | 11785 |
| TNF | 11 | 1.31E+08 | A | G | -0.0717 | 0.0154 | 0.1931 | 3.23E-06 | rs58743186 | 12818 |
| TNF | 9 | 33113322 | T | C | -0.0897 | 0.0193 | 0.1102 | 3.36E-06 | rs7019909 | 14288 |
| TNF | 3 | 98429219 | C | G | 0.0812 | 0.012 | 0.4558 | 1.32E-11 | rs73133996 | 14288 |
| TNF | 12 | 592399 | T | C | -0.0744 | 0.0161 | 0.1517 | 3.82E-06 | rs73590361 | 14726 |
| TNF | 17 | 7789542 | A | G | -0.1386 | 0.0238 | 0.0779 | 5.76E-09 | rs7503751 | 14288 |
| TNF | 10 | 1.33E+08 | T | C | 0.1318 | 0.0286 | 0.0482 | 4.06E-06 | rs76047473 | 14718 |
| TNF | 19 | 19706214 | A | C | 0.1896 | 0.0369 | 0.0315 | 2.77E-07 | rs76133364 | 13224 |
| TNF | 3 | 1.74E+08 | T | C | -0.2108 | 0.0456 | 0.9813 | 3.79E-06 | rs78254232 | 14248 |
| TNF | 2 | 1.09E+08 | A | T | -0.0592 | 0.0125 | 0.3965 | 2.18E-06 | rs826688 | 12835 |
| TNF | 3 | 1.43E+08 | C | G | 0.1192 | 0.014 | 0.748 | 1.68E-17 | rs9842051 | 14288 |
| uPA | 4 | 35016704 | A | G | -0.0548 | 0.0119 | 0.5246 | 4.12E-06 | rs1074669 | 14286 |
| uPA | 11 | 11142087 | A | G | 0.0784 | 0.0154 | 0.1671 | 3.56E-07 | rs10840566 | 14733 |
| uPA | 9 | 94784966 | A | G | 0.1061 | 0.0217 | 0.1034 | 1.01E-06 | rs10992204 | 14286 |
| uPA | 11 | 1.26E+08 | A | G | -0.1024 | 0.0161 | 0.1509 | 2.01E-10 | rs11220462 | 14731 |
| uPA | 9 | 1.36E+08 | A | G | 0.1 | 0.017 | 0.1744 | 4.04E-09 | rs13299342 | 11781 |
| uPA | 12 | 1.06E+08 | C | G | 0.3395 | 0.0656 | 0.0119 | 2.28E-07 | rs144470316 | 12303 |
| uPA | 6 | 32900651 | A | G | -0.0625 | 0.012 | 0.6344 | 1.91E-07 | rs154972 | 14724 |
| uPA | 1 | 1.52E+08 | T | C | 0.2771 | 0.0607 | 0.9835 | 4.99E-06 | rs202063542 | 10407 |
| uPA | 10 | 75677840 | A | C | -0.0946 | 0.0115 | 0.5624 | 1.93E-16 | rs2227575 | 14729 |
| uPA | 1 | 1.1E+08 | A | G | -0.0785 | 0.0166 | 0.1578 | 2.26E-06 | rs333947 | 14286 |
| uPA | 10 | 75424174 | A | G | 0.3099 | 0.0664 | 0.0145 | 3.05E-06 | rs3933086 | 11778 |
| uPA | 19 | 44174441 | T | C | -0.422 | 0.0311 | 0.0405 | 6.10E-42 | rs4251805 | 14734 |
| uPA | 19 | 44153100 | A | G | 0.1408 | 0.0165 | 0.8426 | 1.42E-17 | rs4760 | 14286 |
| uPA | 18 | 24686365 | T | C | -0.094 | 0.0125 | 0.3008 | 5.48E-14 | rs4800787 | 14730 |
| uPA | 7 | 29499619 | C | G | 0.2093 | 0.0456 | 0.0329 | 4.43E-06 | rs577588918 | 10894 |
| uPA | 5 | 1.27E+08 | T | G | 0.0627 | 0.0133 | 0.7552 | 2.43E-06 | rs59772299 | 14730 |
| uPA | 12 | 1.12E+08 | A | G | 0.0662 | 0.0134 | 0.478 | 7.80E-07 | rs597808 | 11334 |
| uPA | 3 | 1.94E+08 | T | C | -0.091 | 0.019 | 0.8498 | 1.67E-06 | rs62286554 | 11335 |
| uPA | 3 | 56631258 | A | G | 0.0952 | 0.0176 | 0.1456 | 6.33E-08 | rs6805474 | 14285 |
| uPA | 17 | 26694861 | A | G | 0.0567 | 0.0115 | 0.4758 | 8.20E-07 | rs704 | 14734 |
| uPA | 8 | 1.4E+08 | C | G | 0.1968 | 0.0417 | 0.0253 | 2.37E-06 | rs72727847 | 14729 |
| uPA | 17 | 7063667 | T | C | -0.1107 | 0.0156 | 0.7855 | 1.28E-12 | rs7406661 | 14286 |
| uPA | 4 | 1.31E+08 | A | G | -0.1817 | 0.0389 | 0.0248 | 3.00E-06 | rs74607099 | 14723 |
| uPA | 2 | 1.61E+08 | A | G | -0.0852 | 0.0114 | 0.5375 | 7.80E-14 | rs7564243 | 14729 |
| VEGF_A | 10 | 65071215 | A | C | 0.0838 | 0.0117 | 0.4155 | 7.93E-13 | rs10822155 | 14744 |
| VEGF_A | 11 | 2028103 | C | G | -0.0683 | 0.0142 | 0.7087 | 1.51E-06 | rs10840177 | 13231 |
| VEGF_A | 5 | 88180196 | T | C | -0.1625 | 0.0267 | 0.9462 | 1.16E-09 | rs114694170 | 14728 |
| VEGF_A | 8 | 73592332 | C | G | -0.2163 | 0.0449 | 0.9739 | 1.45E-06 | rs117976551 | 13809 |
| VEGF_A | 5 | 1.17E+08 | T | C | -0.1842 | 0.0374 | 0.028 | 8.43E-07 | rs11953790 | 14730 |
| VEGF_A | 16 | 231541 | T | C | 0.1174 | 0.0253 | 0.069 | 3.48E-06 | rs1203834 | 13232 |
| VEGF_A | 7 | 1.44E+08 | T | C | -0.079 | 0.0166 | 0.8309 | 1.95E-06 | rs1208181 | 12855 |
| VEGF_A | 10 | 1.04E+08 | A | G | -0.3266 | 0.0715 | 0.0091 | 4.93E-06 | rs12784822 | 14296 |
| VEGF_A | 13 | 29335624 | C | G | 0.1981 | 0.0397 | 0.0248 | 6.04E-07 | rs138648222 | 14739 |
| VEGF_A | 13 | 77462624 | A | C | -0.2779 | 0.0603 | 0.9871 | 4.05E-06 | rs144037665 | 11969 |
| VEGF_A | 9 | 3020960 | T | C | -0.2475 | 0.0539 | 0.0153 | 4.39E-06 | rs145703410 | 14296 |
| VEGF_A | 12 | 75261276 | T | C | 0.2736 | 0.0582 | 0.0162 | 2.59E-06 | rs145846107 | 13422 |
| VEGF_A | 5 | 54043585 | T | C | -0.22 | 0.0457 | 0.9797 | 1.48E-06 | rs16883415 | 14744 |
| VEGF_A | 1 | 32204683 | A | G | -0.0734 | 0.0152 | 0.8126 | 1.37E-06 | rs2050256 | 14739 |
| VEGF_A | 11 | 95274487 | T | G | 0.0992 | 0.0208 | 0.9024 | 1.85E-06 | rs34536806 | 14296 |
| VEGF_A | 19 | 14762156 | A | C | 0.0671 | 0.0127 | 0.704 | 1.27E-07 | rs34562990 | 14295 |
| VEGF_A | 6 | 43924830 | T | G | 0.4259 | 0.0113 | 0.5288 | 1.00E-200 | rs4349809 | 14734 |
| VEGF_A | 8 | 5413008 | C | G | -0.0741 | 0.0158 | 0.8345 | 2.73E-06 | rs4875579 | 14296 |
| VEGF_A | 1 | 1.9E+08 | T | C | -0.0759 | 0.0148 | 0.8114 | 2.92E-07 | rs61818787 | 14732 |
| VEGF_A | 6 | 43855489 | A | C | -0.2112 | 0.0333 | 0.0429 | 2.26E-10 | rs62401205 | 14296 |
| VEGF_A | 9 | 2687795 | A | T | 0.1344 | 0.0125 | 0.542 | 5.80E-27 | rs6475938 | 12412 |
| VEGF_A | 12 | 46909886 | C | G | -0.1288 | 0.0265 | 0.9341 | 1.17E-06 | rs6582666 | 11791 |
| VEGF_A | 8 | 1.07E+08 | A | T | 0.1231 | 0.0134 | 0.7319 | 4.06E-20 | rs6993770 | 14296 |
| VEGF_A | 4 | 94769686 | A | G | -0.0685 | 0.0145 | 0.2065 | 2.31E-06 | rs7688836 | 14742 |
| VEGF_A | 7 | 68565319 | A | C | -0.0628 | 0.0137 | 0.6119 | 4.56E-06 | rs7808444 | 14296 |
| VEGF_A | 6 | 43940269 | A | G | -0.0602 | 0.0124 | 0.5217 | 1.20E-06 | rs9472184 | 14296 |

**Supplementary Table 3.** STROBE-MR checklist

STROBE-MR checklist of recommended items to address in reports of Mendelian randomization studies^1^ ^2^

| **Item No.** | **Section** | **Checklist item** | **Note/ Page No.** |
| --- | --- | --- | --- |
| 1 | **TITLE and ABSTRACT** | Indicate Mendelian randomization (MR) as the study’s design in the title and/or the abstract if that is a main purpose of the study | ‘Mendelian randomization’ in title and abstract |
|  | **INTRODUCTION** |  |  |
| 2 | **Background** | Explain the scientific background and rationale for the reported study. What is the exposure? Is a potential causal relationship between exposure and outcome plausible? Justify why MR is a helpful method to address the study question | Page 1 |
| 3 | **Objectives** | State specific objectives clearly, including pre-specified causal hypotheses (if any). State that MR is a method that, under specific assumptions, intends to estimate causal effects | Page 1 |
|  | **METHODS** |  |  |
| 4 | **Study design and data sources** | Present key elements of the study design early in the article. Consider including a table listing sources of data for all phases of the study. For each data source contributing to the analysis, describe the following: | Page 1-3, Supplementary Table, |
|  | a) | Setting: Describe the study design and the underlying population, if possible. Describe the setting, locations, and relevant dates, including periods of recruitment, exposure, follow-up, and data collection, when available. |  |
|  | b) | Participants: Give the eligibility criteria, and the sources and methods of selection of participants. Report the sample size, and whether any power or sample size calculations were carried out prior to the main analysis |  |
|  | c) | Describe measurement, quality control and selection of genetic variants |  |
|  | d) | For each exposure, outcome, and other relevant variables, describe methods of assessment and diagnostic criteria for diseases |  |
|  | e) | Provide details of ethics committee approval and participant informed consent, if relevant |  |
| 5 | **Assumptions** | Explicitly state the three core IV assumptions for the main analysis (relevance, independence and exclusion restriction) as well assumptions for any additional or sensitivity analysis | Page 4, Fig 1 |
| 6 | **Statistical methods: main analysis** | Describe statistical methods and statistics used | Page 3-4 |
|  | a) | Describe how quantitative variables were handled in the analyses (i.e., scale, units, model) |  |
|  | b) | Describe how genetic variants were handled in the analyses and, if applicable, how their weights were selected |  |
|  | c) | Describe the MR estimator (e.g. two-stage least squares, Wald ratio) and related statistics. Detail the included covariates and, in case of two-sample MR, whether the same covariate set was used for adjustment in the two samples |  |
|  | d) | Explain how missing data were addressed |  |
|  | e) | If applicable, indicate how multiple testing was addressed |  |
| 7 | **Assessment of assumptions** | Describe any methods or prior knowledge used to assess the assumptions or justify their validity | Page 3-4 |
| 8 | **Sensitivity analyses and additional analyses** | Describe any sensitivity analyses or additional analyses performed (e.g. comparison of effect estimates from different approaches, independent replication, bias analytic techniques, validation of instruments, simulations) | Page 3-4 |
| 9 | **Software and pre-registration** |  |  |
|  | a) | Name statistical software and package(s), including version and settings used | Page 3-4 |
|  | b) | State whether the study protocol and details were pre-registered (as well as when and where) |  |
|  | **RESULTS** |  |  |
| 10 | **Descriptive data** |  |  |
|  | a) | Report the numbers of individuals at each stage of included studies and reasons for exclusion. Consider use of a flow diagram |  |
|  | b) | Report summary statistics for phenotypic exposure(s), outcome(s), and other relevant variables (e.g. means, SDs, proportions) | Page 4-8  There is no sample overlap between the exposure and outcome studies. |
|  | c) | If the data sources include meta-analyses of previous studies, provide the assessments of heterogeneity across these studies |  |
|  | d) | For two-sample MR:  i.  Provide justification of the similarity of the genetic variant-exposure associations between the exposure and outcome samples  ii.  Provide information on the number of individuals who overlap between the exposure and outcome studies |  |
| 11 | **Main results** |  |  |
|  | a) | Report the associations between genetic variant and exposure, and between genetic variant and outcome, preferably on an interpretable scale | Page 4-8, Figure 2-6 |
|  | b) | Report MR estimates of the relationship between exposure and outcome, and the measures of uncertainty from the MR analysis, on an interpretable scale, such as odds ratio or relative risk per SD difference |  |
|  | c) | If relevant, consider translating estimates of relative risk into absolute risk for a meaningful time period |  |
|  | d) | Consider plots to visualize results (e.g. forest plot, scatterplot of associations between genetic variants and outcome versus between genetic variants and exposure) |  |
| 12 | **Assessment of assumptions** |  | Page 4-5，Figure 2-6 |
|  | a) | Report the assessment of the validity of the assumptions |  |
|  | b) | Report any additional statistics (e.g., assessments of heterogeneity across genetic variants, such as *I^2^*, Q statistic or E-value) |  |
| 13 | **Sensitivity analyses and additional analyses** |  | Page 4-5，Figure 2-6 |
|  | a) | Report any sensitivity analyses to assess the robustness of the main results to violations of the assumptions |  |
|  | b) | Report results from other sensitivity analyses or additional analyses |  |
|  | c) | Report any assessment of direction of causal relationship (e.g., bidirectional MR) |  |
|  | d) | When relevant, report and compare with estimates from non-MR analyses |  |
|  | e) | Consider additional plots to visualize results (e.g., leave-one-out analyses) |  |
|  | **DISCUSSION** |  | Page 5-7 |
| 14 | **Key results** | Summarize key results with reference to study objectives | Page 5 |
| 15 | **Limitations** | Discuss limitations of the study, taking into account the validity of the IV assumptions, other sources of potential bias, and imprecision. Discuss both direction and magnitude of any potential bias and any efforts to address them | Page 7-8 |
| 16 | **Interpretation** |  |  |
|  | a) | Meaning: Give a cautious overall interpretation of results in the context of their limitations and in comparison with other studies | Page5-7 |
|  | b) | Mechanism: Discuss underlying biological mechanisms that could drive a potential causal relationship between the investigated exposure and the outcome, and whether the gene-environment equivalence assumption is reasonable. Use causal language carefully, clarifying that IV estimates may provide causal effects only under certain assumptions |  |
|  | c) | Clinical relevance: Discuss whether the results have clinical or public policy relevance, and to what extent they inform effect sizes of possible interventions |  |
| 17 | **Generalizability** | Discuss the generalizability of the study results (a) to other populations, (b) across other exposure periods/timings, and (c) across other levels of exposure |  |
|  | **OTHER INFORMATION** |  |  |
| 18 | **Funding** | Describe sources of funding and the role of funders in the present study and, if applicable, sources of funding for the databases and original study or studies on which the present study is based |  |
| 19 | **Data and data sharing** | Provide the data used to perform all analyses or report where and how the data can be accessed, and reference these sources in the article. Provide the statistical code needed to reproduce the results in the article, or report whether the code is publicly accessible and if so, where | Page 8 |
| 20 | **Conflicts of Interest** | All authors should declare all potential conflicts of interest | Page 9 |

This checklist is copyrighted by the Equator Network under the Creative Commons Attribution 3.0 Unported (CC BY 3.0) license.

1. Skrivankova VW, Richmond RC, Woolf BAR, Yarmolinsky J, Davies NM, Swanson SA, et al. Strengthening the Reporting of Observational Studies in Epidemiology using Mendelian Randomization (STROBE-MR) Statement. JAMA. 2021;under review.

2. Skrivankova VW, Richmond RC, Woolf BAR, Davies NM, Swanson SA, VanderWeele TJ, et al. Strengthening the Reporting of Observational Studies in Epidemiology using Mendelian Randomisation (STROBE-MR): Explanation and Elaboration. BMJ. 2021;375:n2233.

**Supplementary Table 4.** The association between 91 circulating inflammatory proteins and ITP

|  | id.exposure | id.outcome | exposure | method | nsnp | b | se | pval | lo_ci | up_ci | or | or_lci95 | or_uci95 |
| --- | --- | --- | --- | --- | --- | --- | --- | --- | --- | --- | --- | --- | --- |
| 1 | GCST90274758 | ebi-a-GCST90018865 | level of eukaryotic translation initiation factor 4E-binding protein 1 in blood plasma | MR Egger | 11 | 0.210663 | 0.709036 | 0.773125 | -1.17905 | 1.600374 | 1.234496 | 0.307571 | 4.954885 |
| 2 | GCST90274758 | ebi-a-GCST90018865 | level of eukaryotic translation initiation factor 4E-binding protein 1 in blood plasma | Weighted median | 11 | 0.383566 | 0.302747 | 0.205172 | -0.20982 | 0.97695 | 1.467508 | 0.810732 | 2.656343 |
| 3 | GCST90274758 | ebi-a-GCST90018865 | level of eukaryotic translation initiation factor 4E-binding protein 1 in blood plasma | Inverse variance weighted | 11 | 0.316306 | 0.264108 | 0.231058 | -0.20135 | 0.833958 | 1.37205 | 0.817629 | 2.302413 |
| 4 | GCST90274758 | ebi-a-GCST90018865 | level of eukaryotic translation initiation factor 4E-binding protein 1 in blood plasma | Simple mode | 11 | 0.178253 | 0.512173 | 0.735031 | -0.82561 | 1.182111 | 1.195127 | 0.43797 | 3.261253 |
| 5 | GCST90274758 | ebi-a-GCST90018865 | level of eukaryotic translation initiation factor 4E-binding protein 1 in blood plasma | Weighted mode | 11 | 0.696918 | 0.359349 | 0.081163 | -0.00741 | 1.401241 | 2.007555 | 0.992621 | 4.060236 |
| 6 | GCST90274759 | ebi-a-GCST90018865 | adenosine deaminase measurement | MR Egger | 15 | -0.08799 | 0.137011 | 0.531903 | -0.35653 | 0.180552 | 0.915771 | 0.700101 | 1.197879 |
| 7 | GCST90274759 | ebi-a-GCST90018865 | adenosine deaminase measurement | Weighted median | 15 | -0.07234 | 0.135562 | 0.593579 | -0.33805 | 0.193358 | 0.930211 | 0.713163 | 1.213317 |
| 8 | GCST90274759 | ebi-a-GCST90018865 | adenosine deaminase measurement | Inverse variance weighted | 15 | -0.07455 | 0.110357 | 0.499342 | -0.29085 | 0.141751 | 0.928162 | 0.747628 | 1.15229 |
| 9 | GCST90274759 | ebi-a-GCST90018865 | adenosine deaminase measurement | Simple mode | 15 | 0.251368 | 0.261929 | 0.3535 | -0.26201 | 0.764749 | 1.285783 | 0.7695 | 2.148456 |
| 10 | GCST90274759 | ebi-a-GCST90018865 | adenosine deaminase measurement | Weighted mode | 15 | -0.05071 | 0.125344 | 0.691918 | -0.29638 | 0.194965 | 0.950556 | 0.743503 | 1.215268 |
| 11 | GCST90274760 | ebi-a-GCST90018865 | artemin measurement | MR Egger | 20 | -0.7093 | 0.470073 | 0.148676 | -1.63064 | 0.212047 | 0.49199 | 0.195804 | 1.236206 |
| 12 | GCST90274760 | ebi-a-GCST90018865 | artemin measurement | Weighted median | 20 | -0.23593 | 0.247286 | 0.340038 | -0.72061 | 0.248747 | 0.789833 | 0.486454 | 1.282418 |
| 13 | GCST90274760 | ebi-a-GCST90018865 | artemin measurement | Inverse variance weighted | 20 | -0.21868 | 0.18413 | 0.23497 | -0.57958 | 0.142212 | 0.803577 | 0.560135 | 1.152821 |
| 14 | GCST90274760 | ebi-a-GCST90018865 | artemin measurement | Simple mode | 20 | -0.57582 | 0.47858 | 0.243684 | -1.51384 | 0.362197 | 0.562244 | 0.220064 | 1.436481 |
| 15 | GCST90274760 | ebi-a-GCST90018865 | artemin measurement | Weighted mode | 20 | -0.55302 | 0.458405 | 0.242467 | -1.4515 | 0.34545 | 0.575208 | 0.234219 | 1.412626 |
| 16 | GCST90274761 | ebi-a-GCST90018865 | axin-1 measurement | MR Egger | 8 | 1.193582 | 0.651348 | 0.116587 | -0.08306 | 2.470223 | 3.298877 | 0.920297 | 11.82509 |
| 17 | GCST90274761 | ebi-a-GCST90018865 | axin-1 measurement | Weighted median | 8 | 0.100989 | 0.356709 | 0.777091 | -0.59816 | 0.800138 | 1.106264 | 0.549822 | 2.225849 |
| 18 | GCST90274761 | ebi-a-GCST90018865 | axin-1 measurement | Inverse variance weighted | 8 | 0.391342 | 0.271584 | 0.149596 | -0.14096 | 0.923646 | 1.478964 | 0.868522 | 2.518456 |
| 19 | GCST90274761 | ebi-a-GCST90018865 | axin-1 measurement | Simple mode | 8 | 0.117664 | 0.538794 | 0.833359 | -0.93837 | 1.173701 | 1.124867 | 0.391264 | 3.233938 |
| 20 | GCST90274761 | ebi-a-GCST90018865 | axin-1 measurement | Weighted mode | 8 | 0.071534 | 0.50264 | 0.890839 | -0.91364 | 1.056708 | 1.074155 | 0.401062 | 2.876886 |
| 21 | GCST90274762 | ebi-a-GCST90018865 | beta-nerve growth factor measurement | MR Egger | 22 | 0.367957 | 0.430719 | 0.403064 | -0.47625 | 1.212167 | 1.44478 | 0.621107 | 3.360759 |
| 22 | GCST90274762 | ebi-a-GCST90018865 | beta-nerve growth factor measurement | Weighted median | 22 | -0.13357 | 0.254902 | 0.600267 | -0.63318 | 0.366035 | 0.874964 | 0.530901 | 1.442006 |
| 23 | GCST90274762 | ebi-a-GCST90018865 | beta-nerve growth factor measurement | Inverse variance weighted | 22 | 0.025349 | 0.181169 | 0.888724 | -0.32974 | 0.380439 | 1.025673 | 0.71911 | 1.462927 |
| 24 | GCST90274762 | ebi-a-GCST90018865 | beta-nerve growth factor measurement | Simple mode | 22 | -0.20899 | 0.45493 | 0.65067 | -1.10066 | 0.68267 | 0.811401 | 0.332653 | 1.979156 |
| 25 | GCST90274762 | ebi-a-GCST90018865 | beta-nerve growth factor measurement | Weighted mode | 22 | -0.20899 | 0.483126 | 0.669726 | -1.15592 | 0.737933 | 0.811401 | 0.314768 | 2.091608 |
| 26 | GCST90274763 | ebi-a-GCST90018865 | caspase-8 measurement | MR Egger | 13 | 0.235778 | 0.485597 | 0.636818 | -0.71599 | 1.187547 | 1.265893 | 0.488707 | 3.279028 |
| 27 | GCST90274763 | ebi-a-GCST90018865 | caspase-8 measurement | Weighted median | 13 | 0.504161 | 0.326971 | 0.123095 | -0.1367 | 1.145024 | 1.655596 | 0.87223 | 3.142518 |
| 28 | GCST90274763 | ebi-a-GCST90018865 | caspase-8 measurement | Inverse variance weighted | 13 | 0.388522 | 0.224114 | 0.082991 | -0.05074 | 0.827786 | 1.4748 | 0.950524 | 2.288247 |
| 29 | GCST90274763 | ebi-a-GCST90018865 | caspase-8 measurement | Simple mode | 13 | 0.733957 | 0.482285 | 0.153957 | -0.21132 | 1.679236 | 2.083309 | 0.809514 | 5.361456 |
| 30 | GCST90274763 | ebi-a-GCST90018865 | caspase-8 measurement | Weighted mode | 13 | 0.585316 | 0.390185 | 0.159432 | -0.17945 | 1.350078 | 1.795559 | 0.835733 | 3.857727 |
| 31 | GCST90274764 | ebi-a-GCST90018865 | eotaxin measurement | MR Egger | 20 | -0.26445 | 0.332079 | 0.4362 | -0.91533 | 0.386419 | 0.767624 | 0.400385 | 1.471702 |
| 32 | GCST90274764 | ebi-a-GCST90018865 | eotaxin measurement | Weighted median | 20 | -0.08272 | 0.22522 | 0.713402 | -0.52415 | 0.35871 | 0.920608 | 0.592057 | 1.431482 |
| 33 | GCST90274764 | ebi-a-GCST90018865 | eotaxin measurement | Inverse variance weighted | 20 | -0.10967 | 0.15889 | 0.490032 | -0.4211 | 0.201749 | 0.896125 | 0.656325 | 1.223541 |
| 34 | GCST90274764 | ebi-a-GCST90018865 | eotaxin measurement | Simple mode | 20 | 0.034934 | 0.331404 | 0.917155 | -0.61462 | 0.684486 | 1.035551 | 0.540847 | 1.982753 |
| 35 | GCST90274764 | ebi-a-GCST90018865 | eotaxin measurement | Weighted mode | 20 | -0.05948 | 0.269675 | 0.82778 | -0.58805 | 0.469081 | 0.942252 | 0.555412 | 1.598525 |
| 36 | GCST90274765 | ebi-a-GCST90018865 | C-C motif chemokine 19 measurement | MR Egger | 19 | 0.09071 | 0.202117 | 0.659243 | -0.30544 | 0.486859 | 1.094952 | 0.7368 | 1.627197 |
| 37 | GCST90274765 | ebi-a-GCST90018865 | C-C motif chemokine 19 measurement | Weighted median | 19 | 0.173647 | 0.191221 | 0.363828 | -0.20115 | 0.54844 | 1.189636 | 0.817793 | 1.730551 |
| 38 | GCST90274765 | ebi-a-GCST90018865 | C-C motif chemokine 19 measurement | Inverse variance weighted | 19 | 0.181437 | 0.132824 | 0.17194 | -0.0789 | 0.441773 | 1.198939 | 0.924134 | 1.555462 |
| 39 | GCST90274765 | ebi-a-GCST90018865 | C-C motif chemokine 19 measurement | Simple mode | 19 | 0.17201 | 0.289473 | 0.559762 | -0.39536 | 0.739377 | 1.18769 | 0.67344 | 2.09463 |
| 40 | GCST90274765 | ebi-a-GCST90018865 | C-C motif chemokine 19 measurement | Weighted mode | 19 | 0.195814 | 0.172344 | 0.270781 | -0.14198 | 0.533607 | 1.2163 | 0.867639 | 1.705072 |
| 41 | GCST90274766 | ebi-a-GCST90018865 | C-C motif chemokine 20 measurement | MR Egger | 22 | 0.221079 | 0.475745 | 0.647165 | -0.71138 | 1.15354 | 1.247422 | 0.490966 | 3.169392 |
| 42 | GCST90274766 | ebi-a-GCST90018865 | C-C motif chemokine 20 measurement | Weighted median | 22 | -0.26185 | 0.259102 | 0.312197 | -0.7697 | 0.245986 | 0.769623 | 0.463154 | 1.278882 |
| 43 | GCST90274766 | ebi-a-GCST90018865 | C-C motif chemokine 20 measurement | Inverse variance weighted | 22 | -0.16288 | 0.183669 | 0.375186 | -0.52287 | 0.197113 | 0.849695 | 0.592818 | 1.217882 |
| 44 | GCST90274766 | ebi-a-GCST90018865 | C-C motif chemokine 20 measurement | Simple mode | 22 | -0.43544 | 0.488124 | 0.382467 | -1.39216 | 0.521286 | 0.646982 | 0.248538 | 1.684192 |
| 45 | GCST90274766 | ebi-a-GCST90018865 | C-C motif chemokine 20 measurement | Weighted mode | 22 | -0.32728 | 0.393644 | 0.41509 | -1.09883 | 0.444258 | 0.720879 | 0.333262 | 1.559333 |
| 46 | GCST90274767 | ebi-a-GCST90018865 | C-C motif chemokine 23 measurement | MR Egger | 26 | -0.00555 | 0.152576 | 0.971295 | -0.3046 | 0.293502 | 0.994467 | 0.73742 | 1.341116 |
| 47 | GCST90274767 | ebi-a-GCST90018865 | C-C motif chemokine 23 measurement | Weighted median | 26 | -0.12166 | 0.123497 | 0.324563 | -0.36371 | 0.120394 | 0.88545 | 0.695091 | 1.127941 |
| 48 | GCST90274767 | ebi-a-GCST90018865 | C-C motif chemokine 23 measurement | Inverse variance weighted | 26 | -0.07458 | 0.108596 | 0.492259 | -0.28742 | 0.138273 | 0.928138 | 0.750194 | 1.148289 |
| 49 | GCST90274767 | ebi-a-GCST90018865 | C-C motif chemokine 23 measurement | Simple mode | 26 | -0.12205 | 0.409803 | 0.768303 | -0.92526 | 0.681167 | 0.885107 | 0.396428 | 1.976182 |
| 50 | GCST90274767 | ebi-a-GCST90018865 | C-C motif chemokine 23 measurement | Weighted mode | 26 | -0.13584 | 0.12558 | 0.289722 | -0.38197 | 0.1103 | 0.872986 | 0.682514 | 1.116613 |
| 51 | GCST90274768 | ebi-a-GCST90018865 | C-C motif chemokine 25 measurement | MR Egger | 24 | 0.124633 | 0.119612 | 0.30874 | -0.10981 | 0.359073 | 1.132732 | 0.896007 | 1.432001 |
| 52 | GCST90274768 | ebi-a-GCST90018865 | C-C motif chemokine 25 measurement | Weighted median | 24 | 0.124536 | 0.088356 | 0.158694 | -0.04864 | 0.297713 | 1.132622 | 0.952522 | 1.346776 |
| 53 | GCST90274768 | ebi-a-GCST90018865 | C-C motif chemokine 25 measurement | Inverse variance weighted | 24 | 0.027727 | 0.090806 | 0.760105 | -0.15025 | 0.205706 | 1.028115 | 0.860491 | 1.228392 |
| 54 | GCST90274768 | ebi-a-GCST90018865 | C-C motif chemokine 25 measurement | Simple mode | 24 | -0.67081 | 0.28278 | 0.026429 | -1.22506 | -0.11656 | 0.511292 | 0.293739 | 0.889973 |
| 55 | GCST90274768 | ebi-a-GCST90018865 | C-C motif chemokine 25 measurement | Weighted mode | 24 | 0.120191 | 0.085331 | 0.172352 | -0.04706 | 0.28744 | 1.127712 | 0.954032 | 1.333011 |
| 56 | GCST90274769 | ebi-a-GCST90018865 | C-C motif chemokine 28 measurement | MR Egger | 22 | 0.052829 | 0.391313 | 0.893957 | -0.71414 | 0.819803 | 1.05425 | 0.489611 | 2.270052 |
| 57 | GCST90274769 | ebi-a-GCST90018865 | C-C motif chemokine 28 measurement | Weighted median | 22 | -0.41869 | 0.255341 | 0.101059 | -0.91916 | 0.081775 | 0.657906 | 0.398853 | 1.085212 |
| 58 | GCST90274769 | ebi-a-GCST90018865 | C-C motif chemokine 28 measurement | Inverse variance weighted | 22 | -0.25988 | 0.189666 | 0.170618 | -0.63163 | 0.111861 | 0.771141 | 0.531725 | 1.118357 |
| 59 | GCST90274769 | ebi-a-GCST90018865 | C-C motif chemokine 28 measurement | Simple mode | 22 | -0.4129 | 0.447181 | 0.366319 | -1.28938 | 0.463575 | 0.661728 | 0.275443 | 1.589748 |
| 60 | GCST90274769 | ebi-a-GCST90018865 | C-C motif chemokine 28 measurement | Weighted mode | 22 | -0.4465 | 0.405493 | 0.2833 | -1.24127 | 0.348264 | 0.639863 | 0.289018 | 1.416606 |
| 61 | GCST90274770 | ebi-a-GCST90018865 | C-C motif chemokine 4-like measurement | MR Egger | 19 | 0.042826 | 0.145392 | 0.7719 | -0.24214 | 0.327795 | 1.043756 | 0.784944 | 1.387905 |
| 62 | GCST90274770 | ebi-a-GCST90018865 | C-C motif chemokine 4-like measurement | Weighted median | 19 | 0.1627 | 0.135945 | 0.231385 | -0.10375 | 0.429153 | 1.176683 | 0.901447 | 1.535955 |
| 63 | GCST90274770 | ebi-a-GCST90018865 | C-C motif chemokine 4-like measurement | Inverse variance weighted | 19 | 0.211445 | 0.104723 | 0.043478 | 0.006188 | 0.416702 | 1.235462 | 1.006208 | 1.516951 |
| 64 | GCST90274770 | ebi-a-GCST90018865 | C-C motif chemokine 4-like measurement | Simple mode | 19 | 0.068322 | 0.28548 | 0.813559 | -0.49122 | 0.627864 | 1.07071 | 0.61188 | 1.873603 |
| 65 | GCST90274770 | ebi-a-GCST90018865 | C-C motif chemokine 4-like measurement | Weighted mode | 19 | 0.153604 | 0.111297 | 0.184449 | -0.06454 | 0.371745 | 1.166029 | 0.937501 | 1.450263 |
| 66 | GCST90274771 | ebi-a-GCST90018865 | natural killer cell receptor 2B4 measurement | MR Egger | 21 | 0.129249 | 0.354417 | 0.719377 | -0.56541 | 0.823907 | 1.137974 | 0.568128 | 2.279388 |
| 67 | GCST90274771 | ebi-a-GCST90018865 | natural killer cell receptor 2B4 measurement | Weighted median | 21 | 0.022366 | 0.213377 | 0.91652 | -0.39585 | 0.440584 | 1.022618 | 0.673106 | 1.553614 |
| 68 | GCST90274771 | ebi-a-GCST90018865 | natural killer cell receptor 2B4 measurement | Inverse variance weighted | 21 | -0.07496 | 0.162632 | 0.644847 | -0.39372 | 0.243796 | 0.927778 | 0.674542 | 1.276084 |
| 69 | GCST90274771 | ebi-a-GCST90018865 | natural killer cell receptor 2B4 measurement | Simple mode | 21 | 0.032441 | 0.388355 | 0.934257 | -0.72873 | 0.793616 | 1.032973 | 0.482519 | 2.211378 |
| 70 | GCST90274771 | ebi-a-GCST90018865 | natural killer cell receptor 2B4 measurement | Weighted mode | 21 | 0.032441 | 0.233501 | 0.890893 | -0.42522 | 0.490102 | 1.032973 | 0.653626 | 1.632483 |
| 71 | GCST90274772 | ebi-a-GCST90018865 | CD40 measurement | MR Egger | 16 | -0.01076 | 0.147131 | 0.942739 | -0.29914 | 0.277617 | 0.989298 | 0.741459 | 1.31998 |
| 72 | GCST90274772 | ebi-a-GCST90018865 | CD40 measurement | Weighted median | 16 | -0.01877 | 0.115837 | 0.871298 | -0.24581 | 0.208274 | 0.981408 | 0.782072 | 1.231551 |
| 73 | GCST90274772 | ebi-a-GCST90018865 | CD40 measurement | Inverse variance weighted | 16 | -0.01968 | 0.106022 | 0.852723 | -0.22749 | 0.188121 | 0.98051 | 0.796534 | 1.206979 |
| 74 | GCST90274772 | ebi-a-GCST90018865 | CD40 measurement | Simple mode | 16 | 0.142272 | 0.363222 | 0.700793 | -0.56964 | 0.854187 | 1.15289 | 0.565727 | 2.349464 |
| 75 | GCST90274772 | ebi-a-GCST90018865 | CD40 measurement | Weighted mode | 16 | -0.01767 | 0.117784 | 0.882759 | -0.24853 | 0.213189 | 0.982487 | 0.77995 | 1.237618 |
| 76 | GCST90274773 | ebi-a-GCST90018865 | t-cell surface glycoprotein CD5 measurement | MR Egger | 22 | -0.29816 | 0.46932 | 0.532428 | -1.21803 | 0.621704 | 0.742179 | 0.295812 | 1.862097 |
| 77 | GCST90274773 | ebi-a-GCST90018865 | t-cell surface glycoprotein CD5 measurement | Weighted median | 22 | -0.24425 | 0.247731 | 0.324162 | -0.7298 | 0.241304 | 0.783294 | 0.482006 | 1.272908 |
| 78 | GCST90274773 | ebi-a-GCST90018865 | t-cell surface glycoprotein CD5 measurement | Inverse variance weighted | 22 | -0.07088 | 0.175073 | 0.685579 | -0.41402 | 0.272263 | 0.931573 | 0.660985 | 1.312932 |
| 79 | GCST90274773 | ebi-a-GCST90018865 | t-cell surface glycoprotein CD5 measurement | Simple mode | 22 | -0.30131 | 0.437636 | 0.498676 | -1.15908 | 0.556456 | 0.739848 | 0.313776 | 1.744479 |
| 80 | GCST90274773 | ebi-a-GCST90018865 | t-cell surface glycoprotein CD5 measurement | Weighted mode | 22 | -0.29176 | 0.452944 | 0.526464 | -1.17952 | 0.596014 | 0.746951 | 0.307425 | 1.81487 |
| 81 | GCST90274774 | ebi-a-GCST90018865 | level of T-cell differentiation antigen CD6 in blood plasma | MR Egger | 17 | -0.17696 | 0.153985 | 0.268477 | -0.47877 | 0.124854 | 0.837816 | 0.619547 | 1.132983 |
| 82 | GCST90274774 | ebi-a-GCST90018865 | level of T-cell differentiation antigen CD6 in blood plasma | Weighted median | 17 | 0.035879 | 0.122695 | 0.769962 | -0.2046 | 0.276362 | 1.036531 | 0.814971 | 1.318324 |
| 83 | GCST90274774 | ebi-a-GCST90018865 | level of T-cell differentiation antigen CD6 in blood plasma | Inverse variance weighted | 17 | 0.045955 | 0.093501 | 0.623079 | -0.13731 | 0.229217 | 1.047027 | 0.871703 | 1.257614 |
| 84 | GCST90274774 | ebi-a-GCST90018865 | level of T-cell differentiation antigen CD6 in blood plasma | Simple mode | 17 | 0.230252 | 0.276083 | 0.416563 | -0.31087 | 0.771375 | 1.258917 | 0.732808 | 2.162738 |
| 85 | GCST90274774 | ebi-a-GCST90018865 | level of T-cell differentiation antigen CD6 in blood plasma | Weighted mode | 17 | -0.04726 | 0.112156 | 0.679109 | -0.26708 | 0.172569 | 0.953843 | 0.76561 | 1.188354 |
| 86 | GCST90274775 | ebi-a-GCST90018865 | CUB domain-containing protein 1 measurement | MR Egger | 24 | -0.0194 | 0.235501 | 0.935076 | -0.48099 | 0.442178 | 0.980782 | 0.618173 | 1.556093 |
| 87 | GCST90274775 | ebi-a-GCST90018865 | CUB domain-containing protein 1 measurement | Weighted median | 24 | 0.052834 | 0.18922 | 0.780078 | -0.31804 | 0.423705 | 1.054254 | 0.727575 | 1.527612 |
| 88 | GCST90274775 | ebi-a-GCST90018865 | CUB domain-containing protein 1 measurement | Inverse variance weighted | 24 | 0.001261 | 0.128502 | 0.992172 | -0.2506 | 0.253124 | 1.001262 | 0.778332 | 1.288043 |
| 89 | GCST90274775 | ebi-a-GCST90018865 | CUB domain-containing protein 1 measurement | Simple mode | 24 | -0.06469 | 0.317684 | 0.840428 | -0.68735 | 0.557967 | 0.937356 | 0.502906 | 1.747118 |
| 90 | GCST90274775 | ebi-a-GCST90018865 | CUB domain-containing protein 1 measurement | Weighted mode | 24 | 0.051075 | 0.193105 | 0.793755 | -0.32741 | 0.42956 | 1.052402 | 0.720788 | 1.536581 |
| 91 | GCST90274776 | ebi-a-GCST90018865 | macrophage colony-stimulating factor 1 measurement | MR Egger | 17 | 0.022486 | 0.487443 | 0.963815 | -0.9329 | 0.977875 | 1.022741 | 0.39341 | 2.658799 |
| 92 | GCST90274776 | ebi-a-GCST90018865 | macrophage colony-stimulating factor 1 measurement | Weighted median | 17 | 0.01744 | 0.246426 | 0.94358 | -0.46555 | 0.500434 | 1.017593 | 0.627787 | 1.649437 |
| 93 | GCST90274776 | ebi-a-GCST90018865 | macrophage colony-stimulating factor 1 measurement | Inverse variance weighted | 17 | -0.15203 | 0.184041 | 0.408772 | -0.51275 | 0.208693 | 0.858964 | 0.598847 | 1.232066 |
| 94 | GCST90274776 | ebi-a-GCST90018865 | macrophage colony-stimulating factor 1 measurement | Simple mode | 17 | -0.77026 | 0.512721 | 0.1525 | -1.77519 | 0.234676 | 0.462894 | 0.169451 | 1.264499 |
| 95 | GCST90274776 | ebi-a-GCST90018865 | macrophage colony-stimulating factor 1 measurement | Weighted mode | 17 | 0.048512 | 0.299988 | 0.873557 | -0.53947 | 0.636489 | 1.049708 | 0.58306 | 1.889834 |
| 96 | GCST90274777 | ebi-a-GCST90018865 | cystatin-D measurement | MR Egger | 30 | -0.09256 | 0.099893 | 0.362059 | -0.28835 | 0.103231 | 0.911594 | 0.749498 | 1.108747 |
| 97 | GCST90274777 | ebi-a-GCST90018865 | cystatin-D measurement | Weighted median | 30 | -0.0587 | 0.088881 | 0.508948 | -0.23291 | 0.115503 | 0.942986 | 0.792224 | 1.122438 |
| 98 | GCST90274777 | ebi-a-GCST90018865 | cystatin-D measurement | Inverse variance weighted | 30 | -0.05376 | 0.077587 | 0.488396 | -0.20583 | 0.098314 | 0.947662 | 0.813973 | 1.103309 |
| 99 | GCST90274777 | ebi-a-GCST90018865 | cystatin-D measurement | Simple mode | 30 | -0.16656 | 0.31066 | 0.595942 | -0.77545 | 0.442332 | 0.846571 | 0.460495 | 1.556332 |
| 100 | GCST90274777 | ebi-a-GCST90018865 | cystatin-D measurement | Weighted mode | 30 | -0.07192 | 0.091423 | 0.437856 | -0.25111 | 0.10727 | 0.930605 | 0.777937 | 1.113234 |
| 101 | GCST90274778 | ebi-a-GCST90018865 | fractalkine measurement | MR Egger | 23 | 0.58699 | 0.624483 | 0.357924 | -0.637 | 1.810976 | 1.798566 | 0.528879 | 6.116415 |
| 102 | GCST90274778 | ebi-a-GCST90018865 | fractalkine measurement | Weighted median | 23 | 0.403518 | 0.278762 | 0.147747 | -0.14286 | 0.949892 | 1.497082 | 0.866879 | 2.58543 |
| 103 | GCST90274778 | ebi-a-GCST90018865 | fractalkine measurement | Inverse variance weighted | 23 | 0.305589 | 0.23425 | 0.192049 | -0.15354 | 0.764719 | 1.357424 | 0.857665 | 2.148391 |
| 104 | GCST90274778 | ebi-a-GCST90018865 | fractalkine measurement | Simple mode | 23 | 0.382491 | 0.576666 | 0.514042 | -0.74777 | 1.512757 | 1.465932 | 0.473419 | 4.539226 |
| 105 | GCST90274778 | ebi-a-GCST90018865 | fractalkine measurement | Weighted mode | 23 | 0.449627 | 0.44531 | 0.323618 | -0.42318 | 1.322434 | 1.567727 | 0.654961 | 3.752543 |
| 106 | GCST90274779 | ebi-a-GCST90018865 | CXCL1 measurement | MR Egger | 12 | -0.06715 | 0.273929 | 0.81132 | -0.60405 | 0.469756 | 0.935059 | 0.546595 | 1.599604 |
| 107 | GCST90274779 | ebi-a-GCST90018865 | CXCL1 measurement | Weighted median | 12 | 0.05882 | 0.174021 | 0.735361 | -0.28226 | 0.399901 | 1.060584 | 0.754076 | 1.491677 |
| 108 | GCST90274779 | ebi-a-GCST90018865 | CXCL1 measurement | Inverse variance weighted | 12 | -0.00461 | 0.166979 | 0.977992 | -0.33189 | 0.322673 | 0.995404 | 0.717569 | 1.380814 |
| 109 | GCST90274779 | ebi-a-GCST90018865 | CXCL1 measurement | Simple mode | 12 | 0.096812 | 0.362944 | 0.794604 | -0.61456 | 0.808182 | 1.101653 | 0.540879 | 2.243826 |
| 110 | GCST90274779 | ebi-a-GCST90018865 | CXCL1 measurement | Weighted mode | 12 | 0.062276 | 0.171947 | 0.724078 | -0.27474 | 0.399291 | 1.064256 | 0.75977 | 1.490768 |
| 111 | GCST90274780 | ebi-a-GCST90018865 | C-X-C motif chemokine 10 measurement | MR Egger | 23 | -0.00256 | 0.324512 | 0.99379 | -0.6386 | 0.633487 | 0.997447 | 0.528032 | 1.884169 |
| 112 | GCST90274780 | ebi-a-GCST90018865 | C-X-C motif chemokine 10 measurement | Weighted median | 23 | -0.09434 | 0.238365 | 0.692265 | -0.56154 | 0.372855 | 0.909973 | 0.570332 | 1.451873 |
| 113 | GCST90274780 | ebi-a-GCST90018865 | C-X-C motif chemokine 10 measurement | Inverse variance weighted | 23 | 0.115352 | 0.182232 | 0.526738 | -0.24182 | 0.472527 | 1.122268 | 0.785195 | 1.604042 |
| 114 | GCST90274780 | ebi-a-GCST90018865 | C-X-C motif chemokine 10 measurement | Simple mode | 23 | -0.04457 | 0.41258 | 0.914945 | -0.85323 | 0.764082 | 0.956405 | 0.426037 | 2.147022 |
| 115 | GCST90274780 | ebi-a-GCST90018865 | C-X-C motif chemokine 10 measurement | Weighted mode | 23 | -0.17596 | 0.293463 | 0.554893 | -0.75115 | 0.399225 | 0.838649 | 0.471824 | 1.490668 |
| 116 | GCST90274781 | ebi-a-GCST90018865 | C-X-C motif chemokine 11 measurement | MR Egger | 24 | 0.109073 | 0.367018 | 0.769112 | -0.61028 | 0.828429 | 1.115244 | 0.543197 | 2.289719 |
| 117 | GCST90274781 | ebi-a-GCST90018865 | C-X-C motif chemokine 11 measurement | Weighted median | 24 | 0.308484 | 0.238306 | 0.195498 | -0.1586 | 0.775564 | 1.36136 | 0.853341 | 2.171817 |
| 118 | GCST90274781 | ebi-a-GCST90018865 | C-X-C motif chemokine 11 measurement | Inverse variance weighted | 24 | 0.245786 | 0.150493 | 0.102426 | -0.04918 | 0.540753 | 1.278626 | 0.952009 | 1.717299 |
| 119 | GCST90274781 | ebi-a-GCST90018865 | C-X-C motif chemokine 11 measurement | Simple mode | 24 | -0.00269 | 0.388309 | 0.994541 | -0.76377 | 0.7584 | 0.997318 | 0.465906 | 2.134857 |
| 120 | GCST90274781 | ebi-a-GCST90018865 | C-X-C motif chemokine 11 measurement | Weighted mode | 24 | 0.299242 | 0.286933 | 0.307835 | -0.26315 | 0.861632 | 1.348836 | 0.768628 | 2.36702 |
| 121 | GCST90274782 | ebi-a-GCST90018865 | C-X-C motif chemokine 5 measurement | MR Egger | 15 | 0.06111 | 0.20098 | 0.765896 | -0.33281 | 0.45503 | 1.063015 | 0.716906 | 1.576221 |
| 122 | GCST90274782 | ebi-a-GCST90018865 | C-X-C motif chemokine 5 measurement | Weighted median | 15 | 0.003459 | 0.152088 | 0.981854 | -0.29463 | 0.301551 | 1.003465 | 0.744805 | 1.351954 |
| 123 | GCST90274782 | ebi-a-GCST90018865 | C-X-C motif chemokine 5 measurement | Inverse variance weighted | 15 | -0.04267 | 0.131323 | 0.745229 | -0.30007 | 0.214722 | 0.958226 | 0.74077 | 1.239517 |
| 124 | GCST90274782 | ebi-a-GCST90018865 | C-X-C motif chemokine 5 measurement | Simple mode | 15 | 0.425466 | 0.3767 | 0.277687 | -0.31287 | 1.163798 | 1.530304 | 0.731348 | 3.202072 |
| 125 | GCST90274782 | ebi-a-GCST90018865 | C-X-C motif chemokine 5 measurement | Weighted mode | 15 | 0.024679 | 0.153288 | 0.874398 | -0.27577 | 0.325124 | 1.024986 | 0.75899 | 1.384202 |
| 126 | GCST90274783 | ebi-a-GCST90018865 | C-X-C motif chemokine 6 measurement | MR Egger | 15 | -0.55832 | 0.522345 | 0.304569 | -1.58212 | 0.465471 | 0.572167 | 0.205539 | 1.592765 |
| 127 | GCST90274783 | ebi-a-GCST90018865 | C-X-C motif chemokine 6 measurement | Weighted median | 15 | 0.303706 | 0.301673 | 0.314061 | -0.28757 | 0.894984 | 1.35487 | 0.750082 | 2.447297 |
| 128 | GCST90274783 | ebi-a-GCST90018865 | C-X-C motif chemokine 6 measurement | Inverse variance weighted | 15 | 0.075893 | 0.241361 | 0.75319 | -0.39717 | 0.54896 | 1.078847 | 0.672217 | 1.731451 |
| 129 | GCST90274783 | ebi-a-GCST90018865 | C-X-C motif chemokine 6 measurement | Simple mode | 15 | 0.230885 | 0.512376 | 0.659165 | -0.77337 | 1.235142 | 1.259715 | 0.461455 | 3.438866 |
| 130 | GCST90274783 | ebi-a-GCST90018865 | C-X-C motif chemokine 6 measurement | Weighted mode | 15 | 0.275582 | 0.422926 | 0.525206 | -0.55335 | 1.104517 | 1.317298 | 0.575019 | 3.017766 |
| 131 | GCST90274784 | ebi-a-GCST90018865 | C-X-C motif chemokine 9 measurement | MR Egger | 22 | 0.90458 | 0.420027 | 0.043649 | 0.081328 | 1.727832 | 2.470894 | 1.084727 | 5.628441 |
| 132 | GCST90274784 | ebi-a-GCST90018865 | C-X-C motif chemokine 9 measurement | Weighted median | 22 | 0.627545 | 0.258838 | 0.015331 | 0.120222 | 1.134868 | 1.873007 | 1.127747 | 3.110764 |
| 133 | GCST90274784 | ebi-a-GCST90018865 | C-X-C motif chemokine 9 measurement | Inverse variance weighted | 22 | 0.597022 | 0.179663 | 0.000891 | 0.244882 | 0.949162 | 1.816701 | 1.277471 | 2.583544 |
| 134 | GCST90274784 | ebi-a-GCST90018865 | C-X-C motif chemokine 9 measurement | Simple mode | 22 | 0.617251 | 0.442402 | 0.177529 | -0.24986 | 1.484359 | 1.853825 | 0.778912 | 4.412138 |
| 135 | GCST90274784 | ebi-a-GCST90018865 | C-X-C motif chemokine 9 measurement | Weighted mode | 22 | 0.607164 | 0.451995 | 0.193513 | -0.27875 | 1.493074 | 1.835219 | 0.756732 | 4.450758 |
| 136 | GCST90274785 | ebi-a-GCST90018865 | delta and Notch-like epidermal growth factor-related receptor measurement | MR Egger | 16 | -0.77865 | 0.425587 | 0.088686 | -1.6128 | 0.055504 | 0.459027 | 0.199329 | 1.057073 |
| 137 | GCST90274785 | ebi-a-GCST90018865 | delta and Notch-like epidermal growth factor-related receptor measurement | Weighted median | 16 | -0.32793 | 0.221997 | 0.139624 | -0.76305 | 0.107182 | 0.720411 | 0.466243 | 1.113136 |
| 138 | GCST90274785 | ebi-a-GCST90018865 | delta and Notch-like epidermal growth factor-related receptor measurement | Inverse variance weighted | 16 | -0.19238 | 0.194349 | 0.322232 | -0.57331 | 0.188541 | 0.824992 | 0.563659 | 1.207487 |
| 139 | GCST90274785 | ebi-a-GCST90018865 | delta and Notch-like epidermal growth factor-related receptor measurement | Simple mode | 16 | -0.20482 | 0.477313 | 0.673948 | -1.14035 | 0.730717 | 0.814798 | 0.319708 | 2.076569 |
| 140 | GCST90274785 | ebi-a-GCST90018865 | delta and Notch-like epidermal growth factor-related receptor measurement | Weighted mode | 16 | -0.41257 | 0.254115 | 0.125297 | -0.91063 | 0.085499 | 0.66195 | 0.402271 | 1.08926 |
| 141 | GCST90274786 | ebi-a-GCST90018865 | protein S100-A12 measurement | MR Egger | 17 | -0.3363 | 0.342097 | 0.341168 | -1.00681 | 0.334207 | 0.714406 | 0.365381 | 1.396833 |
| 142 | GCST90274786 | ebi-a-GCST90018865 | protein S100-A12 measurement | Weighted median | 17 | -0.3632 | 0.234329 | 0.121152 | -0.82249 | 0.096086 | 0.695447 | 0.439338 | 1.100853 |
| 143 | GCST90274786 | ebi-a-GCST90018865 | protein S100-A12 measurement | Inverse variance weighted | 17 | -0.26826 | 0.169574 | 0.113664 | -0.60062 | 0.064109 | 0.764712 | 0.548471 | 1.066209 |
| 144 | GCST90274786 | ebi-a-GCST90018865 | protein S100-A12 measurement | Simple mode | 17 | -0.32261 | 0.365507 | 0.390496 | -1.03901 | 0.39378 | 0.724253 | 0.353806 | 1.482574 |
| 145 | GCST90274786 | ebi-a-GCST90018865 | protein S100-A12 measurement | Weighted mode | 17 | -0.4092 | 0.27257 | 0.152765 | -0.94343 | 0.125039 | 0.664183 | 0.389289 | 1.133192 |
| 146 | GCST90274787 | ebi-a-GCST90018865 | fibroblast growth factor 19 measurement | MR Egger | 21 | -0.15354 | 0.474938 | 0.750004 | -1.08442 | 0.777334 | 0.857663 | 0.338097 | 2.175663 |
| 147 | GCST90274787 | ebi-a-GCST90018865 | fibroblast growth factor 19 measurement | Weighted median | 21 | 0.257704 | 0.251164 | 0.304874 | -0.23458 | 0.749985 | 1.293956 | 0.790905 | 2.116969 |
| 148 | GCST90274787 | ebi-a-GCST90018865 | fibroblast growth factor 19 measurement | Inverse variance weighted | 21 | 0.10046 | 0.193589 | 0.603806 | -0.27897 | 0.479895 | 1.10568 | 0.756559 | 1.615905 |
| 149 | GCST90274787 | ebi-a-GCST90018865 | fibroblast growth factor 19 measurement | Simple mode | 21 | 0.656516 | 0.473075 | 0.180477 | -0.27071 | 1.583743 | 1.928064 | 0.762838 | 4.87316 |
| 150 | GCST90274787 | ebi-a-GCST90018865 | fibroblast growth factor 19 measurement | Weighted mode | 21 | 0.315042 | 0.292928 | 0.294958 | -0.2591 | 0.88918 | 1.370317 | 0.771748 | 2.433134 |
| 151 | GCST90274788 | ebi-a-GCST90018865 | fibroblast growth factor 21 measurement | MR Egger | 14 | -0.62993 | 0.425455 | 0.164484 | -1.46382 | 0.203961 | 0.532629 | 0.23135 | 1.226251 |
| 152 | GCST90274788 | ebi-a-GCST90018865 | fibroblast growth factor 21 measurement | Weighted median | 14 | -0.26003 | 0.234069 | 0.266606 | -0.7188 | 0.198745 | 0.771029 | 0.487334 | 1.219871 |
| 153 | GCST90274788 | ebi-a-GCST90018865 | fibroblast growth factor 21 measurement | Inverse variance weighted | 14 | -0.16348 | 0.182308 | 0.369867 | -0.5208 | 0.193844 | 0.849184 | 0.594044 | 1.213906 |
| 154 | GCST90274788 | ebi-a-GCST90018865 | fibroblast growth factor 21 measurement | Simple mode | 14 | 0.156835 | 0.441914 | 0.728356 | -0.70932 | 1.022987 | 1.169803 | 0.49198 | 2.78149 |
| 155 | GCST90274788 | ebi-a-GCST90018865 | fibroblast growth factor 21 measurement | Weighted mode | 14 | -0.5091 | 0.254156 | 0.066473 | -1.00724 | -0.01095 | 0.601039 | 0.365225 | 0.989111 |
| 156 | GCST90274789 | ebi-a-GCST90018865 | fibroblast growth factor 23 measurement | MR Egger | 16 | 0.44853 | 0.647588 | 0.499879 | -0.82074 | 1.717802 | 1.566008 | 0.440105 | 5.572266 |
| 157 | GCST90274789 | ebi-a-GCST90018865 | fibroblast growth factor 23 measurement | Weighted median | 16 | -0.36918 | 0.282923 | 0.191936 | -0.92371 | 0.18535 | 0.691301 | 0.397044 | 1.20364 |
| 158 | GCST90274789 | ebi-a-GCST90018865 | fibroblast growth factor 23 measurement | Inverse variance weighted | 16 | -0.24576 | 0.202976 | 0.225984 | -0.64359 | 0.152076 | 0.782112 | 0.525402 | 1.164249 |
| 159 | GCST90274789 | ebi-a-GCST90018865 | fibroblast growth factor 23 measurement | Simple mode | 16 | -0.28504 | 0.489653 | 0.569131 | -1.24476 | 0.674682 | 0.751985 | 0.28801 | 1.963409 |
| 160 | GCST90274789 | ebi-a-GCST90018865 | fibroblast growth factor 23 measurement | Weighted mode | 16 | -0.48857 | 0.386413 | 0.225395 | -1.24594 | 0.268805 | 0.613506 | 0.287672 | 1.3084 |
| 161 | GCST90274790 | ebi-a-GCST90018865 | fibroblast growth factor 5 measurement | MR Egger | 24 | -0.09723 | 0.126606 | 0.450656 | -0.34538 | 0.150915 | 0.907345 | 0.707951 | 1.162898 |
| 162 | GCST90274790 | ebi-a-GCST90018865 | fibroblast growth factor 5 measurement | Weighted median | 24 | -0.11088 | 0.098799 | 0.261728 | -0.30453 | 0.082762 | 0.895043 | 0.737471 | 1.086283 |
| 163 | GCST90274790 | ebi-a-GCST90018865 | fibroblast growth factor 5 measurement | Inverse variance weighted | 24 | -0.10831 | 0.085554 | 0.205513 | -0.276 | 0.059375 | 0.897348 | 0.758814 | 1.061173 |
| 164 | GCST90274790 | ebi-a-GCST90018865 | fibroblast growth factor 5 measurement | Simple mode | 24 | -0.27561 | 0.34039 | 0.426414 | -0.94278 | 0.391553 | 0.759107 | 0.389545 | 1.479276 |
| 165 | GCST90274790 | ebi-a-GCST90018865 | fibroblast growth factor 5 measurement | Weighted mode | 24 | -0.11882 | 0.106992 | 0.278256 | -0.32852 | 0.090889 | 0.887972 | 0.719989 | 1.095147 |
| 166 | GCST90274791 | ebi-a-GCST90018865 | Fms-related tyrosine kinase 3 ligand measurement | MR Egger | 29 | 0.119109 | 0.219242 | 0.591398 | -0.31061 | 0.548823 | 1.126492 | 0.733003 | 1.731214 |
| 167 | GCST90274791 | ebi-a-GCST90018865 | Fms-related tyrosine kinase 3 ligand measurement | Weighted median | 29 | 0.225259 | 0.192383 | 0.241644 | -0.15181 | 0.602329 | 1.252647 | 0.85915 | 1.826368 |
| 168 | GCST90274791 | ebi-a-GCST90018865 | Fms-related tyrosine kinase 3 ligand measurement | Inverse variance weighted | 29 | 0.080948 | 0.12582 | 0.519992 | -0.16566 | 0.327556 | 1.084314 | 0.847334 | 1.387572 |
| 169 | GCST90274791 | ebi-a-GCST90018865 | Fms-related tyrosine kinase 3 ligand measurement | Simple mode | 29 | 0.353222 | 0.335609 | 0.301578 | -0.30457 | 1.011017 | 1.423648 | 0.737439 | 2.748394 |
| 170 | GCST90274791 | ebi-a-GCST90018865 | Fms-related tyrosine kinase 3 ligand measurement | Weighted mode | 29 | 0.248914 | 0.185408 | 0.190213 | -0.11449 | 0.612314 | 1.282631 | 0.891824 | 1.844694 |
| 171 | GCST90274792 | ebi-a-GCST90018865 | glial cell line-derived neurotrophic factor measurement | MR Egger | 16 | 0.171376 | 0.378562 | 0.657699 | -0.57061 | 0.913357 | 1.186937 | 0.565183 | 2.492677 |
| 172 | GCST90274792 | ebi-a-GCST90018865 | glial cell line-derived neurotrophic factor measurement | Weighted median | 16 | 0.291845 | 0.225071 | 0.194741 | -0.14929 | 0.732984 | 1.338896 | 0.861316 | 2.081281 |
| 173 | GCST90274792 | ebi-a-GCST90018865 | glial cell line-derived neurotrophic factor measurement | Inverse variance weighted | 16 | 0.052086 | 0.206623 | 0.800979 | -0.3529 | 0.457068 | 1.053466 | 0.70265 | 1.579436 |
| 174 | GCST90274792 | ebi-a-GCST90018865 | glial cell line-derived neurotrophic factor measurement | Simple mode | 16 | 0.356856 | 0.436889 | 0.426819 | -0.49945 | 1.213159 | 1.42883 | 0.606866 | 3.364094 |
| 175 | GCST90274792 | ebi-a-GCST90018865 | glial cell line-derived neurotrophic factor measurement | Weighted mode | 16 | 0.289587 | 0.218247 | 0.204395 | -0.13818 | 0.717351 | 1.335875 | 0.870944 | 2.048997 |
| 176 | GCST90274793 | ebi-a-GCST90018865 | hepatocyte growth factor measurement | MR Egger | 19 | -0.10816 | 0.53298 | 0.841593 | -1.1528 | 0.936479 | 0.897483 | 0.315751 | 2.550985 |
| 177 | GCST90274793 | ebi-a-GCST90018865 | hepatocyte growth factor measurement | Weighted median | 19 | -0.29555 | 0.314069 | 0.346688 | -0.91112 | 0.320026 | 0.744123 | 0.402072 | 1.377164 |
| 178 | GCST90274793 | ebi-a-GCST90018865 | hepatocyte growth factor measurement | Inverse variance weighted | 19 | -0.10429 | 0.224604 | 0.642399 | -0.54452 | 0.33593 | 0.90096 | 0.580121 | 1.399241 |
| 179 | GCST90274793 | ebi-a-GCST90018865 | hepatocyte growth factor measurement | Simple mode | 19 | 0.687286 | 0.638543 | 0.295993 | -0.56426 | 1.938831 | 1.988312 | 0.568781 | 6.950618 |
| 180 | GCST90274793 | ebi-a-GCST90018865 | hepatocyte growth factor measurement | Weighted mode | 19 | -0.84595 | 0.441445 | 0.071342 | -1.71118 | 0.019278 | 0.429148 | 0.180652 | 1.019465 |
| 181 | GCST90274794 | ebi-a-GCST90018865 | interferon gamma measurement | MR Egger | 10 | -0.42494 | 0.813795 | 0.615701 | -2.01997 | 1.170101 | 0.653812 | 0.132659 | 3.222319 |
| 182 | GCST90274794 | ebi-a-GCST90018865 | interferon gamma measurement | Weighted median | 10 | -0.72703 | 0.396705 | 0.066853 | -1.50457 | 0.050517 | 0.483344 | 0.222113 | 1.051814 |
| 183 | GCST90274794 | ebi-a-GCST90018865 | interferon gamma measurement | Inverse variance weighted | 10 | -0.50027 | 0.429189 | 0.243774 | -1.34148 | 0.340946 | 0.60637 | 0.261459 | 1.406277 |
| 184 | GCST90274794 | ebi-a-GCST90018865 | interferon gamma measurement | Simple mode | 10 | -1.13066 | 0.650828 | 0.11635 | -2.40628 | 0.14496 | 0.32282 | 0.09015 | 1.155994 |
| 185 | GCST90274794 | ebi-a-GCST90018865 | interferon gamma measurement | Weighted mode | 10 | -1.02129 | 0.61371 | 0.130444 | -2.22417 | 0.181577 | 0.360129 | 0.108158 | 1.199107 |
| 186 | GCST90274795 | ebi-a-GCST90018865 | interleukin-10 measurement | MR Egger | 19 | -0.15762 | 0.406923 | 0.703298 | -0.95519 | 0.639945 | 0.85417 | 0.384737 | 1.896376 |
| 187 | GCST90274795 | ebi-a-GCST90018865 | interleukin-10 measurement | Weighted median | 19 | 0.159365 | 0.254703 | 0.531519 | -0.33985 | 0.658582 | 1.172766 | 0.711875 | 1.932051 |
| 188 | GCST90274795 | ebi-a-GCST90018865 | interleukin-10 measurement | Inverse variance weighted | 19 | 0.247385 | 0.189922 | 0.192726 | -0.12486 | 0.619632 | 1.280672 | 0.882618 | 1.858245 |
| 189 | GCST90274795 | ebi-a-GCST90018865 | interleukin-10 measurement | Simple mode | 19 | 0.267181 | 0.420029 | 0.532716 | -0.55608 | 1.090438 | 1.306277 | 0.573455 | 2.975578 |
| 190 | GCST90274795 | ebi-a-GCST90018865 | interleukin-10 measurement | Weighted mode | 19 | 0.172426 | 0.369182 | 0.646068 | -0.55117 | 0.896023 | 1.188184 | 0.576275 | 2.449839 |
| 191 | GCST90274796 | ebi-a-GCST90018865 | interleukin-10 receptor subunit alpha measurement | MR Egger | 10 | 0.240825 | 0.321681 | 0.475504 | -0.38967 | 0.87132 | 1.272298 | 0.67728 | 2.390064 |
| 192 | GCST90274796 | ebi-a-GCST90018865 | interleukin-10 receptor subunit alpha measurement | Weighted median | 10 | 0.365439 | 0.2908 | 0.208875 | -0.20453 | 0.935408 | 1.441146 | 0.81503 | 2.548252 |
| 193 | GCST90274796 | ebi-a-GCST90018865 | interleukin-10 receptor subunit alpha measurement | Inverse variance weighted | 10 | 0.340995 | 0.202419 | 0.092065 | -0.05575 | 0.737735 | 1.406346 | 0.94578 | 2.091194 |
| 194 | GCST90274796 | ebi-a-GCST90018865 | interleukin-10 receptor subunit alpha measurement | Simple mode | 10 | -0.0642 | 0.437245 | 0.886511 | -0.9212 | 0.792805 | 0.937821 | 0.398043 | 2.209585 |
| 195 | GCST90274796 | ebi-a-GCST90018865 | interleukin-10 receptor subunit alpha measurement | Weighted mode | 10 | 0.520659 | 0.336051 | 0.155709 | -0.138 | 1.17932 | 1.683137 | 0.871097 | 3.252161 |
| 196 | GCST90274797 | ebi-a-GCST90018865 | interleukin-10 receptor subunit beta measurement | MR Egger | 18 | 0.042251 | 0.156956 | 0.791226 | -0.26538 | 0.349885 | 1.043156 | 0.766912 | 1.418904 |
| 197 | GCST90274797 | ebi-a-GCST90018865 | interleukin-10 receptor subunit beta measurement | Weighted median | 18 | 0.029993 | 0.12654 | 0.81264 | -0.21803 | 0.278012 | 1.030447 | 0.804104 | 1.320502 |
| 198 | GCST90274797 | ebi-a-GCST90018865 | interleukin-10 receptor subunit beta measurement | Inverse variance weighted | 18 | -0.0272 | 0.107586 | 0.800407 | -0.23807 | 0.183668 | 0.973167 | 0.78815 | 1.201617 |
| 199 | GCST90274797 | ebi-a-GCST90018865 | interleukin-10 receptor subunit beta measurement | Simple mode | 18 | -0.17878 | 0.396198 | 0.657519 | -0.95533 | 0.597767 | 0.836289 | 0.384686 | 1.818055 |
| 200 | GCST90274797 | ebi-a-GCST90018865 | interleukin-10 receptor subunit beta measurement | Weighted mode | 18 | 0.037874 | 0.13199 | 0.777624 | -0.22083 | 0.296574 | 1.0386 | 0.801856 | 1.345242 |
| 201 | GCST90274798 | ebi-a-GCST90018865 | interleukin-12 subunit B measurement | MR Egger | 27 | 0.379797 | 0.140441 | 0.012137 | 0.104532 | 0.655062 | 1.461988 | 1.110191 | 1.925262 |
| 202 | GCST90274798 | ebi-a-GCST90018865 | interleukin-12 subunit B measurement | Weighted median | 27 | 0.369542 | 0.10908 | 0.000704 | 0.155746 | 0.583338 | 1.447072 | 1.16853 | 1.79201 |
| 203 | GCST90274798 | ebi-a-GCST90018865 | interleukin-12 subunit B measurement | Inverse variance weighted | 27 | 0.329199 | 0.090193 | 0.000262 | 0.15242 | 0.505977 | 1.389854 | 1.164649 | 1.658605 |
| 204 | GCST90274798 | ebi-a-GCST90018865 | interleukin-12 subunit B measurement | Simple mode | 27 | 0.10586 | 0.294254 | 0.721933 | -0.47088 | 0.682597 | 1.111667 | 0.624455 | 1.979011 |
| 205 | GCST90274798 | ebi-a-GCST90018865 | interleukin-12 subunit B measurement | Weighted mode | 27 | 0.325396 | 0.101698 | 0.003606 | 0.126068 | 0.524724 | 1.384579 | 1.134359 | 1.689992 |
| 206 | GCST90274799 | ebi-a-GCST90018865 | interleukin-13 measurement | MR Egger | 13 | 0.480323 | 0.584272 | 0.428487 | -0.66485 | 1.625497 | 1.616597 | 0.514351 | 5.080941 |
| 207 | GCST90274799 | ebi-a-GCST90018865 | interleukin-13 measurement | Weighted median | 13 | 0.485798 | 0.299911 | 0.105273 | -0.10203 | 1.073623 | 1.625472 | 0.903005 | 2.925962 |
| 208 | GCST90274799 | ebi-a-GCST90018865 | interleukin-13 measurement | Inverse variance weighted | 13 | 0.160874 | 0.251018 | 0.521597 | -0.33112 | 0.652869 | 1.174537 | 0.718118 | 1.921044 |
| 209 | GCST90274799 | ebi-a-GCST90018865 | interleukin-13 measurement | Simple mode | 13 | 0.402805 | 0.528669 | 0.460819 | -0.63339 | 1.438996 | 1.496015 | 0.530792 | 4.216458 |
| 210 | GCST90274799 | ebi-a-GCST90018865 | interleukin-13 measurement | Weighted mode | 13 | 0.503546 | 0.443451 | 0.27833 | -0.36562 | 1.37271 | 1.654578 | 0.693767 | 3.94603 |
| 211 | GCST90274800 | ebi-a-GCST90018865 | interleukin-15 receptor subunit alpha measurement | MR Egger | 13 | -0.13 | 0.234998 | 0.591194 | -0.5906 | 0.330596 | 0.878095 | 0.553997 | 1.391797 |
| 212 | GCST90274800 | ebi-a-GCST90018865 | interleukin-15 receptor subunit alpha measurement | Weighted median | 13 | 0.029277 | 0.125213 | 0.815126 | -0.21614 | 0.274694 | 1.02971 | 0.805623 | 1.316128 |
| 213 | GCST90274800 | ebi-a-GCST90018865 | interleukin-15 receptor subunit alpha measurement | Inverse variance weighted | 13 | 0.01209 | 0.133887 | 0.928047 | -0.25033 | 0.274509 | 1.012164 | 0.778545 | 1.315885 |
| 214 | GCST90274800 | ebi-a-GCST90018865 | interleukin-15 receptor subunit alpha measurement | Simple mode | 13 | -0.01549 | 0.310423 | 0.961022 | -0.62392 | 0.592939 | 0.984629 | 0.53584 | 1.809298 |
| 215 | GCST90274800 | ebi-a-GCST90018865 | interleukin-15 receptor subunit alpha measurement | Weighted mode | 13 | 0.047625 | 0.124884 | 0.709606 | -0.19715 | 0.292398 | 1.048778 | 0.82107 | 1.339636 |
| 216 | GCST90274801 | ebi-a-GCST90018865 | Interleukin-17A measurement | MR Egger | 14 | -0.22914 | 0.611719 | 0.714507 | -1.42811 | 0.96983 | 0.795218 | 0.239762 | 2.637497 |
| 217 | GCST90274801 | ebi-a-GCST90018865 | Interleukin-17A measurement | Weighted median | 14 | -0.09999 | 0.322115 | 0.756254 | -0.73133 | 0.531361 | 0.90485 | 0.481268 | 1.701245 |
| 218 | GCST90274801 | ebi-a-GCST90018865 | Interleukin-17A measurement | Inverse variance weighted | 14 | -0.15363 | 0.228307 | 0.500995 | -0.60111 | 0.293848 | 0.857586 | 0.5482 | 1.34158 |
| 219 | GCST90274801 | ebi-a-GCST90018865 | Interleukin-17A measurement | Simple mode | 14 | 0.171765 | 0.598298 | 0.778569 | -1.0009 | 1.344428 | 1.187398 | 0.367549 | 3.835992 |
| 220 | GCST90274801 | ebi-a-GCST90018865 | Interleukin-17A measurement | Weighted mode | 14 | 0.171765 | 0.593897 | 0.776976 | -0.99227 | 1.335802 | 1.187398 | 0.370733 | 3.803045 |
| 221 | GCST90274802 | ebi-a-GCST90018865 | interleukin-17C measurement | MR Egger | 20 | 0.456403 | 0.585245 | 0.445614 | -0.69068 | 1.603484 | 1.578386 | 0.501236 | 4.970317 |
| 222 | GCST90274802 | ebi-a-GCST90018865 | interleukin-17C measurement | Weighted median | 20 | 0.060301 | 0.249076 | 0.808704 | -0.42789 | 0.548489 | 1.062156 | 0.651885 | 1.730636 |
| 223 | GCST90274802 | ebi-a-GCST90018865 | interleukin-17C measurement | Inverse variance weighted | 20 | 0.196597 | 0.200364 | 0.326494 | -0.19612 | 0.58931 | 1.217253 | 0.821917 | 1.802743 |
| 224 | GCST90274802 | ebi-a-GCST90018865 | interleukin-17C measurement | Simple mode | 20 | -0.19832 | 0.42531 | 0.646302 | -1.03193 | 0.635284 | 0.820105 | 0.356319 | 1.887559 |
| 225 | GCST90274802 | ebi-a-GCST90018865 | interleukin-17C measurement | Weighted mode | 20 | -0.02339 | 0.359341 | 0.948777 | -0.7277 | 0.680916 | 0.97688 | 0.483019 | 1.975686 |
| 226 | GCST90274803 | ebi-a-GCST90018865 | interleukin 18 measurement | MR Egger | 23 | 0.202884 | 0.29631 | 0.50102 | -0.37788 | 0.783651 | 1.22493 | 0.68531 | 2.189452 |
| 227 | GCST90274803 | ebi-a-GCST90018865 | interleukin 18 measurement | Weighted median | 23 | -0.09246 | 0.185804 | 0.618732 | -0.45664 | 0.271711 | 0.911681 | 0.633408 | 1.312207 |
| 228 | GCST90274803 | ebi-a-GCST90018865 | interleukin 18 measurement | Inverse variance weighted | 23 | -0.09552 | 0.137538 | 0.487348 | -0.3651 | 0.17405 | 0.908896 | 0.694128 | 1.190115 |
| 229 | GCST90274803 | ebi-a-GCST90018865 | interleukin 18 measurement | Simple mode | 23 | -0.03326 | 0.29238 | 0.910473 | -0.60632 | 0.539809 | 0.967291 | 0.545353 | 1.715679 |
| 230 | GCST90274803 | ebi-a-GCST90018865 | interleukin 18 measurement | Weighted mode | 23 | -0.09252 | 0.197133 | 0.643449 | -0.4789 | 0.293859 | 0.91163 | 0.619464 | 1.341595 |
| 231 | GCST90274804 | ebi-a-GCST90018865 | interleukin-18 receptor 1 measurement | MR Egger | 22 | -0.04456 | 0.196716 | 0.823082 | -0.43013 | 0.340999 | 0.956415 | 0.650427 | 1.406352 |
| 232 | GCST90274804 | ebi-a-GCST90018865 | interleukin-18 receptor 1 measurement | Weighted median | 22 | 0.002328 | 0.125422 | 0.985191 | -0.2435 | 0.248155 | 1.002331 | 0.78388 | 1.281659 |
| 233 | GCST90274804 | ebi-a-GCST90018865 | interleukin-18 receptor 1 measurement | Inverse variance weighted | 22 | 0.034056 | 0.101787 | 0.737942 | -0.16545 | 0.233559 | 1.034643 | 0.847515 | 1.263087 |
| 234 | GCST90274804 | ebi-a-GCST90018865 | interleukin-18 receptor 1 measurement | Simple mode | 22 | 0.252027 | 0.270455 | 0.361998 | -0.27806 | 0.782118 | 1.28663 | 0.757248 | 2.186097 |
| 235 | GCST90274804 | ebi-a-GCST90018865 | interleukin-18 receptor 1 measurement | Weighted mode | 22 | -0.02257 | 0.133218 | 0.867099 | -0.28367 | 0.238539 | 0.977685 | 0.753012 | 1.269393 |
| 236 | GCST90274805 | ebi-a-GCST90018865 | interleukin-1 alpha measurement | MR Egger | 14 | -0.32405 | 0.313524 | 0.321711 | -0.93856 | 0.290453 | 0.723211 | 0.39119 | 1.337033 |
| 237 | GCST90274805 | ebi-a-GCST90018865 | interleukin-1 alpha measurement | Weighted median | 14 | -0.28593 | 0.234707 | 0.223127 | -0.74596 | 0.174093 | 0.751313 | 0.47428 | 1.190166 |
| 238 | GCST90274805 | ebi-a-GCST90018865 | interleukin-1 alpha measurement | Inverse variance weighted | 14 | -0.373 | 0.171455 | 0.029594 | -0.70905 | -0.03694 | 0.688667 | 0.492112 | 0.963729 |
| 239 | GCST90274805 | ebi-a-GCST90018865 | interleukin-1 alpha measurement | Simple mode | 14 | -0.4851 | 0.403389 | 0.250591 | -1.27574 | 0.305541 | 0.615635 | 0.279223 | 1.357359 |
| 240 | GCST90274805 | ebi-a-GCST90018865 | interleukin-1 alpha measurement | Weighted mode | 14 | -0.16011 | 0.254727 | 0.540527 | -0.65938 | 0.339155 | 0.85205 | 0.517174 | 1.403761 |
| 241 | GCST90274806 | ebi-a-GCST90018865 | interleukin-2 measurement | MR Egger | 16 | -0.48858 | 0.536618 | 0.377981 | -1.54035 | 0.563194 | 0.613499 | 0.214306 | 1.756273 |
| 242 | GCST90274806 | ebi-a-GCST90018865 | interleukin-2 measurement | Weighted median | 16 | 0.229038 | 0.270667 | 0.397442 | -0.30147 | 0.759544 | 1.257389 | 0.739731 | 2.137301 |
| 243 | GCST90274806 | ebi-a-GCST90018865 | interleukin-2 measurement | Inverse variance weighted | 16 | 0.126822 | 0.195279 | 0.516053 | -0.25592 | 0.509568 | 1.135215 | 0.774201 | 1.664572 |
| 244 | GCST90274806 | ebi-a-GCST90018865 | interleukin-2 measurement | Simple mode | 16 | 0.310046 | 0.442181 | 0.493929 | -0.55663 | 1.176721 | 1.363488 | 0.573138 | 3.243721 |
| 245 | GCST90274806 | ebi-a-GCST90018865 | interleukin-2 measurement | Weighted mode | 16 | 0.321292 | 0.424908 | 0.461274 | -0.51153 | 1.154112 | 1.378908 | 0.599578 | 3.171207 |
| 246 | GCST90274807 | ebi-a-GCST90018865 | interleukin-20 measurement | MR Egger | 12 | 0.419115 | 0.575552 | 0.483196 | -0.70897 | 1.547197 | 1.520616 | 0.492153 | 4.698282 |
| 247 | GCST90274807 | ebi-a-GCST90018865 | interleukin-20 measurement | Weighted median | 12 | -0.0848 | 0.339074 | 0.802512 | -0.74939 | 0.579783 | 0.918695 | 0.472657 | 1.785651 |
| 248 | GCST90274807 | ebi-a-GCST90018865 | interleukin-20 measurement | Inverse variance weighted | 12 | -0.39863 | 0.308675 | 0.196556 | -1.00363 | 0.206372 | 0.671238 | 0.366545 | 1.229211 |
| 249 | GCST90274807 | ebi-a-GCST90018865 | interleukin-20 measurement | Simple mode | 12 | 0.021509 | 0.480061 | 0.965066 | -0.91941 | 0.962429 | 1.021742 | 0.398754 | 2.618047 |
| 250 | GCST90274807 | ebi-a-GCST90018865 | interleukin-20 measurement | Weighted mode | 12 | 0.021509 | 0.466869 | 0.96408 | -0.89355 | 0.936573 | 1.021742 | 0.409199 | 2.551223 |
| 251 | GCST90274808 | ebi-a-GCST90018865 | interleukin-20 receptor subunit alpha measurement | MR Egger | 12 | 0.063662 | 0.493059 | 0.899826 | -0.90273 | 1.030057 | 1.065732 | 0.40546 | 2.801224 |
| 252 | GCST90274808 | ebi-a-GCST90018865 | interleukin-20 receptor subunit alpha measurement | Weighted median | 12 | 0.201166 | 0.330366 | 0.542579 | -0.44635 | 0.848684 | 1.222828 | 0.639959 | 2.336569 |
| 253 | GCST90274808 | ebi-a-GCST90018865 | interleukin-20 receptor subunit alpha measurement | Inverse variance weighted | 12 | 0.162986 | 0.23136 | 0.481141 | -0.29048 | 0.616451 | 1.17702 | 0.747905 | 1.852342 |
| 254 | GCST90274808 | ebi-a-GCST90018865 | interleukin-20 receptor subunit alpha measurement | Simple mode | 12 | 0.253892 | 0.545567 | 0.650742 | -0.81542 | 1.323204 | 1.289033 | 0.442454 | 3.755434 |
| 255 | GCST90274808 | ebi-a-GCST90018865 | interleukin-20 receptor subunit alpha measurement | Weighted mode | 12 | 0.150063 | 0.51017 | 0.774124 | -0.84987 | 1.149996 | 1.161908 | 0.42747 | 3.158182 |
| 256 | GCST90274809 | ebi-a-GCST90018865 | interleukin-22 receptor subunit alpha-1 measurement | MR Egger | 12 | 0.271164 | 0.445517 | 0.556329 | -0.60205 | 1.144377 | 1.31149 | 0.547688 | 3.140485 |
| 257 | GCST90274809 | ebi-a-GCST90018865 | interleukin-22 receptor subunit alpha-1 measurement | Weighted median | 12 | 0.050228 | 0.308499 | 0.870665 | -0.55443 | 0.654887 | 1.051511 | 0.574399 | 1.924924 |
| 258 | GCST90274809 | ebi-a-GCST90018865 | interleukin-22 receptor subunit alpha-1 measurement | Inverse variance weighted | 12 | 0.175937 | 0.23459 | 0.453269 | -0.28386 | 0.635733 | 1.192363 | 0.752873 | 1.888406 |
| 259 | GCST90274809 | ebi-a-GCST90018865 | interleukin-22 receptor subunit alpha-1 measurement | Simple mode | 12 | 0.07813 | 0.552664 | 0.890134 | -1.00509 | 1.16135 | 1.081263 | 0.366011 | 3.194244 |
| 260 | GCST90274809 | ebi-a-GCST90018865 | interleukin-22 receptor subunit alpha-1 measurement | Weighted mode | 12 | 0.089679 | 0.439679 | 0.842106 | -0.77209 | 0.95145 | 1.093823 | 0.462045 | 2.589462 |
| 261 | GCST90274810 | ebi-a-GCST90018865 | interleukin-24 measurement | MR Egger | 12 | -0.41308 | 0.679156 | 0.556599 | -1.74423 | 0.918066 | 0.661609 | 0.17478 | 2.504442 |
| 262 | GCST90274810 | ebi-a-GCST90018865 | interleukin-24 measurement | Weighted median | 12 | -0.5597 | 0.335108 | 0.094876 | -1.21652 | 0.097108 | 0.571378 | 0.296261 | 1.101979 |
| 263 | GCST90274810 | ebi-a-GCST90018865 | interleukin-24 measurement | Inverse variance weighted | 12 | -0.32203 | 0.289216 | 0.265507 | -0.8889 | 0.24483 | 0.724674 | 0.411109 | 1.277404 |
| 264 | GCST90274810 | ebi-a-GCST90018865 | interleukin-24 measurement | Simple mode | 12 | -0.57328 | 0.47493 | 0.252717 | -1.50414 | 0.357583 | 0.563674 | 0.222208 | 1.42987 |
| 265 | GCST90274810 | ebi-a-GCST90018865 | interleukin-24 measurement | Weighted mode | 12 | -0.58462 | 0.430444 | 0.201613 | -1.42829 | 0.259054 | 0.55732 | 0.239719 | 1.295704 |
| 266 | GCST90274811 | ebi-a-GCST90018865 | interleukin-2 receptor subunit beta measurement | MR Egger | 11 | 0.050364 | 0.645308 | 0.939498 | -1.21444 | 1.315167 | 1.051654 | 0.296877 | 3.725374 |
| 267 | GCST90274811 | ebi-a-GCST90018865 | interleukin-2 receptor subunit beta measurement | Weighted median | 11 | 0.138159 | 0.373715 | 0.711612 | -0.59432 | 0.870642 | 1.148159 | 0.551936 | 2.388443 |
| 268 | GCST90274811 | ebi-a-GCST90018865 | interleukin-2 receptor subunit beta measurement | Inverse variance weighted | 11 | 0.135679 | 0.295839 | 0.646504 | -0.44417 | 0.715524 | 1.145314 | 0.641359 | 2.045257 |
| 269 | GCST90274811 | ebi-a-GCST90018865 | interleukin-2 receptor subunit beta measurement | Simple mode | 11 | 0.223216 | 0.688994 | 0.752632 | -1.12721 | 1.573645 | 1.250091 | 0.323935 | 4.824203 |
| 270 | GCST90274811 | ebi-a-GCST90018865 | interleukin-2 receptor subunit beta measurement | Weighted mode | 11 | 0.164969 | 0.707487 | 0.820328 | -1.2217 | 1.551643 | 1.179357 | 0.294727 | 4.719217 |
| 271 | GCST90274812 | ebi-a-GCST90018865 | level of interleukin-33 in blood plasma | MR Egger | 12 | 0.761191 | 0.609158 | 0.239908 | -0.43276 | 1.955141 | 2.140825 | 0.648717 | 7.064917 |
| 272 | GCST90274812 | ebi-a-GCST90018865 | level of interleukin-33 in blood plasma | Weighted median | 12 | -0.0014 | 0.298458 | 0.996268 | -0.58637 | 0.583581 | 0.998605 | 0.556342 | 1.792446 |
| 273 | GCST90274812 | ebi-a-GCST90018865 | level of interleukin-33 in blood plasma | Inverse variance weighted | 12 | 0.196172 | 0.22184 | 0.376537 | -0.23863 | 0.630979 | 1.216737 | 0.787703 | 1.87945 |
| 274 | GCST90274812 | ebi-a-GCST90018865 | level of interleukin-33 in blood plasma | Simple mode | 12 | -0.15997 | 0.496645 | 0.753409 | -1.1334 | 0.813451 | 0.852166 | 0.321937 | 2.255678 |
| 275 | GCST90274812 | ebi-a-GCST90018865 | level of interleukin-33 in blood plasma | Weighted mode | 12 | -0.15997 | 0.421608 | 0.711586 | -0.98632 | 0.666377 | 0.852166 | 0.372945 | 1.94717 |
| 276 | GCST90274813 | ebi-a-GCST90018865 | interleukin-4 measurement | MR Egger | 11 | 1.15329 | 0.917914 | 0.240593 | -0.64582 | 2.952401 | 3.1686 | 0.524232 | 19.15187 |
| 277 | GCST90274813 | ebi-a-GCST90018865 | interleukin-4 measurement | Weighted median | 11 | -0.02259 | 0.34421 | 0.947664 | -0.69725 | 0.652058 | 0.977659 | 0.497955 | 1.919487 |
| 278 | GCST90274813 | ebi-a-GCST90018865 | interleukin-4 measurement | Inverse variance weighted | 11 | -0.14083 | 0.355526 | 0.692014 | -0.83766 | 0.555998 | 0.868635 | 0.432721 | 1.74368 |
| 279 | GCST90274813 | ebi-a-GCST90018865 | interleukin-4 measurement | Simple mode | 11 | 0.037469 | 0.49599 | 0.941272 | -0.93467 | 1.00961 | 1.03818 | 0.392715 | 2.744532 |
| 280 | GCST90274813 | ebi-a-GCST90018865 | interleukin-4 measurement | Weighted mode | 11 | 0.051046 | 0.471945 | 0.916007 | -0.87397 | 0.976058 | 1.052372 | 0.417293 | 2.653974 |
| 281 | GCST90274814 | ebi-a-GCST90018865 | interleukin-5 measurement | MR Egger | 11 | -0.5359 | 0.536979 | 0.344355 | -1.58838 | 0.516574 | 0.58514 | 0.204256 | 1.676276 |
| 282 | GCST90274814 | ebi-a-GCST90018865 | interleukin-5 measurement | Weighted median | 11 | 0.036341 | 0.328015 | 0.911784 | -0.60657 | 0.67925 | 1.037009 | 0.545218 | 1.972399 |
| 283 | GCST90274814 | ebi-a-GCST90018865 | interleukin-5 measurement | Inverse variance weighted | 11 | -0.02028 | 0.242105 | 0.933233 | -0.49481 | 0.454242 | 0.979921 | 0.609688 | 1.574979 |
| 284 | GCST90274814 | ebi-a-GCST90018865 | interleukin-5 measurement | Simple mode | 11 | -0.00285 | 0.507955 | 0.995631 | -0.99844 | 0.99274 | 0.997153 | 0.368452 | 2.69862 |
| 285 | GCST90274814 | ebi-a-GCST90018865 | interleukin-5 measurement | Weighted mode | 11 | -0.00285 | 0.471381 | 0.995292 | -0.92676 | 0.921056 | 0.997153 | 0.395835 | 2.511941 |
| 286 | GCST90274815 | ebi-a-GCST90018865 | interleukin-6 measurement | MR Egger | 8 | -0.41157 | 0.541639 | 0.476127 | -1.47318 | 0.650045 | 0.662611 | 0.229196 | 1.915627 |
| 287 | GCST90274815 | ebi-a-GCST90018865 | interleukin-6 measurement | Weighted median | 8 | -0.09669 | 0.262074 | 0.712182 | -0.61035 | 0.41698 | 0.907841 | 0.54316 | 1.517372 |
| 288 | GCST90274815 | ebi-a-GCST90018865 | interleukin-6 measurement | Inverse variance weighted | 8 | -0.28613 | 0.256406 | 0.264448 | -0.78869 | 0.216423 | 0.751162 | 0.45444 | 1.241627 |
| 289 | GCST90274815 | ebi-a-GCST90018865 | interleukin-6 measurement | Simple mode | 8 | 0.091767 | 0.39247 | 0.821817 | -0.67748 | 0.861008 | 1.096109 | 0.507898 | 2.365544 |
| 290 | GCST90274815 | ebi-a-GCST90018865 | interleukin-6 measurement | Weighted mode | 8 | -0.0559 | 0.286184 | 0.850693 | -0.61682 | 0.505024 | 0.945637 | 0.539659 | 1.657026 |
| 291 | GCST90274816 | ebi-a-GCST90018865 | interleukin-7 measurement | MR Egger | 16 | 0.999634 | 0.764134 | 0.21188 | -0.49807 | 2.497337 | 2.717287 | 0.607703 | 12.15009 |
| 292 | GCST90274816 | ebi-a-GCST90018865 | interleukin-7 measurement | Weighted median | 16 | -0.28674 | 0.36054 | 0.426434 | -0.9934 | 0.419918 | 0.750707 | 0.370316 | 1.521836 |
| 293 | GCST90274816 | ebi-a-GCST90018865 | interleukin-7 measurement | Inverse variance weighted | 16 | -0.28213 | 0.306809 | 0.357809 | -0.88347 | 0.31922 | 0.754179 | 0.413345 | 1.376054 |
| 294 | GCST90274816 | ebi-a-GCST90018865 | interleukin-7 measurement | Simple mode | 16 | 0.601429 | 0.772799 | 0.448526 | -0.91326 | 2.116115 | 1.824725 | 0.401216 | 8.298834 |
| 295 | GCST90274816 | ebi-a-GCST90018865 | interleukin-7 measurement | Weighted mode | 16 | 0.273797 | 0.640657 | 0.675181 | -0.98189 | 1.529484 | 1.314948 | 0.374603 | 4.615796 |
| 296 | GCST90274817 | ebi-a-GCST90018865 | interleukin-8 measurement | MR Egger | 17 | -0.48632 | 0.510034 | 0.35545 | -1.48598 | 0.513348 | 0.614886 | 0.22628 | 1.670877 |
| 297 | GCST90274817 | ebi-a-GCST90018865 | interleukin-8 measurement | Weighted median | 17 | -0.06049 | 0.308777 | 0.844684 | -0.66569 | 0.544712 | 0.941302 | 0.513917 | 1.724112 |
| 298 | GCST90274817 | ebi-a-GCST90018865 | interleukin-8 measurement | Inverse variance weighted | 17 | -0.05891 | 0.253637 | 0.816338 | -0.55604 | 0.43822 | 0.942793 | 0.573477 | 1.549945 |
| 299 | GCST90274817 | ebi-a-GCST90018865 | interleukin-8 measurement | Simple mode | 17 | 0.457808 | 0.540328 | 0.409336 | -0.60124 | 1.516851 | 1.580605 | 0.548134 | 4.557852 |
| 300 | GCST90274817 | ebi-a-GCST90018865 | interleukin-8 measurement | Weighted mode | 17 | 0.279025 | 0.435919 | 0.531178 | -0.57538 | 1.133427 | 1.32184 | 0.562493 | 3.106282 |
| 301 | GCST90274818 | ebi-a-GCST90018865 | transforming growth factor beta-1 measurement | MR Egger | 19 | -0.1549 | 0.424202 | 0.719493 | -0.98634 | 0.676533 | 0.856498 | 0.372939 | 1.967046 |
| 302 | GCST90274818 | ebi-a-GCST90018865 | transforming growth factor beta-1 measurement | Weighted median | 19 | -0.23882 | 0.266361 | 0.369941 | -0.76088 | 0.283253 | 0.78756 | 0.467254 | 1.327441 |
| 303 | GCST90274818 | ebi-a-GCST90018865 | transforming growth factor beta-1 measurement | Inverse variance weighted | 19 | 0.023916 | 0.232537 | 0.918085 | -0.43186 | 0.479688 | 1.024204 | 0.649302 | 1.61557 |
| 304 | GCST90274818 | ebi-a-GCST90018865 | transforming growth factor beta-1 measurement | Simple mode | 19 | -0.01967 | 0.440403 | 0.964861 | -0.88286 | 0.843517 | 0.980519 | 0.413597 | 2.324528 |
| 305 | GCST90274818 | ebi-a-GCST90018865 | transforming growth factor beta-1 measurement | Weighted mode | 19 | -0.22919 | 0.328511 | 0.4943 | -0.87307 | 0.41469 | 0.795176 | 0.417666 | 1.513902 |
| 306 | GCST90274819 | ebi-a-GCST90018865 | leukemia inhibitory factor measurement | MR Egger | 12 | 0.425408 | 0.495615 | 0.410804 | -0.546 | 1.396813 | 1.530214 | 0.579264 | 4.042297 |
| 307 | GCST90274819 | ebi-a-GCST90018865 | leukemia inhibitory factor measurement | Weighted median | 12 | 0.47721 | 0.284025 | 0.092925 | -0.07948 | 1.033899 | 1.611571 | 0.923596 | 2.81201 |
| 308 | GCST90274819 | ebi-a-GCST90018865 | leukemia inhibitory factor measurement | Inverse variance weighted | 12 | 0.338577 | 0.224401 | 0.13135 | -0.10125 | 0.778403 | 1.402949 | 0.903708 | 2.17799 |
| 309 | GCST90274819 | ebi-a-GCST90018865 | leukemia inhibitory factor measurement | Simple mode | 12 | 0.449087 | 0.434506 | 0.323532 | -0.40255 | 1.30072 | 1.566881 | 0.668616 | 3.671938 |
| 310 | GCST90274819 | ebi-a-GCST90018865 | leukemia inhibitory factor measurement | Weighted mode | 12 | 0.46391 | 0.401233 | 0.27209 | -0.32251 | 1.250327 | 1.590279 | 0.72433 | 3.491483 |
| 311 | GCST90274820 | ebi-a-GCST90018865 | leukemia inhibitory factor receptor measurement | MR Egger | 18 | 0.258997 | 0.266699 | 0.345941 | -0.26373 | 0.781728 | 1.29563 | 0.768178 | 2.185246 |
| 312 | GCST90274820 | ebi-a-GCST90018865 | leukemia inhibitory factor receptor measurement | Weighted median | 18 | 0.225159 | 0.197406 | 0.25404 | -0.16176 | 0.612074 | 1.252522 | 0.850649 | 1.844253 |
| 313 | GCST90274820 | ebi-a-GCST90018865 | leukemia inhibitory factor receptor measurement | Inverse variance weighted | 18 | 0.058585 | 0.144994 | 0.686176 | -0.2256 | 0.342773 | 1.060335 | 0.798035 | 1.408848 |
| 314 | GCST90274820 | ebi-a-GCST90018865 | leukemia inhibitory factor receptor measurement | Simple mode | 18 | 0.300841 | 0.393252 | 0.454757 | -0.46993 | 1.071616 | 1.350995 | 0.625044 | 2.920094 |
| 315 | GCST90274820 | ebi-a-GCST90018865 | leukemia inhibitory factor receptor measurement | Weighted mode | 18 | 0.238943 | 0.202946 | 0.255267 | -0.15883 | 0.636717 | 1.269906 | 0.853141 | 1.890265 |
| 316 | GCST90274821 | ebi-a-GCST90018865 | CCL2 measurement | MR Egger | 23 | -0.16622 | 0.23466 | 0.486519 | -0.62615 | 0.293712 | 0.84686 | 0.534645 | 1.341398 |
| 317 | GCST90274821 | ebi-a-GCST90018865 | CCL2 measurement | Weighted median | 23 | 0.005245 | 0.189648 | 0.977934 | -0.36646 | 0.376954 | 1.005259 | 0.693181 | 1.457838 |
| 318 | GCST90274821 | ebi-a-GCST90018865 | CCL2 measurement | Inverse variance weighted | 23 | -0.11443 | 0.131092 | 0.382729 | -0.37137 | 0.142512 | 0.891877 | 0.689791 | 1.153167 |
| 319 | GCST90274821 | ebi-a-GCST90018865 | CCL2 measurement | Simple mode | 23 | 0.091488 | 0.322942 | 0.779599 | -0.54148 | 0.724454 | 1.095803 | 0.581888 | 2.063603 |
| 320 | GCST90274821 | ebi-a-GCST90018865 | CCL2 measurement | Weighted mode | 23 | 0.097276 | 0.25887 | 0.710687 | -0.41011 | 0.604662 | 1.102164 | 0.663577 | 1.830633 |
| 321 | GCST90274822 | ebi-a-GCST90018865 | monocyte chemotactic protein-2 measurement | MR Egger | 30 | 0.110772 | 0.089794 | 0.227599 | -0.06522 | 0.286769 | 1.11714 | 0.936857 | 1.332117 |
| 322 | GCST90274822 | ebi-a-GCST90018865 | monocyte chemotactic protein-2 measurement | Weighted median | 30 | 0.073838 | 0.09203 | 0.422365 | -0.10654 | 0.254218 | 1.076633 | 0.898938 | 1.289453 |
| 323 | GCST90274822 | ebi-a-GCST90018865 | monocyte chemotactic protein-2 measurement | Inverse variance weighted | 30 | 0.053331 | 0.071985 | 0.458778 | -0.08776 | 0.194421 | 1.054778 | 0.915981 | 1.214607 |
| 324 | GCST90274822 | ebi-a-GCST90018865 | monocyte chemotactic protein-2 measurement | Simple mode | 30 | 0.024476 | 0.154495 | 0.875221 | -0.27834 | 0.327287 | 1.024778 | 0.757043 | 1.387199 |
| 325 | GCST90274822 | ebi-a-GCST90018865 | monocyte chemotactic protein-2 measurement | Weighted mode | 30 | 0.067891 | 0.081845 | 0.413594 | -0.09252 | 0.228306 | 1.070248 | 0.911627 | 1.25647 |
| 326 | GCST90274823 | ebi-a-GCST90018865 | monocyte chemotactic protein 3 measurement | MR Egger | 19 | 0.179033 | 0.357483 | 0.622924 | -0.52163 | 0.8797 | 1.19606 | 0.593549 | 2.410177 |
| 327 | GCST90274823 | ebi-a-GCST90018865 | monocyte chemotactic protein 3 measurement | Weighted median | 19 | 0.012306 | 0.219195 | 0.95523 | -0.41732 | 0.441929 | 1.012382 | 0.658812 | 1.555705 |
| 328 | GCST90274823 | ebi-a-GCST90018865 | monocyte chemotactic protein 3 measurement | Inverse variance weighted | 19 | 0.17282 | 0.15657 | 0.269688 | -0.13406 | 0.479697 | 1.188652 | 0.874539 | 1.615586 |
| 329 | GCST90274823 | ebi-a-GCST90018865 | monocyte chemotactic protein 3 measurement | Simple mode | 19 | 0.142993 | 0.404292 | 0.727683 | -0.64942 | 0.935405 | 1.153721 | 0.522349 | 2.548245 |
| 330 | GCST90274823 | ebi-a-GCST90018865 | monocyte chemotactic protein 3 measurement | Weighted mode | 19 | 0.0025 | 0.278154 | 0.992927 | -0.54268 | 0.547682 | 1.002503 | 0.581188 | 1.72924 |
| 331 | GCST90274824 | ebi-a-GCST90018865 | monocyte chemotactic protein-4 measurement | MR Egger | 19 | 0.414542 | 0.250118 | 0.115779 | -0.07569 | 0.904774 | 1.513678 | 0.927104 | 2.471374 |
| 332 | GCST90274824 | ebi-a-GCST90018865 | monocyte chemotactic protein-4 measurement | Weighted median | 19 | 0.152157 | 0.156604 | 0.331249 | -0.15479 | 0.459101 | 1.164343 | 0.856597 | 1.582651 |
| 333 | GCST90274824 | ebi-a-GCST90018865 | monocyte chemotactic protein-4 measurement | Inverse variance weighted | 19 | 0.174263 | 0.12547 | 0.164869 | -0.07166 | 0.420184 | 1.190369 | 0.930849 | 1.522242 |
| 334 | GCST90274824 | ebi-a-GCST90018865 | monocyte chemotactic protein-4 measurement | Simple mode | 19 | 0.288946 | 0.235091 | 0.234867 | -0.17183 | 0.749724 | 1.33502 | 0.842121 | 2.116416 |
| 335 | GCST90274824 | ebi-a-GCST90018865 | monocyte chemotactic protein-4 measurement | Weighted mode | 19 | 0.196722 | 0.144895 | 0.191341 | -0.08727 | 0.480716 | 1.217406 | 0.916428 | 1.617232 |
| 336 | GCST90274825 | ebi-a-GCST90018865 | macrophage inflammatory protein 1a measurement | MR Egger | 15 | 0.204723 | 0.289005 | 0.491222 | -0.36173 | 0.771173 | 1.227185 | 0.696473 | 2.162301 |
| 337 | GCST90274825 | ebi-a-GCST90018865 | macrophage inflammatory protein 1a measurement | Weighted median | 15 | 0.20859 | 0.142601 | 0.143535 | -0.07091 | 0.488089 | 1.23194 | 0.931547 | 1.629199 |
| 338 | GCST90274825 | ebi-a-GCST90018865 | macrophage inflammatory protein 1a measurement | Inverse variance weighted | 15 | 0.165711 | 0.183764 | 0.367184 | -0.19447 | 0.525887 | 1.180232 | 0.823274 | 1.691959 |
| 339 | GCST90274825 | ebi-a-GCST90018865 | macrophage inflammatory protein 1a measurement | Simple mode | 15 | -0.94259 | 0.635152 | 0.159962 | -2.18749 | 0.302308 | 0.389618 | 0.112198 | 1.352978 |
| 340 | GCST90274825 | ebi-a-GCST90018865 | macrophage inflammatory protein 1a measurement | Weighted mode | 15 | 0.231815 | 0.153892 | 0.154205 | -0.06981 | 0.533444 | 1.260887 | 0.932568 | 1.704794 |
| 341 | GCST90274826 | ebi-a-GCST90018865 | matrix metalloproteinase 1 measurement | MR Egger | 16 | 0.037207 | 0.327378 | 0.911129 | -0.60445 | 0.678868 | 1.037908 | 0.546372 | 1.971645 |
| 342 | GCST90274826 | ebi-a-GCST90018865 | matrix metalloproteinase 1 measurement | Weighted median | 16 | -0.23142 | 0.214087 | 0.27972 | -0.65103 | 0.188194 | 0.793408 | 0.521509 | 1.207067 |
| 343 | GCST90274826 | ebi-a-GCST90018865 | matrix metalloproteinase 1 measurement | Inverse variance weighted | 16 | -0.10931 | 0.17149 | 0.523843 | -0.44543 | 0.226807 | 0.896449 | 0.640547 | 1.254587 |
| 344 | GCST90274826 | ebi-a-GCST90018865 | matrix metalloproteinase 1 measurement | Simple mode | 16 | 0.147859 | 0.352163 | 0.680539 | -0.54238 | 0.838098 | 1.15935 | 0.581363 | 2.311965 |
| 345 | GCST90274826 | ebi-a-GCST90018865 | matrix metalloproteinase 1 measurement | Weighted mode | 16 | -0.19354 | 0.287223 | 0.510671 | -0.7565 | 0.369418 | 0.824038 | 0.469308 | 1.446892 |
| 346 | GCST90274827 | ebi-a-GCST90018865 | matrix metalloproteinase 10 measurement | MR Egger | 16 | 0.15892 | 0.178665 | 0.388773 | -0.19126 | 0.509104 | 1.172244 | 0.825914 | 1.6638 |
| 347 | GCST90274827 | ebi-a-GCST90018865 | matrix metalloproteinase 10 measurement | Weighted median | 16 | 0.222282 | 0.16427 | 0.176009 | -0.09969 | 0.544252 | 1.248924 | 0.90512 | 1.723319 |
| 348 | GCST90274827 | ebi-a-GCST90018865 | matrix metalloproteinase 10 measurement | Inverse variance weighted | 16 | 0.13932 | 0.130078 | 0.284148 | -0.11563 | 0.394272 | 1.149492 | 0.890802 | 1.483304 |
| 349 | GCST90274827 | ebi-a-GCST90018865 | matrix metalloproteinase 10 measurement | Simple mode | 16 | 0.114281 | 0.328896 | 0.733066 | -0.53035 | 0.758917 | 1.121068 | 0.588396 | 2.135963 |
| 350 | GCST90274827 | ebi-a-GCST90018865 | matrix metalloproteinase 10 measurement | Weighted mode | 16 | 0.221152 | 0.16582 | 0.202206 | -0.10386 | 0.546159 | 1.247513 | 0.901355 | 1.726609 |
| 351 | GCST90274828 | ebi-a-GCST90018865 | level of neurturin in blood plasma | MR Egger | 14 | -0.31927 | 0.39111 | 0.43023 | -1.08584 | 0.447309 | 0.726682 | 0.337617 | 1.564098 |
| 352 | GCST90274828 | ebi-a-GCST90018865 | level of neurturin in blood plasma | Weighted median | 14 | -0.15253 | 0.272428 | 0.57556 | -0.68649 | 0.381432 | 0.858535 | 0.503341 | 1.46438 |
| 353 | GCST90274828 | ebi-a-GCST90018865 | level of neurturin in blood plasma | Inverse variance weighted | 14 | -0.23006 | 0.203281 | 0.257751 | -0.62849 | 0.168373 | 0.794488 | 0.533398 | 1.183378 |
| 354 | GCST90274828 | ebi-a-GCST90018865 | level of neurturin in blood plasma | Simple mode | 14 | -0.15833 | 0.475538 | 0.744482 | -1.09038 | 0.773725 | 0.853569 | 0.336088 | 2.167826 |
| 355 | GCST90274828 | ebi-a-GCST90018865 | level of neurturin in blood plasma | Weighted mode | 14 | -0.10092 | 0.419517 | 0.813649 | -0.92317 | 0.721335 | 0.904007 | 0.397257 | 2.057178 |
| 356 | GCST90274829 | ebi-a-GCST90018865 | neurotrophin-3 measurement | MR Egger | 18 | -0.74202 | 0.55402 | 0.19918 | -1.8279 | 0.343864 | 0.476153 | 0.160751 | 1.410387 |
| 357 | GCST90274829 | ebi-a-GCST90018865 | neurotrophin-3 measurement | Weighted median | 18 | -0.56589 | 0.296865 | 0.056621 | -1.14775 | 0.015963 | 0.567854 | 0.317351 | 1.016091 |
| 358 | GCST90274829 | ebi-a-GCST90018865 | neurotrophin-3 measurement | Inverse variance weighted | 18 | -0.3724 | 0.236175 | 0.114841 | -0.8353 | 0.090501 | 0.689078 | 0.433743 | 1.094723 |
| 359 | GCST90274829 | ebi-a-GCST90018865 | neurotrophin-3 measurement | Simple mode | 18 | -0.83347 | 0.532241 | 0.13578 | -1.87666 | 0.20972 | 0.434538 | 0.1531 | 1.233332 |
| 360 | GCST90274829 | ebi-a-GCST90018865 | neurotrophin-3 measurement | Weighted mode | 18 | -0.84599 | 0.572689 | 0.157903 | -1.96846 | 0.276484 | 0.429134 | 0.139672 | 1.318486 |
| 361 | GCST90274830 | ebi-a-GCST90018865 | osteoprotegerin measurement | MR Egger | 21 | 0.233352 | 0.331013 | 0.489385 | -0.41543 | 0.882137 | 1.262826 | 0.660055 | 2.416058 |
| 362 | GCST90274830 | ebi-a-GCST90018865 | osteoprotegerin measurement | Weighted median | 21 | -0.05649 | 0.208511 | 0.786446 | -0.46517 | 0.35219 | 0.945074 | 0.628026 | 1.422179 |
| 363 | GCST90274830 | ebi-a-GCST90018865 | osteoprotegerin measurement | Inverse variance weighted | 21 | 0.176669 | 0.153446 | 0.249593 | -0.12409 | 0.477423 | 1.193236 | 0.883304 | 1.611916 |
| 364 | GCST90274830 | ebi-a-GCST90018865 | osteoprotegerin measurement | Simple mode | 21 | 0.027513 | 0.372162 | 0.941803 | -0.70192 | 0.756949 | 1.027894 | 0.495631 | 2.131763 |
| 365 | GCST90274830 | ebi-a-GCST90018865 | osteoprotegerin measurement | Weighted mode | 21 | 0.027513 | 0.22697 | 0.904729 | -0.41735 | 0.472374 | 1.027894 | 0.658791 | 1.603797 |
| 366 | GCST90274831 | ebi-a-GCST90018865 | oncostatin-M measurement | MR Egger | 17 | -0.21468 | 0.537 | 0.694955 | -1.2672 | 0.837839 | 0.806799 | 0.281619 | 2.311368 |
| 367 | GCST90274831 | ebi-a-GCST90018865 | oncostatin-M measurement | Weighted median | 17 | -0.14044 | 0.256471 | 0.583986 | -0.64312 | 0.362247 | 0.868979 | 0.52565 | 1.436553 |
| 368 | GCST90274831 | ebi-a-GCST90018865 | oncostatin-M measurement | Inverse variance weighted | 17 | 0.018626 | 0.211597 | 0.929855 | -0.3961 | 0.433356 | 1.018801 | 0.672937 | 1.542425 |
| 369 | GCST90274831 | ebi-a-GCST90018865 | oncostatin-M measurement | Simple mode | 17 | -0.32648 | 0.450902 | 0.479488 | -1.21025 | 0.557292 | 0.721461 | 0.298124 | 1.745937 |
| 370 | GCST90274831 | ebi-a-GCST90018865 | oncostatin-M measurement | Weighted mode | 17 | -0.43201 | 0.374699 | 0.265869 | -1.16642 | 0.302404 | 0.649205 | 0.311481 | 1.353108 |
| 371 | GCST90274832 | ebi-a-GCST90018865 | programmed cell death 1 ligand 1 measurement | MR Egger | 19 | -0.18777 | 0.536699 | 0.730736 | -1.2397 | 0.864157 | 0.828803 | 0.28947 | 2.373006 |
| 372 | GCST90274832 | ebi-a-GCST90018865 | programmed cell death 1 ligand 1 measurement | Weighted median | 19 | -0.04283 | 0.264184 | 0.871198 | -0.56063 | 0.474966 | 0.95807 | 0.570847 | 1.60796 |
| 373 | GCST90274832 | ebi-a-GCST90018865 | programmed cell death 1 ligand 1 measurement | Inverse variance weighted | 19 | -0.09999 | 0.222361 | 0.652931 | -0.53582 | 0.335833 | 0.904842 | 0.585188 | 1.399106 |
| 374 | GCST90274832 | ebi-a-GCST90018865 | programmed cell death 1 ligand 1 measurement | Simple mode | 19 | -0.04274 | 0.463613 | 0.927566 | -0.95142 | 0.865942 | 0.95816 | 0.386191 | 2.377244 |
| 375 | GCST90274832 | ebi-a-GCST90018865 | programmed cell death 1 ligand 1 measurement | Weighted mode | 19 | -0.11931 | 0.375391 | 0.754268 | -0.85508 | 0.616454 | 0.887531 | 0.42525 | 1.852348 |
| 376 | GCST90274833 | ebi-a-GCST90018865 | stem Cell Factor measurement | MR Egger | 33 | 0.50644 | 0.197885 | 0.01559 | 0.118585 | 0.894295 | 1.659373 | 1.125902 | 2.445612 |
| 377 | GCST90274833 | ebi-a-GCST90018865 | stem Cell Factor measurement | Weighted median | 33 | 0.272286 | 0.16611 | 0.101172 | -0.05329 | 0.597861 | 1.312963 | 0.948106 | 1.818225 |
| 378 | GCST90274833 | ebi-a-GCST90018865 | stem Cell Factor measurement | Inverse variance weighted | 33 | 0.240561 | 0.108595 | 0.026746 | 0.027714 | 0.453407 | 1.271962 | 1.028102 | 1.573665 |
| 379 | GCST90274833 | ebi-a-GCST90018865 | stem Cell Factor measurement | Simple mode | 33 | 0.324123 | 0.238099 | 0.182929 | -0.14255 | 0.790797 | 1.382818 | 0.867144 | 2.205154 |
| 380 | GCST90274833 | ebi-a-GCST90018865 | stem Cell Factor measurement | Weighted mode | 33 | 0.305392 | 0.172066 | 0.085435 | -0.03186 | 0.642641 | 1.357157 | 0.968646 | 1.901496 |
| 381 | GCST90274834 | ebi-a-GCST90018865 | SIR2-like protein 2 measurement | MR Egger | 13 | 0.239915 | 0.453974 | 0.607665 | -0.64987 | 1.129705 | 1.271142 | 0.522111 | 3.094745 |
| 382 | GCST90274834 | ebi-a-GCST90018865 | SIR2-like protein 2 measurement | Weighted median | 13 | 0.167908 | 0.314427 | 0.593332 | -0.44837 | 0.784184 | 1.182828 | 0.63867 | 2.190619 |
| 383 | GCST90274834 | ebi-a-GCST90018865 | SIR2-like protein 2 measurement | Inverse variance weighted | 13 | 0.368911 | 0.226091 | 0.102743 | -0.07423 | 0.812049 | 1.44616 | 0.928462 | 2.252519 |
| 384 | GCST90274834 | ebi-a-GCST90018865 | SIR2-like protein 2 measurement | Simple mode | 13 | -0.02003 | 0.525844 | 0.970244 | -1.05068 | 1.010625 | 0.980171 | 0.349699 | 2.747318 |
| 385 | GCST90274834 | ebi-a-GCST90018865 | SIR2-like protein 2 measurement | Weighted mode | 13 | 0.081791 | 0.464232 | 0.863086 | -0.8281 | 0.991686 | 1.085229 | 0.436877 | 2.695774 |
| 386 | GCST90274835 | ebi-a-GCST90018865 | signaling lymphocytic activation molecule measurement | MR Egger | 28 | 0.194643 | 0.347347 | 0.580023 | -0.48616 | 0.875443 | 1.214878 | 0.614986 | 2.399939 |
| 387 | GCST90274835 | ebi-a-GCST90018865 | signaling lymphocytic activation molecule measurement | Weighted median | 28 | 0.033126 | 0.223518 | 0.882184 | -0.40497 | 0.471221 | 1.03368 | 0.666997 | 1.601948 |
| 388 | GCST90274835 | ebi-a-GCST90018865 | signaling lymphocytic activation molecule measurement | Inverse variance weighted | 28 | 0.159458 | 0.154353 | 0.301569 | -0.14307 | 0.461989 | 1.172875 | 0.86669 | 1.587228 |
| 389 | GCST90274835 | ebi-a-GCST90018865 | signaling lymphocytic activation molecule measurement | Simple mode | 28 | 0.028907 | 0.455061 | 0.949817 | -0.86301 | 0.920826 | 1.029329 | 0.42189 | 2.511365 |
| 390 | GCST90274835 | ebi-a-GCST90018865 | signaling lymphocytic activation molecule measurement | Weighted mode | 28 | 0.039578 | 0.452554 | 0.930956 | -0.84743 | 0.926584 | 1.040371 | 0.428516 | 2.525866 |
| 391 | GCST90274836 | ebi-a-GCST90018865 | sulfotrasferase 1A1 measurement | MR Egger | 23 | -0.43725 | 0.363251 | 0.2421 | -1.14922 | 0.274728 | 0.645813 | 0.316885 | 1.316172 |
| 392 | GCST90274836 | ebi-a-GCST90018865 | sulfotrasferase 1A1 measurement | Weighted median | 23 | -0.1993 | 0.203527 | 0.327453 | -0.59822 | 0.199607 | 0.8193 | 0.549791 | 1.220923 |
| 393 | GCST90274836 | ebi-a-GCST90018865 | sulfotrasferase 1A1 measurement | Inverse variance weighted | 23 | -0.22798 | 0.148813 | 0.125527 | -0.51965 | 0.063695 | 0.796141 | 0.594727 | 1.065767 |
| 394 | GCST90274836 | ebi-a-GCST90018865 | sulfotrasferase 1A1 measurement | Simple mode | 23 | 0.05634 | 0.393537 | 0.887464 | -0.71499 | 0.827673 | 1.057957 | 0.489195 | 2.287989 |
| 395 | GCST90274836 | ebi-a-GCST90018865 | sulfotrasferase 1A1 measurement | Weighted mode | 23 | -0.16162 | 0.272757 | 0.559535 | -0.69622 | 0.372986 | 0.850766 | 0.498465 | 1.452064 |
| 396 | GCST90274837 | ebi-a-GCST90018865 | STAM binding protein measurement | MR Egger | 12 | -0.90403 | 0.703796 | 0.227924 | -2.28347 | 0.475412 | 0.404935 | 0.10193 | 1.608677 |
| 397 | GCST90274837 | ebi-a-GCST90018865 | STAM binding protein measurement | Weighted median | 12 | 0.015679 | 0.370057 | 0.966204 | -0.70963 | 0.740991 | 1.015803 | 0.491825 | 2.098013 |
| 398 | GCST90274837 | ebi-a-GCST90018865 | STAM binding protein measurement | Inverse variance weighted | 12 | 0.174875 | 0.267555 | 0.513367 | -0.34953 | 0.699282 | 1.191097 | 0.705018 | 2.012307 |
| 399 | GCST90274837 | ebi-a-GCST90018865 | STAM binding protein measurement | Simple mode | 12 | 0.22866 | 0.723755 | 0.757965 | -1.1899 | 1.647219 | 1.256914 | 0.304252 | 5.19252 |
| 400 | GCST90274837 | ebi-a-GCST90018865 | STAM binding protein measurement | Weighted mode | 12 | -0.28494 | 0.584609 | 0.635543 | -1.43077 | 0.860895 | 0.752061 | 0.239125 | 2.365276 |
| 401 | GCST90274838 | ebi-a-GCST90018865 | transforming growth factor-alpha measurement | MR Egger | 14 | -0.48193 | 0.50911 | 0.362504 | -1.47979 | 0.515921 | 0.617588 | 0.227686 | 1.67518 |
| 402 | GCST90274838 | ebi-a-GCST90018865 | transforming growth factor-alpha measurement | Weighted median | 14 | -0.30436 | 0.30434 | 0.317273 | -0.90087 | 0.292143 | 0.737592 | 0.406216 | 1.339294 |
| 403 | GCST90274838 | ebi-a-GCST90018865 | transforming growth factor-alpha measurement | Inverse variance weighted | 14 | 0.028927 | 0.21757 | 0.894229 | -0.39751 | 0.455365 | 1.02935 | 0.671991 | 1.576748 |
| 404 | GCST90274838 | ebi-a-GCST90018865 | transforming growth factor-alpha measurement | Simple mode | 14 | -0.43485 | 0.525522 | 0.422903 | -1.46487 | 0.595172 | 0.647361 | 0.231107 | 1.813342 |
| 405 | GCST90274838 | ebi-a-GCST90018865 | transforming growth factor-alpha measurement | Weighted mode | 14 | -0.46072 | 0.520425 | 0.392089 | -1.48075 | 0.559314 | 0.63083 | 0.227466 | 1.749471 |
| 406 | GCST90274839 | ebi-a-GCST90018865 | tumor necrosis factor measurement | MR Egger | 18 | 0.07836 | 0.465506 | 0.86843 | -0.83403 | 0.990753 | 1.081512 | 0.434295 | 2.693262 |
| 407 | GCST90274839 | ebi-a-GCST90018865 | tumor necrosis factor measurement | Weighted median | 18 | 0.266742 | 0.268888 | 0.321189 | -0.26028 | 0.793762 | 1.305703 | 0.770836 | 2.211702 |
| 408 | GCST90274839 | ebi-a-GCST90018865 | tumor necrosis factor measurement | Inverse variance weighted | 18 | 0.064243 | 0.204617 | 0.753547 | -0.33681 | 0.465293 | 1.066351 | 0.714046 | 1.592481 |
| 409 | GCST90274839 | ebi-a-GCST90018865 | tumor necrosis factor measurement | Simple mode | 18 | 0.245883 | 0.501074 | 0.629904 | -0.73622 | 1.227988 | 1.27875 | 0.47892 | 3.414354 |
| 410 | GCST90274839 | ebi-a-GCST90018865 | tumor necrosis factor measurement | Weighted mode | 18 | 0.330937 | 0.430261 | 0.452356 | -0.51237 | 1.174248 | 1.392273 | 0.599072 | 3.235709 |
| 411 | GCST90274840 | ebi-a-GCST90018865 | lymphotoxin-alpha measurement | MR Egger | 24 | 0.003481 | 0.138834 | 0.980223 | -0.26863 | 0.275595 | 1.003487 | 0.764423 | 1.317315 |
| 412 | GCST90274840 | ebi-a-GCST90018865 | lymphotoxin-alpha measurement | Weighted median | 24 | 0.03715 | 0.106454 | 0.72711 | -0.1715 | 0.2458 | 1.037848 | 0.842399 | 1.278644 |
| 413 | GCST90274840 | ebi-a-GCST90018865 | lymphotoxin-alpha measurement | Inverse variance weighted | 24 | 0.075334 | 0.089635 | 0.400653 | -0.10035 | 0.251018 | 1.078244 | 0.904521 | 1.285333 |
| 414 | GCST90274840 | ebi-a-GCST90018865 | lymphotoxin-alpha measurement | Simple mode | 24 | 0.0486 | 0.23743 | 0.839613 | -0.41676 | 0.513963 | 1.0498 | 0.659177 | 1.671904 |
| 415 | GCST90274840 | ebi-a-GCST90018865 | lymphotoxin-alpha measurement | Weighted mode | 24 | 0.032847 | 0.103839 | 0.75461 | -0.17068 | 0.236372 | 1.033392 | 0.843093 | 1.266645 |
| 416 | GCST90274841 | ebi-a-GCST90018865 | tumor necrosis factor receptor superfamily member 9 measurement | MR Egger | 28 | 0.292285 | 0.339785 | 0.397538 | -0.37369 | 0.958262 | 1.339484 | 0.688188 | 2.607162 |
| 417 | GCST90274841 | ebi-a-GCST90018865 | tumor necrosis factor receptor superfamily member 9 measurement | Weighted median | 28 | 0.285027 | 0.199947 | 0.15401 | -0.10687 | 0.676924 | 1.329798 | 0.898643 | 1.967815 |
| 418 | GCST90274841 | ebi-a-GCST90018865 | tumor necrosis factor receptor superfamily member 9 measurement | Inverse variance weighted | 28 | 0.275447 | 0.14077 | 0.050381 | -0.00046 | 0.551356 | 1.31712 | 0.999538 | 1.735605 |
| 419 | GCST90274841 | ebi-a-GCST90018865 | tumor necrosis factor receptor superfamily member 9 measurement | Simple mode | 28 | 0.500705 | 0.391063 | 0.211306 | -0.26578 | 1.267188 | 1.649884 | 0.766609 | 3.550854 |
| 420 | GCST90274841 | ebi-a-GCST90018865 | tumor necrosis factor receptor superfamily member 9 measurement | Weighted mode | 28 | 0.3577 | 0.341473 | 0.304146 | -0.31159 | 1.026987 | 1.430037 | 0.732284 | 2.792638 |
| 421 | GCST90274842 | ebi-a-GCST90018865 | tumor necrosis factor ligand superfamily member 14 measurement | MR Egger | 26 | 0.010845 | 0.234182 | 0.963446 | -0.44815 | 0.469842 | 1.010904 | 0.638807 | 1.599742 |
| 422 | GCST90274842 | ebi-a-GCST90018865 | tumor necrosis factor ligand superfamily member 14 measurement | Weighted median | 26 | -0.04 | 0.181452 | 0.825534 | -0.39564 | 0.315648 | 0.960791 | 0.673246 | 1.371148 |
| 423 | GCST90274842 | ebi-a-GCST90018865 | tumor necrosis factor ligand superfamily member 14 measurement | Inverse variance weighted | 26 | -0.13414 | 0.144425 | 0.352984 | -0.41722 | 0.148928 | 0.874464 | 0.658878 | 1.16059 |
| 424 | GCST90274842 | ebi-a-GCST90018865 | tumor necrosis factor ligand superfamily member 14 measurement | Simple mode | 26 | 0.14669 | 0.344713 | 0.674083 | -0.52895 | 0.822327 | 1.157995 | 0.589225 | 2.27579 |
| 425 | GCST90274842 | ebi-a-GCST90018865 | tumor necrosis factor ligand superfamily member 14 measurement | Weighted mode | 26 | 0.066084 | 0.196681 | 0.739678 | -0.31941 | 0.451578 | 1.068316 | 0.726577 | 1.570789 |
| 426 | GCST90274843 | ebi-a-GCST90018865 | TNF-related apoptosis-inducing ligand measurement | MR Egger | 25 | -0.07392 | 0.177719 | 0.68133 | -0.42224 | 0.274414 | 0.92875 | 0.655574 | 1.315759 |
| 427 | GCST90274843 | ebi-a-GCST90018865 | TNF-related apoptosis-inducing ligand measurement | Weighted median | 25 | 0.038554 | 0.167185 | 0.81762 | -0.28913 | 0.366236 | 1.039307 | 0.748916 | 1.442295 |
| 428 | GCST90274843 | ebi-a-GCST90018865 | TNF-related apoptosis-inducing ligand measurement | Inverse variance weighted | 25 | 0.0167 | 0.108793 | 0.878004 | -0.19654 | 0.229935 | 1.01684 | 0.821573 | 1.258518 |
| 429 | GCST90274843 | ebi-a-GCST90018865 | TNF-related apoptosis-inducing ligand measurement | Simple mode | 25 | 0.293347 | 0.331443 | 0.384905 | -0.35628 | 0.942975 | 1.340908 | 0.700276 | 2.567608 |
| 430 | GCST90274843 | ebi-a-GCST90018865 | TNF-related apoptosis-inducing ligand measurement | Weighted mode | 25 | -0.04703 | 0.158596 | 0.769387 | -0.35787 | 0.263822 | 0.954062 | 0.699161 | 1.301896 |
| 431 | GCST90274844 | ebi-a-GCST90018865 | TNF-related activation-induced cytokine measurement | MR Egger | 29 | -0.36739 | 0.250497 | 0.154019 | -0.85837 | 0.123579 | 0.692537 | 0.423853 | 1.13154 |
| 432 | GCST90274844 | ebi-a-GCST90018865 | TNF-related activation-induced cytokine measurement | Weighted median | 29 | -0.17632 | 0.179057 | 0.324774 | -0.52727 | 0.174635 | 0.838353 | 0.590216 | 1.190811 |
| 433 | GCST90274844 | ebi-a-GCST90018865 | TNF-related activation-induced cytokine measurement | Inverse variance weighted | 29 | -0.25459 | 0.122074 | 0.037022 | -0.49385 | -0.01532 | 0.775235 | 0.61027 | 0.984794 |
| 434 | GCST90274844 | ebi-a-GCST90018865 | TNF-related activation-induced cytokine measurement | Simple mode | 29 | 0.187142 | 0.334324 | 0.580093 | -0.46813 | 0.842417 | 1.205798 | 0.62617 | 2.321974 |
| 435 | GCST90274844 | ebi-a-GCST90018865 | TNF-related activation-induced cytokine measurement | Weighted mode | 29 | -0.06573 | 0.223681 | 0.771021 | -0.50415 | 0.37268 | 0.93638 | 0.60402 | 1.45162 |
| 436 | GCST90274845 | ebi-a-GCST90018865 | thymic stromal lymphopoietin measurement | MR Egger | 15 | 0.340498 | 0.591829 | 0.574891 | -0.81949 | 1.500483 | 1.405647 | 0.440657 | 4.483856 |
| 437 | GCST90274845 | ebi-a-GCST90018865 | thymic stromal lymphopoietin measurement | Weighted median | 15 | 0.386344 | 0.334477 | 0.248062 | -0.26923 | 1.041919 | 1.471591 | 0.763967 | 2.834651 |
| 438 | GCST90274845 | ebi-a-GCST90018865 | thymic stromal lymphopoietin measurement | Inverse variance weighted | 15 | 0.50099 | 0.268524 | 0.062081 | -0.02532 | 1.027296 | 1.650354 | 0.975001 | 2.793503 |
| 439 | GCST90274845 | ebi-a-GCST90018865 | thymic stromal lymphopoietin measurement | Simple mode | 15 | 0.366035 | 0.648571 | 0.581431 | -0.90516 | 1.637235 | 1.442006 | 0.404475 | 5.140937 |
| 440 | GCST90274845 | ebi-a-GCST90018865 | thymic stromal lymphopoietin measurement | Weighted mode | 15 | 0.218075 | 0.546898 | 0.696094 | -0.85384 | 1.289995 | 1.24368 | 0.425775 | 3.632768 |
| 441 | GCST90274846 | ebi-a-GCST90018865 | tumor necrosis factor ligand superfamily member 12 measurement | MR Egger | 29 | 0.489412 | 0.352789 | 0.176706 | -0.20205 | 1.180878 | 1.631357 | 0.817051 | 3.257233 |
| 442 | GCST90274846 | ebi-a-GCST90018865 | tumor necrosis factor ligand superfamily member 12 measurement | Weighted median | 29 | 0.111735 | 0.212075 | 0.598285 | -0.30393 | 0.527402 | 1.118217 | 0.737912 | 1.694524 |
| 443 | GCST90274846 | ebi-a-GCST90018865 | tumor necrosis factor ligand superfamily member 12 measurement | Inverse variance weighted | 29 | 0.110509 | 0.152122 | 0.467566 | -0.18765 | 0.408669 | 1.116846 | 0.828904 | 1.504813 |
| 444 | GCST90274846 | ebi-a-GCST90018865 | tumor necrosis factor ligand superfamily member 12 measurement | Simple mode | 29 | -0.02941 | 0.353135 | 0.934217 | -0.72155 | 0.662733 | 0.971017 | 0.485996 | 1.940087 |
| 445 | GCST90274846 | ebi-a-GCST90018865 | tumor necrosis factor ligand superfamily member 12 measurement | Weighted mode | 29 | 0.060307 | 0.263438 | 0.820592 | -0.45603 | 0.576646 | 1.062163 | 0.633794 | 1.780058 |
| 446 | GCST90274847 | ebi-a-GCST90018865 | urokinase-type plasminogen activator measurement | MR Egger | 24 | -0.25982 | 0.375892 | 0.49666 | -0.99657 | 0.476926 | 0.771188 | 0.369143 | 1.611115 |
| 447 | GCST90274847 | ebi-a-GCST90018865 | urokinase-type plasminogen activator measurement | Weighted median | 24 | -0.13011 | 0.206703 | 0.52906 | -0.53525 | 0.275031 | 0.878001 | 0.585526 | 1.316571 |
| 448 | GCST90274847 | ebi-a-GCST90018865 | urokinase-type plasminogen activator measurement | Inverse variance weighted | 24 | 0.058559 | 0.184825 | 0.75137 | -0.3037 | 0.420815 | 1.060307 | 0.738084 | 1.523203 |
| 449 | GCST90274847 | ebi-a-GCST90018865 | urokinase-type plasminogen activator measurement | Simple mode | 24 | -0.32231 | 0.342297 | 0.356179 | -0.99321 | 0.348592 | 0.724474 | 0.370386 | 1.417071 |
| 450 | GCST90274847 | ebi-a-GCST90018865 | urokinase-type plasminogen activator measurement | Weighted mode | 24 | -0.19584 | 0.230512 | 0.404312 | -0.64764 | 0.255963 | 0.822144 | 0.523278 | 1.291705 |
| 451 | GCST90274848 | ebi-a-GCST90018865 | vascular endothelial growth factor A measurement | MR Egger | 25 | 0.133112 | 0.187045 | 0.483832 | -0.2335 | 0.49972 | 1.142378 | 0.791761 | 1.64826 |
| 452 | GCST90274848 | ebi-a-GCST90018865 | vascular endothelial growth factor A measurement | Weighted median | 25 | 0.087798 | 0.115981 | 0.449047 | -0.13952 | 0.31512 | 1.091767 | 0.869772 | 1.370424 |
| 453 | GCST90274848 | ebi-a-GCST90018865 | vascular endothelial growth factor A measurement | Inverse variance weighted | 25 | 0.092072 | 0.119134 | 0.439614 | -0.14143 | 0.325576 | 1.096444 | 0.868115 | 1.384828 |
| 454 | GCST90274848 | ebi-a-GCST90018865 | vascular endothelial growth factor A measurement | Simple mode | 25 | -0.37404 | 0.445822 | 0.409758 | -1.24785 | 0.499774 | 0.687952 | 0.287122 | 1.648349 |
| 455 | GCST90274848 | ebi-a-GCST90018865 | vascular endothelial growth factor A measurement | Weighted mode | 25 | 0.058658 | 0.114401 | 0.612818 | -0.16557 | 0.282884 | 1.060413 | 0.847413 | 1.326951 |

**Supplementary Table 5.** The significance results between 91 circulating inflammatory proteins and ITP

|  | method | id.exposure.x | id.outcome.x | outcome.x | exposure.x | nsnp | b | se | pval | lo_ci | up_ci | or | or_lci95 | or_uci95 | id.exposure.y | id.outcome.y | outcome.y | exposure.y | Q | Q_df | Q_pval | egger_intercept | pval |
| --- | --- | --- | --- | --- | --- | --- | --- | --- | --- | --- | --- | --- | --- | --- | --- | --- | --- | --- | --- | --- | --- | --- | --- |
| 1 | Inverse variance weighted | GCST90274770 | ebi-a-GCST90018865 | Idiopathic thrombocytopenic purpura \|\| id:ebi-a-GCST90018865 | C-C motif chemokine 4-like measurement | 19 | 0.211445 | 0.104723 | 0.043478 | 0.006188 | 0.416702 | 1.235462 | 1.006208 | 1.516951 | GCST90274770 | ebi-a-GCST90018865 | Idiopathic thrombocytopenic purpura \|\| id:ebi-a-GCST90018865 | C-C motif chemokine 4-like measurement | 19.26333 | 18 | 0.375779 | 0.047045 | 0.124559 |
| 2 | MR Egger | GCST90274770 | ebi-a-GCST90018865 | Idiopathic thrombocytopenic purpura \|\| id:ebi-a-GCST90018865 | C-C motif chemokine 4-like measurement | 19 | 0.042826 | 0.145392 | 0.7719 | -0.24214 | 0.327795 | 1.043756 | 0.784944 | 1.387905 | GCST90274770 | ebi-a-GCST90018865 | Idiopathic thrombocytopenic purpura \|\| id:ebi-a-GCST90018865 | C-C motif chemokine 4-like measurement | 16.65277 | 17 | 0.478121 | 0.047045 | 0.124559 |
| 3 | Simple mode | GCST90274770 | ebi-a-GCST90018865 | Idiopathic thrombocytopenic purpura \|\| id:ebi-a-GCST90018865 | C-C motif chemokine 4-like measurement | 19 | 0.068322 | 0.298476 | 0.821524 | -0.51669 | 0.653335 | 1.07071 | 0.596491 | 1.92194 | NA | NA | NA | NA | NA | NA | NA | 0.047045 | 0.124559 |
| 4 | Weighted median | GCST90274770 | ebi-a-GCST90018865 | Idiopathic thrombocytopenic purpura \|\| id:ebi-a-GCST90018865 | C-C motif chemokine 4-like measurement | 19 | 0.1627 | 0.132331 | 0.21889 | -0.09667 | 0.422069 | 1.176683 | 0.907855 | 1.525114 | NA | NA | NA | NA | NA | NA | NA | 0.047045 | 0.124559 |
| 5 | Weighted mode | GCST90274770 | ebi-a-GCST90018865 | Idiopathic thrombocytopenic purpura \|\| id:ebi-a-GCST90018865 | C-C motif chemokine 4-like measurement | 19 | 0.153604 | 0.116061 | 0.202242 | -0.07388 | 0.381083 | 1.166029 | 0.928787 | 1.463868 | NA | NA | NA | NA | NA | NA | NA | 0.047045 | 0.124559 |
| 6 | Inverse variance weighted | GCST90274784 | ebi-a-GCST90018865 | Idiopathic thrombocytopenic purpura \|\| id:ebi-a-GCST90018865 | C-X-C motif chemokine 9 measurement | 22 | 0.597022 | 0.179663 | 0.000891 | 0.244882 | 0.949162 | 1.816701 | 1.277471 | 2.583544 | GCST90274784 | ebi-a-GCST90018865 | Idiopathic thrombocytopenic purpura \|\| id:ebi-a-GCST90018865 | C-X-C motif chemokine 9 measurement | 15.2965 | 21 | 0.807786 | -0.03365 | 0.427427 |
| 7 | MR Egger | GCST90274784 | ebi-a-GCST90018865 | Idiopathic thrombocytopenic purpura \|\| id:ebi-a-GCST90018865 | C-X-C motif chemokine 9 measurement | 22 | 0.90458 | 0.420027 | 0.043649 | 0.081328 | 1.727832 | 2.470894 | 1.084727 | 5.628441 | GCST90274784 | ebi-a-GCST90018865 | Idiopathic thrombocytopenic purpura \|\| id:ebi-a-GCST90018865 | C-X-C motif chemokine 9 measurement | 14.64026 | 20 | 0.796609 | -0.03365 | 0.427427 |
| 8 | Simple mode | GCST90274784 | ebi-a-GCST90018865 | Idiopathic thrombocytopenic purpura \|\| id:ebi-a-GCST90018865 | C-X-C motif chemokine 9 measurement | 22 | 0.617251 | 0.49475 | 0.225911 | -0.35246 | 1.586962 | 1.853825 | 0.702957 | 4.888875 | NA | NA | NA | NA | NA | NA | NA | -0.03365 | 0.427427 |
| 9 | Weighted median | GCST90274784 | ebi-a-GCST90018865 | Idiopathic thrombocytopenic purpura \|\| id:ebi-a-GCST90018865 | C-X-C motif chemokine 9 measurement | 22 | 0.627545 | 0.244483 | 0.010263 | 0.148359 | 1.106731 | 1.873007 | 1.159929 | 3.024455 | NA | NA | NA | NA | NA | NA | NA | -0.03365 | 0.427427 |
| 10 | Weighted mode | GCST90274784 | ebi-a-GCST90018865 | Idiopathic thrombocytopenic purpura \|\| id:ebi-a-GCST90018865 | C-X-C motif chemokine 9 measurement | 22 | 0.607164 | 0.419889 | 0.162935 | -0.21582 | 1.430147 | 1.835219 | 0.805881 | 4.179314 | NA | NA | NA | NA | NA | NA | NA | -0.03365 | 0.427427 |
| 11 | Inverse variance weighted | GCST90274798 | ebi-a-GCST90018865 | Idiopathic thrombocytopenic purpura \|\| id:ebi-a-GCST90018865 | interleukin-12 subunit B measurement | 27 | 0.329199 | 0.090193 | 0.000262 | 0.15242 | 0.505977 | 1.389854 | 1.164649 | 1.658605 | GCST90274798 | ebi-a-GCST90018865 | Idiopathic thrombocytopenic purpura \|\| id:ebi-a-GCST90018865 | interleukin-12 subunit B measurement | 32.15777 | 26 | 0.187956 | -0.01099 | 0.638795 |
| 12 | MR Egger | GCST90274798 | ebi-a-GCST90018865 | Idiopathic thrombocytopenic purpura \|\| id:ebi-a-GCST90018865 | interleukin-12 subunit B measurement | 27 | 0.379797 | 0.140441 | 0.012137 | 0.104532 | 0.655062 | 1.461988 | 1.110191 | 1.925262 | GCST90274798 | ebi-a-GCST90018865 | Idiopathic thrombocytopenic purpura \|\| id:ebi-a-GCST90018865 | interleukin-12 subunit B measurement | 31.86995 | 25 | 0.16181 | -0.01099 | 0.638795 |
| 13 | Simple mode | GCST90274798 | ebi-a-GCST90018865 | Idiopathic thrombocytopenic purpura \|\| id:ebi-a-GCST90018865 | interleukin-12 subunit B measurement | 27 | 0.10586 | 0.286728 | 0.714967 | -0.45613 | 0.667847 | 1.111667 | 0.633734 | 1.950034 | NA | NA | NA | NA | NA | NA | NA | -0.01099 | 0.638795 |
| 14 | Weighted median | GCST90274798 | ebi-a-GCST90018865 | Idiopathic thrombocytopenic purpura \|\| id:ebi-a-GCST90018865 | interleukin-12 subunit B measurement | 27 | 0.369542 | 0.109201 | 0.000714 | 0.155507 | 0.583577 | 1.447072 | 1.168251 | 1.792438 | NA | NA | NA | NA | NA | NA | NA | -0.01099 | 0.638795 |
| 15 | Weighted mode | GCST90274798 | ebi-a-GCST90018865 | Idiopathic thrombocytopenic purpura \|\| id:ebi-a-GCST90018865 | interleukin-12 subunit B measurement | 27 | 0.325396 | 0.102457 | 0.003824 | 0.12458 | 0.526212 | 1.384579 | 1.132672 | 1.692509 | NA | NA | NA | NA | NA | NA | NA | -0.01099 | 0.638795 |
| 16 | Inverse variance weighted | GCST90274805 | ebi-a-GCST90018865 | Idiopathic thrombocytopenic purpura \|\| id:ebi-a-GCST90018865 | interleukin-1 alpha measurement | 14 | -0.373 | 0.171455 | 0.029594 | -0.70905 | -0.03694 | 0.688667 | 0.492112 | 0.963729 | GCST90274805 | ebi-a-GCST90018865 | Idiopathic thrombocytopenic purpura \|\| id:ebi-a-GCST90018865 | interleukin-1 alpha measurement | 5.323995 | 13 | 0.967351 | -0.0077 | 0.855201 |
| 17 | MR Egger | GCST90274805 | ebi-a-GCST90018865 | Idiopathic thrombocytopenic purpura \|\| id:ebi-a-GCST90018865 | interleukin-1 alpha measurement | 14 | -0.32405 | 0.313524 | 0.321711 | -0.93856 | 0.290453 | 0.723211 | 0.39119 | 1.337033 | GCST90274805 | ebi-a-GCST90018865 | Idiopathic thrombocytopenic purpura \|\| id:ebi-a-GCST90018865 | interleukin-1 alpha measurement | 5.289228 | 12 | 0.947614 | -0.0077 | 0.855201 |
| 18 | Simple mode | GCST90274805 | ebi-a-GCST90018865 | Idiopathic thrombocytopenic purpura \|\| id:ebi-a-GCST90018865 | interleukin-1 alpha measurement | 14 | -0.4851 | 0.36805 | 0.210246 | -1.20648 | 0.236276 | 0.615635 | 0.299249 | 1.266523 | NA | NA | NA | NA | NA | NA | NA | -0.0077 | 0.855201 |
| 19 | Weighted median | GCST90274805 | ebi-a-GCST90018865 | Idiopathic thrombocytopenic purpura \|\| id:ebi-a-GCST90018865 | interleukin-1 alpha measurement | 14 | -0.28593 | 0.223888 | 0.201559 | -0.72475 | 0.152888 | 0.751313 | 0.484444 | 1.165195 | NA | NA | NA | NA | NA | NA | NA | -0.0077 | 0.855201 |
| 20 | Weighted mode | GCST90274805 | ebi-a-GCST90018865 | Idiopathic thrombocytopenic purpura \|\| id:ebi-a-GCST90018865 | interleukin-1 alpha measurement | 14 | -0.16011 | 0.272002 | 0.566193 | -0.69323 | 0.373014 | 0.85205 | 0.499957 | 1.452104 | NA | NA | NA | NA | NA | NA | NA | -0.0077 | 0.855201 |
| 21 | Inverse variance weighted | GCST90274833 | ebi-a-GCST90018865 | Idiopathic thrombocytopenic purpura \|\| id:ebi-a-GCST90018865 | stem Cell Factor measurement | 33 | 0.240561 | 0.108595 | 0.026746 | 0.027714 | 0.453407 | 1.271962 | 1.028102 | 1.573665 | GCST90274833 | ebi-a-GCST90018865 | Idiopathic thrombocytopenic purpura \|\| id:ebi-a-GCST90018865 | stem Cell Factor measurement | 29.51498 | 32 | 0.592915 | -0.03608 | 0.118141 |
| 22 | MR Egger | GCST90274833 | ebi-a-GCST90018865 | Idiopathic thrombocytopenic purpura \|\| id:ebi-a-GCST90018865 | stem Cell Factor measurement | 33 | 0.50644 | 0.197885 | 0.01559 | 0.118585 | 0.894295 | 1.659373 | 1.125902 | 2.445612 | GCST90274833 | ebi-a-GCST90018865 | Idiopathic thrombocytopenic purpura \|\| id:ebi-a-GCST90018865 | stem Cell Factor measurement | 26.93176 | 31 | 0.675597 | -0.03608 | 0.118141 |
| 23 | Simple mode | GCST90274833 | ebi-a-GCST90018865 | Idiopathic thrombocytopenic purpura \|\| id:ebi-a-GCST90018865 | stem Cell Factor measurement | 33 | 0.324123 | 0.2461 | 0.197179 | -0.15823 | 0.806479 | 1.382818 | 0.853652 | 2.240006 | NA | NA | NA | NA | NA | NA | NA | -0.03608 | 0.118141 |
| 24 | Weighted median | GCST90274833 | ebi-a-GCST90018865 | Idiopathic thrombocytopenic purpura \|\| id:ebi-a-GCST90018865 | stem Cell Factor measurement | 33 | 0.272286 | 0.164228 | 0.097322 | -0.0496 | 0.594173 | 1.312963 | 0.951609 | 1.811533 | NA | NA | NA | NA | NA | NA | NA | -0.03608 | 0.118141 |
| 25 | Weighted mode | GCST90274833 | ebi-a-GCST90018865 | Idiopathic thrombocytopenic purpura \|\| id:ebi-a-GCST90018865 | stem Cell Factor measurement | 33 | 0.305392 | 0.166504 | 0.075946 | -0.02095 | 0.63174 | 1.357157 | 0.979263 | 1.88088 | NA | NA | NA | NA | NA | NA | NA | -0.03608 | 0.118141 |
| 26 | Inverse variance weighted | GCST90274844 | ebi-a-GCST90018865 | Idiopathic thrombocytopenic purpura \|\| id:ebi-a-GCST90018865 | TNF-related activation-induced cytokine measurement | 29 | -0.25459 | 0.122074 | 0.037022 | -0.49385 | -0.01532 | 0.775235 | 0.61027 | 0.984794 | GCST90274844 | ebi-a-GCST90018865 | Idiopathic thrombocytopenic purpura \|\| id:ebi-a-GCST90018865 | TNF-related activation-induced cytokine measurement | 17.68085 | 28 | 0.933904 | 0.015637 | 0.610254 |
| 27 | MR Egger | GCST90274844 | ebi-a-GCST90018865 | Idiopathic thrombocytopenic purpura \|\| id:ebi-a-GCST90018865 | TNF-related activation-induced cytokine measurement | 29 | -0.36739 | 0.250497 | 0.154019 | -0.85837 | 0.123579 | 0.692537 | 0.423853 | 1.13154 | GCST90274844 | ebi-a-GCST90018865 | Idiopathic thrombocytopenic purpura \|\| id:ebi-a-GCST90018865 | TNF-related activation-induced cytokine measurement | 17.41489 | 27 | 0.920387 | 0.015637 | 0.610254 |
| 28 | Simple mode | GCST90274844 | ebi-a-GCST90018865 | Idiopathic thrombocytopenic purpura \|\| id:ebi-a-GCST90018865 | TNF-related activation-induced cytokine measurement | 29 | 0.187142 | 0.332171 | 0.577654 | -0.46391 | 0.838196 | 1.205798 | 0.628818 | 2.312192 | NA | NA | NA | NA | NA | NA | NA | 0.015637 | 0.610254 |
| 29 | Weighted median | GCST90274844 | ebi-a-GCST90018865 | Idiopathic thrombocytopenic purpura \|\| id:ebi-a-GCST90018865 | TNF-related activation-induced cytokine measurement | 29 | -0.17632 | 0.17939 | 0.325675 | -0.52792 | 0.175289 | 0.838353 | 0.58983 | 1.19159 | NA | NA | NA | NA | NA | NA | NA | 0.015637 | 0.610254 |
| 30 | Weighted mode | GCST90274844 | ebi-a-GCST90018865 | Idiopathic thrombocytopenic purpura \|\| id:ebi-a-GCST90018865 | TNF-related activation-induced cytokine measurement | 29 | -0.06573 | 0.216308 | 0.763457 | -0.4897 | 0.358229 | 0.93638 | 0.612812 | 1.430793 | NA | NA | NA | NA | NA | NA | NA | 0.015637 | 0.610254 |
